# Moxibustion combinations for diabetic peripheral neuropathy: a network meta-analysis of 40 randomised trials

**DOI:** 10.3389/fendo.2026.1941999

**Published:** 2026-09-03

**Authors:** Dongmin Du, Shurong Wang, Xiaoyu Chen, Jiahui Wang, Xin Zhang, Yue Gao

**Affiliations:** 1 Heilongjiang University of Chinese Medicine, Harbin, China; 2 Heilongjiang University of Chinese Medicine Affiliated Second Hospital, Harbin, China

**Keywords:** diabetic peripheral neuropathy, external herbal medicine, internal herbal medicine, moxibustion, network meta-analysis

## Abstract

**Objective:**

Moxibustion, an external treatment used in traditional Chinese medicine, is increasingly applied to diabetic peripheral neuropathy (DPN). We compared the effectiveness of the principal moxibustion-containing treatment combinations for this condition. We searched for randomised controlled trials (RCTs) available by 14 April 2026. Sources were PubMed, EMBASE, the Cochrane Library, Web of Science, China National Knowledge Infrastructure (CNKI), Wanfang Medical Database and VIP Database. The resulting network meta-analysis (NMA) covered moxibustion (MOXI) and combinations with acupuncture therapy (ACU), massage therapy (MT), internal herbal medicine (IHM), external herbal medicine (EHM) or conventional treatment (CT). Outcomes included common peroneal and median motor nerve conduction velocity (MNCV), sensory nerve conduction velocity (SNCV), Toronto Clinical Scoring System (TCSS) score and markedly effective rate. Random-effects models in Stata MP 17.0 generated mean differences (MDs) and risk ratios (RRs). Evidence certainty was graded with the Grading of Recommendations Assessment, Development and Evaluation (GRADE) framework.

**Results:**

Forty studies involving 3,718 participants were included. Moderate-certainty evidence favoured MOXI+EHM over CT for common peroneal MNCV (MD = 6.14 m/s, 95% CI 3.82 to 8.46). Evidence also favoured MOXI+EHM for median MNCV (MD = 6.35 m/s, 95% CI 2.35 to 10.36). The corresponding estimate for common peroneal SNCV was MD = 8.44 m/s (95% CI 4.80 to 12.08). For median SNCV, MOXI+ACU outperformed CT (MD = 6.61 m/s, 95% CI 1.29 to 11.92). MOXI+EHM showed a similar effect (MD = 4.32 m/s, 95% CI 1.01 to 7.63). Certainty for this outcome ranged from moderate to very low. Moderate-certainty evidence also showed that MOXI+EHM reduced TCSS scores (MD=-2.55 points, 95% CI -3.81 to -1.30). MOXI+EHM (RR = 1.74, 95% CI 1.27 to 2.40) and MOXI+ACU (RR = 1.59, 95% CI 1.05 to 2.41) increased the markedly effective rate versus CT.

**Conclusion:**

Moderate-certainty evidence suggests that MOXI+EHM may improve nerve conduction, clinical symptoms and signs, and the markedly effective rate in patients with DPN. Moxibustion-based combination therapy may therefore offer a useful adjunct to conservative DPN management, although the findings require confirmation in rigorous trials.

**Systematic review registration:**

https://www.crd.york.ac.uk/PROSPERO/view/CRD420261444059, identifier CRD420261444059.

## 1 Introduction

Diabetic peripheral neuropathy (DPN) is a frequent, long-term consequence of diabetes. Its predominant presentation, distal symmetric polyneuropathy, produces bilateral distal pain, burning, numbness and paraesthesia. Progressive sensory loss can impair balance, mobility and quality of life. Management must therefore integrate glycaemic control, pain relief and preservation of function (1).

Conservative management of DPN combines optimisation of glycaemia and metabolic risk factors with neuropathic pain control, foot care and exercise (2). Treatment response varies with diabetes duration, metabolic control, nerve damage, comorbidities, tolerability and adherence (1). Multimodal treatment is therefore common in clinical practice. However, comparative evidence remains insufficient to determine which interventions should be combined and which combinations offer the greatest benefit.

Moxibustion is an external therapy in traditional Chinese medicine that has increasingly been used for DPN and may improve symptoms and nerve function (3). In traditional Chinese medicine, DPN is commonly classified as bi syndrome or blood bi. It is attributed to prolonged wasting-thirst, deficiencies of qi, yin or yang, and dysfunction of the liver, spleen and kidney. The resulting pattern combines underlying deficiency with blood stasis, phlegm and obstruction of the collaterals, impairing nourishment of the sinews and causing numbness and pain (4).

The thermal stimulus of moxibustion may activate polymodal receptors, sensory input and axon reflexes. These responses may regulate local blood flow, neuropeptide release, the tissue microenvironment and neural sensitisation (5). Acupuncture may influence neurotrophic factors and support peripheral nerve repair and regeneration (4). Internal and external herbal medicines may reduce inflammation, oxidative stress and apoptosis while improving mitochondrial function and glucose-lipid metabolism (6). Massage may improve local microcirculation, neural oxygenation, metabolite clearance, pain thresholds and joint mobility (7). These distinct actions provide a rationale for combining moxibustion with acupuncture, massage or herbal medicine, although synergistic effects remain unproven.

Previous pairwise meta-analysis found that moxibustion combined with Western medicine was more effective than Western medicine alone for DPN (3). However, direct comparisons among different moxibustion-based combinations were unavailable. Consequently, the relative effectiveness of moxibustion combined with acupuncture, massage, internal herbal medicine or external herbal medicine remains uncertain.

Moxibustion techniques differ in materials, procedures and stimulation intensity, which may influence their implementation and effects (8). We therefore compared warm-needle moxibustion, mild moxibustion, heat-sensitive moxibustion, thunder-fire moxibustion, ginger-partitioned moxibustion and warming-device moxibustion.

This network meta-analysis (NMA) compared compared the effectiveness and safety of moxibustion-based combination therapies for DPN at two complementary levels. The class-level analysis treated moxibustion techniques as one intervention class and ranked moxibustion-containing combinations within the overall network. The technique-level analysis separated the six moxibustion techniques to examine their relative effects and potential sources of variation.

Primary outcomes were common peroneal motor nerve conduction velocity (MNCV), median MNCV, common peroneal sensory nerve conduction velocity (SNCV), median SNCV, Toronto Clinical Scoring System (TCSS) score and markedly effective rate. Secondary outcomes were fasting plasma glucose (FPG) and 2-h postprandial glucose (2hPG). Treatment rankings were interpreted alongside certainty-of-evidence assessments to inform clinical decision-making and future research.

## 2 Methods

### 2.1 Materials and methods

This NMA was reported in accordance with the Preferred Reporting Items for Systematic Reviews and Meta-Analyses (PRISMA) extension for network meta-analyses (**Supplementary Table 1**) (9). This review was registered in PROSPERO (CRD420261444059).

### 2.2 Data sources and search strategy

Seven databases were searched from inception through 14 April 2026: PubMed, EMBASE, the Cochrane Library, Web of Science, China National Knowledge Infrastructure (CNKI), Wanfang Medical Database and VIP Database. No language filter was applied. Controlled and free-text terms covered ‘Diabetic Neuropathy’, ‘Neuropathies, Diabetic’, ‘Diabetic Amyotrophy’, ‘Moxibustion’, ‘heat-sensitive moxibustion’, ‘thunder-fire moxibustion’, ‘warm-needling moxibustion’ and ‘randomized controlled trial’ (**Supplementary Table 2**).

### 2.3 Eligibility criteria

#### 2.3.1 Inclusion criteria

1. Population (P): Randomised controlled trials (RCTs) enrolling adults with DPN diagnosed according to Chinese or Western medical criteria were eligible, irrespective of sex, diabetes type or DPN severity. Eligible studies used the integrated disease-and-syndrome guideline for DPN (10) or the 2016 traditional Chinese medicine clinical guideline for DPN (11).
2. Intervention (I): Randomized controlled trials evaluating moxibustion alone or in combination with adjunctive therapies were eligible for inclusion. Moxibustion was classified by technique into six categories: warm-needle moxibustion (WN), mild moxibustion (MM), heat-sensitive moxibustion (HM), thunder-fire moxibustion (TF), ginger-partitioned moxibustion (GIM), and warming-device moxibustion (WDM). In the primary analysis, all moxibustion techniques were grouped into a single MOXI node, whereas in subgroup analyses, each technique was treated as a separate node. Adjunctive therapies were classified by treatment modality. Acupuncture, thumbtack needle therapy, and acupoint catgut embedding were grouped as acupuncture therapy (ACU); acupoint massage and foot care as massage therapy (MT); Chinese herbal foot baths, Chinese herbal fumigation and washing, acupoint application, transdermal herbal preparations applied to acupoints, and targeted transdermal delivery of Chinese herbal medicine as external herbal medicine (EHM); and Huangqi Guizhi Wuwu Decoction and its modified formulations, Tongxinluo Capsule, Huazhuo Tongluo Formula, Jingluoshu Capsule, Fu’s Tongbi Decoction, Yiqi Ziyin Formula, Yiqi Yangyin Decoction, and Buyang Huanwu Decoction as internal herbal medicine (IHM).
3. Comparator (C): Eligible control groups received conventional DPN treatment. Health education, psychological support, appropriate exercise, dietary management, glycaemic control and conventional pharmacotherapy were grouped as CT.
4. Outcomes (O): Trials reported at least one prespecified outcome. TCSS assesses DPN presence and severity using symptoms, tendon reflexes and sensory examination (12). Common peroneal MNCV and median MNCV assess motor nerve conduction and reflect peripheral motor nerve dysfunction and recovery (13, 14). Common peroneal SNCV and median SNCV assess sensory nerve conduction and reflect peripheral sensory dysfunction (15, 16). FPG assesses fasting glycaemic control (17, 18). The 2hPG outcome assesses postprandial glycaemic control (17, 18). One markedly effective criterion was a nerve-conduction velocity increase of at least 5 m/s (19). The alternative criterion was a clear improvement in traditional Chinese medicine symptoms and signs with a syndrome-score reduction of at least 70% (20).
5. Study design (S): Only RCTs were eligible, with no language restrictions.

#### 2.3.2 Exclusion criteria

1. RCTs with insufficient outcome data for quantitative synthesis when the required information could not be derived from the available data, including studies that failed to report standard deviations or other measures of variability for continuous outcomes, and studies that used unvalidated measurement scales or instruments without established validity.
2. reviews and case reports

Two reviewers first assessed titles and abstracts, then re-examined potentially eligible RCTs and retained the latest published dataset.

### 2.4 Data extraction and risk-of-bias assessment

Two reviewers independently extracted data under PRISMA procedures, consulting another reviewer to resolve disagreements. Recorded items were first author, year, sample size, age, sex, location, follow-up and intervention details for each group. Change-from-baseline means and standard deviations (SDs) were preferred for continuous variables. If only baseline and endpoint statistics were reported, mean change was calculated by subtraction. Its SD was derived from the two reported SDs and correlation coefficient R using the following formula:


$$SD_{change}=\sqrt{Baseline\;SD^{2}+Endpoint\;SD^{2}-2R\cdot Baseline\;SD\cdot Endpoint\;SD}$$

When R could not be inferred, R = 0.5 was assumed in the primary analysis. Sensitivity analyses used R = 0.25 and R = 0.75. Event counts and group totals were extracted for dichotomous outcomes.

Risk of bias was assessed using the Cochrane Risk of Bias 2 (RoB 2) tool. The five domains covered randomisation, deviations from intended interventions, missing outcome data, outcome measurement and selective reporting. Each domain and the overall study were judged as low risk, some concerns or high risk (21).

### 2.5 Statistical analysis

Analyses were conducted with Stata MP 17.0. Mean differences (MDs) with 95% confidence intervals (CIs) summarised continuous outcomes sharing a scale; risk ratios (RRs) with 95% CIs summarised dichotomous outcomes. Multi-arm trials were represented by augmented pairwise comparisons that preserved within-study correlations, preventing underestimated standard errors. For dichotomous outcomes, when any treatment arm had zero events or zero non-events, a continuity correction was applied to all arms within that study before effect-size estimation (r = r + 0.5, n = n + 1) to avoid infinite RR estimates. Studies with zero events in all comparison arms (double-zero studies) were excluded from the relative-effect analysis for the corresponding outcome because they provide no information on the direction or magnitude of relative effects such as the RR.Random-effects consistency models formed the primary analyses, and restricted maximum likelihood estimated between-study variance (τ²). For networks containing closed loops, global inconsistency was assessed using the design-by-treatment interaction model, and local inconsistency using node-splitting analysis. Loop-specific inconsistency factors were also calculated to evaluate agreement between direct and indirect evidence within individual loops. A P value <0.05 indicated potential inconsistency. For loop-specific analyses, an inconsistency factor (IF) with a 95% confidence interval CI including 0 was considered to indicate no statistically detectable disagreement between direct and indirect evidence.Network plots were used to depict the geometry of the treatment networks, with node size proportional to the total number of participants assigned to each intervention and edge width proportional to the number of studies contributing direct evidence. Treatment rankings were summarized using the surface under the cumulative ranking curve (SUCRA), probability of being the best treatment (PreBest), and mean rank. For outcomes reported in more than 10 studies, comparison-adjusted funnel plots were examined for publication bias and small-study effects.Leave-one-out sensitivity analyses were performed to assess the robustness of the network estimates. Each study was sequentially excluded and the random-effects consistency model refitted. Robustness was evaluated by determining whether exclusion of any individual study materially changed the direction or magnitude of the relative effects, their 95% CIs, or statistical significance. Univariable network meta-regression tested study-level modifiers and reported coefficients, 95% CIs and Wald-test P values. Effect modification was inferred at P<0.05.

### 2.6 GRADE grading

We determined evidence certainty with the Grading of Recommendations Assessment, Development and Evaluation (GRADE) framework and Confidence in Network Meta-Analysis (CINeMA). RCT evidence began at high certainty, then underwent appraisal for within-study bias, indirectness, imprecision, heterogeneity, incoherence and across-study bias. The CINeMA contribution matrix supplied comparison-level weights for RoB 2 judgements.Evaluation of indirectness focused on transitivity and exchangeability. Baseline severity, treatment intensity and follow-up duration were prespecified modifiers. Minimal important difference (MID) thresholds informed imprecision. We used MD = 1.2 m/s for each nerve-conduction endpoint (22). No established MID is available for TCSS in DPN; therefore, MD = 3 points served as a surrogate based on the approximate difference between neighbouring severity categories (23). Judgements incorporated whether the 95% CI crossed the null and the applicable MID.Heterogeneity judgements considered τ² and whether the prediction interval extended beyond the MID. For closed networks, CINeMA compared direct and indirect estimates through node splitting/SIDE or design-by-treatment approaches. Across-study bias was appraised from registration, grey-literature searching and comparison-adjusted funnel-plot patterns. Each domain was classified as no concerns, some concerns or major concerns. Applying GRADE principles, downgrading by one or two levels produced high, moderate, low or very low certainty.

## 3 Results

### 3.1 Study selection and characteristics

**Figure 1:**
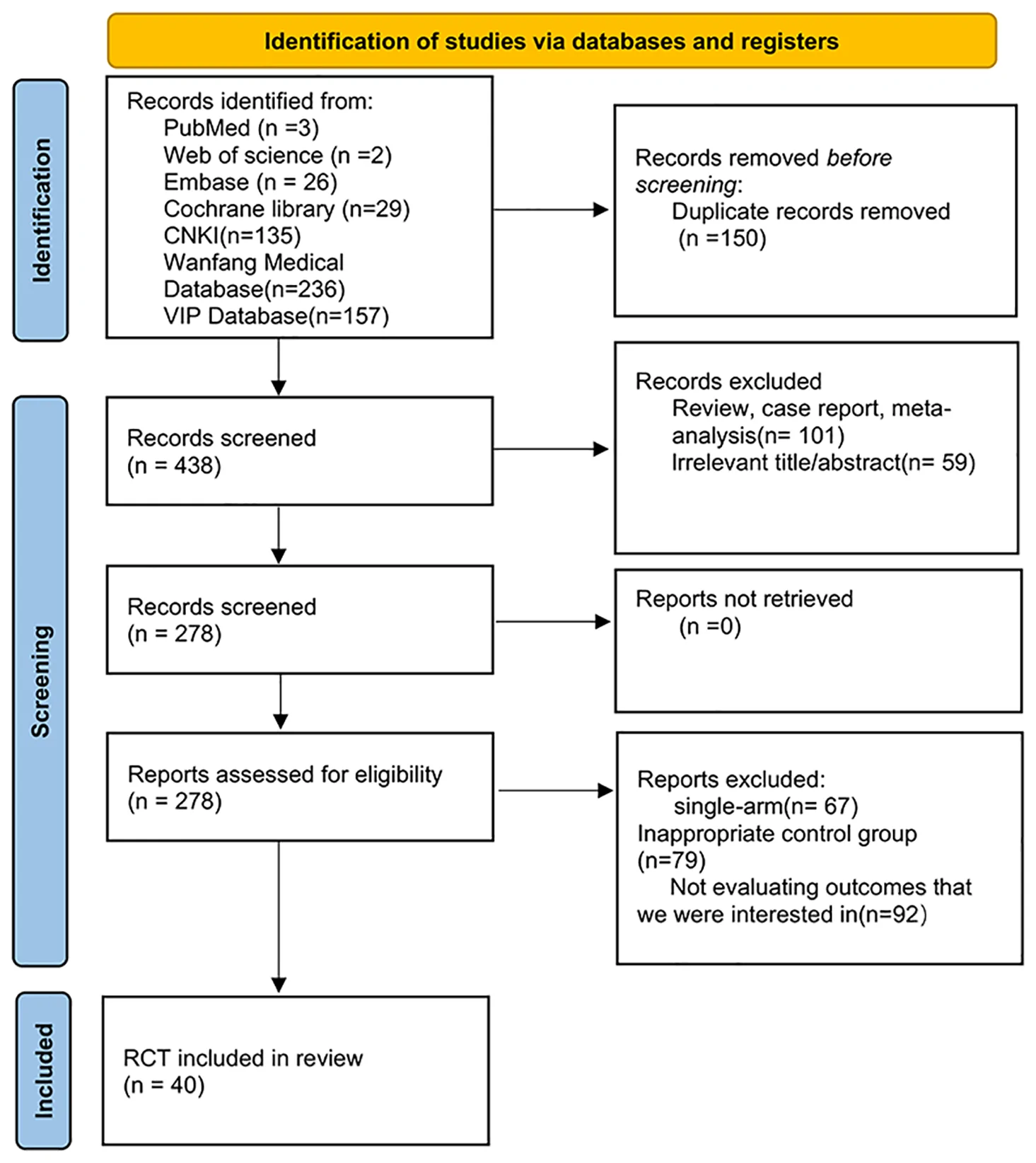
Literature search flow diagram.

Database searching returned 588 records. After removal of duplicates and irrelevant abstracts, 278 reports proceeded to full-text assessment. Of these, 40 studies satisfied eligibility requirements (**Figure 1**; **Supplementary Table 3** References). Across 3,718 participants, the network contained nine intervention categories: MOXI, ACU, EHM, IHM, CT, MOXI+ACU, MOXI+MT, MOXI+EHM and MOXI+IHM. The technique-level network further divided MOXI into TF, WN, HM, MM, GIM and WDM.Treatment courses ranged from 14–28 days for MOXI, 19–21 days for ACU, 14–28 days for EHM, 43 days for IHM and 14–56 days for CT. Corresponding ranges were 28 days for MOXI+ACU, 20–56 days for MOXI+MT, 14–42 days for MOXI+EHM and 28–90 days for MOXI+IHM. Every study was conducted in China. Mean participant age was generally 54 to 68 years, and samples contained more men than women. Follow-up after treatment was usually 2–4 weeks. **Supplementary Tables 4, S5** provide full study details.

### 3.2 Risk of bias

All 40 RCTs were judged to have some concerns overall, and none was judged at high risk of bias (**Supplementary Figure 1**). Thirty-nine studies had some concerns regarding randomisation because several did not report sequence-generation methods. All 40 studies had some concerns regarding deviations from intended interventions because blinding is difficult for traditional Chinese medicine interventions. Missing outcome data were insufficiently reported in 35 studies. Thirty-one studies had some concerns regarding outcome measurement because some outcomes were subjective, and 31 had some concerns regarding selective reporting.

### 3.3 Consistency assessment

**Figure 2:**
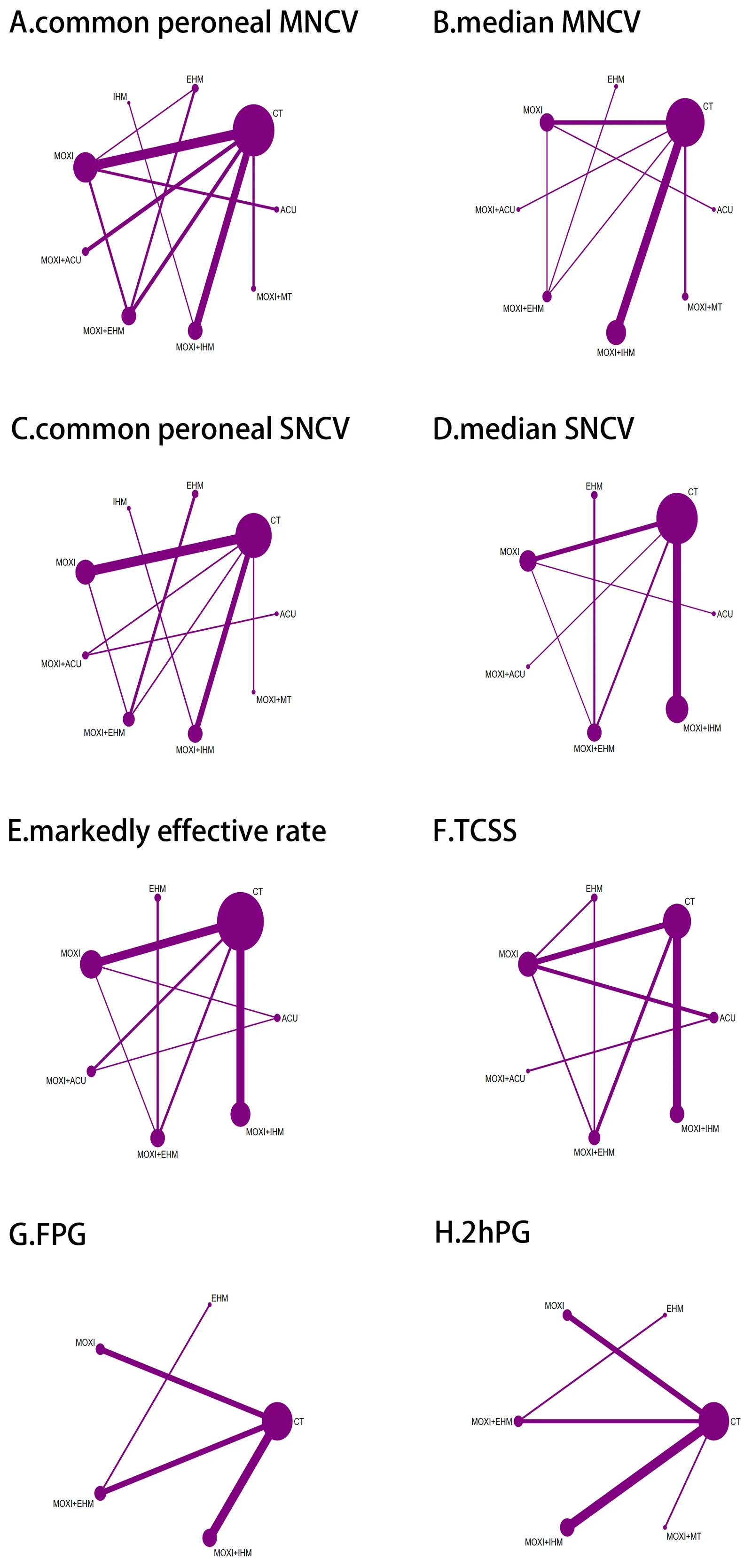
Network diagrams for the primary analyses.

Closed loops were present for common peroneal MNCV, median MNCV, common peroneal SNCV, median SNCV, TCSS and markedly effective rate (**Figure 2**). Global inconsistency tests yielded P>0.05 for all outcomes (**Supplementary Table 6**). Node-splitting analyses also yielded P>0.05 for every comparison (**Supplementary Tables 7–S12**). All loop-specific inconsistency-factor CIs crossed 0 (**Supplementary Tables 13–S18**). The primary NMA therefore used consistency models.

The FPG and 2hPG networks contained no closed loops (**Figure 2**), precluding global, local and loop-specific inconsistency analyses. These outcomes were analysed under random-effects consistency models. Transitivity was assessed by comparing key clinical characteristics. Age and follow-up duration were broadly comparable across studies (**Supplementary Tables 4, S5**). Evidence for these outcomes was downgraded for indirectness because closed loops were absent and transitivity remained uncertain; the results should therefore be interpreted cautiously.

### 3.4 Primary outcomes

#### 3.4.1 Common peroneal MNCV

**Figure 3:**
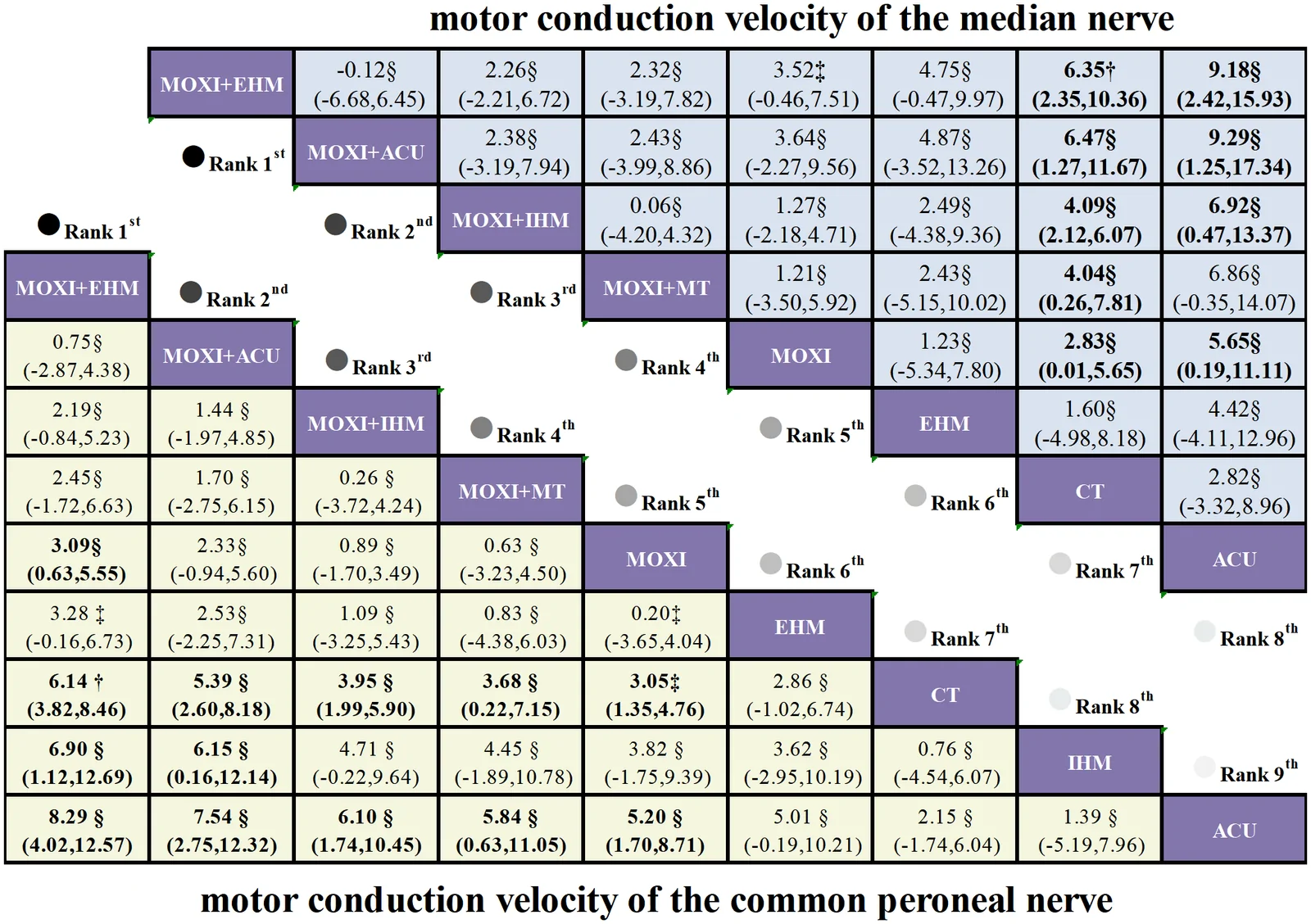
League tables for common peroneal MNCV and median MNCV in the primary analyses. League tables from the network meta-analysis of moxibustion-based combination therapies for DPN, ordered by SUCRA value. Comparisons are read from left to right, with estimates shown at the intersection of the column and row treatments. Continuous outcomes are reported as MDs with 95% CIs. In the lower-left triangle (common peroneal MNCV), MD>0 favours the column treatment. In the upper-right triangle (median MNCV), MD>0 favours the column treatment. Bold values indicate 95% CIs that do not cross 0. CT is the reference comparator. GRADE certainty is denoted as follows: *, high; †, moderate; ‡, low; §, very low.

Twenty-seven studies involving 2,625 participants evaluated nine interventions for common peroneal MNCV. Moderate-certainty evidence showed that MOXI+EHM increased common peroneal MNCV versus CT (MD = 6.14 m/s, 95% CI 3.82 to 8.46; **Figure 3**). MOXI+ACU also increased common peroneal MNCV (MD = 5.39 m/s, 95% CI 2.60 to 8.18). A similar effect was observed for MOXI+IHM (MD = 3.95 m/s, 95% CI 1.99 to 5.9), but both comparisons had very-low-certainty evidence.

MOXI+EHM had the highest SUCRA value (92.3%), followed by MOXI+ACU (83.5%). Because certainty was low for several interventions, these rankings should be considered exploratory (**Supplementary Table 19**; **Supplementary Figure 2**).

#### 3.4.2 Median MNCV

Seventeen studies involving 1,981 participants evaluated eight interventions for median MNCV. Moderate-certainty evidence showed that MOXI+EHM increased median MNCV versus CT (MD = 6.35 m/s, 95% CI 2.35 to 10.36; **Figure 3**). MOXI+ACU also increased median MNCV (MD = 6.47 m/s, 95% CI 1.27 to 11.67). A similar effect was observed for MOXI+IHM (MD = 4.09 m/s, 95% CI 2.12 to 6.07), but both comparisons had very-low-certainty evidence.

MOXI+EHM had the highest SUCRA value (86.5%), followed by MOXI+ACU (83%). Because certainty was low for several interventions, these rankings should be considered exploratory (**Supplementary Table 20**; **Supplementary Figure 3**).

#### 3.4.3 Common peroneal SNCV

**Figure 4:**
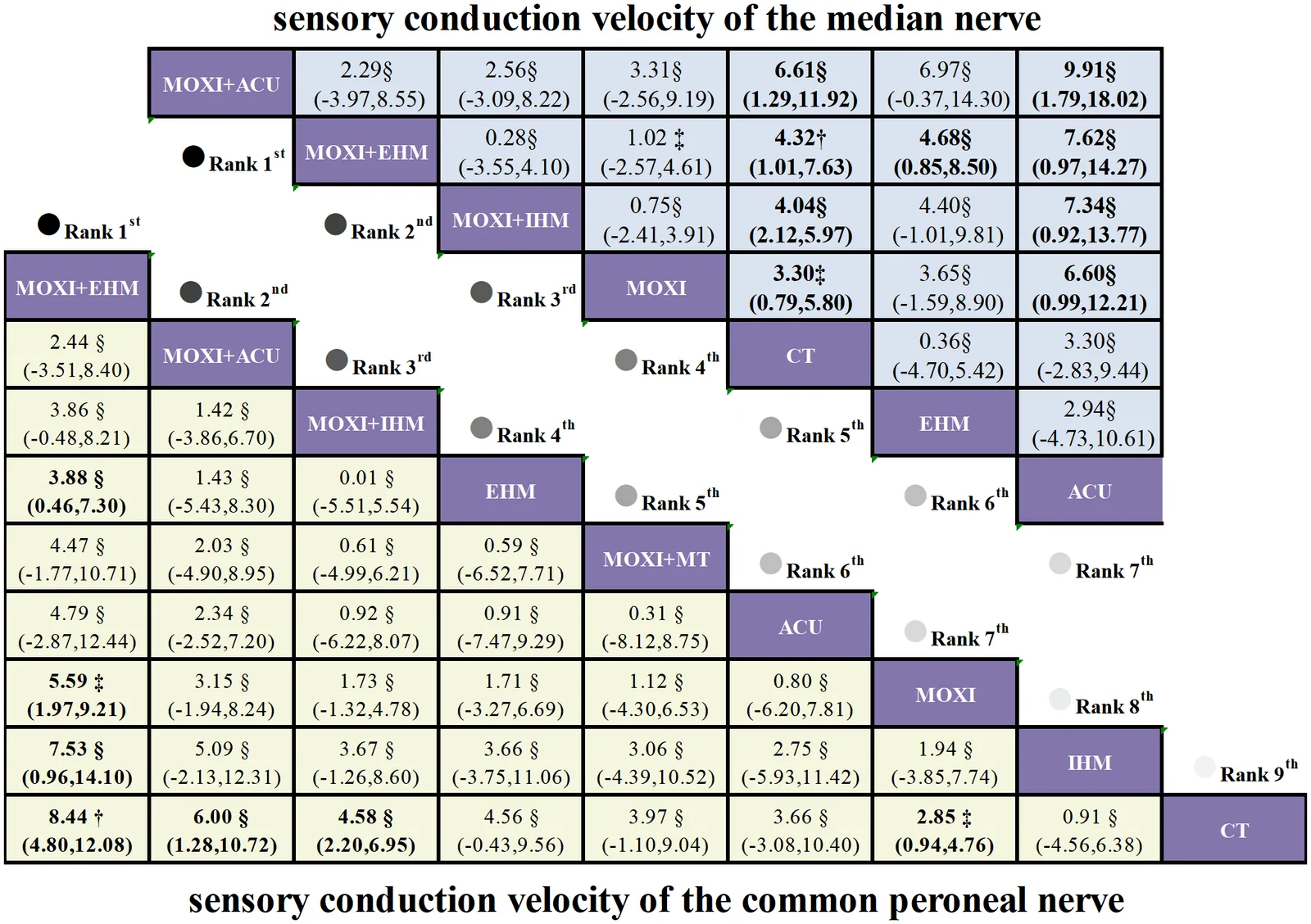
League tables for common peroneal SNCV and median SNCV in the primary analyses. League tables from the network meta-analysis of moxibustion-based combination therapies for DPN, ordered by SUCRA value. Comparisons are read from left to right, with estimates shown at the intersection of the column and row treatments. Continuous outcomes are reported as MDs with 95% CIs. In the lower-left triangle (common peroneal SNCV), MD>0 favours the column treatment. In the upper-right triangle (median SNCV), MD>0 favours the column treatment. Bold values indicate 95% CIs that do not cross 0. CT is the reference comparator. GRADE certainty is denoted as follows: *, high; †, moderate; ‡, low; §, very low.

Eighteen studies involving 1,864 participants evaluated nine interventions for common peroneal SNCV. Moderate-certainty evidence showed that MOXI+EHM increased common peroneal SNCV versus CT (MD = 8.44 m/s, 95% CI 4.80 to 12.08; **Figure 4**). MOXI+ACU also increased common peroneal SNCV (MD = 6.00 m/s, 95% CI 1.28 to 10.72). A similar effect was observed for MOXI+IHM (MD = 4.58 m/s, 95% CI 2.20 to 6.95), but both comparisons had very-low-certainty evidence.

MOXI+EHM had the highest SUCRA value (94.1%), followed by MOXI+ACU (74.2%). Because certainty was low for several interventions, these rankings should be considered exploratory (**Supplementary Table 21**; **Supplementary Figure 4**).

#### 3.4.4 Median SNCV

Nineteen studies involving 2,029 participants evaluated seven interventions for median SNCV. Moderate-certainty evidence showed that MOXI+EHM increased median SNCV versus CT (MD = 4.32 m/s, 95% CI 1.01 to 7.63; **Figure 4**). MOXI+ACU also increased median SNCV (MD = 6.61 m/s, 95% CI 1.29 to 11.92). MOXI+IHM showed a similar effect (MD = 4.04 m/s, 95% CI 2.12 to 5.97).

MOXI+ACU had the highest SUCRA value (89.9%), followed by MOXI+EHM (75%). Because certainty was low for several interventions, these rankings should be considered exploratory (**Supplementary Table 22**; **Supplementary Figure 5**).

#### 3.4.5 TCSS

**Figure 5:**
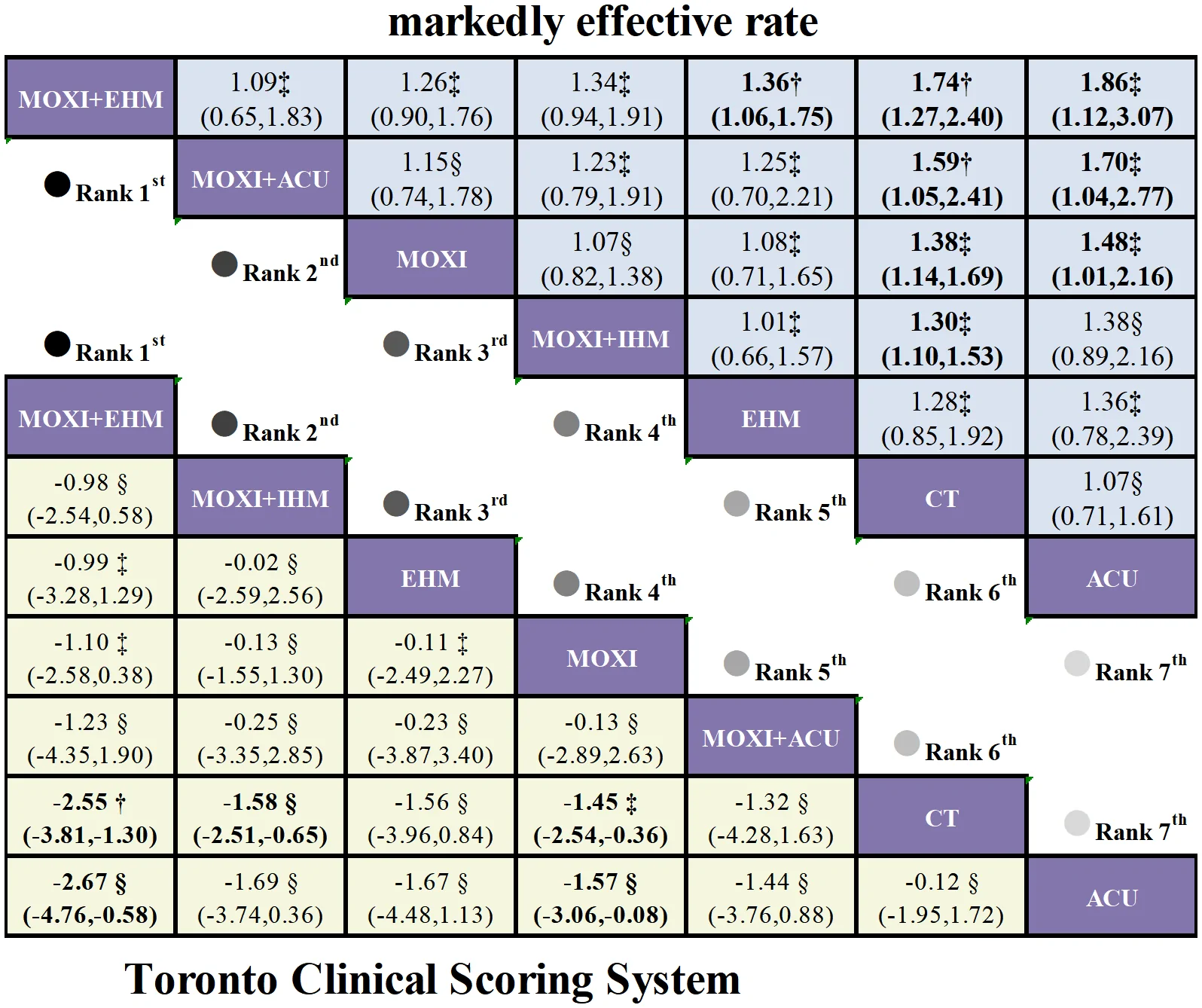
League tables for TCSS and markedly effective rate in the primary analyses. League tables from the network meta-analysis of moxibustion-based combination therapies for DPN, ordered by SUCRA value. TCSS is reported as an MD and markedly effective rate as an RR, each with 95% CIs. In the lower-left triangle, MD<0 favours the column treatment and indicates a greater reduction in TCSS. In the upper-right triangle, RR>1 favours the column treatment. Bold values indicate CIs that do not cross 0 for MDs or 1 for RRs. CT is the reference comparator. GRADE certainty is denoted as follows: *, high; †, moderate; ‡, low; §, very low.

Fourteen studies involving 1,413 participants evaluated seven interventions for TCSS. Moderate-certainty evidence showed that MOXI+EHM reduced TCSS scores versus CT (MD=-2.55 points, 95% CI -3.81 to -1.30; **Figure 5**). MOXI+IHM also reduced TCSS scores (MD=-1.58 points, 95% CI -2.51 to -0.65). MOXI alone showed a similar effect (MD=-1.45 points, 95% CI -2.54 to -0.36).

MOXI+EHM had the highest SUCRA value (89.8%), followed by MOXI+IHM (61.7%). Because certainty was low for several interventions, these rankings should be considered exploratory (**Supplementary Table 23**; **Supplementary Figure 6**).

#### 3.4.6 Markedly effective rate

Twenty-two studies involving 1,950 participants evaluated seven interventions for markedly effective rate. Overall, 978 participants achieved a markedly effective response (approximately 50.2%). Moderate-certainty evidence favoured MOXI+EHM over CT (RR = 1.74, 95% CI 1.27 to 2.40; **Figure 5**). Evidence also favoured MOXI+ACU (RR = 1.59, 95% CI 1.05 to 2.41). MOXI alone increased the markedly effective rate (RR = 1.38, 95% CI 1.14 to 1.69). MOXI+IHM showed a similar effect (RR = 1.30, 95% CI 1.1 to 1.53), but both comparisons had very-low-certainty evidence.

MOXI+EHM had the highest SUCRA value (91.3%), followed by MOXI+ACU (77.2%). Because certainty was low for several interventions, these rankings should be considered exploratory (**Supplementary Table 24**; **Supplementary Figure 7**).

### 3.5 Secondary outcomes

#### 3.5.1 FPG

**Figure 6:**
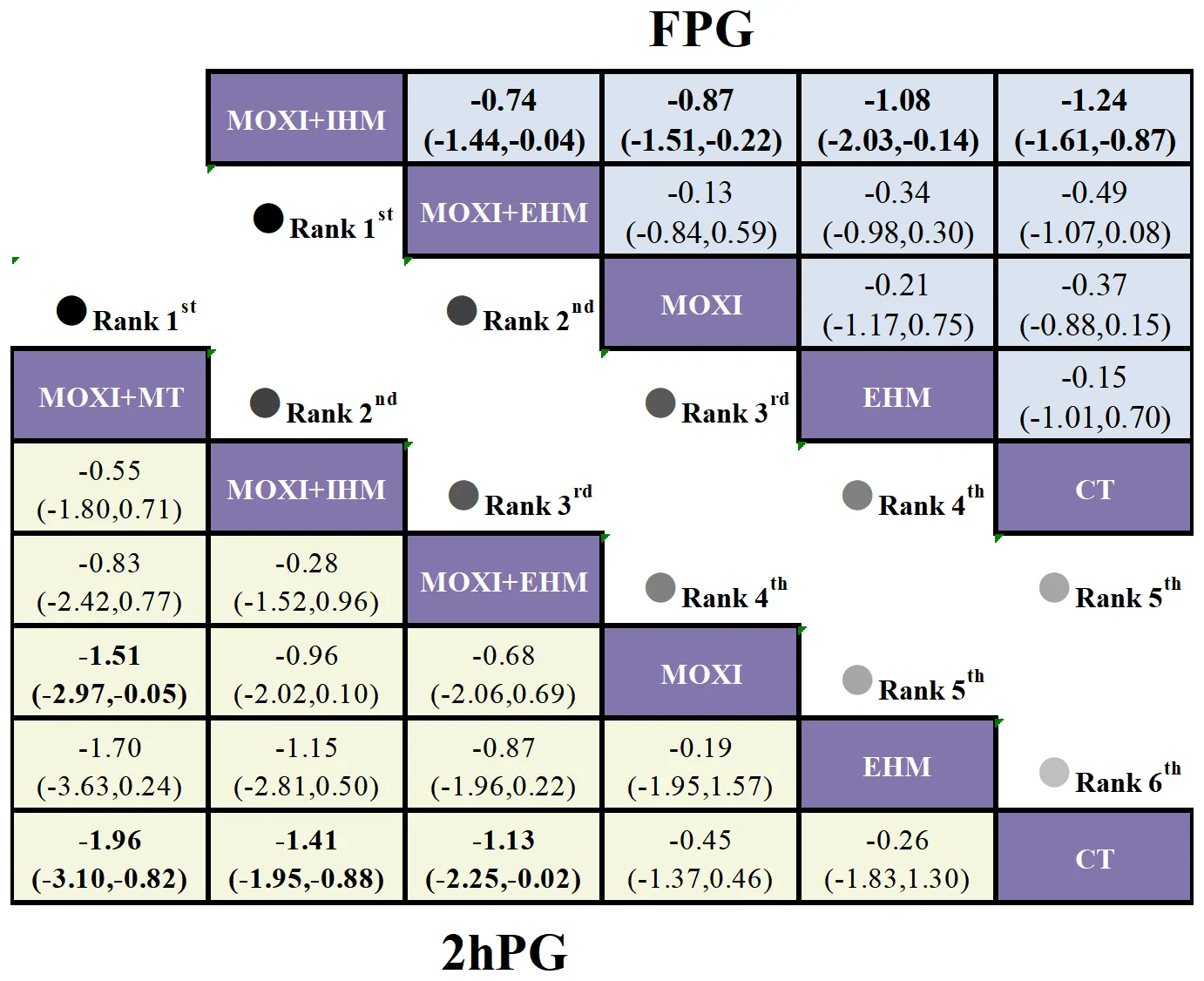
League tables for FPG and 2hPG in the primary analyses. League tables from the network meta-analysis of moxibustion-based combination therapies for DPN, ordered by SUCRA value. FPG and 2hPG are reported as MDs with 95% CIs. In the lower-left triangle (2hPG) and upper-right triangle (FPG), MD<0 favours the column treatment and indicates a greater reduction in glucose concentration. Bold values indicate 95% CIs that do not cross 0. CT is the reference comparator.

Twelve studies involving 1,100 participants evaluated five interventions for FPG. MOXI+IHM reduced FPG versus CT (MD=-1.24 mmol/L, 95% CI -1.61 to -0.87; **Figure 6**).

MOXI+IHM had the highest SUCRA value (99%), followed by MOXI+EHM (61.5%). GRADE was not applied to this outcome, so certainty remains unclear and the ranking is exploratory (**Supplementary Table 25**; **Supplementary Figure 8**).

#### 3.5.2 2hPG

Eleven studies involving 1,042 participants evaluated six interventions for 2hPG. MOXI+IHM reduced 2hPG versus CT (MD=-1.41 mmol/L, 95% CI -1.95 to -0.88; **Figure 6**). MOXI+MT also reduced 2hPG (MD=-1.96 mmol/L, 95% CI -3.10 to -0.82).

MOXI+MT had the highest SUCRA value (91.7%), followed by MOXI+IHM (74.8%). GRADE was not applied to this outcome, so certainty remains unclear and the ranking is exploratory (**Supplementary Table 26**; **Supplementary Figure 9**).

### 3.6 Subgroup analysis

Technique-level subgroup analyses separated WN-, MM-, HM-, TF-, GIM- and WDM-based combinations. Closed loops were present in the TCSS and markedly effective rate networks (**Supplementary Figures 10**, **11**). Global inconsistency tests, node splitting and loop-specific analyses showed no evidence of inconsistency (**Supplementary Tables 27–S33**; **Supplementary Figures 12**, **13**).

**Figure 7:**
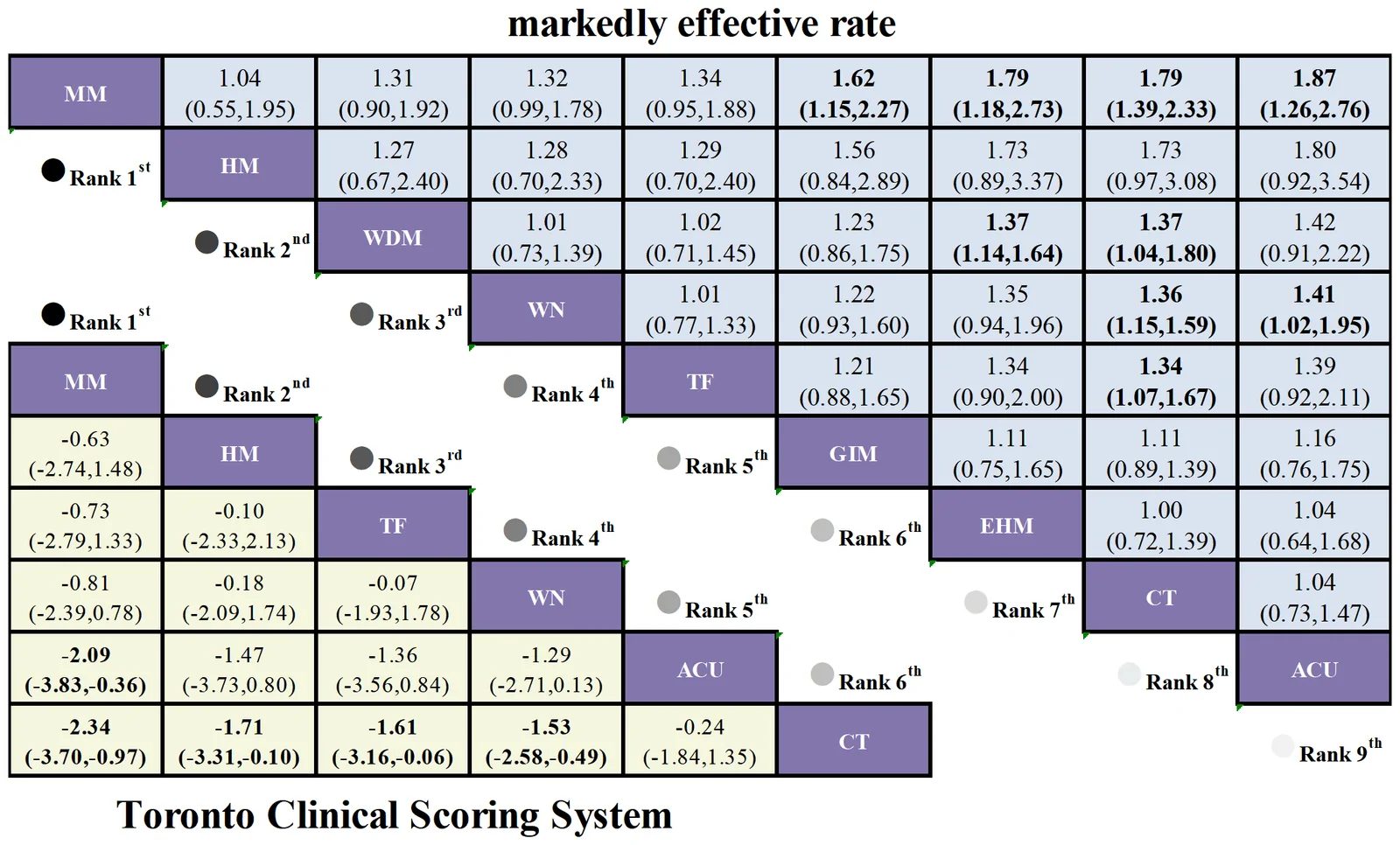
League tables for TCSS and markedly effective rate in the subgroup analyses. League tables for technique-level subgroup analyses, ordered by SUCRA value. TCSS is reported as an MD and markedly effective rate as an RR, each with 95% CIs. In the lower-left triangle, MD<0 favours the column treatment. In the upper-right triangle, RR>1 favours the column treatment. Bold values indicate CIs that do not cross 0 for MDs or 1 for RRs. CT is the reference comparator.

For TCSS, MM-based combinations reduced scores versus CT (MD=-2.34 points, 95% CI -3.70 to -0.97). HM-based combinations also reduced scores (MD=-1.71 points, 95% CI -3.31 to -0.10). For markedly effective rate, evidence favoured MM-based combinations (RR = 1.79, 95% CI 1.39 to 2.33). WDM-based combinations also outperformed CT (RR = 1.37, 95% CI 1.04 to 1.80; **Figure 7**).

**Figure 8:**
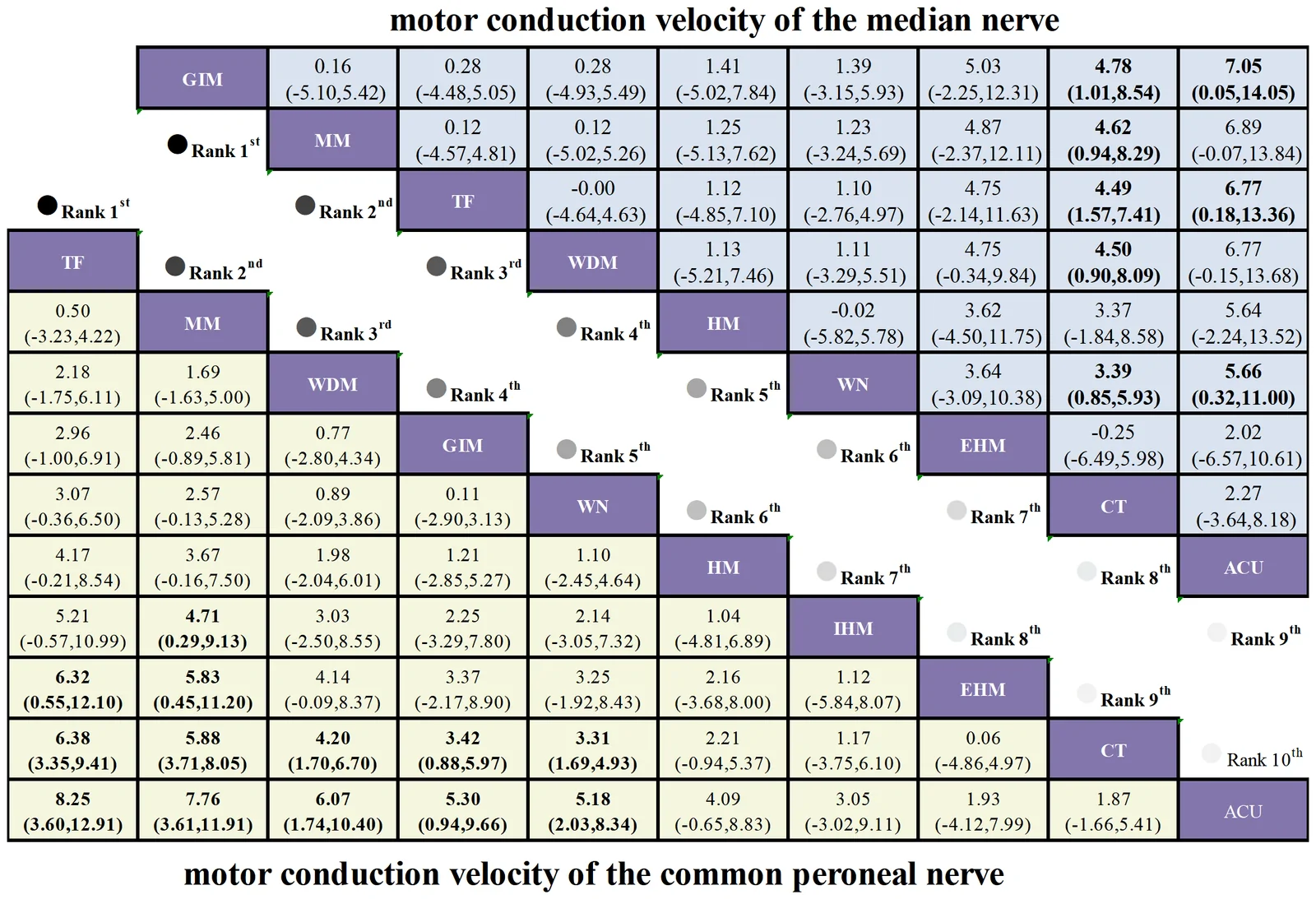
League tables for common peroneal MNCV and median MNCV in the subgroup analyses. League tables for technique-level subgroup analyses, ordered by SUCRA value. Common peroneal MNCV and median MNCV are reported as MDs with 95% CIs. MD>0 favours the column treatment in both triangles. Bold values indicate 95% CIs that do not cross 0. CT is the reference comparator.

**Figure 9:**
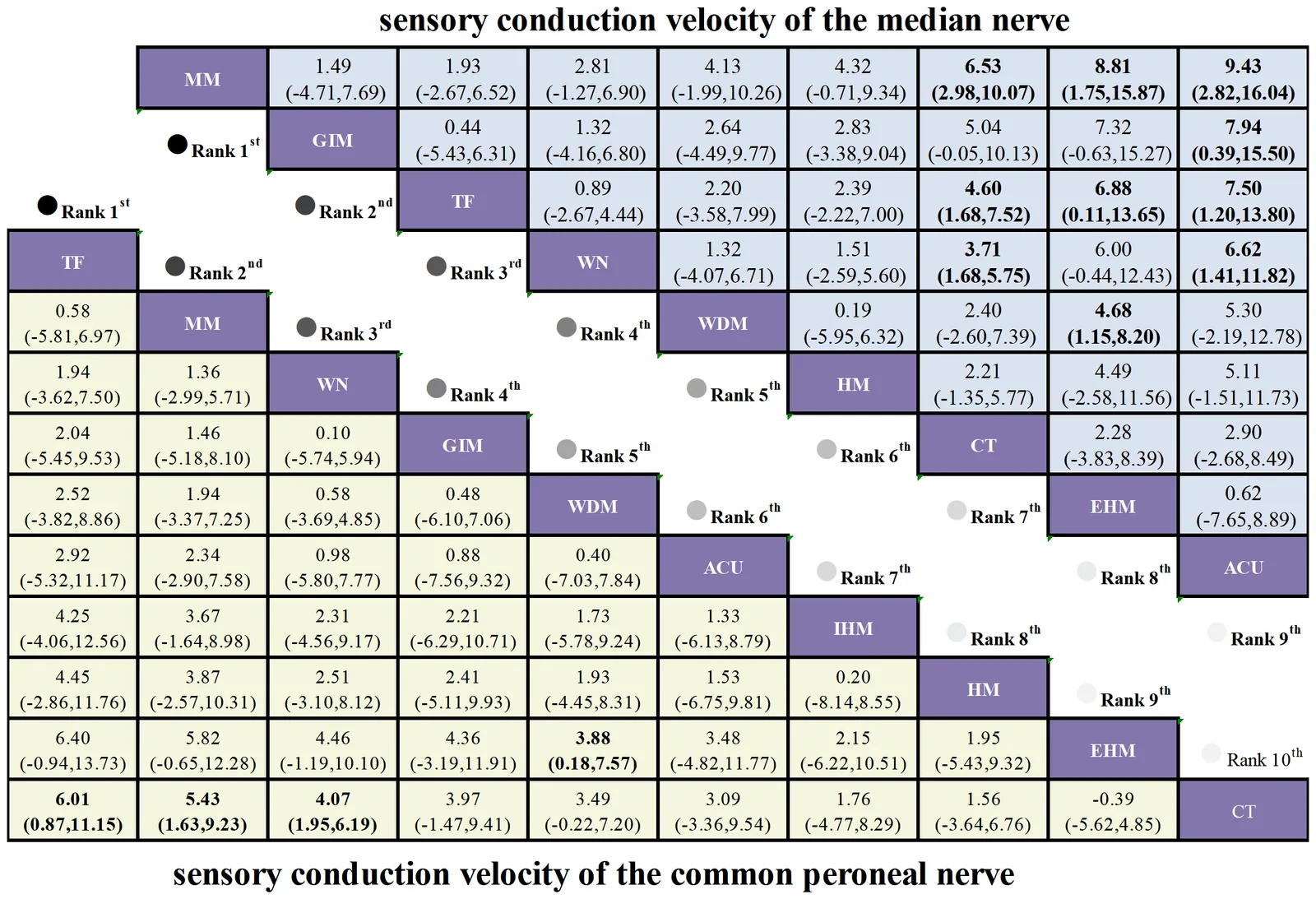
League tables for common peroneal SNCV and median SNCV in the subgroup analyses. League tables for technique-level subgroup analyses, ordered by SUCRA value. Common peroneal SNCV and median SNCV are reported as MDs with 95% CIs. MD>0 favours the column treatment in both triangles. Bold values indicate 95% CIs that do not cross 0. CT is the reference comparator.

**Figure 10:**
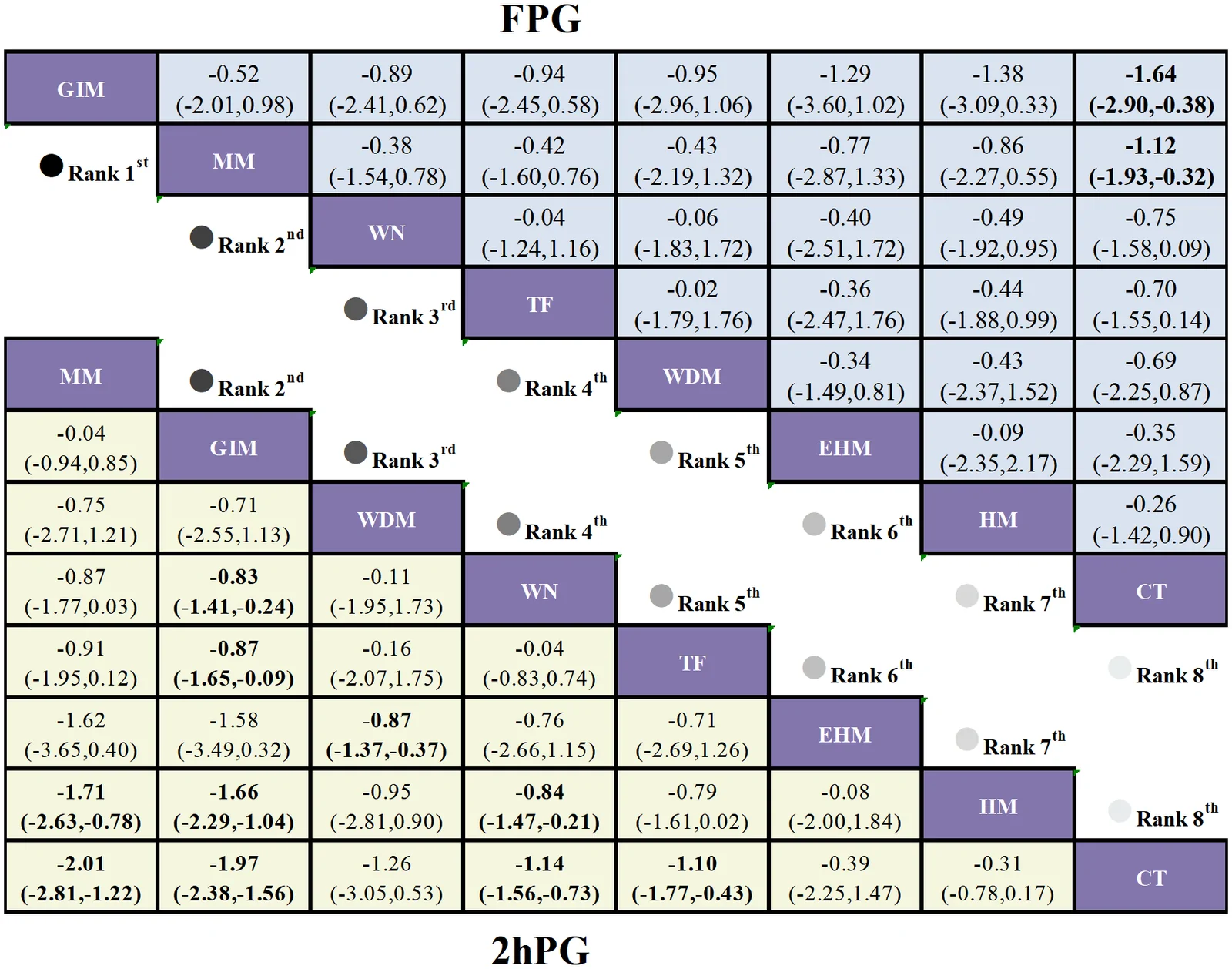
League tables for FPG and 2hPG in the subgroup analyses. League tables for technique-level subgroup analyses, ordered by SUCRA value. FPG and 2hPG are reported as MDs with 95% CIs. MD<0 favours the column treatment in both triangles and indicates a greater reduction in glucose concentration. Bold values indicate 95% CIs that do not cross 0. CT is the reference comparator.

The networks for common peroneal MNCV, median MNCV, common peroneal SNCV, median SNCV, FPG and 2hPG contained no closed loops (**Supplementary Tables 34–S39**; **Supplementary Figures 14–25**). Compared with CT, common peroneal MNCV increased with TF-based combinations (MD = 6.38 m/s, 95% CI 3.35 to 9.41). MM-based combinations showed a similar effect (MD = 5.88 m/s, 95% CI 3.71 to 8.05). Median MNCV increased with GIM-based combinations (MD = 4.78 m/s, 95% CI 1.01 to 8.54). It also increased with MM-based combinations (MD = 4.62 m/s, 95% CI 0.94 to 8.29).Common peroneal SNCV increased with TF-based combinations (MD = 6.01 m/s, 95% CI 0.87 to 11.15). MM-based combinations showed a similar effect (MD = 5.43 m/s, 95% CI 1.63 to 9.23). Median SNCV increased with MM-based combinations (MD = 6.53 m/s, 95% CI 2.98 to 10.09). TF-based combinations also increased median SNCV (MD = 4.60 m/s, 95% CI 1.68 to 7.52). FPG decreased with GIM-based combinations (MD=-1.64 mmol/L, 95% CI -2.90 to -0.38). It also decreased with MM-based combinations (MD=-1.12 mmol/L, 95% CI -1.93 to -0.32). 2hPG decreased with MM-based combinations (MD=-2.01 mmol/L, 95% CI -2.81 to -1.22). GIM-based combinations showed a similar effect (MD=-1.97 mmol/L, 95% CI -2.38 to -1.56; **Figures 8**–**10**).

### 3.7 Meta-regression, sensitivity analyses and publication bias

Key baseline characteristics were broadly balanced across treatment groups. In primary and subgroup analyses, network meta-regression found no statistically significant associations of mean intervention duration, age or diabetes duration with treatment effects. These findings supported the transitivity assumption (**Supplementary Tables 40–S87**).

The main analysis set R = 0.5, while sensitivity analyses set R = 0.25 or R = 0.75 (**Supplementary Tables 88–S132**). We also excluded each study sequentially in leave-one-out analyses. Varying R produced little change in relative-effect direction, magnitude, 95% CIs or statistical significance. Removing individual studies likewise did not materially change comparisons between moxibustion-based combinations and CT. The principal results were therefore insensitive to these assumptions (**Supplementary Tables 133–S148**).

Funnel plots were used to assess small-study effects and publication bias. Scatter distributions were broadly symmetrical, without marked systematic asymmetry or extreme outliers, although this does not exclude publication bias (**Supplementary Figures 26–41**).

### 3.8 Certainty of evidence

**Figure 11:**
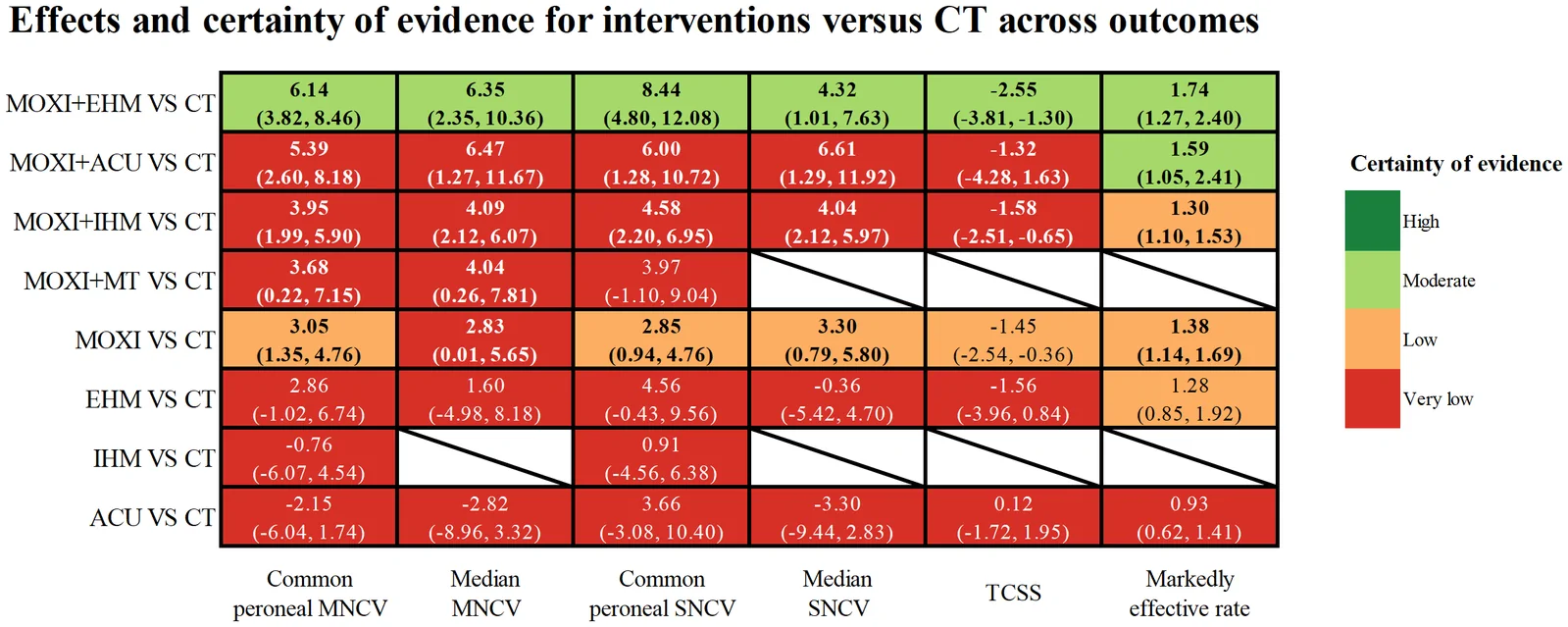
Summary of network estimates and certainty of evidence for interventions versus CT across primary outcomes. Values are network estimates with 95% CIs. MDs are presented for nerve conduction outcomes and TCSS, and RRs for the markedly effective rate. MD > 0 favours the intervention for nerve conduction outcomes, MD < 0 favours the intervention for TCSS, and RR > 1 favours the intervention for the markedly effective rate. Colours represent CINeMA certainty ratings: dark green, high; light green, moderate; orange, low; and red, very low. White cells with diagonal lines indicate unavailable network estimates.

Certainty was assessed with GRADE and CINeMA (**Supplementary Tables 149–S154**). **Figure 11** summarizes the principal network estimates for each intervention versus CT, together with the corresponding certainty-of-evidence ratings, providing an overview of treatment effects and evidence certainty across the six primary outcomes. Across comparisons in the six primary-outcome networks, 0 were rated high certainty, 8 moderate, 26 low and 129 very low. Higher-certainty comparisons were generally supported by several direct head-to-head trials and had CIs that crossed neither the null nor the MID. Low- and very-low-certainty comparisons commonly arose from sparse networks, reliance on indirect evidence, wide CIs or potential small-study effects.

## 4 Discussion

### 4.1 Principal findings

This NMA integrated direct and indirect evidence on moxibustion combined with different adjunctive therapies for DPN. It compared relative effects and treatment rankings to inform the selection of integrated traditional Chinese medicine interventions.

The review included 40 RCTs, 3,718 participants and nine intervention categories. Moderate- to very-low-certainty evidence suggested that MOXI+EHM, MOXI+ACU and MOXI+IHM may improve motor and sensory nerve conduction relative to CT. MOXI+EHM, MOXI+IHM and MOXI alone may improve TCSS symptoms and signs. Moderate-certainty evidence suggested higher markedly effective rates with MOXI+EHM and MOXI+ACU. For secondary outcomes, MOXI+IHM ranked highly for FPG, whereas MOXI+MT, MOXI+IHM and MOXI+EHM ranked highly for 2hPG. These glycaemic rankings remain uncertain because GRADE was not applied.

Technique-level analyses indicated possible differences among moxibustion methods. MM- and TF-based combinations ranked highly for several nerve-conduction and clinical outcomes. GIM-, HM-, WN- and WDM-based combinations showed potential benefits for selected outcomes. GIM- and MM-based combinations ranked favourably for FPG and 2hPG. These subgroup rankings are exploratory because several networks lacked closed loops and much of the evidence was sparse.

### 4.2 Interpretation of the findings

MOXI+EHM was among the most consistently ranked interventions in the primary analyses. Moxibustion-induced heat may modulate neuroinflammation and redox homeostasis, potentially reducing peripheral nerve damage and improving conduction (24). EHM included herbal foot baths, fumigation or washing, and acupoint application. In addition to local drug exposure, these treatments may provide thermal stimulation. Herbal medicines may reduce free-radical production, improve endothelial function and limb perfusion, and thereby relieve pain and numbness (25).

MOXI and EHM could plausibly have complementary effects on inflammation, oxidative stress and neural sensitisation. However, the included trials did not directly test this synergy. The relatively consistent clinical ranking of MOXI+EHM should therefore not be interpreted as mechanistic evidence.

MOXI+IHM also showed potential benefits. IHM included Huangqi Guizhi Wuwu decoction, Huazhuo Tongluo formula, Yiqi Ziyin formula, Yiqi Yangyin decoction and Buyang Huanwu decoction. Internal herbal medicines may modulate inflammation, reduce inflammatory mediators (26) and improve glucose-lipid metabolism, thereby limiting chronic hyperglycaemic nerve injury (27). MOXI+IHM could therefore exert complementary thermal, circulatory and metabolic effects. Direct evidence for synergy is nevertheless lacking, and the observed rankings do not establish a shared mechanism.

In the technique-level analyses, MM may provide sustained thermal stimulation that improves haemorheology, microcirculation and the ischaemic-hypoxic microenvironment of neural tissue (28). Evidence supporting this mechanism remains limited. TF combines moxa heat with the thermal effects of herbal materials. It may improve local perfusion and reduce hyperglycaemia-related neural ischaemia. Acupoint stimulation may also influence pain pathways and neural sensitisation (29). These mechanisms remain hypotheses and require direct experimental confirmation.

### 4.3 Implications for practice and research

The findings support further evaluation of moxibustion-based combinations as adjuncts within comprehensive DPN care. MOXI+EHM showed potential benefits across several primary and secondary outcomes, warranting assessment of its feasibility and acceptability in primary and community care. Different combinations may serve different clinical goals, which supports an individualised approach.

MOXI+EHM or MOXI+ACU may be considered for further evaluation when impaired nerve conduction is a primary treatment target. However, this inference is based on network comparisons with evidence ranging from moderate to very low certainty. Decisions should account for previous treatment response, treatment burden and acceptable frequency, and patients should be informed that higher-quality trials are needed.

### 4.4 Comparison with previous research

The intervention scope differed from that of the pairwise meta-analysis by Tan and colleagues (3). That review compared moxibustion plus Western medicine with Western medicine alone and excluded combinations involving acupuncture, massage or herbal medicine. Pairwise comparisons against a common control could not compare all moxibustion-based combinations simultaneously.

By contrast, our NMA placed different combinations in one evidence network and estimated their relative effects using direct and indirect evidence. Technique-level subgroup analyses treated WN, MM, HM, TF, GIM and WDM as separate nodes. The present review assessed four nerve-conduction outcomes, TCSS and markedly effective rate as primary outcomes, together with FPG and 2hPG as secondary outcomes. It also applied GRADE to distinguish treatment rankings from certainty of evidence.

### 4.5 Strengths and limitations

The review used RoB 2 to assess risk of bias and GRADE to evaluate certainty of evidence. Technique-level subgroup analyses explored differences among moxibustion methods. Sensitivity analyses and network meta-regression assessed the robustness of the principal findings.

Several limitations should be acknowledged. Blinding participants and practitioners is generally impractical for moxibustion, and all included studies (100%) were judged to have some concerns overall. This limitation may reduce the reliability of effect estimates, particularly for comparisons supported by few studies. Although funnel plots showed no marked asymmetry, most comparisons included too few studies to exclude publication bias.Only four studies reported adverse events, so a safety NMA was not performed. The included studies differed in intervention duration and treatment regimens. Although network meta-regression based on mean intervention duration did not identify a significant modifying effect on treatment outcomes, the potential influence of variation in treatment duration and intervention protocols cannot be entirely excluded. Future studies should adopt more standardized treatment durations and intervention protocols to improve comparability across studies and strengthen the reliability of the evidence. In addition, the included studies generally lacked medium- and long-term follow-up. Given the recurrent nature of DPN, the present findings therefore mainly reflect short-term treatment effects, and evidence on medium- and long-term efficacy remains limited. Finally, every study was conducted in China. The generalisability of these findings to other populations and healthcare settings is therefore uncertain.

### 4.6 Conclusion

In this NMA of 40 RCTs, moderate-certainty evidence suggested that MOXI+EHM may improve common peroneal MNCV, median MNCV, common peroneal SNCV, median SNCV, TCSS and markedly effective rate in DPN. Because all included studies were conducted in China and many comparisons had low or very low certainty, these findings should guide research rather than establish a preferred treatment. Large, rigorous RCTs should use standardised interventions and assess long-term outcomes after treatment.

## Data Availability

The original contributions presented in the study are included in the article/**Supplementary Material**. Further inquiries can be directed to the corresponding author.
