# Supplementary material for: Moxibustion combinations for diabetic peripheral neuropathy: a network meta-analysis of 40 randomised trials
Source: Front Endocrinol (Lausanne). 2026 Sep 3;17:1941999. doi: 10.3389/fendo.2026.1941999 (PMC13581844; doi:10.3389/fendo.2026.1941999)
Supplement: Supplementary file 1 [file DataSheet1.docx]

Figure1: Result of the risk of bias assessment using the RoB 2 tool

Figure2: Rank plot based on the common peroneal MNCV in the primary analysis of patients with DPN

Figure3: Rank plot based on the median MNCV in the primary analysis of patients with DPN

Figure4: Rank plot based on the common peroneal SNCV in the primary analysis of patients with DPN

Figure5: Rank plot based on the median SNCV in the primary analysis of patients with DPN

Figure6: Rank plot based on the TCSS in the primary analysis of patients with DPN

Figure7: Rank plot based on the markedly effective rate in the primary analysis of patients with DPN

Figure8: Rank plot based on the FPG in the primary analysis of patients with DPN

Figure9: Rank plot based on the 2hPG in the primary analysis of patients with DPN

Figure10: Network plot based on the TCSS in the subgroup analysis of patients with DPN

Figure11: Network plot based on the markedly effective rate in the subgroup analysis of patients with DPN

Figure12: Rank plot based on the TCSS in the subgroup analysis of patients with DPN.

Figure13: Rank plot based on the markedly effective rate in the subgroup analysis of patients with DPN

Figure14:

Network plot based on the common peroneal MNCV in the subgroup analysis of patients with DPN

Figure15:Network plot based on the median MNCV in the subgroup analysis of patients with DPN

Figure16:

Network plot based on the common peroneal SNCV in the subgroup analysis of patients with DPN

Figure17:Network plot based on the median SNCV in the subgroup analysis of patients with DPN

Figure18:Network plot based on the FPG in the subgroup analysis of patients with DPN

Figure19:Network plot based on the 2hPG in the subgroup analysis of patients with DPN

Figure20: Rank plot based on the common peroneal MNCV in the subgroup analysis of patients with DPN

Figure21: Rank plot based on the median MNCV in the subgroup analysis of patients with DPN

Figure22: Rank plot based on the common peroneal SNCV in the subgroup analysis of patients with DPN

Figure23: Rank plot based on the median SNCV in the subgroup analysis of patients with DPN

Figure24: Rank plot based on the FPG in the subgroup analysis of patients with DPN

Figure25: Rank plot based on the 2hPG in the subgroup analysis of patients with DPN

Figure26: Funnel plot based on the common peroneal MNCV in the primary analysis of patients with DPN

. Figure27: Funnel plot based on the median MNCV in the primary analysis of patients with DPN

Figure28: Funnel plot based on the common peroneal SNCV in the primary analysis of patients with DPN

. Figure29: Funnel plot based on the median SNCV in the primary analysis of patients with DPN

Figure30: Funnel plot based on the TCSS in the primary analysis of patients with DPN

. Figure31: Funnel plot based on the markedly effective rate in the primary analysis of patients with DPN

Figure32: Funnel plot based on the FPG in the primary analysis of patients with DPN

. Figure33: Funnel plot based on the 2hPG in the primary analysis of patients with DPN

Figure34: Funnel plot based on the common peroneal MNCV in the subgroup analysis of patients with DPN

. Figure35: Funnel plot based on the median MNCV in the subgroup analysis of patients with DPN

Figure36: Funnel plot based on the common peroneal SNCV in the subgroup analysis of patients with DPN

. Figure37: Funnel plot based on the median SNCV in the subgroup analysis of patients with DPN

Figure38: Funnel plot based on the TCSS in the subgroup analysis of patients with DPN

. Figure39: Funnel plot based on the markedly effective rate in the subgroup analysis of patients with DPN

Figure40: Funnel plot based on the FPG in the subgroup analysis of patients with DPN

. Figure41: Funnel plot based on the 2hPG in the subgroup analysis of patients with DPN
