# Supplementary material for: Moxibustion combinations for diabetic peripheral neuropathy: a network meta-analysis of 40 randomised trials
Source: Front Endocrinol (Lausanne). 2026 Sep 3;17:1941999. doi: 10.3389/fendo.2026.1941999 (PMC13581844; doi:10.3389/fendo.2026.1941999)
Supplement: Supplementary file 2 [file Table1.docx]

Table S1	PRISMA NMA Checklist of Items to Include When Reporting a Systematic Review Involving a Network Meta-analysis

| Section/Topic | Item # | Checklist Item | Reported on Page # |
| --- | --- | --- | --- |
| TITLE |  |  |  |
| Title | 1 | Identify the report as a systematic review *incorporating*  anetwork meta-analysis (or related form of meta-analysis). | 1 |
|  |  |  |  |
| ABSTRACT |  |  |  |
| Structured summary | 2 | Provide a structured summary including, as applicable:  **Background:** main objectives  **Methods:** data sources; study eligibility criteria, participants, and interventions; study appraisal; and *synthesis methods, such as network meta-analysis.*  **Results:** number of studies and participants identified; summary estimates with corresponding confidence/credible intervals; treatment rankings may also be discussed. Authors may choose to summarize pairwise comparisons against a chosen treatment included in their analyses for brevity.  **Discussion/Conclusions:** limitations; conclusions and implications of findings.  **Other:** systematic review registration number with registry name. | 1 |
|  |  |  |  |
| INTRODUCTION |  |  |  |
| Rationale | 3 | Describe the rationale for the review in the context of what is already known*, including mention of why a network meta-analysis has been conducted.* | 1 |
| Objectives | 4 | Provide an explicit statement of questions being addressed, with reference to participants, interventions, comparisons, outcomes, and study design (PICOS). | 2 |
|  |  |  |  |
| METHODS |  |  |  |
| Protocol and registration | 5 | Indicate whether a review protocol exists and if and where it can be accessed (e.g., Web address); and, if available, provide registration information, including registration number. | 2 |
| Eligibility criteria | 6 | Specify study characteristics (e.g., PICOS, length of follow-up) and report characteristics (e.g., years considered, language, publication status) used as criteria for eligibility, giving rationale. *Clearly describe eligible treatments included in the treatment network, and note whether any have been clustered or merged into the same node (with justification).* | 2 |
| Information sources | 7 | Describe all information sources (e.g., databases with dates of coverage, contact with study authors to identify additional studies) in the search and date last searched. | 2 |
| Search | 8 | Present full electronic search strategy for at least one database, including any limits used, such that it could be repeated. | 2，Supplementary  TableS2 |
| Study selection | 9 | State the process for selecting studies (i.e., screening, eligibility, included in systematic review, and, if applicable, included in the meta-analysis). | 3 |
| Data collection process | 10 | Describe method of data extraction from reports (e.g., piloted forms, independently, in duplicate) and any processes for obtaining and confirming data from investigators. | 3 |
| Data items | 11 | List and define all variables for which data were sought (e.g., PICOS, funding sources) and any assumptions and simplifications made. | 3-4 |
| Geometry of the network | S1 | Describe methods used to explore the geometry of the treatment network under study and potential biases related to it. This should include how the evidence base has been graphically summarized for presentation, and what characteristics were compiled and used to describe the evidence base to readers. | 4 |
| Risk of bias within individual studies | 12 | Describe methods used for assessing risk of bias of individual studies (including specification of whether this was done at the study or outcome level), and how this information is to be used in any data synthesis. | 4 |
| Summary measures | 13 | State the principal summary measures (e.g., risk ratio, difference in means). Also describe the use of additional summary measures assessed, such as treatment rankings and surface under the cumulative ranking curve (SUCRA) values, as well as modified approaches used to present summary findings from meta-analyses. | 4 |
| Planned methods of analysis | 14 | Describe the methods of handling data and combining results of studies for each network meta-analysis. This should include, but not be limited to:   - Handling of multi-arm trials; - Selection of variance structure; - Selection of prior distributions in Bayesian analyses; and - Assessment of model fit. | 4 |
| Assessment of Inconsistency | S2 | Describe the statistical methods used to evaluate the agreement of direct and indirect evidence in the treatment network(s) studied. Describe efforts taken to address its presence when found. | 5 |
| Risk of bias across studies | 15 | Specify any assessment of risk of bias that may affect the cumulative evidence (e.g., publication bias, selective reporting within studies). | 5 |
| Additional analyses | 16 | Describe methods of additional analyses if done, indicating which were pre-specified. This may include, but not be limited to, the following:   - Sensitivity or subgroup analyses; - Meta-regression analyses; - Alternative formulations of the treatment network; and - Use of alternative prior distributions for Bayesian analyses (if applicable). | 5 |
| RESULTS† |  |  |  |
| Study selection | 17 | Give numbers of studies screened, assessed for eligibility, and included in the review, with reasons for exclusions at each stage, ideally with a flow diagram. | 5，Figure1 |
| Presentation of network structure | S3 | Provide a network graph of the included studies to enable visualization of the geometry of the treatment network. | 5-8，Figure2 |
| Summary of network geometry | S4 | Provide a brief overview of characteristics of the treatment network. This may include commentary on the abundance of trials and randomized patients for the different interventions and pairwise comparisons in the network, gaps of evidence in the treatment network, and potential biases reflected by the network structure. | 10-13 |
| Study characteristics | 18 | For each study, present characteristics for which data were extracted (e.g., study size, PICOS, follow-up period) and provide the citations. | 5-7, Supplementary  Tables S4-5 |
| Risk of bias within studies | 19 | Present data on risk of bias of each study and, if available, any outcome level assessment. | 7-8 |
| Results of individual studies | 20 | For all outcomes considered (benefits or harms), present, for each study: 1) simple summary data for each intervention group, and 2) effect estimates and confidence intervals. *Modified approaches may be needed to deal with information from larger networks.* | 7-8 |
| Synthesis of results | 21 | Present results of each meta-analysis done, including confidence/credible intervals. In larger networks, authors may focus on comparisons versus a particular comparator (e.g. placebo or standard care), with full findings presented in an appendix. League tables and forest plots may be considered to summarize pairwise comparisons. If additional summary measures were explored (such as treatment rankings), these should also be presented. | 8 |
| Exploration for inconsistency | S5 | Describe results from investigations of inconsistency. This may include such information as measures of model fit to compare consistency and inconsistency models, *P* values from statistical tests, or summary of inconsistency estimates from different parts of the treatment network. | 17 |
| Risk of bias across studies | 22 | Present results of any assessment of risk of bias across studies for the evidence base being studied. | 8 Figure 3-5 |
| Results of additional analyses | 23 | Give results of additional analyses, if done (e.g., sensitivity or subgroup analyses, meta-regression analyses*, alternative network geometries studied, alternative choice of prior distributions for Bayesian analyses,* and so forth). | 8 |
|  |  |  |  |
| DISCUSSION |  |  |  |
| Summary of evidence | 24 | Summarize the main findings, including the strength of evidence for each main outcome; consider their relevance to key groups (e.g., healthcare providers, users, and policy-makers). | 8 |
| Limitations | 25 | Discuss limitations at study and outcome level (e.g., risk of bias), and at review level (e.g., incomplete retrieval of identified research, reporting bias). *Comment on the validity of the assumptions, such as transitivity and consistency. Comment on any concerns regarding network geometry (e.g., avoidance of certain comparisons).* | 9 |
| Conclusions | 26 | Provide a general interpretation of the results in the context of other evidence, and implications for future research. | 9-10 |
|  |  |  |  |
| FUNDING |  |  |  |
| Funding | 27 | Describe sources of funding for the systematic review and other support (e.g., supply of data); role of funders for the systematic review. This should also include information regarding whether funding has been received from manufacturers of treatments in the network and/or whether some of the authors are content experts with professional conflicts of interest that could affect use of treatments in the network. | NA |

PICOS = population, intervention, comparators, outcomes, study design.

* Text in italics indicate S wording specific to reporting of network meta-analyses that has been added to guidance from the PRISMA statement.

† Authors may wish to plan for use of appendices to present all relevant information in full detail for items in this section.

检索式

| **Table S2.Literature Search Strategy** | |
| --- | --- |
| Pubmed | 1 "Diabetic Neuropathies"[Mesh] Most Recent "Diabetic Neuropathies"[MeSH Terms] 30,536  2 ((("Diabetic Neuropathies"[Mesh]) OR (Diabetic Neuropathies[Title/Abstract])) OR (Diabetic Neuropathy[Title/Abstract]) OR (Neuropathies, Diabetic[Title/Abstract]) OR (Neuropathy, Diabetic[Title/Abstract]) OR (Diabetic Amyotrophy[Title/Abstract]) OR (Amyotrophies, Diabetic[Title/Abstract]) OR (Amyotrophy, Diabetic[Title/Abstract]) OR (Diabetic Amyotrophies[Title/Abstract]) OR (Diabetic Autonomic Neuropathy[Title/Abstract]) OR (Autonomic Neuropathies, Diabetic[Title/Abstract]) OR (Autonomic Neuropathy, Diabetic[Title/Abstract]) OR (Diabetic Autonomic Neuropathies[Title/Abstract]) OR (Neuropathies, Diabetic Autonomic[Title/Abstract]) OR (Neuropathy, Diabetic Autonomic[Title/Abstract]) OR (Diabetic Neuralgia[Title/Abstract]) OR (Diabetic Neuralgias[Title/Abstract]) OR (Neuralgias, Diabetic[Title/Abstract]) OR (Neuralgia, Diabetic[Title/Abstract]) OR (Diabetic Neuropathy, Painful[Title/Abstract]) OR (Diabetic Neuropathies, Painful[Title/Abstract]) OR (Neuropathies, Painful Diabetic[Title/Abstract]) OR (Neuropathy, Painful Diabetic[Title/Abstract]) OR (Painful Diabetic Neuropathies[Title/Abstract]) OR (Painful Diabetic Neuropathy[Title/Abstract]) OR (Diabetic Polyneuropathy[Title/Abstract]) OR (Diabetic Polyneuropathies[Title/Abstract]) OR (Polyneuropathies, Diabetic[Title/Abstract]) OR (Polyneuropathy, Diabetic[Title/Abstract]) OR (Asymmetric Diabetic Proximal Motor Neuropathy[Title/Abstract]) OR (Diabetic Asymmetric Polyneuropathy[Title/Abstract]) OR (Asymmetric Polyneuropathies, Diabetic[Title/Abstract]) OR (Asymmetric Polyneuropathy, Diabetic[Title/Abstract]) OR (Diabetic Asymmetric Polyneuropathies[Title/Abstract]) OR (Polyneuropathies, Diabetic Asymmetric[Title/Abstract]) OR (Polyneuropathy, Diabetic Asymmetric[Title/Abstract]) OR (Diabetic Mononeuropathy[Title/Abstract]) OR (Diabetic Mononeuropathies[Title/Abstract]) OR (Mononeuropathies, Diabetic[Title/Abstract]) OR (Diabetic Mononeuropathy Simplex[Title/Abstract]) OR (Diabetic Mononeuropathy Simplices[Title/Abstract]) OR (Mononeuropathy Simplex, Diabetic[Title/Abstract]) OR (Mononeuropathy Simplices, Diabetic[Title/Abstract]) OR (Simplex, Diabetic Mononeuropathy[Title/Abstract]) OR (Simplices, Diabetic Mononeuropathy[Title/Abstract]) OR (Mononeuropathy, Diabetic[Title/Abstract]) OR (Symmetric Diabetic Proximal Motor Neuropathy[Title/Abstract])) "Diabetic Neuropathies"[MeSH Terms] OR "Diabetic Neuropathies"[Title/Abstract] OR "diabetic neuropathy"[Title/Abstract] OR "neuropathies diabetic"[Title/Abstract] OR "neuropathy diabetic"[Title/Abstract] OR "diabetic amyotrophy"[Title/Abstract] OR (("muscular atrophy"[MeSH Terms] OR ("muscular"[All Fields] AND "atrophy"[All Fields]) OR "muscular atrophy"[All Fields] OR "Amyotrophies"[All Fields] OR "Amyotrophy"[All Fields]) AND "Diabetic"[Title/Abstract]) OR "amyotrophy diabetic"[Title/Abstract] OR (("diabete"[All Fields] OR "diabetes mellitus"[MeSH Terms] OR ("diabetes"[All Fields] AND "mellitus"[All Fields]) OR "diabetes mellitus"[All Fields] OR "diabetes"[All Fields] OR "diabetes insipidus"[MeSH Terms] OR ("diabetes"[All Fields] AND "insipidus"[All Fields]) OR "diabetes insipidus"[All Fields] OR "Diabetic"[All Fields] OR "diabetics"[All Fields] OR "diabets"[All Fields]) AND "Amyotrophies"[Title/Abstract]) OR "diabetic autonomic neuropathy"[Title/Abstract] OR "autonomic neuropathies diabetic"[Title/Abstract] OR "autonomic neuropathy diabetic"[Title/Abstract] OR "diabetic autonomic neuropathies"[Title/Abstract] OR (("Neuropathies"[All Fields] OR "Neuropathy"[All Fields]) AND "diabetic autonomic"[Title/Abstract]) OR "neuropathy diabetic autonomic"[Title/Abstract] OR "diabetic neuralgia"[Title/Abstract] OR (("diabete"[All Fields] OR "diabetes mellitus"[MeSH Terms] OR ("diabetes"[All Fields] AND "mellitus"[All Fields]) OR "diabetes mellitus"[All Fields] OR "diabetes"[All Fields] OR "diabetes insipidus"[MeSH Terms] OR ("diabetes"[All Fields] AND "insipidus"[All Fields]) OR "diabetes insipidus"[All Fields] OR "Diabetic"[All Fields] OR "diabetics"[All Fields] OR "diabets"[All Fields]) AND "Neuralgias"[Title/Abstract]) OR (("Neuralgia"[MeSH Terms] OR "Neuralgia"[All Fields] OR "Neuralgias"[All Fields]) AND "Diabetic"[Title/Abstract]) OR "neuralgia diabetic"[Title/Abstract] OR "diabetic neuropathy painful"[Title/Abstract] OR "diabetic neuropathies painful"[Title/Abstract] OR (("Neuropathies"[All Fields] OR "Neuropathy"[All Fields]) AND "painful diabetic"[Title/Abstract]) OR "neuropathy painful diabetic"[Title/Abstract] OR "painful diabetic neuropathies"[Title/Abstract] OR "painful diabetic neuropathy"[Title/Abstract] OR "diabetic polyneuropathy"[Title/Abstract] OR "diabetic polyneuropathies"[Title/Abstract] OR "polyneuropathies diabetic"[Title/Abstract] OR "polyneuropathy diabetic"[Title/Abstract] OR ((("Asymmetric"[All Fields] OR "asymmetrical"[All Fields] OR "asymmetrically"[All Fields] OR "asymmetrics"[All Fields] OR "asymmetrization"[All Fields]) AND ("diabete"[All Fields] OR "diabetes mellitus"[MeSH Terms] OR ("diabetes"[All Fields] AND "mellitus"[All Fields]) OR "diabetes mellitus"[All Fields] OR "diabetes"[All Fields] OR "diabetes insipidus"[MeSH Terms] OR ("diabetes"[All Fields] AND "insipidus"[All Fields]) OR "diabetes insipidus"[All Fields] OR "Diabetic"[All Fields] OR "diabetics"[All Fields] OR "diabets"[All Fields])) AND "proximal motor neuropathy"[Title/Abstract]) OR (("diabete"[All Fields] OR "diabetes mellitus"[MeSH Terms] OR ("diabetes"[All Fields] AND "mellitus"[All Fields]) OR "diabetes mellitus"[All Fields] OR "diabetes"[All Fields] OR "diabetes insipidus"[MeSH Terms] OR ("diabetes"[All Fields] AND "insipidus"[All Fields]) OR "diabetes insipidus"[All Fields] OR "Diabetic"[All Fields] OR "diabetics"[All Fields] OR "diabets"[All Fields]) AND "asymmetric polyneuropathy"[Title/Abstract]) OR (("Asymmetric"[All Fields] OR "asymmetrical"[All Fields] OR "asymmetrically"[All Fields] OR "asymmetrics"[All Fields] OR "asymmetrization"[All Fields]) AND "polyneuropathies diabetic"[Title/Abstract]) OR (("Asymmetric"[All Fields] OR "asymmetrical"[All Fields] OR "asymmetrically"[All Fields] OR "asymmetrics"[All Fields] OR "asymmetrization"[All Fields]) AND "polyneuropathy diabetic"[Title/Abstract]) OR (("diabete"[All Fields] OR "diabetes mellitus"[MeSH Terms] OR ("diabetes"[All Fields] AND "mellitus"[All Fields]) OR "diabetes mellitus"[All Fields] OR "diabetes"[All Fields] OR "diabetes insipidus"[MeSH Terms] OR ("diabetes"[All Fields] AND "insipidus"[All Fields]) OR "diabetes insipidus"[All Fields] OR "Diabetic"[All Fields] OR "diabetics"[All Fields] OR "diabets"[All Fields]) AND "asymmetric polyneuropathies"[Title/Abstract]) OR (("Polyneuropathies"[MeSH Terms] OR "Polyneuropathies"[All Fields] OR "Polyneuropathy"[All Fields]) AND "diabetic asymmetric"[Title/Abstract]) OR (("Polyneuropathies"[MeSH Terms] OR "Polyneuropathies"[All Fields] OR "Polyneuropathy"[All Fields]) AND "diabetic asymmetric"[Title/Abstract]) OR "diabetic mononeuropathy"[Title/Abstract] OR "diabetic mononeuropathies"[Title/Abstract] OR (("Mononeuropathies"[MeSH Terms] OR "Mononeuropathies"[All Fields] OR "Mononeuropathy"[All Fields]) AND "Diabetic"[Title/Abstract]) OR (("diabete"[All Fields] OR "diabetes mellitus"[MeSH Terms] OR ("diabetes"[All Fields] AND "mellitus"[All Fields]) OR "diabetes mellitus"[All Fields] OR "diabetes"[All Fields] OR "diabetes insipidus"[MeSH Terms] OR ("diabetes"[All Fields] AND "insipidus"[All Fields]) OR "diabetes insipidus"[All Fields] OR "Diabetic"[All Fields] OR "diabetics"[All Fields] OR "diabets"[All Fields]) AND "mononeuropathy simplex"[Title/Abstract]) OR (("Diabetic Neuropathies"[MeSH Terms] OR ("Diabetic"[All Fields] AND "Neuropathies"[All Fields]) OR "Diabetic Neuropathies"[All Fields] OR ("Diabetic"[All Fields] AND "Mononeuropathy"[All Fields]) OR "diabetic mononeuropathy"[All Fields]) AND "Simplices"[Title/Abstract]) OR (("Mononeuropathies"[MeSH Terms] OR "Mononeuropathies"[All Fields] OR "Mononeuropathy"[All Fields]) AND "simplex diabetic"[Title/Abstract]) OR ((("Mononeuropathies"[MeSH Terms] OR "Mononeuropathies"[All Fields] OR "Mononeuropathy"[All Fields]) AND "Simplices"[All Fields]) AND "Diabetic"[Title/Abstract]) OR (("Simplex"[All Fields] OR "simplexes"[All Fields]) AND "diabetic mononeuropathy"[Title/Abstract]) OR ("Simplices"[All Fields] AND "diabetic mononeuropathy"[Title/Abstract]) OR "mononeuropathy diabetic"[Title/Abstract] OR ((("symmetric"[All Fields] OR "symmetrical"[All Fields] OR "symmetrically"[All Fields] OR "symmetrization"[All Fields] OR "symmetrize"[All Fields] OR "symmetrized"[All Fields] OR "symmetrizes"[All Fields] OR "symmetrizing"[All Fields]) AND ("diabete"[All Fields] OR "diabetes mellitus"[MeSH Terms] OR ("diabetes"[All Fields] AND "mellitus"[All Fields]) OR "diabetes mellitus"[All Fields] OR "diabetes"[All Fields] OR "diabetes insipidus"[MeSH Terms] OR ("diabetes"[All Fields] AND "insipidus"[All Fields]) OR "diabetes insipidus"[All Fields] OR "Diabetic"[All Fields] OR "diabetics"[All Fields] OR "diabets"[All Fields])) AND "proximal motor neuropathy"[Title/Abstract]) 35,828  3 ("Moxibustion"[Mesh] OR moxibustion[Title/Abstract] OR moxabustion[Title/Abstract] OR moxa[Title/Abstract] OR "moxa therapy"[Title/Abstract] OR "moxibustion therapy"[Title/Abstract] OR "warm needling"[Title/Abstract] OR "warm needle"[Title/Abstract] OR "warm-needling"[Title/Abstract] OR "warming needle moxibustion"[Title/Abstract] OR "warm acupuncture"[Title/Abstract] OR "warm acupuncture and moxibustion"[Title/Abstract] OR "warming needle"[Title/Abstract] OR "indirect moxibustion"[Title/Abstract] OR "partitioned moxibustion"[Title/Abstract] OR "interposed moxibustion"[Title/Abstract] OR "separated moxibustion"[Title/Abstract] OR "ginger moxibustion"[Title/Abstract] OR "ginger-separated moxibustion"[Title/Abstract] OR "ginger partition moxibustion"[Title/Abstract] OR "ginger-partitioned moxibustion"[Title/Abstract] OR "herb-partitioned moxibustion"[Title/Abstract] OR "herb-separated moxibustion"[Title/Abstract] OR "medicinal moxibustion"[Title/Abstract] OR "drug-separated moxibustion"[Title/Abstract] OR "governor vessel moxibustion"[Title/Abstract] OR "du moxibustion"[Title/Abstract] OR "du meridian moxibustion"[Title/Abstract] OR "heat-sensitive moxibustion"[Title/Abstract] OR "heat sensitization moxibustion"[Title/Abstract] OR "thermal sensitization moxibustion"[Title/Abstract] OR "thermosensitive moxibustion"[Title/Abstract] OR "thunder-fire moxibustion"[Title/Abstract] OR "thunder fire moxibustion"[Title/Abstract] OR "mild moxibustion"[Title/Abstract] OR "gentle moxibustion"[Title/Abstract] OR "fire dragon pot moxibustion"[Title/Abstract] OR "fire dragon moxibustion"[Title/Abstract] OR "fire dragon pot"[Title/Abstract] OR "fire dragon cupping"[Title/Abstract]) "Moxibustion"[MeSH Terms] OR "Moxibustion"[Title/Abstract] OR "moxabustion"[Title/Abstract] OR "moxa"[Title/Abstract] OR "moxibustion therapy"[Title/Abstract] OR "warm-needling"[Title/Abstract] OR "warm needle"[Title/Abstract] OR "warm-needling"[Title/Abstract] OR "warming needle moxibustion"[Title/Abstract] OR "warm acupuncture"[Title/Abstract] OR "warming needle"[Title/Abstract] OR "indirect moxibustion"[Title/Abstract] OR "partitioned moxibustion"[Title/Abstract] OR "separated moxibustion"[Title/Abstract] OR "ginger moxibustion"[Title/Abstract] OR "ginger-separated moxibustion"[Title/Abstract] OR "ginger partition moxibustion"[Title/Abstract] OR "ginger-partitioned moxibustion"[Title/Abstract] OR "herb-partitioned moxibustion"[Title/Abstract] OR "herb-separated moxibustion"[Title/Abstract] OR "medicinal moxibustion"[Title/Abstract] OR "drug-separated moxibustion"[Title/Abstract] OR "governor vessel moxibustion"[Title/Abstract] OR "du moxibustion"[Title/Abstract] OR "du meridian moxibustion"[Title/Abstract] OR "heat-sensitive moxibustion"[Title/Abstract] OR "thermosensitive moxibustion"[Title/Abstract] OR "thunder-fire moxibustion"[Title/Abstract] OR "thunder-fire moxibustion"[Title/Abstract] OR "mild moxibustion"[Title/Abstract] OR "fire dragon pot moxibustion"[Title/Abstract] OR "fire dragon moxibustion"[Title/Abstract] OR "fire dragon pot"[Title/Abstract] OR "fire dragon cupping"[Title/Abstract] 5,049  4 #2 and #3 ("Diabetic Neuropathies"[MeSH Terms] OR "Diabetic Neuropathies"[Title/Abstract] OR "diabetic neuropathy"[Title/Abstract] OR "neuropathies diabetic"[Title/Abstract] OR "neuropathy diabetic"[Title/Abstract] OR "diabetic amyotrophy"[Title/Abstract] OR (("muscular atrophy"[MeSH Terms] OR ("muscular"[All Fields] AND "atrophy"[All Fields]) OR "muscular atrophy"[All Fields] OR "Amyotrophies"[All Fields] OR "Amyotrophy"[All Fields]) AND "Diabetic"[Title/Abstract]) OR "amyotrophy diabetic"[Title/Abstract] OR (("diabete"[All Fields] OR "diabetes mellitus"[MeSH Terms] OR ("diabetes"[All Fields] AND "mellitus"[All Fields]) OR "diabetes mellitus"[All Fields] OR "diabetes"[All Fields] OR "diabetes insipidus"[MeSH Terms] OR ("diabetes"[All Fields] AND "insipidus"[All Fields]) OR "diabetes insipidus"[All Fields] OR "Diabetic"[All Fields] OR "diabetics"[All Fields] OR "diabets"[All Fields]) AND "Amyotrophies"[Title/Abstract]) OR "diabetic autonomic neuropathy"[Title/Abstract] OR "autonomic neuropathies diabetic"[Title/Abstract] OR "autonomic neuropathy diabetic"[Title/Abstract] OR "diabetic autonomic neuropathies"[Title/Abstract] OR (("Neuropathies"[All Fields] OR "Neuropathy"[All Fields]) AND "diabetic autonomic"[Title/Abstract]) OR "neuropathy diabetic autonomic"[Title/Abstract] OR "diabetic neuralgia"[Title/Abstract] OR (("diabete"[All Fields] OR "diabetes mellitus"[MeSH Terms] OR ("diabetes"[All Fields] AND "mellitus"[All Fields]) OR "diabetes mellitus"[All Fields] OR "diabetes"[All Fields] OR "diabetes insipidus"[MeSH Terms] OR ("diabetes"[All Fields] AND "insipidus"[All Fields]) OR "diabetes insipidus"[All Fields] OR "Diabetic"[All Fields] OR "diabetics"[All Fields] OR "diabets"[All Fields]) AND "Neuralgias"[Title/Abstract]) OR (("Neuralgia"[MeSH Terms] OR "Neuralgia"[All Fields] OR "Neuralgias"[All Fields]) AND "Diabetic"[Title/Abstract]) OR "neuralgia diabetic"[Title/Abstract] OR "diabetic neuropathy painful"[Title/Abstract] OR "diabetic neuropathies painful"[Title/Abstract] OR (("Neuropathies"[All Fields] OR "Neuropathy"[All Fields]) AND "painful diabetic"[Title/Abstract]) OR "neuropathy painful diabetic"[Title/Abstract] OR "painful diabetic neuropathies"[Title/Abstract] OR "painful diabetic neuropathy"[Title/Abstract] OR "diabetic polyneuropathy"[Title/Abstract] OR "diabetic polyneuropathies"[Title/Abstract] OR "polyneuropathies diabetic"[Title/Abstract] OR "polyneuropathy diabetic"[Title/Abstract] OR ((("Asymmetric"[All Fields] OR "asymmetrical"[All Fields] OR "asymmetrically"[All Fields] OR "asymmetrics"[All Fields] OR "asymmetrization"[All Fields]) AND ("diabete"[All Fields] OR "diabetes mellitus"[MeSH Terms] OR ("diabetes"[All Fields] AND "mellitus"[All Fields]) OR "diabetes mellitus"[All Fields] OR "diabetes"[All Fields] OR "diabetes insipidus"[MeSH Terms] OR ("diabetes"[All Fields] AND "insipidus"[All Fields]) OR "diabetes insipidus"[All Fields] OR "Diabetic"[All Fields] OR "diabetics"[All Fields] OR "diabets"[All Fields])) AND "proximal motor neuropathy"[Title/Abstract]) OR (("diabete"[All Fields] OR "diabetes mellitus"[MeSH Terms] OR ("diabetes"[All Fields] AND "mellitus"[All Fields]) OR "diabetes mellitus"[All Fields] OR "diabetes"[All Fields] OR "diabetes insipidus"[MeSH Terms] OR ("diabetes"[All Fields] AND "insipidus"[All Fields]) OR "diabetes insipidus"[All Fields] OR "Diabetic"[All Fields] OR "diabetics"[All Fields] OR "diabets"[All Fields]) AND "asymmetric polyneuropathy"[Title/Abstract]) OR (("Asymmetric"[All Fields] OR "asymmetrical"[All Fields] OR "asymmetrically"[All Fields] OR "asymmetrics"[All Fields] OR "asymmetrization"[All Fields]) AND "polyneuropathies diabetic"[Title/Abstract]) OR (("Asymmetric"[All Fields] OR "asymmetrical"[All Fields] OR "asymmetrically"[All Fields] OR "asymmetrics"[All Fields] OR "asymmetrization"[All Fields]) AND "polyneuropathy diabetic"[Title/Abstract]) OR (("diabete"[All Fields] OR "diabetes mellitus"[MeSH Terms] OR ("diabetes"[All Fields] AND "mellitus"[All Fields]) OR "diabetes mellitus"[All Fields] OR "diabetes"[All Fields] OR "diabetes insipidus"[MeSH Terms] OR ("diabetes"[All Fields] AND "insipidus"[All Fields]) OR "diabetes insipidus"[All Fields] OR "Diabetic"[All Fields] OR "diabetics"[All Fields] OR "diabets"[All Fields]) AND "asymmetric polyneuropathies"[Title/Abstract]) OR (("Polyneuropathies"[MeSH Terms] OR "Polyneuropathies"[All Fields] OR "Polyneuropathy"[All Fields]) AND "diabetic asymmetric"[Title/Abstract]) OR (("Polyneuropathies"[MeSH Terms] OR "Polyneuropathies"[All Fields] OR "Polyneuropathy"[All Fields]) AND "diabetic asymmetric"[Title/Abstract]) OR "diabetic mononeuropathy"[Title/Abstract] OR "diabetic mononeuropathies"[Title/Abstract] OR (("Mononeuropathies"[MeSH Terms] OR "Mononeuropathies"[All Fields] OR "Mononeuropathy"[All Fields]) AND "Diabetic"[Title/Abstract]) OR (("diabete"[All Fields] OR "diabetes mellitus"[MeSH Terms] OR ("diabetes"[All Fields] AND "mellitus"[All Fields]) OR "diabetes mellitus"[All Fields] OR "diabetes"[All Fields] OR "diabetes insipidus"[MeSH Terms] OR ("diabetes"[All Fields] AND "insipidus"[All Fields]) OR "diabetes insipidus"[All Fields] OR "Diabetic"[All Fields] OR "diabetics"[All Fields] OR "diabets"[All Fields]) AND "mononeuropathy simplex"[Title/Abstract]) OR (("Diabetic Neuropathies"[MeSH Terms] OR ("Diabetic"[All Fields] AND "Neuropathies"[All Fields]) OR "Diabetic Neuropathies"[All Fields] OR ("Diabetic"[All Fields] AND "Mononeuropathy"[All Fields]) OR "diabetic mononeuropathy"[All Fields]) AND "Simplices"[Title/Abstract]) OR (("Mononeuropathies"[MeSH Terms] OR "Mononeuropathies"[All Fields] OR "Mononeuropathy"[All Fields]) AND "simplex diabetic"[Title/Abstract]) OR ((("Mononeuropathies"[MeSH Terms] OR "Mononeuropathies"[All Fields] OR "Mononeuropathy"[All Fields]) AND "Simplices"[All Fields]) AND "Diabetic"[Title/Abstract]) OR (("Simplex"[All Fields] OR "simplexes"[All Fields]) AND "diabetic mononeuropathy"[Title/Abstract]) OR ("Simplices"[All Fields] AND "diabetic mononeuropathy"[Title/Abstract]) OR "mononeuropathy diabetic"[Title/Abstract] OR ((("symmetric"[All Fields] OR "symmetrical"[All Fields] OR "symmetrically"[All Fields] OR "symmetrization"[All Fields] OR "symmetrize"[All Fields] OR "symmetrized"[All Fields] OR "symmetrizes"[All Fields] OR "symmetrizing"[All Fields]) AND ("diabete"[All Fields] OR "diabetes mellitus"[MeSH Terms] OR ("diabetes"[All Fields] AND "mellitus"[All Fields]) OR "diabetes mellitus"[All Fields] OR "diabetes"[All Fields] OR "diabetes insipidus"[MeSH Terms] OR ("diabetes"[All Fields] AND "insipidus"[All Fields]) OR "diabetes insipidus"[All Fields] OR "Diabetic"[All Fields] OR "diabetics"[All Fields] OR "diabets"[All Fields])) AND "proximal motor neuropathy"[Title/Abstract])) AND ("Moxibustion"[MeSH Terms] OR "Moxibustion"[Title/Abstract] OR "moxabustion"[Title/Abstract] OR "moxa"[Title/Abstract] OR "moxibustion therapy"[Title/Abstract] OR "warm-needling"[Title/Abstract] OR "warm needle"[Title/Abstract] OR "warm-needling"[Title/Abstract] OR "warming needle moxibustion"[Title/Abstract] OR "warm acupuncture"[Title/Abstract] OR "warming needle"[Title/Abstract] OR "indirect moxibustion"[Title/Abstract] OR "partitioned moxibustion"[Title/Abstract] OR "separated moxibustion"[Title/Abstract] OR "ginger moxibustion"[Title/Abstract] OR "ginger-separated moxibustion"[Title/Abstract] OR "ginger partition moxibustion"[Title/Abstract] OR "ginger-partitioned moxibustion"[Title/Abstract] OR "herb-partitioned moxibustion"[Title/Abstract] OR "herb-separated moxibustion"[Title/Abstract] OR "medicinal moxibustion"[Title/Abstract] OR "drug-separated moxibustion"[Title/Abstract] OR "governor vessel moxibustion"[Title/Abstract] OR "du moxibustion"[Title/Abstract] OR "du meridian moxibustion"[Title/Abstract] OR "heat-sensitive moxibustion"[Title/Abstract] OR "thermosensitive moxibustion"[Title/Abstract] OR "thunder-fire moxibustion"[Title/Abstract] OR "thunder-fire moxibustion"[Title/Abstract] OR "mild moxibustion"[Title/Abstract] OR "fire dragon pot moxibustion"[Title/Abstract] OR "fire dragon moxibustion"[Title/Abstract] OR "fire dragon pot"[Title/Abstract] OR "fire dragon cupping"[Title/Abstract]) 21  5 #2 and #3 Randomized Controlled Trial (("Diabetic Neuropathies"[MeSH Terms] OR "Diabetic Neuropathies"[Title/Abstract] OR "diabetic neuropathy"[Title/Abstract] OR "neuropathies diabetic"[Title/Abstract] OR "neuropathy diabetic"[Title/Abstract] OR "diabetic amyotrophy"[Title/Abstract] OR (("muscular atrophy"[MeSH Terms] OR ("muscular"[All Fields] AND "atrophy"[All Fields]) OR "muscular atrophy"[All Fields] OR "Amyotrophies"[All Fields] OR "Amyotrophy"[All Fields]) AND "Diabetic"[Title/Abstract]) OR "amyotrophy diabetic"[Title/Abstract] OR (("diabete"[All Fields] OR "diabetes mellitus"[MeSH Terms] OR ("diabetes"[All Fields] AND "mellitus"[All Fields]) OR "diabetes mellitus"[All Fields] OR "diabetes"[All Fields] OR "diabetes insipidus"[MeSH Terms] OR ("diabetes"[All Fields] AND "insipidus"[All Fields]) OR "diabetes insipidus"[All Fields] OR "Diabetic"[All Fields] OR "diabetics"[All Fields] OR "diabets"[All Fields]) AND "Amyotrophies"[Title/Abstract]) OR "diabetic autonomic neuropathy"[Title/Abstract] OR "autonomic neuropathies diabetic"[Title/Abstract] OR "autonomic neuropathy diabetic"[Title/Abstract] OR "diabetic autonomic neuropathies"[Title/Abstract] OR (("Neuropathies"[All Fields] OR "Neuropathy"[All Fields]) AND "diabetic autonomic"[Title/Abstract]) OR "neuropathy diabetic autonomic"[Title/Abstract] OR "diabetic neuralgia"[Title/Abstract] OR (("diabete"[All Fields] OR "diabetes mellitus"[MeSH Terms] OR ("diabetes"[All Fields] AND "mellitus"[All Fields]) OR "diabetes mellitus"[All Fields] OR "diabetes"[All Fields] OR "diabetes insipidus"[MeSH Terms] OR ("diabetes"[All Fields] AND "insipidus"[All Fields]) OR "diabetes insipidus"[All Fields] OR "Diabetic"[All Fields] OR "diabetics"[All Fields] OR "diabets"[All Fields]) AND "Neuralgias"[Title/Abstract]) OR (("Neuralgia"[MeSH Terms] OR "Neuralgia"[All Fields] OR "Neuralgias"[All Fields]) AND "Diabetic"[Title/Abstract]) OR "neuralgia diabetic"[Title/Abstract] OR "diabetic neuropathy painful"[Title/Abstract] OR "diabetic neuropathies painful"[Title/Abstract] OR (("Neuropathies"[All Fields] OR "Neuropathy"[All Fields]) AND "painful diabetic"[Title/Abstract]) OR "neuropathy painful diabetic"[Title/Abstract] OR "painful diabetic neuropathies"[Title/Abstract] OR "painful diabetic neuropathy"[Title/Abstract] OR "diabetic polyneuropathy"[Title/Abstract] OR "diabetic polyneuropathies"[Title/Abstract] OR "polyneuropathies diabetic"[Title/Abstract] OR "polyneuropathy diabetic"[Title/Abstract] OR ((("Asymmetric"[All Fields] OR "asymmetrical"[All Fields] OR "asymmetrically"[All Fields] OR "asymmetrics"[All Fields] OR "asymmetrization"[All Fields]) AND ("diabete"[All Fields] OR "diabetes mellitus"[MeSH Terms] OR ("diabetes"[All Fields] AND "mellitus"[All Fields]) OR "diabetes mellitus"[All Fields] OR "diabetes"[All Fields] OR "diabetes insipidus"[MeSH Terms] OR ("diabetes"[All Fields] AND "insipidus"[All Fields]) OR "diabetes insipidus"[All Fields] OR "Diabetic"[All Fields] OR "diabetics"[All Fields] OR "diabets"[All Fields])) AND "proximal motor neuropathy"[Title/Abstract]) OR (("diabete"[All Fields] OR "diabetes mellitus"[MeSH Terms] OR ("diabetes"[All Fields] AND "mellitus"[All Fields]) OR "diabetes mellitus"[All Fields] OR "diabetes"[All Fields] OR "diabetes insipidus"[MeSH Terms] OR ("diabetes"[All Fields] AND "insipidus"[All Fields]) OR "diabetes insipidus"[All Fields] OR "Diabetic"[All Fields] OR "diabetics"[All Fields] OR "diabets"[All Fields]) AND "asymmetric polyneuropathy"[Title/Abstract]) OR (("Asymmetric"[All Fields] OR "asymmetrical"[All Fields] OR "asymmetrically"[All Fields] OR "asymmetrics"[All Fields] OR "asymmetrization"[All Fields]) AND "polyneuropathies diabetic"[Title/Abstract]) OR (("Asymmetric"[All Fields] OR "asymmetrical"[All Fields] OR "asymmetrically"[All Fields] OR "asymmetrics"[All Fields] OR "asymmetrization"[All Fields]) AND "polyneuropathy diabetic"[Title/Abstract]) OR (("diabete"[All Fields] OR "diabetes mellitus"[MeSH Terms] OR ("diabetes"[All Fields] AND "mellitus"[All Fields]) OR "diabetes mellitus"[All Fields] OR "diabetes"[All Fields] OR "diabetes insipidus"[MeSH Terms] OR ("diabetes"[All Fields] AND "insipidus"[All Fields]) OR "diabetes insipidus"[All Fields] OR "Diabetic"[All Fields] OR "diabetics"[All Fields] OR "diabets"[All Fields]) AND "asymmetric polyneuropathies"[Title/Abstract]) OR (("Polyneuropathies"[MeSH Terms] OR "Polyneuropathies"[All Fields] OR "Polyneuropathy"[All Fields]) AND "diabetic asymmetric"[Title/Abstract]) OR (("Polyneuropathies"[MeSH Terms] OR "Polyneuropathies"[All Fields] OR "Polyneuropathy"[All Fields]) AND "diabetic asymmetric"[Title/Abstract]) OR "diabetic mononeuropathy"[Title/Abstract] OR "diabetic mononeuropathies"[Title/Abstract] OR (("Mononeuropathies"[MeSH Terms] OR "Mononeuropathies"[All Fields] OR "Mononeuropathy"[All Fields]) AND "Diabetic"[Title/Abstract]) OR (("diabete"[All Fields] OR "diabetes mellitus"[MeSH Terms] OR ("diabetes"[All Fields] AND "mellitus"[All Fields]) OR "diabetes mellitus"[All Fields] OR "diabetes"[All Fields] OR "diabetes insipidus"[MeSH Terms] OR ("diabetes"[All Fields] AND "insipidus"[All Fields]) OR "diabetes insipidus"[All Fields] OR "Diabetic"[All Fields] OR "diabetics"[All Fields] OR "diabets"[All Fields]) AND "mononeuropathy simplex"[Title/Abstract]) OR (("Diabetic Neuropathies"[MeSH Terms] OR ("Diabetic"[All Fields] AND "Neuropathies"[All Fields]) OR "Diabetic Neuropathies"[All Fields] OR ("Diabetic"[All Fields] AND "Mononeuropathy"[All Fields]) OR "diabetic mononeuropathy"[All Fields]) AND "Simplices"[Title/Abstract]) OR (("Mononeuropathies"[MeSH Terms] OR "Mononeuropathies"[All Fields] OR "Mononeuropathy"[All Fields]) AND "simplex diabetic"[Title/Abstract]) OR ((("Mononeuropathies"[MeSH Terms] OR "Mononeuropathies"[All Fields] OR "Mononeuropathy"[All Fields]) AND "Simplices"[All Fields]) AND "Diabetic"[Title/Abstract]) OR (("Simplex"[All Fields] OR "simplexes"[All Fields]) AND "diabetic mononeuropathy"[Title/Abstract]) OR ("Simplices"[All Fields] AND "diabetic mononeuropathy"[Title/Abstract]) OR "mononeuropathy diabetic"[Title/Abstract] OR ((("symmetric"[All Fields] OR "symmetrical"[All Fields] OR "symmetrically"[All Fields] OR "symmetrization"[All Fields] OR "symmetrize"[All Fields] OR "symmetrized"[All Fields] OR "symmetrizes"[All Fields] OR "symmetrizing"[All Fields]) AND ("diabete"[All Fields] OR "diabetes mellitus"[MeSH Terms] OR ("diabetes"[All Fields] AND "mellitus"[All Fields]) OR "diabetes mellitus"[All Fields] OR "diabetes"[All Fields] OR "diabetes insipidus"[MeSH Terms] OR ("diabetes"[All Fields] AND "insipidus"[All Fields]) OR "diabetes insipidus"[All Fields] OR "Diabetic"[All Fields] OR "diabetics"[All Fields] OR "diabets"[All Fields])) AND "proximal motor neuropathy"[Title/Abstract])) AND ("Moxibustion"[MeSH Terms] OR "Moxibustion"[Title/Abstract] OR "moxabustion"[Title/Abstract] OR "moxa"[Title/Abstract] OR "moxibustion therapy"[Title/Abstract] OR "warm-needling"[Title/Abstract] OR "warm needle"[Title/Abstract] OR "warm-needling"[Title/Abstract] OR "warming needle moxibustion"[Title/Abstract] OR "warm acupuncture"[Title/Abstract] OR "warming needle"[Title/Abstract] OR "indirect moxibustion"[Title/Abstract] OR "partitioned moxibustion"[Title/Abstract] OR "separated moxibustion"[Title/Abstract] OR "ginger moxibustion"[Title/Abstract] OR "ginger-separated moxibustion"[Title/Abstract] OR "ginger partition moxibustion"[Title/Abstract] OR "ginger-partitioned moxibustion"[Title/Abstract] OR "herb-partitioned moxibustion"[Title/Abstract] OR "herb-separated moxibustion"[Title/Abstract] OR "medicinal moxibustion"[Title/Abstract] OR "drug-separated moxibustion"[Title/Abstract] OR "governor vessel moxibustion"[Title/Abstract] OR "du moxibustion"[Title/Abstract] OR "du meridian moxibustion"[Title/Abstract] OR "heat-sensitive moxibustion"[Title/Abstract] OR "thermosensitive moxibustion"[Title/Abstract] OR "thunder-fire moxibustion"[Title/Abstract] OR "thunder-fire moxibustion"[Title/Abstract] OR "mild moxibustion"[Title/Abstract] OR "fire dragon pot moxibustion"[Title/Abstract] OR "fire dragon moxibustion"[Title/Abstract] OR "fire dragon pot"[Title/Abstract] OR "fire dragon cupping"[Title/Abstract])) AND (randomizedcontrolledtrial[Filter]) 3 |
|  |  |
|  |  |
|  |  |
|  |  |
|  |  |
|  |  |
| Web of Science | #1  "TS=(""Diabetic Neuropathies"" OR ""Amyotrophies, Diabetic"" OR ""Amyotrophy, Diabetic"" OR ""Diabetic Amyotrophy"" OR ""Diabetic Amyotrophies"" OR ""Symmetric Diabetic Proximal Motor Neuropathy"" OR ""Asymmetric Polyneuropathy, Diabetic"" OR ""Asymmetric Polyneuropathies, Diabetic"" OR ""Diabetic Asymmetric Polyneuropathies"" OR ""Diabetic Asymmetric Polyneuropathy"" OR ""Polyneuropathy, Diabetic Asymmetric"" OR ""Polyneuropathies, Diabetic Asymmetric"" OR ""Diabetic Mononeuropathy Simplices"" OR ""Mononeuropathy Simplices, Diabetic"" OR ""Simplices, Diabetic Mononeuropathy"" OR ""Diabetic Mononeuropathy"" OR ""Mononeuropathy Simplex, Diabetic"" OR ""Mononeuropathies, Diabetic"" OR ""Diabetic Mononeuropathy Simplex"" OR ""Mononeuropathy, Diabetic"" OR ""Simplex, Diabetic Mononeuropathy"" OR ""Diabetic Mononeuropathies"" OR ""Neuropathy, Diabetic Autonomic"" OR ""Autonomic Neuropathy, Diabetic"" OR ""Autonomic Neuropathies, Diabetic"" OR ""Diabetic Autonomic Neuropathy"" OR ""Diabetic Autonomic Neuropathies"" OR ""Neuropathies, Diabetic Autonomic"" OR ""Asymmetric Diabetic Proximal Motor Neuropathy"" OR ""Polyneuropathy, Diabetic"" OR ""Diabetic Polyneuropathy"" OR ""Polyneuropathies, Diabetic"" OR ""Diabetic Polyneuropathies"" OR ""Painful Diabetic Neuropathies"" OR ""Diabetic Neuralgia"" OR ""Neuropathies, Painful Diabetic"" OR ""Diabetic Neuropathy, Painful"" OR ""Neuralgia, Diabetic"" OR ""Neuropathy, Painful Diabetic"" OR ""Painful Diabetic Neuropathy"" OR ""Diabetic Neuralgias"" OR ""Neuralgias, Diabetic"" OR ""Diabetic Neuropathies, Painful"" OR ""Diabetic Neuropathy"" OR ""Neuropathy, Diabetic"" OR ""Neuropathies, Diabetic"") " Web of Science Core Collection 16943  #2  "TS=(""Moxibustion"" OR ""moxibustion"" OR ""moxabustion"" OR ""moxa"" OR ""moxa therapy"" OR ""moxibustion therapy"" OR ""warm needling"" OR ""warm needle"" OR ""warm-needling"" OR ""warming needle moxibustion"" OR ""warm acupuncture"" OR ""warm acupuncture and moxibustion"" OR ""warming needle"" OR ""indirect moxibustion"" OR ""partitioned moxibustion"" OR ""interposed moxibustion"" OR ""separated moxibustion"" OR ""ginger moxibustion"" OR ""ginger-separated moxibustion"" OR ""ginger partition moxibustion"" OR ""ginger-partitioned moxibustion"" OR ""herb-partitioned moxibustion"" OR ""herb-separated moxibustion"" OR ""medicinal moxibustion"" OR ""drug-separated moxibustion"" OR ""governor vessel moxibustion"" OR ""du moxibustion"" OR ""du meridian moxibustion"" OR ""heat-sensitive moxibustion"" OR ""heat sensitization moxibustion"" OR ""thermal sensitization moxibustion"" OR ""thermosensitive moxibustion"" OR ""thunder-fire moxibustion"" OR ""thunder fire moxibustion"" OR ""mild moxibustion"" OR ""gentle moxibustion"" OR ""fire dragon pot moxibustion"" OR ""fire dragon moxibustion"" OR ""fire dragon pot"" OR ""fire dragon cupping"") " Web of Science Core Collection 3818  #3  "TS=(  random* OR randomi* OR randomly  OR placebo* OR sham  OR trial OR ""clinical trial"" OR ""controlled clinical trial""  OR ""double blind"" OR ""single blind"" OR ""triple blind""  OR crossover OR ""cross over"" OR ""parallel group""  OR cluster random*  OR RCT  ) " Web of Science Core Collection 4759062  #4 "#3 AND #2 AND #1 " Web of Science Core Collection 2 |
|  |  |
|  |  |
|  |  |
|  |  |
|  |  |
|  |  |
|  |  |
|  |  |
| Cochrane | #1 MeSH descriptor: [Diabetic Neuropathies] explode all trees 3292  #2 "Diabetic Neuropathies" OR "Amyotrophies, Diabetic" OR "Amyotrophy, Diabetic" OR "Diabetic Amyotrophy" OR "Diabetic Amyotrophies" OR "Symmetric Diabetic Proximal Motor Neuropathy" OR "Asymmetric Polyneuropathy, Diabetic" OR "Asymmetric Polyneuropathies, Diabetic" OR "Diabetic Asymmetric Polyneuropathies" OR "Diabetic Asymmetric Polyneuropathy" OR "Polyneuropathy, Diabetic Asymmetric" OR "Polyneuropathies, Diabetic Asymmetric" OR "Diabetic Mononeuropathy Simplices" OR "Mononeuropathy Simplices, Diabetic" OR "Simplices, Diabetic Mononeuropathy" OR "Diabetic Mononeuropathy" OR "Mononeuropathy Simplex, Diabetic" OR "Mononeuropathies, Diabetic" OR "Diabetic Mononeuropathy Simplex" OR "Mononeuropathy, Diabetic" OR "Simplex, Diabetic Mononeuropathy" OR "Diabetic Mononeuropathies" OR "Neuropathy, Diabetic Autonomic" OR "Autonomic Neuropathy, Diabetic" OR "Autonomic Neuropathies, Diabetic" OR "Diabetic Autonomic Neuropathy" OR "Diabetic Autonomic Neuropathies" OR "Neuropathies, Diabetic Autonomic" OR "Asymmetric Diabetic Proximal Motor Neuropathy" OR "Polyneuropathy, Diabetic" OR "Diabetic Polyneuropathy" OR "Polyneuropathies, Diabetic" OR "Diabetic Polyneuropathies" OR "Painful Diabetic Neuropathies" OR "Diabetic Neuralgia" OR "Neuropathies, Painful Diabetic" OR "Diabetic Neuropathy, Painful" OR "Neuralgia, Diabetic" OR "Neuropathy, Painful Diabetic" OR "Painful Diabetic Neuropathy" OR "Diabetic Neuralgias" OR "Neuralgias, Diabetic" OR "Diabetic Neuropathies, Painful" OR "Diabetic Neuropathy" OR "Neuropathy, Diabetic" OR "Neuropathies, Diabetic" 3890  #3 #1 or #2 5449  #4 "Moxibustion" OR "moxibustion" OR "moxabustion" OR "moxa" OR "moxa therapy" OR "moxibustion therapy" OR "warm needling" OR "warm needle" OR "warm-needling" OR "warming needle moxibustion" OR "warm acupuncture" OR "warm acupuncture and moxibustion" OR "warming needle" OR "indirect moxibustion" OR "partitioned moxibustion" OR "interposed moxibustion" OR "separated moxibustion" OR "ginger moxibustion" OR "ginger-separated moxibustion" OR "ginger partition moxibustion" OR "ginger-partitioned moxibustion" OR "herb-partitioned moxibustion" OR "herb-separated moxibustion" OR "medicinal moxibustion" OR "drug-separated moxibustion" OR "governor vessel moxibustion" OR "du moxibustion" OR "du meridian moxibustion" OR "heat-sensitive moxibustion" OR "heat sensitization moxibustion" OR "thermal sensitization moxibustion" OR "thermosensitive moxibustion" OR "thunder-fire moxibustion" OR "thunder fire moxibustion" OR "mild moxibustion" OR "gentle moxibustion" OR "fire dragon pot moxibustion" OR "fire dragon moxibustion" OR "fire dragon pot" OR "fire dragon cupping" 7399  #5 #3 and #4 29 |
|  |  |
|  |  |
|  |  |
| Embase | #1 'diabetic neuropathy'/exp OR 'diabetes mellitus with neuropathy' OR 'diabetes neuropathy' OR 'diabetic mononeuritis' OR 'diabetic mononeuropathy' OR 'diabetic neuritis' OR 'diabetic neuropathies' OR 'diabetic peripheral neuropathy' OR 'diabetic peripheral polyneuropathy' OR 'diabetic polyneuritis' OR 'diabetic polyneuropathy' OR 'diabetic sensorimotor polyneuropathy' OR 'neuropathies in diabetes' OR 'neuropathy in diabetes' OR 'peripheral diabetic neuropathy' OR 'polyneuropathy in diabetes' OR 'diabetic neuropathy' 40741  #2 'moxibustion'/exp OR 'moxibustion' OR 'moxabustion' OR 'moxa' OR 'moxa therapy' OR 'moxibustion therapy' OR 'warm needling' OR 'warm needle' OR 'warm-needling' OR 'warming needle moxibustion' OR 'warm acupuncture' OR 'warm acupuncture and moxibustion' OR 'warming needle' OR 'indirect moxibustion' OR 'partitioned moxibustion' OR 'interposed moxibustion' OR 'separated moxibustion' OR 'ginger moxibustion' OR 'ginger-separated moxibustion' OR 'ginger partition moxibustion' OR 'ginger-partitioned moxibustion' OR 'herb-partitioned moxibustion' OR 'herb-separated moxibustion' OR 'medicinal moxibustion' OR 'drug-separated moxibustion' OR 'governor vessel moxibustion' OR 'du moxibustion' OR 'du meridian moxibustion' OR 'heat-sensitive moxibustion' OR 'heat sensitization moxibustion' OR 'thermal sensitization moxibustion' OR 'thermosensitive moxibustion' OR 'thunder-fire moxibustion' OR 'thunder fire moxibustion' OR 'mild moxibustion' OR 'gentle moxibustion' OR 'fire dragon pot moxibustion' OR 'fire dragon moxibustion' OR 'fire dragon pot' OR 'fire dragon cupping' 21319  #3 #1 AND #2 110  #4 #3 AND 'randomized controlled trial'/de 26 |
| China National Knowledge Infrastructure | (SU = diabetic peripheral neuropathy OR SU = diabetic peripheral diabetic neuropathy OR SU = diabetic neuropathy) AND (SU = moxibustion OR SU = warm needling moxibustion OR SU = indirect moxibustion OR SU = ginger-partitioned moxibustion OR SU = herbal-partitioned moxibustion OR SU = heat-sensitive moxibustion OR SU = thunder-fire moxibustion OR SU = mild moxibustion OR SU = governor vessel moxibustion OR SU = Huolongcuan comprehensive moxibustion) AND (SU = randomized controlled trial OR SU = clinical observation OR SU = efficacy OR SU = randomized) 135 |
| Wanfang Medical Database | (Topic: (diabetic peripheral neuropathy OR diabetic peripheral diabetic neuropathy OR diabetic neuropathy)) AND (Topic: (moxibustion OR warm needling moxibustion OR indirect moxibustion OR ginger-partitioned moxibustion OR herbal-partitioned moxibustion OR thunder-fire moxibustion OR heat-sensitive moxibustion OR mild moxibustion OR governor vessel moxibustion OR Huolongcuan comprehensive moxibustion)) AND (Topic: (randomized controlled trial OR randomized OR efficacy OR clinical observation)) 236 |
| VIP Database | (SU = diabetic peripheral neuropathy OR SU = diabetic peripheral diabetic neuropathy OR SU = diabetic neuropathy) AND (SU = moxibustion OR SU = warm needling moxibustion OR SU = indirect moxibustion OR SU = ginger-partitioned moxibustion OR SU = herbal-partitioned moxibustion OR SU = heat-sensitive moxibustion OR SU = thunder-fire moxibustion OR SU = mild moxibustion OR SU = governor vessel moxibustion OR SU = Huolongcuan comprehensive moxibustion) AND (SU = randomized controlled trial OR SU = clinical observation OR SU = efficacy OR SU = randomized) 157 |

Table S3 References

1.	Ma G, Ye T, Sun Z. Warming Acupuncture Combined with Conventional Acupuncture for Diabetic Peripheral Neuropathy with Syndrome of Yang Deficiency and Cold Coagulation, Obstruction of Collaterals and Blood Stasis. *Chinese Acupuncture & Moxibustion* (2018) 38(3):229–32. doi: 10.13703/j.0255-2930.2018.03.001.

2.	Wang J, Mao Y, Jiang C, He M, Tan Q, Zheng W. Efficacy of Combined Application of Traditional Chinese Medicine Foot Bath and Mild Moxibustion in the Treatment O F Limb Pain in Diabetic Peripheral Neuropathy Patients. *Tropical Journal of Pharmaceutical Research* (2023) 22(1):175–81. doi: 10.4314/tjpr.v22i1.24.

3.	Fang X-j, Zheng G-x, Huangfu Y-h, Yu N-t. Observation on Therapeutic Efficacy of Heat-Sensitive Moxibustion Plus Western Medicine for Diabetic Peripheral Neuropathy. *Journal of Acupuncture and Tuina Science* (2020) 18(6):452–7. doi: 10.1007/s11726-020-1214-8.

4.	Feng Y, Li H. Clinical Effect of Tongxinluo Capsule Combined with Warm Needle Moxibustion Therapy in the Treatment of Diabetic Peripheral Neuropathy. *Clinical Research and Practice* (2022) 7(9):147–50. doi: 10.19347/j.cnki.2096-1413.202209040.

5.	Xie L. Moxibustion Combined with Acupoint Massage in the Treatment of Diabetes Peripheral Neuropathy. *Chinese Medicine Modern Distance Education of China* (2025) 23(4):116–8. doi: 10.3969/j.issn.1672⁃2779.2025.04.039.

6.	Wang D. Clinical Observation of Moxibustion Combined with Mecobalamin in the Treatment of Diabetic Peripheral Neuropathy. *Journal of Practical Traditional Chinese Medicine* (2021) 37(6):1042–4.

7.	Ji H, Wu H, Wang K. Effect of Moxibustion at Zusanli Combined with Mecobalamin on Traditional Chinese Medicine Syndrome Score and Impaired Nerve Conduction Velocity in Patients with Diabetic Peripheral Neuropathy. *Chinese Journal of Practical Medicine* (2025) 52(14):104–7. doi: 10.3760/cma.j.cn115689-20241118-02885.

8.	Bai F, Li B. Clinical Observation of Mild Moxibustion Combined with Huangqi Guizhi Wuwu Decoction in the Treatment of Diabetic Peripheral Neuropathy with Qi Deficiency and Blood Stasis Syndrome. *Practical Clinical Journal of Integrated Traditional Chinese and Western Medicine* (2025) 25(22):11–3. doi: 10.13638/j.issn.1671-4040.2025.22.003.

9.	Tang H. Nursing Effect of Ginger Partitioned Moxibustion Combined with Foot Nursing for Patients with Diabetic Peripheral Neuropathy with Blood Stasis Syndrome. *Chinese Manipulation & Rehabilitation Medicine* (2022) 3(1):51–4.

10.	Wu N, Liu Y, Wei J, Jin X, Sheng J, Wang Y, et al. Therapeutic Effect of Ginger-Partition Moxibustion Therapy on Diabetes Patients with Peripheral Neuropathy and Its Influence on Hs-Crp. *Modern Journal of Integrated Traditional Chinese and Western Medicine* (2015) 24(1):24–6. doi: 10.3969/j.issn.1008-8849.2015.01.008.

11.	Zhang X, Yang J, Ma X, Wang K, Yao J. Effect of Thunder-Fire Moxibustion Combined with Huangqi Guizhi Wuwu Decoction on Electromyography, Hemorheology and Quality of Life for Patients with Type 2 Diabetic Peripheral Neuropathy. *Journal of Sichuan of Traditional Chinese Medicine* (2020) 38(11):140–4.

12.	Xu X. Analysis of the Effect of Acupoint Application and Ginger Moxibustion in the Treatment of Diabetic Peripheral Neuropathy. *Guide of China Medicine* (2025) 23(18):156–8. doi: 10.15912/j.issn.1671-8194.2025.18.046.

13.	Zhang S, Li X, Huo Y, Fan Z, Kang Y, Xue W. Clinical Study of Warm Acupuncture Combined with Huazhuo Tongluo Formula in the Treatment of Diabetic Peripheral Neuropathy with Phlegm and Blood Stasis Obstruction Syndrome. *Journal of Hebei Traditional Chinese Medicine and Pharmacology* (2024) 39(2):37–40. doi: 10.16370/j.cnki.13-1214/r.2024.02.005.

14.	Zhang X, Yang J, Ma X, Wang K, Yao J. Clinical Observation of Modified Huangqi Guizhi Wuwu Decoction Combined with Thunder-Fire Moxibustion in the Treatment of Diabetic Peripheral Neuropathy. *Hebei Journal of Traditional Chinese Medicine* (2020) 42(12):1866–70. doi: 10.3969/j.issn.1002-2619.2020.12.022.

15.	Hu G. Clinical Study on the Treatment of Type 2 Diabetic Peripheral Neuropathy with Supplemented Astragalus and Cinnamon Twig Five Substances Decoction Combined with Warming Needle Moxibustion. *Henan Traditional Chinese Medicine* (2024) 44(1):18–22. doi: 10.16367/j.issn.1003-5028.2024.01.0005.

16.	Liu X, Dai F, Yue X, Li B, Kong D. Clinical Observation on Jingluoshu Capsule Combined with Mild-Warm Moxibustion in Treatment of Diabetic Peripheral Neuropathy. *Liaoning Journal of Traditional Chinese Medicine* (2012) 39(8):1559–61. doi: 10.13192/j.ljtcm.2012.08.126.liuxx.058.

17.	Xu L, Qu Y, Li S, Miao X, Shi H, Zhu R. Clinical Observation of Thunder-Fire Moxibustion Combined with Intradermal Needle in the Treatment of Diabetic Peripheral Neuropathy with Qi-Yin Deficiency and Blood Stasis Syndrome. *Journal of Sichuan of Traditional Chinese Medicine* (2021) 39(6):183–6.

18.	Wan Z. Effects of Thunder-Fire Moxibustion Combined with Shuluo Tongmai Decoction on Patients with Diabetic Peripheral Neuropathy of Cold Coagulation and Blood Stasis Type. *The Journal of Medical Theory and Practice* (2025) 38(15):2568–71. doi: 10.19381/j.issn.1001-7585.2025.15.013.

19.	Li S-m, Shao J-g, Chen L, Da K-l, Han Y, Zhang Y. Efficacy Observation of Thunder-Fire Moxibustion on Painful Diabetic Neuropathy with Syndrome of Blood Stasis Due to Cold Coagulation. *Shanxi Journal of Traditional Chinese Medicine* (2024) 40(6):37–8. doi: 10.20002/j.issn.1000-7156.2024.06.014.

20.	Du M, Dong J, Huang Z, Zhang D. Effects of Warm Acupuncture on Nerve Conduction and Blood Glucose Metabolism in Patients with Diabetic Peripheral Neuropathy. *World Chinese Medicine* (2019) 14(3009-3012):3009. doi: 10.3969/j.issn.1673-7202.2019.11.040.

21.	Wu M, Zhang H, Li Z-f, He Z-z, Hong E-s. Clinical Application of Acupoints Thermal Moxibustion Combined with Vitamin B12 Acupoint Injection in the Treatment of Diabetic Peripheral Neuropathy. *Journal of Jiangxi University of Traditional Chinese Medicine* (2017) 29:62–4.

22.	Jin Z, Wang Q, Huang L, Ding L, Yao Q, Fan Y, et al. Clinical Observation on Treating Diabetic Peripheral Neuropathy with Needle Warming Moxibustion of Hands and Feet. *Hebei Journal of Traditional Chinese Medicine* (2020) 42(9):1374–8. doi: 10.3969/j.issn.1002-2619.2020.09.021.

23.	Luo X, Fei A, Wu H, Guo F, Jin Y, Hao L, et al. Clinical Observation on the Treatment of Diabetic Peripheral Neuropathy by Mild Moxibustion at Back-Shu Point and Acupuncture. *Journal of Jiangxi University of Traditional Chinese Medicine* (2020) 32(2):62–4.

24.	Li J, Fu C. Therapeutic Efficacy of Warm Acupuncture Combined with Shuluo Tongmai Decoction in Treating Diabetic Peripheral Neuropathy of Cold Congealing and Blood Stasis Type: A Clinical Analysis. *Information on Traditional Chinese Medicine* (2025) 42(4):40–4. doi: 10.19656/j.cnki.1002-2406.20250407.

25.	Wang Y, Yuan X, Guo S. Effect of Warming Needle Moxibustion Combined with Acupoint Catgut Embedding on Diabetic Peripheral Neuropathy. *Jilin Journal of Chinese Medicine* (2022) 42:850–3. doi: 10.13463/j.cnki.jlzyy.2022.07.027.

26.	Feng X, Xiang J, Li Q, Cao Z. Effect of Warming Needle Moxibustion Combined with Epalrestat on Elderly Patients with Diabetic Peripheral Neuropathy. *Chinese Journal of Gerontolog* (2024) 44(19):4681–4. doi: 10.3969/j.issn.1005-9202.2024.19.020.

27.	Du X, Wei N, Zhao F. Investigation of the Effect of Warm Acupuncture and Moxibustion Combined with Yiqi Ziyin Formula on Neurophysiology in Patients with Diabetic Peripheral Neuropathy. *Diabetes New World* (2023) (20):181–4. doi: 10.16658/j.cnki.1672-4062.2023.20.181.

28.	Wang L, Fang Z, Fei A, Pan L, Hu P, Ge X, et al. Effect of Warming Needle Moxibustion Combined with Yiqi Ziyin Decoction on Oxidative Stress Level and Nerve Electrophysiology in Patients with Diabetic Peripheral Neuropathy. *Shaanxi Journal of Traditional Chinese Medicine* (2022) 43(2):248–51. doi: 10.3969/j.issn.1000-7369.2022.02.026.

29.	Ma M. Therapeutic Effect of Warm Acupuncture on Diabetic Peripheral Neuropathy. *Diabetes New World* (2024) (7):12–5. doi: 10.16658/j.cnki.1672-4062.2024.07.012.

30.	Dong Q, Zhao H, Peng S, Zhang L, Xiao X, Wu Jn. Effect Analysis of Stone Needle Warm Moxibustion Therapy Combined with Conventional Therapy on Diabetic Peripheral Neuropathy. *Chinese Community Doctors* (2023) 39(33):71–3. doi: 10.3969/j.issn.1007-614x.2023.33.024.

31.	Li X, Wang F, Liang J, Li D. Acupuncture Combined with Moxibustion in the Treatment of 40 Cases of Diabetic Peripheral Neuropathy. *Medical Information* (2014) 27(8):341–2.

32.	Luo X, Liu S, Feng M. Effects of Foot Bath with Traditional Chinese Medicine Combined with Thunder-Fire Moxibustion on Diabetic Peripheral Neuropathy. *Chinese General Practice Nursing* (2022) 20(4):507–9. doi: 10.12104/j.issn.1674-4748.2022.04.018.

33.	Wang J, Zhan J, Li D, Lv J, Yao J, Xu C, et al. Research on Application of Chinese Medicine Foot Bath Combined with Mild Moxibustion in Diabetic Peripheral Neuropathy Patients. *Nursing and Rehabilitation* (2023) 22(7):6–10. doi: 10.3969/j.issn.1671-9875.2023.07.002.

34.	Hu S, Liu M, Liu Y, Zou P, Jiang L, Zhuang Q. Clinical Study of Directional Permeation of Traditional Chinese Medicine Combined with Heat Sensitive Moxibustion on Diabetic Peripheral Neuropathy. *Modern Chinese Medicin* (2026) 46(1):23–7. doi: 10.13424/j.cnki.mtcm.2026.01.004.

35.	Liu M, Sun X, Wang H, Fu D, Liu L. Clinical Observation of Heat-Sensitive Moxibustion Combined with Fushi Tongbi Decoction as an Adjunctive Treatment for Diabetic Peripheral Neuropathy. *Journal of Practical Traditional Chinese Medicine* (2023) 39(12):2393–5.

36.	Huang X, Feng X, Zheng Y. Analysis of the Effect of Huolongguan Comprehensive Moxibustion Combined with Transdermal Acupoint Application on Improving Diabetic Peripheral Neuropathy Patients. *Journal of Medicine and Pharmacy of Chinese Minorities* (2024) 30(6):23–5. doi: 10.16041/j.cnki.cn15-1175.2024.06.027.

37.	Peng J. Clinical Observation of Huolongguan Comprehensive Moxibustion Combined with Transdermal Acupoint Application in the Treatment of Diabetic Peripheral Neuropathy. *Journal of Practical Traditional Chinese Medicine* (2022) 38(8):1390–2.

38.	Liang G, Li M, Long Y, Luo D, Liao T. Observation on Clinical Effect of Fire Dragon Jar Comprehensive Moxibustion Therapy Combined with Conventional Western Medicine in the Intervention of Diabetic Peripheral Neuropathy. *Chinese and Foreign Medical Research* (2025) 23(11):165–9. doi: 10.14033/j.cnki.cfmr.2025.11.044.

39.	Hu S, Liu M, Mu W, Huang M. Heat-Sensitive Moxibustion in the Treatment of Type 2 Diabetic Peripheral Neuropathy. *Chinese Medicine Modern Distance Education of China* (2026) 24(1):143–5. doi: 10.3969/j.issn.1672⁃2779.2026.01.040.

40.	Hu G. Clinical Study of Buyang Huanwu Decoction Combined with Ginger-Separated Moxibustion as an Adjunctive Treatment for Diabetic Peripheral Neuropathy. *Journal of Practical Traditional Chinese Medicine* (2023) 39(6):1109–11.

Table S4: Detailed characteristics of the included studies

| **No.** | **First author and year of publication** | **Disease** | **Age（mean±sd)** | **Sex (male/female)** | **Duration of intervention** | **Country** | **Reasearch Ttpe** |
| --- | --- | --- | --- | --- | --- | --- | --- |
| 1 | Ma Guoqing 2018 | DPN | 55±6/56±5 | 31/33 | 28 | CHINA | RCT |
| 2 | Wang Jiawei 2023 | DPN | 60.65±12.16/59.33±9.69 | 81/39 | 60 | CHINA | RCT |
| 3 | Fang Xiujuan 2020 | DPN | 56.8±5.1/54.3±5.0 | 33/30 | 28 | CHINA | RCT |
| 4 | Feng Yun 2022 | DPN | 52.48±7.64/53.71±8.06 | 178/148 | 15 | CHINA | RCT |
| 5 | Xie Lingling 2025 | DPN | 54.71±5.36/54.76±5.31 | 51/45 | 20 | CHINA | RCT |
| 6 | Wang Dan 2021 | DPN | 51.73±2.62/48.65±2.73 | 58/67 | 28 | CHINA | RCT |
| 7 | Ji Hua 2025 | DPN | 66.83±3.24/66.79±3.18 | 70/52 | 28 | CHINA | RCT |
| 8 | Bai Fubin 2025 | DPN | 57.12±3.74/57.41±3.10 | 48/32 | 43 | CHINA | RCT |
| 9 | Tang Huazhen 2022 | DPN | 57.53±1.58/57.49±1.61 | 33/29 | 56 | CHINA | RCT |
| 10 | Wu Naijun 2015 | DPN | 58.76±7.92/60.69±7.84 | 27/25 | 28 | CHINA | RCT |
| 11 | Zhang Xiujun 2020 | DPN | 61.56±7.12/62.41±8.45 | 90/66 | 56 | CHINA | RCT |
| 12 | Xu Xiaowei 2025 | DPN | 59.08±5.53/58.56±4.28 | 54/36 | 14 | CHINA | RCT |
| 13 | Zhang Suzhao 2024 | DPN | 56.80±7.59/56.75±6.85 | 43/37 | 28 | CHINA | RCT |
| 14 | Zhang Xiujun 2020 | DPN | 57.4±6.61/56.19 ± 7.23 | 96/64 | 56 | CHINA | RCT |
| 15 | Hu Guanghui 2024 | DPN | 51.49±7.33/51.38±7.26 | 74/26 | 90 | CHINA | RCT |
| 16 | Liu Xiangxiu 2012 | DPN | 57.8±3.18/56.9±2.29 | 51/29 | 30 | CHINA | RCT |
| 17 | Xu Lilii 2021 | DPN | 56.16±8.25/56.92±10.58 | 69/51 | 42 | CHINA | RCT |
| 18 | Wan Zhihui 2025 | DPN | 62.15±4.02/62.48±4.11 | 41/47 | 14 | CHINA | RCT |
| 19 | Li Shuomiao 2024 | DPN | 59.10±1.47/59.77±1.72 | 30/30 | 14 | CHINA | RCT |
| 20 | Du Minzhen 2019 | DPN | 54.74±8.19/54.67±7.03 | 50/30 | 29 | CHINA | RCT |
| 21 | Wu Min 2017 | DPN | 63.3±7.1/61.3±7.4 | 24/36 | 28 | CHINA | RCT |
| 22 | Jin Zhu 2020 | DPN | 62.18±6.10/61.87±5.92 | 38/22 | 28 | CHINA | RCT |
| 23 | Luo Xingzi 2020 | DPN | 53.23±7.78/53.90±8.86 | 28/34 | 21 | CHINA | RCT |
| 24 | Li Jianshu 2025 | DPN | 61.55±9.43/62.88 ± 8.94 | 51/29 | 14 | CHINA | RCT |
| 25 | Wang Yue 2022 | DPN | 60.45±10.81/60.72±10.52 | 57/63 | 28 | CHINA | RCT |
| 26 | Feng Xueqiang 2024 | DPN | 69.85±3.40/69.87±3.36 | 51/31 | 19 | CHINA | RCT |
| 27 | Du Xinze 2023 | DPN | 65.48±2.51/65.51±2.54 | 61/42 | 90 | CHINA | RCT |
| 28 | Wang Lina 2022 | DPN | 58.36±7.8/61.02±6.7 | 33/27 | 90 | CHINA | RCT |
| 29 | Ma Mengmeng 2024 | DPN | 68.67±7.39/68.74±7.42 | 39/43 | 28 | CHINA | RCT |
| 30 | Dong Qingmei 2023 | DPN | 57.33±9.81/56.92±8.37 | 36/24 | 365 | CHINA | RCT |
| 31 | Li Xiaoting 2014 | DPN | 49.51±3.4/51.32±9.1 | 21/19 | 28 | CHINA | RCT |
| 32 | Luo Xiaohong 2022 | DPN | 50.42±5.23/50.36±5.18 | 48/34 | 28 | CHINA | RCT |
| 33 | Wang Jiawei 2023 | DPN | 60.50±12.04/59.46±9.82 | 69/45 | 56 | CHINA | RCT |
| 34 | Hu Shuhan 2026 | DPN | 66.14±5.33 /67.60±4.27/65.93±7.01 | 43/23 | 14 | CHINA | RCT |
| 35 | Liu Maomao 2023 | DPN | 56.13±7.21/54.38±6.95 | 38/35 | 28 | CHINA | RCT |
| 36 | Huang Xingduan 2024 | DPN | 56.44±2.51/55.32±2.47 | 45/15 | 14 | CHINA | RCT |
| 37 | Peng Jing 2022 | DPN | 58.65±9.40/58.62±9.33 | 70/64 | 14 | CHINA | RCT |
| 38 | Liang Guoyu 2025 | DPN | 59.71±6.20/59.60±6.18 | 55/41 | 14 | CHINA | RCT |
| 39 | Hu Shuhan 2026 | DPN | 68.35±5.37/68.49±6.01 | 31/29 | 14 | CHINA | RCT |
| 40 | Hu Guanghui 2023 | DPN | 53.69±2.60/53.75±2.68 | 79/21 | 28 | CHINA | RCT |

Table S5 Detailed characteristics of the included studies

| **No.** | **experimental group** | **Control Group** | **Experimental group(n)** | **Control group (n)** | **Common peroneal MNCV** | **Median MNCV** | **Common peroneal SNCV** | **Median SNCV** |
| --- | --- | --- | --- | --- | --- | --- | --- | --- |
| 1 | MOXI | ACU | 32 | 32 | 36.19±3.33/36.82±2.99 | NR | NR | NR |
| 2 | MOXI+EHM | CT | 60 | 60 | NR | NR | NR | NR |
| 3 | MOXI | CT | 31 | 32 | NR | NR | NR | NR |
| 4 | MOXI+IHM | CT | 163 | 163 | 42.79±3.45/43.16±3.38 | 45.23±3.49/44.81±3.52 | 34.41±2.91/34.75±2.97 | 33.62±2.79/34.17±2.83 |
| 5 | MOXI+MT | CT | 48 | 48 | 31.75±2.45/31.74±2.46 | 37.25±3.47/37.18±3.71 | NR | NR |
| 6 | MOXI | CT | 63 | 62 | 34.64±5.26/34.63±5.28 | 37.14±4.73/37.15±4.71 | 29.62±5.14/29.63±5.12 | 32.46±4.31/32.47±4.29 |
| 7 | MOXI | CT | 61 | 61 | 37.96±5.20/37.92±5.14 | 34.89±4.7/34.93±4.73 | 36.09±3.94/36.12±4.08 | NR |
| 8 | MOXI+IHM | IHM | 40 | 40 | 36.72±4.67/36.56±4.15 | NR | 35.53±3.68/35.10±3.59 | NR |
| 9 | MOXI+MT | CT | 31 | 31 | 39.67±4.23/39.61±4.21 | 39.55±4.59/39.56±4.51 | 35.23±4.21/35.19±4.12 | NR |
| 10 | MOXI | CT | 26 | 26 | 36.07±1.87/37.16±2.11 | NR | NR | NR |
| 11 | MOXI+IHM | CT | 78 | 78 | NR | 38.41±4.79/37.31±4.11 | NR | 34.50±5.73/32.59±5.27 |
| 12 | MOXI+EHM | CT | 45 | 45 | 39.68±4.24/39.62±4.22 | 39.54±4.58/39.57±4.52 | NR | 36.30±4.28/36.12±4.15 |
| 13 | MOXI+IHM | CT | 40 | 40 | 35.65±2.45/35.68±2.60 | 42.23±3.08/43.04±2.65 | 36.08±2.36/36.36±2.09 | 38.19±2.18/38.20±2.29 |
| 14 | MOXI+IHM | CT | 80 | 80 | NR | 42.67±3.45/41.83±4.17 | NR | 35.42±4.24/34.59±5.19 |
| 15 | MOXI+IHM | CT | 50 | 50 | 40.13±2.88/40.05±2.73 | 41.47±5.06/41.61±4.98 | 32.71±3.79/32.68±3.62 | 38.04±3.76/37.91±3.68 |
| 16 | MOXI+IHM | CT | 40 | 40 | 39.7±3.2/39.8±3.2 | 39.6±4.5/39.4±4.8 | NR | 36.5±3.6/36.4±3.8 |
| 17 | MOXI+ACU | CT | 60 | 60 | 26.14±2.51/26.79±2.82 | 30.51±3.59/31.11±4.52 | 25.01±3.32/25.07±2.84 | 27.34±2.86/28.41±2.32 |
| 18 | MOXI+EHM | MOXI | 44 | 44 | 33.82±3.05/34.01±3.11 | 37.75±3.85/37.82±3.72 | 34.81±3.35/33.96±3.40 | 34.26±3.20/34.21±3.17 |
| 19 | MOXI | CT | 30 | 30 | NR | NR | NR | NR |
| 20 | MOXI | CT | 40 | 40 | NR | NR | 32.08±2.87/31.57±3.04 | 40.95±3.05/41.27±3.28 |
| 21 | MOXI | CT | 30 | 30 | 38.78±3.38/38.63±2.75 | NR | NR | NR |
| 22 | MOXI | CT | 30 | 30 | 40.10±2.61/39.82±3.87 | 41.30±2.49/39.72±4.38 | 35.89±4.12/36.28±4.35 | 38.30±2.60/37.92±2.75 |
| 23 | MOXI+ACU | ACU | 31 | 31 | NR | NR | 34.29±3.15/34.42±2.99 | NR |
| 24 | MOXI+EHM | CT | 40 | 40 | NR | NR | NR | 40.71±5.15/40.86±5.01 |
| 25 | MOXI+ACU | CT | 60 | 60 | 33.14±6.15/32.62±6.07 | NR | NR | NR |
| 26 | MOXI | ACU | 41 | 41 | 38.49±4.11/38.54±4.15 | 40.98±4.85/40.88±4.90 | NR | 35.94±4.57/35.97±4.48 |
| 27 | MOXI+IHM | CT | 52 | 51 | 40.47±3.16/40.52±3.1 | NR | NR | NR |
| 28 | MOXI+IHM | CT | 30 | 30 | NR | NR | NR | NR |
| 29 | MOXI | CT | 41 | 41 | 38.62±1.8639.29±1.79 | NR | 28.39±1.45/28.46±1.48 | NR |
| 30 | MOXI | CT | 30 | 30 | NR | NR | NR | NR |
| 31 | MOXI+ACU | CT | 20 | 20 | 35.70±1.74/36.33±1.58 | NR | NR | NR |
| 32 | MOXI+EHM | CT | 41 | 41 | 35.02±3.19/34.89±3.15 | NR | NR | NR |
| 33 | MOXI+EHM | CT | 58 | 56 | 27.29±4.96/26.43±5.11 | NR | 24.38±5.23/25.80±5.99 | NR |
| 34 | MOXI+EHM | EHM/MOXI | 22 | 22/22 | 38.88±4.54/38.70±2.55/38.78±3.41 | NR | NR | NR |
| 35 | MOXI+IHM | CT | 36 | 37 | NR | NR | 37.52±3.09/37.66±3.12 | 38.63±2.71/38.94±3.04 |
| 36 | MOXI+EHM | EHM | 30 | 30 | NR | NR | 37.94±2.84/38.21±2.91 | 42.97±2.55/43.11±2.49 |
| 37 | MOXI+EHM | EHM | 67 | 67 | 41.98±3.26/42.02±3.28 | 36.64±4.28/36.71±4.31 | 37.66±4.00/37.71±4.02 | 34.19±3.91/34.22±3.92 |
| 38 | MOXI | CT | 48 | 48 | 39.91±4.11/40.03±3.87 | NR | 33.44±3.42/33.71±3.46 | 35.71±3.75/35.26±3.68 |
| 39 | MOXI | CT | 30 | 30 | NR | NR | NR | NR |
| 40 | MOXI+IHM | CT | 50 | 50 | 41.43±4.59/41.45±4.50 | 43.48±5.43/43.53±5.44 | NR | 37.69±4.59/37.59±4.65 |

Table of P-values for Global Inconsistency Tests of Each Outcome in the Main Analysis of DPN Patients(Table S6)

| **Outcome** | **P value (Global Inconsistency Test)** |
| --- | --- |
| Primary analysis:common peroneal MNCV | 0.3547 |
| Primary analysis:median MNCV | 0.066 |
| Primary analysis:common peroneal SNCV | 0.3267 |
| Primary analysis:median SNCV | 0.0561 |
| Primary analysis:TCSS | 0.9394 |
| Primary analysis:markedly effective rate | 0.2548 |

Node-splitting analysis in the primary analysis

Node-splitting analysis based on the common peroneal MNCV in the primary analysis of patients with DPN (Table S7)

| **Side** | **Coef.** | **Std. Err.** | **Coef.** | **Std. Err.** | **Coef.** | **Std. Err.** | **P>z** | **tau** |
| --- | --- | --- | --- | --- | --- | --- | --- | --- |
| ACU VS MOXI | 5.239013 | 1.794622 | 0.8455352 | 20.11422 | 4.393478 | 20.19409 | 0.828 | 2.348179 |
| CT VS MOXI | 3.495843 | 0.9146913 | 0.3122365 | 2.271074 | 3.183606 | 2.447879 | 0.193 | 2.280526 |
| CT VS MOXI+ACU | 5.387423 | 1.423796 | -4.336197 | 91.76648 | 9.72362 | 91.77725 | 0.916 | 2.347686 |
| CT VS MOXI+EHM | 5.063826 | 1.41287 | 8.31381 | 2.00785 | -3.249984 | 2.455076 | 0.186 | 2.280407 |
| CT VS MOXI+IHM | 3.948016 | 0.9988435 | -2.903279 | 71.21584 | 6.851294 | 71.22282 | 0.923 | 2.347667 |
| CT VS MOXI+MT | 3.685881 | 1.76979 | -4.308916 | 157.6726 | 7.994797 | 157.6825 | 0.96 | 2.347547 |
| EHM VS MOXI | -0.1625619 | 2.884619 | 0.5266377 | 2.791818 | -0.6891997 | 4.026193 | 0.864 | 2.40601 |
| EHM VS MOXI+EHM | 3.163882 | 1.902634 | 4.254352 | 5.871478 | -1.09047 | 6.231528 | 0.861 | 2.401277 |
| IHM VS MOXI+IHM | 4.71 | 2.513788 | 12.19912 | 253.7287 | -7.489115 | 253.7416 | 0.976 | 2.347517 |
| MOXI VS MOXI+EHM | 4.696274 | 1.879976 | 1.846587 | 1.649117 | 2.849687 | 2.505165 | 0.255 | 2.307118 |

Node-splitting analysis based on the median MNCV in the primary analysis of patients with DPN (Table S8)

| **Side** | **Coef.** | **Std. Err.** | **Coef.** | **Std. Err.** | **Coef.** | **Std. Err.** | **P>z** | **tau** |
| --- | --- | --- | --- | --- | --- | --- | --- | --- |
| ACU VS MOXI | 5.69 | 2.798463 | 0.4682819 | 32.37625 | 5.221718 | 32.49697 | 0.872 | 2.549043 |
| CT VS MOXI | 3.795819 | 1.400851 | -2.935743 | 3.427323 | 6.731562 | 3.702724 | 0.069 | 2.265828 |
| CT VS MOXI+ACU | 6.47 | 2.653112 | -5.654842 | 256.1951 | 12.12484 | 256.2088 | 0.962 | 2.548017 |
| CT VS MOXI+EHM | 3.369992 | 2.462474 | 10.19615 | 2.782558 | -6.826159 | 3.715698 | 0.066 | 2.266519 |
| CT VS MOXI+IHM | 4.094249 | 1.009015 | -5.691302 | 96.02073 | 9.78555 | 96.02601 | 0.919 | 2.548487 |
| CT VS MOXI+MT | 4.036878 | 1.926284 | -5.657488 | 183.7848 | 9.694366 | 183.7948 | 0.958 | 2.548089 |
| EHM VS MOXI+EHM | 4.75 | 2.665199 | 18.36022 | 286.1791 | -13.61022 | 286.1916 | 0.962 | 2.547996 |
| MOXI VS MOXI+EHM | 6.400013 | 2.404986 | -0.4348288 | 2.831767 | 6.834841 | 3.715218 | 0.066 | 2.266484 |

Node-splitting analysis based on the common peroneal SNCV in the primary analysis of patients with DPN (Table S9)

| **Side** | **Coef.** | **Std. Err.** | **Coef.** | **Std. Err.** | **Coef.** | **Std. Err.** | **P>z** | **tau** |
| --- | --- | --- | --- | --- | --- | --- | --- | --- |
| ACU VS MOXI+ACU | 2.33 | 2.494023 | 3.474106 | 24.2752 | -1.144105 | 24.40298 | 0.963 | 2.352216 |
| CT VS MOXI | 3.12381 | 1.016556 | -0.5111196 | 3.580688 | 3.634929 | 3.722158 | 0.329 | 2.356108 |
| CT VS MOXI+ACU | 6.01 | 2.422317 | 4.833651 | 24.84407 | 1.176349 | 24.96193 | 0.962 | 2.35222 |
| CT VS MOXI+EHM | 6.679999 | 2.590927 | 10.32788 | 2.679752 | -3.647877 | 3.727463 | 0.328 | 2.35639 |
| CT VS MOXI+IHM | 4.578065 | 1.212294 | 6.657646 | 76.52623 | -2.079581 | 76.5358 | 0.978 | 2.350399 |
| CT VS MOXI+MT | 3.97 | 2.585935 | 7.316215 | 268.9407 | -3.346215 | 268.9532 | 0.99 | 2.349774 |
| EHM VS MOXI+EHM | 3.877487 | 1.743746 | 9.585793 | 154.8144 | -5.708306 | 154.8243 | 0.971 | 2.350021 |
| IHM VS MOXI+IHM | 3.67 | 2.513766 | 1.841679 | 251.68 | 1.828321 | 251.692 | 0.994 | 2.349805 |
| MOXI VS MOXI+EHM | 7.210003 | 2.48157 | 3.553871 | 2.781744 | 3.656132 | 3.727771 | 0.327 | 2.356434 |

Node-splitting analysis based on the median SNCV in the primary analysis of patients with DPN (Table S10)

| **Side** | **Coef.** | **Std. Err.** | **Coef.** | **Std. Err.** | **Coef.** | **Std. Err.** | **P>z** | **tau** |
| --- | --- | --- | --- | --- | --- | --- | --- | --- |
| ACU VS MOXI | 6.66 | 2.878729 | 1.15642 | 26.84268 | 5.50358 | 26.99661 | 0.838 | 2.659825 |
| CT VS MOXI | 4.186079 | 1.263742 | -2.176406 | 3.13382 | 6.362485 | 3.378961 | 0.06 | 2.417087 |
| CT VS MOXI+ACU | 6.610001 | 2.712599 | -6.61887 | 185.1254 | 13.22887 | 185.1449 | 0.943 | 2.658751 |
| CT VS MOXI+EHM | 2.395341 | 1.855576 | 8.859121 | 2.838194 | -6.46378 | 3.390942 | 0.057 | 2.417539 |
| CT VS MOXI+IHM | 4.044489 | 0.9817038 | -6.661604 | 83.84622 | 10.70609 | 83.85196 | 0.898 | 2.659133 |
| EHM VS MOXI+EHM | 4.676252 | 1.952213 | 15.26448 | 154.255 | -10.58823 | 154.2675 | 0.945 | 2.658794 |
| MOXI VS MOXI+EHM | 4.680012 | 2.543258 | -1.801496 | 2.243452 | 6.481507 | 3.391346 | 0.056 | 2.417549 |

Node-splitting analysis based on the TCSS in the primary analysis of patients with DPN (Table S11)

| **Side** | **Coef.** | **Std. Err.** | **Coef.** | **Std. Err.** | **Coef.** | **Std. Err.** | **P>z** | **tau** |
| --- | --- | --- | --- | --- | --- | --- | --- | --- |
| ACU VS MOXI | -1.597103 | 0.7761585 | -0.7099399 | 4.060507 | -0.8871635 | 4.134018 | 0.83 | 0.9721641 |
| CT VS MOXI | -1.428213 | 0.6264928 | -1.619487 | 1.491886 | 0.1912741 | 1.617394 | 0.906 | 1.016998 |
| CT VS MOXI+EHM | -2.583947 | 0.7511488 | -2.457435 | 1.461872 | -0.1265124 | 1.64311 | 0.939 | 1.018322 |
| CT VS MOXI+IHM | -1.576956 | 0.473901 | 0.2178882 | 56.23788 | -1.794844 | 56.23981 | 0.975 | 0.9678516 |
| EHM VS MOXI | 0.06 | 1.396428 | 0.3083962 | 2.9392 | -0.2483963 | 3.286074 | 0.94 | 1.018308 |
| EHM VS MOXI+EHM | -0.96 | 1.281252 | -1.209765 | 3.092749 | 0.2497645 | 3.28622 | 0.939 | 1.018316 |
| MOXI VS MOXI+EHM | -1.019961 | 1.321474 | -1.144512 | 0.9765386 | 0.1245507 | 1.643149 | 0.94 | 1.018316 |

Node-splitting analysis based on the markedly effective rate in the primary analysis of patients with DPN(Table S12)

| **Side** | **Coef.** | **Std. Err.** | **Coef.** | **Std. Err.** | **Coef.** | **Std. Err.** | **P>z** | **tau** |
| --- | --- | --- | --- | --- | --- | --- | --- | --- |
| ACU VS MOXI | 0.2876821 | 0.2116264 | 0.8215127 | 0.4408161 | -0.5338306 | 0.4889832 | 0.275 | 0.1011577 |
| ACU VS MOXI+ACU | 0.8109302 | 0.3553447 | 0.2738194 | 0.3369553 | 0.5371108 | 0.4897027 | 0.273 | 0.1011456 |
| CT VS MOXI | 0.3905004 | 0.099844 | -0.0623484 | 0.2535501 | 0.4528488 | 0.2732612 | 0.097 | 0.0650087 |
| CT VS MOXI+ACU | 0.335406 | 0.241533 | 0.8724992 | 0.4257069 | -0.5370931 | 0.4897139 | 0.273 | 0.1011464 |
| CT VS MOXI+EHM | 0.4268458 | 0.1970746 | 0.7864355 | 0.2654718 | -0.3595897 | 0.3320772 | 0.279 | 0.0903474 |
| CT VS MOXI+IHM | 0.2610759 | 0.0826962 | -0.1208484 | 19.56999 | 0.3819243 | 19.57027 | 0.984 | 0.1095619 |
| EHM VS MOXI+EHM | 0.3087428 | 0.1282347 | 1.262803 | 29.53176 | -0.9540602 | 29.5319 | 0.974 | 0.1095621 |
| MOXI VS MOXI+EHM | 0.4248788 | 0.2449332 | 0.0649698 | 0.2242222 | 0.3599091 | 0.3320668 | 0.278 | 0.090343 |

Loop-Specific Inconsistency Test for the Primary Analysis

Loop inconsistency analysis of the common peroneal MNCV based on the primary analysis in patients with DPN(Table S13)

| **Loop** | **IF** | **seIF** | **z_value** | **p_value** | **CI_95** | **Loop_Heterog_tau2** |
| --- | --- | --- | --- | --- | --- | --- |
| CT-MOXI-MOXI+EHM | 3.193 | 2.332 | 1.369 | 0.171 | (0.00,7.76) | 4.106 |
| EHM-MOXI-MOXI+EHM | 1.636 | 1.844 | 0.887 | 0.375 | (0.00,5.25) | 0 |

Loop inconsistency analysis of the median MNCV based on the primary analysis in patients with DPN(Table S14)

| **Loop** | **IF** | **seIF** | **z_value** | **p_value** | **CI_95** | **Loop_Heterog_tau2** |
| --- | --- | --- | --- | --- | --- | --- |
| CT-MOXI-MOXI+EHM | 6.822 | 4.002 | 1.705 | 0.088 | (0.00,14.66) | 6.015 |

Loop inconsistency analysis of the common peroneal SNCV based on the primary analysis in patients with DPN(Table S15)

| **Loop** | **IF** | **seIF** | **z_value** | **p_value** | **CI_95** | **Loop_Heterog_tau2** |
| --- | --- | --- | --- | --- | --- | --- |
| CT-MOXI-MOXI+EHM | 3.447 | 1.656 | 2.082 | 0.037 | (0.00,6.69) | 0.409 |

Loop inconsistency analysis of the median SNCV based on the primary analysis in patients with DPN(Table S16)

| **Loop** | **IF** | **seIF** | **z_value** | **p_value** | **CI_95** | **Loop_Heterog_tau2** |
| --- | --- | --- | --- | --- | --- | --- |
| CT-MOXI-MOXI+EHM | 6.47 | 3.256 | 1.987 | 0.047 | (0.00,12.85) | 5.326 |

Loop inconsistency analysis of the TCSS based on the primary analysis in patients with DPN(Table S17)

| **Loop** | **IF** | **seIF** | **z_value** | **p_value** | **CI_95** | **Loop_Heterog_tau2** |
| --- | --- | --- | --- | --- | --- | --- |
| CT-MOXI-MOXI+ACU | 0.131 | 2.088 | 0.063 | 0.95 | (0.00,4.22) | 1.94 |

Loop inconsistency analysis of the markedly effective rate based on the primary analysis in patients with DPN(Table S18)

| **Loop** | **IF** | **seIF** | **z_value** | **p_value** | **CI_95** | **Loop_Heterog_tau2** |
| --- | --- | --- | --- | --- | --- | --- |
| ACU-CT-MOXI-MOXI+ACU | 0.594 | 0.46 | 1.291 | 0.197 | (0.00,1.49) | 0 |
| CT-MOXI-MOXI+EHM | 0.41 | 0.336 | 1.22 | 0.222 | (0.00,1.07) | 0 |

SUCRA Primary analysis

Ranking of treatment effects based on the common peroneal MNCV in the primary analysis of patients with DPN (Table S19)

| **Treatment** | **SUCRA** | **PrBest** | **MeanRank** |
| --- | --- | --- | --- |
| MOXI+EHM | 92.3 | 55.7 | 1.6 |
| MOXI+ACU | 83.5 | 30.9 | 2.3 |
| MOXI+IHM | 65.3 | 2.9 | 3.8 |
| MOXI+MT | 62.2 | 8.4 | 4 |
| MOXI | 52.2 | 0 | 4.8 |
| EHM | 50.5 | 1.8 | 5 |
| CT | 19.8 | 0 | 7.4 |
| IHM | 17.5 | 0.4 | 7.6 |
| ACU | 6.7 | 0 | 8.5 |

Ranking of treatment effects based on the median MNCV in the primary analysis of patients with DPN (Table S20)

| **Treatment** | **SUCRA** | **PrBest** | **MeanRank** |
| --- | --- | --- | --- |
| MOXI+EHM | 86.5 | 40.9 | 1.9 |
| MOXI+ACU | 83 | 45.4 | 2.2 |
| MOXI+IHM | 62.8 | 2.8 | 3.6 |
| MOXI+MT | 60.9 | 8.5 | 3.7 |
| MOXI | 47.1 | 0.3 | 4.7 |
| EHM | 36.6 | 2.1 | 5.4 |
| CT | 16.9 | 0 | 6.8 |
| ACU | 6.2 | 0.1 | 7.6 |

Ranking of treatment effects based on the common peroneal SNCV in the primary analysis of patients with DPN (Table S21)

| **Treatment** | **SUCRA** | **PrBest** | **MeanRank** |
| --- | --- | --- | --- |
| MOXI+EHM | 94.1 | 69.4 | 1.5 |
| MOXI+ACU | 74.2 | 16.2 | 3.1 |
| MOXI+IHM | 60.4 | 1.6 | 4.2 |
| EHM | 56.7 | 0.9 | 4.5 |
| MOXI+MT | 51.2 | 5.4 | 4.9 |
| ACU | 47.5 | 5.9 | 5.2 |
| MOXI | 37.2 | 0 | 6 |
| IHM | 20.9 | 0.6 | 7.3 |
| CT | 7.8 | 0 | 8.4 |

Ranking of treatment effects based on the median SNCV in the primary analysis of patients with DPN (Table S22)

| **Treatment** | **SUCRA** | **PrBest** | **MeanRank** |
| --- | --- | --- | --- |
| MOXI+ACU | 89.9 | 70.1 | 1.6 |
| MOXI+EHM | 75 | 17.4 | 2.5 |
| MOXI+IHM | 70.7 | 8.9 | 2.8 |
| MOXI | 60.4 | 3.4 | 3.4 |
| CT | 23.9 | 0 | 5.6 |
| EHM | 23.6 | 0.2 | 5.6 |
| ACU | 6.5 | 0.1 | 6.6 |

Ranking of treatment effects based on the TCSS in the primary analysis of patients with DPN (Table S23)

| **Treatment** | **SUCRA** | **PrBest** | **MeanRank** |
| --- | --- | --- | --- |
| MOXI+EHM | 89.8 | 57.3 | 1.6 |
| MOXI+IHM | 61.7 | 5.6 | 3.3 |
| EHM | 59.8 | 16.7 | 3.4 |
| MOXI | 57.6 | 1.5 | 3.5 |
| MOXI+ACU | 54.2 | 18.8 | 3.7 |
| CT | 14.3 | 0 | 6.1 |
| ACU | 12.5 | 0 | 6.3 |

Ranking of treatment effects based on the markedly effective rate in the primary analysis of patients with DPN (Table S24)

| **Treatment** | **SUCRA** | **PrBest** | **MeanRank** |
| --- | --- | --- | --- |
| MOXI+EHM | 91.3 | 60.1 | 1.5 |
| MOXI+ACU | 77.2 | 35.1 | 2.4 |
| MOXI | 61.1 | 2.8 | 3.3 |
| MOXI+IHM | 49.9 | 1.5 | 4 |
| EHM | 47.6 | 0.5 | 4.1 |
| CT | 12.5 | 0 | 6.3 |
| ACU | 10.5 | 0.1 | 6.4 |

Ranking of treatment effects based on the FPG in the primary analysis of patients with DPN (Table S25)

| **Treatment** | **SUCRA** | **PrBest** | **MeanRank** |
| --- | --- | --- | --- |
| MOXI+IHM | 99 | 96.6 | 1 |
| MOXI+EHM | 61.5 | 1.7 | 2.5 |
| MOXI | 49 | 0.5 | 3 |
| EHM | 28.2 | 1.2 | 3.9 |
| CT | 12.3 | 0 | 4.5 |

Ranking of treatment effects based on the 2hPG in the primary analysis of patients with DPN (Table S26)

| **Treatment** | **SUCRA** | **PrBest** | **MeanRank** |
| --- | --- | --- | --- |
| MOXI+MT | 91.7 | 72.9 | 1.4 |
| MOXI+IHM | 74.8 | 14.8 | 2.3 |
| MOXI+EHM | 64.9 | 10.7 | 2.8 |
| MOXI | 32.6 | 0.4 | 4.4 |
| EHM | 24.9 | 1.1 | 4.8 |
| CT | 11.2 | 0 | 5.4 |

Table of P values for the Global Inconsistency Test of Each Outcome in the Subgroup Analysis of Patients with DPN(Table S27)

| **Outcome** | **P value (Global Inconsistency Test)** |
| --- | --- |
| subgroup analysis:TCSS | 0.4515 |
| subgroup analysis:markedly effective rate | 0.4987 |

Node-splitting analysis in the subgroup analysis

Node-splitting analysis based on the TCSS in the subgroup analysis of patients with DPN (Table S28)

| **Side** | **Coef.** | **Std. Err.** | **Coef.** | **Std. Err.** | **Coef.** | **Std. Err.** | **P>z** | **tau** |
| --- | --- | --- | --- | --- | --- | --- | --- | --- |
| ACU VS MM | -1.44 | 1.284032 | -2.734612 | 1.275272 | 1.294612 | 1.809712 | 0.474 | 1.086131 |
| ACU VS WN | -1.597561 | 0.8460165 | -0.2822704 | 1.52709 | -1.31529 | 1.745779 | 0.451 | 1.082322 |
| CT VS HM | -1.709353 | 0.8189554 | -0.4809277 | 77.16329 | -1.228426 | 77.16791 | 0.987 | 1.055688 |
| CT VS MM | -2.589122 | 0.7972413 | -1.292634 | 1.62557 | -1.296488 | 1.810135 | 0.474 | 1.086162 |
| CT VS TF | -1.618061 | 0.7982422 | -0.4419184 | 8.495422 | -1.176142 | 8.532604 | 0.89 | 1.06246 |
| CT VS WN | -1.391211 | 0.5761488 | -2.707763 | 1.649688 | 1.316552 | 1.747358 | 0.451 | 1.082425 |

Node-splitting analysis based on the markedly effective rate in the subgroup analysis of patients with DPN (Table S29)

| **Side** | **Coef.** | **Std. Err.** | **Coef.** | **Std. Err.** | **Coef.** | **Std. Err.** | **P>z** | **tau** |
| --- | --- | --- | --- | --- | --- | --- | --- | --- |
| ACU VS MM | 0.8109302 | 0.3406456 | 0.5253405 | 0.2473102 | 0.2855897 | 0.4209534 | 0.497 | 1.88e-07 |
| ACU VS WN | 0.2876821 | 0.185884 | 0.5718316 | 0.3771813 | -0.2841496 | 0.420498 | 0.499 | 2.42e-07 |
| CT VS GIM | 0.1053605 | 0.1130388 | -0.0806945 | 35.55248 | 0.186055 | 35.55274 | 0.996 | 1.09e-07 |
| CT VS HM | 0.5503777 | 0.2939131 | -0.102062 | 66.59289 | 0.6524397 | 66.59403 | 0.992 | 9.53e-07 |
| CT VS MM | 0.5532805 | 0.1403084 | 0.8387977 | 0.3968878 | -0.2855172 | 0.4209602 | 0.498 | 5.02e-07 |
| CT VS TF | 0.2937164 | 0.1130392 | -0.0989713 | 38.50023 | 0.3926877 | 38.5005 | 0.992 | 5.78e-09 |
| CT VS WDM | 0.3123087 | 0.1410483 | 0.0853104 | 13.99385 | 0.2269983 | 13.99461 | 0.987 | 1.70e-07 |
| CT VS WN | 0.3154903 | 0.0834495 | 0.0310343 | 0.4122837 | 0.284456 | 0.4206432 | 0.499 | 4.94e-09 |
| EHM VS WDM | 0.3114213 | 0.0926168 | 0.726332 | 29.53456 | -0.4149106 | 29.53456 | 0.989 | 3.42e-07 |

Loop-Specific Inconsistency Analysis in the Subgroup Analysis

Loop inconsistency analysis based on TCSS in the subgroup analysis of patients with DPN (Table S30)

| **Loop** | **IF** | **seIF** | **z_value** | **p_value** | **CI_95** | **Loop_Heterog_tau2** |
| --- | --- | --- | --- | --- | --- | --- |
| ACU-CT-MM-WN | 1.359 | 1.948 | 0.698 | 0.485 | (0.00,5.18) | 1.383 |

Loop inconsistency analysis based on markedly effective rate in the subgroup analysis of patients with DPN (Table S31)

| **Loop** | **IF** | **seIF** | **z_value** | **p_value** | **CI_95** | **Loop_Heterog_tau2** |
| --- | --- | --- | --- | --- | --- | --- |
| ACU-CT-MM-WN | 0.23 | 0.56 | 0.411 | 0.681 | (0.00,1.33) | 0.039 |

SUCRA Subgroup analysis

Ranking of treatment effects based on the TCSS in the subgroup analysis of patients with DPN(Table S32)

| **Treatment** | **SUCRA** | **PrBest** | **MeanRank** |
| --- | --- | --- | --- |
| MM | 86.3 | 56 | 1.7 |
| HM | 65.2 | 20.3 | 2.7 |
| TF | 61.9 | 16.6 | 2.9 |
| WN | 60.8 | 7 | 3 |
| ACU | 17.5 | 0.1 | 5.1 |
| CT | 8.3 | 0 | 5.6 |

Ranking of treatment effects based on the markedly effective rate in the subgroup analysis of patients with DPN(Table S33)

| **Treatment** | **SUCRA** | **PrBest** | **MeanRank** |
| --- | --- | --- | --- |
| MM | 92.1 | 49.5 | 1.6 |
| HM | 82.7 | 45.2 | 2.4 |
| WDM | 64.7 | 3.3 | 3.8 |
| WN | 63.6 | 0.5 | 3.9 |
| TF | 61.8 | 1.4 | 4.1 |
| GIM | 33.6 | 0 | 6.3 |
| EHM | 19.6 | 0 | 7.4 |
| CT | 16.4 | 0 | 7.7 |
| ACU | 15.6 | 0 | 7.8 |

Ranking of treatment effects based on the common peroneal MNCV in the subgroup analysis of patients with DPN(Table S34)

| **Treatment** | **SUCRA** | **PrBest** | **MeanRank** |
| --- | --- | --- | --- |
| TF | 92 | 56.7 | 1.7 |
| MM | 89.7 | 34.7 | 1.9 |
| WDM | 70.2 | 4.6 | 3.7 |
| GIM | 59.8 | 1.8 | 4.6 |
| WN | 58.1 | 0.2 | 4.8 |
| HM | 45.3 | 0.6 | 5.9 |
| IHM | 34.6 | 1 | 6.9 |
| EHM | 23.5 | 0.4 | 7.9 |
| CT | 19.5 | 0 | 8.2 |
| ACU | 7.2 | 0 | 9.3 |

Ranking of treatment effects based on the median MNCV in the subgroup analysis of patients with DPN(Table S35)

| **Treatment** | **SUCRA** | **PrBest** | **MeanRank** |
| --- | --- | --- | --- |
| GIM | 74 | 25.3 | 3.1 |
| MM | 72.2 | 22 | 3.2 |
| TF | 71.1 | 15.9 | 3.3 |
| WDM | 70.8 | 18.5 | 3.3 |
| HM | 56.8 | 14 | 4.5 |
| WNT | 56.1 | 3 | 4.5 |
| EHM | 21.5 | 1.1 | 7.3 |
| CT | 18.3 | 0 | 7.5 |
| ACU | 9.3 | 0.1 | 8.3 |

Ranking of treatment effects based on the common peroneal SNCV in the subgroup analysis of patients with DPN(Table S36)

| **Treatment** | **SUCRA** | **PrBest** | **MeanRank** |
| --- | --- | --- | --- |
| TF | 80.4 | 41 | 2.8 |
| MM | 79.1 | 19.6 | 2.9 |
| WN | 64.3 | 4.1 | 4.2 |
| GIM | 60.9 | 14.8 | 4.5 |
| WDM | 57.8 | 5.4 | 4.8 |
| ACU | 50.7 | 9.1 | 5.4 |
| IHM | 37.1 | 3.3 | 6.7 |
| HM | 35.9 | 2.4 | 6.8 |
| EHM | 17.6 | 0.2 | 8.4 |
| CT | 16.1 | 0 | 8.6 |

Ranking of treatment effects based on the median SNCV in the subgroup analysis of patients with DPN(Table S37)

| **Treatment** | **SUCRA** | **PrBest** | **MeanRank** |
| --- | --- | --- | --- |
| MM | 90.7 | 55.2 | 1.7 |
| GIM | 76 | 26.8 | 2.9 |
| TF | 74.1 | 10.8 | 3.1 |
| WN | 64.2 | 1.9 | 3.9 |
| WDM | 51.1 | 4.1 | 4.9 |
| HM | 47.4 | 1.1 | 5.2 |
| CT | 24.3 | 0 | 7.1 |
| EHM | 12.5 | 0.1 | 8 |
| ACU | 9.8 | 0 | 8.2 |

Ranking of treatment effects based on the FPG in the subgroup analysis of patients with DPN(Table S38)

| **Treatment** | **SUCRA** | **PrBest** | **MeanRank** |
| --- | --- | --- | --- |
| GIM | 87.9 | 60.5 | 1.8 |
| MM | 72.3 | 15.6 | 2.9 |
| WN | 54.7 | 3.9 | 4.2 |
| TF | 52.4 | 3.5 | 4.3 |
| WDM | 51.4 | 8.3 | 4.4 |
| EHM | 36.2 | 6.5 | 5.5 |
| HM | 31 | 1.7 | 5.8 |
| CT | 14.2 | 0 | 7 |

Ranking of treatment effects based on the 2hPG in the subgroup analysis of patients with DPN(Table S39)

| **Treatment** | **SUCRA** | **PrBest** | **MeanRank** |
| --- | --- | --- | --- |
| MM | 88.1 | 45 | 1.8 |
| GIM | 88 | 36.9 | 1.8 |
| WDM | 62.3 | 17.6 | 3.6 |
| WN | 54.3 | 0 | 4.2 |
| TF | 52.3 | 0.5 | 4.3 |
| EHM | 25.9 | 0 | 6.2 |
| HM | 21.8 | 0 | 6.5 |
| CT | 7.4 | 0 | 7.5 |

Meta-regression in the primary analysis

Meta-regression analysis of the common peroneal MNCV in the primary analysis of DPN: intervention duration(Table S40)

|  |  | **Coefficient** | **Std. err.** | **z** | **P>z** | **[95% conf.interval]** | |
| --- | --- | --- | --- | --- | --- | --- | --- |
| ACU VS CT | cov1 | -0.8025498 | 0.3344886 | -2.14 | 0.057 | -1.458135 | -0.1469643 |
| ACU VS MOXI | cov1 | -0.6421293 | 0.3151651 | -2.04 | 0.06 | -1.259842 | -0.0244171 |
| ACU VS MOXI+ACU | cov1 | -0.7085515 | 0.3777653 | -1.88 | 0.061 | -1.448958 | 0.0318549 |
| ACU VS MOXI+EHM | cov1 | -0.7669101 | 0.3331083 | -2.23 | 0.054 | -1.41979 | -0.1140298 |
| ACU VS MOXI+IHM | cov1 | -0.872358 | 0.3354914 | -2.16 | 0.056 | -1.529909 | -0.214807 |
| ACU VS MOXI+MT | cov1 | -0.8677015 | 0.3442741 | -2.22 | 0.053 | -1.542466 | -0.1929367 |

Meta-regression analysis of the common peroneal MNCV in the primary analysis of DPN: age(Table S41)

|  |  | **Coefficient** | **Std. err.** | **z** | **P>z** | **[95% conf.interval]** | |
| --- | --- | --- | --- | --- | --- | --- | --- |
| ACU VS CT | age | 0.6824339 | 0.2973886 | 2.09 | 0.059 | 0.099563 | 1.265305 |
| ACU VS EHM | age | 0.8401297 | 0.5244316 | 1.6 | 0.109 | -0.1877374 | 1.867997 |
| ACU VS MOXI | age | 0.4701267 | 0.2497857 | 1.88 | 0.06 | -0.0194442 | 0.9596976 |
| ACU VS MOXI+ACU | age | 0.3684478 | 0.4520814 | 0.82 | 0.415 | -0.5176155 | 1.254511 |
| ACU VS MOXI+EHM | age | 0.6572562 | 0.3599174 | 1.83 | 0.068 | -0.0481689 | 1.362681 |
| ACU VS MOXI+IHM | age | 0.4698128 | 0.3580226 | 1.31 | 0.189 | -0.2318986 | 1.171524 |
| ACU VS MOXI+MT | age | -0.100977 | 1.192317 | -0.08 | 0.933 | -2.437876 | 2.235922 |

Meta-regression analysis of the common peroneal MNCV in the primary analysis of DPN: disease duration (Table S42)

|  |  | **Coefficient** | **Std. err.** | **z** | **P>z** | **[95% conf.interval]** | |
| --- | --- | --- | --- | --- | --- | --- | --- |
| ACU VS CT | time | 0.8033461 | 12.55414 | 0.06 | 0.949 | -23.80232 | 25.40902 |
| ACU VS MOXI | time | 0.5986049 | 12.55761 | 0.05 | 0.962 | -24.01386 | 25.21107 |
| ACU VS MOXI+EHM | time | 1.47632 | 12.56445 | 0.12 | 0.906 | -23.14954 | 26.10218 |
| ACU VS MOXI+IHM | time | 0.1744208 | 12.56084 | 0.01 | 0.989 | -24.44437 | 24.79321 |
| ACU VS MOXI+MT | time | 1.977786 | 12.64462 | 0.16 | 0.876 | -22.80522 | 26.76079 |

Meta-regression analysis of the median MNCV in the primary analysis of DPN: intervention duration(Table S43)

|  |  | **Coefficient** | **Std. err.** | **z** | **P>z** | **[95% conf.interval]** | |
| --- | --- | --- | --- | --- | --- | --- | --- |
| ACU VS CT | cov1 | -0.1952604 | 1.370857 | -0.14 | 0.887 | -2.88209 | 2.49157 |
| ACU VS MOXI | cov1 | 0.2944322 | 1.377846 | 0.21 | 0.831 | -2.406097 | 2.994961 |
| ACU VS MOXI+IHM | cov1 | -0.2878988 | 1.370998 | -0.21 | 0.834 | -2.975006 | 2.399209 |
| ACU VS MOXI+MT | cov1 | -0.1785788 | 1.372296 | -0.13 | 0.896 | -2.868229 | 2.511071 |

Meta-regression analysis of the median MNCV in the primary analysis of DPN: age(Table S44)

|  |  | **Coefficient** | **Std. err.** | **z** | **P>z** | **[95% conf.interval]** | |
| --- | --- | --- | --- | --- | --- | --- | --- |
| ACU VS CT | age | 0.3919111 | 2.698171 | 0.15 | 0.885 | -4.896406 | 5.680229 |
| ACU VS MOXI | age | 0.286081 | 2.693159 | 0.11 | 0.915 | -4.992414 | 5.564576 |
| ACU VS MOXI+EHM | age | 2.582188 | 3.044835 | 0.85 | 0.396 | -3.385579 | 8.549955 |
| ACU VS MOXI+IHM | age | 0.404043 | 2.715826 | 0.15 | 0.882 | -4.918877 | 5.726963 |
| ACU VS MOXI+MT | age | 0.5917804 | 3.030981 | 0.2 | 0.845 | -5.348834 | 6.532395 |

Meta-regression analysis of the median MNCV in the primary analysis of DPN: disease duration (Table S45)

|  |  | **Coefficient** | **Std. err.** | **z** | **P>z** | **[95% conf.interval]** | |
| --- | --- | --- | --- | --- | --- | --- | --- |
| ACU VS CT | time | 0.3004217 | 15.39867 | 0.02 | 0.984 | -29.88041 | 30.48126 |
| ACU VS MOXI | time | -0.451707 | 15.40277 | -0.03 | 0.977 | -30.64058 | 29.73717 |
| ACU VS MOXI+EHM | time | 4.062474 | 15.48009 | 0.26 | 0.793 | -26.27794 | 34.40288 |
| ACU VS MOXI+IHM | time | -0.7566791 | 15.40302 | -0.05 | 0.961 | -30.94604 | 29.43268 |
| ACU VS MOXI+MT | time | 0.000381 | 15.46222 | 0 | 1 | -30.30501 | 30.30577 |

Meta-regression analysis of the common peroneal SNCV in the primary analysis of DPN: intervention duration(Table S46)

|  |  | **Coefficient** | **Std. err.** | **z** | **P>z** | **[95% conf.interval]** | |
| --- | --- | --- | --- | --- | --- | --- | --- |
| ACU VS CT | cov1 | 0.0623073 | 1.382575 | 0.05 | 0.964 | -2.64749 | 2.772105 |
| ACU VS MOXI | cov1 | 0.0617551 | 1.389681 | 0.04 | 0.965 | -2.66197 | 2.785481 |
| ACU VS MOXI+ACU | cov1 | 0.0740764 | 1.369325 | 0.05 | 0.957 | -2.609752 | 2.757904 |
| ACU VS MOXI+EHM | cov1 | -0.0243771 | 1.384085 | -0.02 | 0.986 | -2.737135 | 2.68838 |
| ACU VS MOXI+IHM | cov1 | -0.0226555 | 1.382737 | -0.02 | 0.987 | -2.73277 | 2.687459 |

Meta-regression analysis of the common peroneal SNCV in the primary analysis of DPN: age(Table S47)

|  |  | **Coefficient** | **Std. err.** | **z** | **P>z** | **[95% conf.interval]** | |
| --- | --- | --- | --- | --- | --- | --- | --- |
| ACU VS CT | age | 0.001386 | 4.056942 | 0 | 1 | -7.950075 | 7.952847 |
| ACU VS EHM | age | 2.045686 | 5.011524 | 0.41 | 0.683 | -7.77672 | 11.86809 |
| ACU VS MOXI | age | -0.0234453 | 4.058136 | -0.01 | 0.995 | -7.977245 | 7.930354 |
| ACU VS MOXI+ACU | age | 0.5943439 | 9.715289 | 0.06 | 0.951 | -18.44727 | 19.63596 |
| ACU VS MOXI+EHM | age | 1.796647 | 4.563162 | 0.39 | 0.694 | -7.146987 | 10.74028 |
| ACU VS MOXI+IHM | age | 0.2176121 | 4.082712 | 0.05 | 0.957 | -7.784356 | 8.21958 |

Meta-regression analysis of the common peroneal SNCV in the primary analysis of DPN: disease duration (Table S48)

|  |  | **Coefficient** | **Std. err.** | **z** | **P>z** | **[95% conf.interval]** | |
| --- | --- | --- | --- | --- | --- | --- | --- |
| ACU VS IHM | time | -0.3052818 | 1.150685 | -0.27 | 0.791 | -2.560584 | 1.95002 |
| ACU VS MOXI+ACU | time | 4.105874 | 38.72198 | 0.11 | 0.916 | -71.78782 | 79.99957 |
| ACU VS MOXI+EHM | time | -0.2863167 | 0.6679024 | -0.43 | 0.668 | -1.595381 | 1.022748 |

Meta-regression analysis of the median SNCV in the primary analysis of DPN: intervention duration(Table S49)

|  |  | **Coefficient** | **Std. err.** | **z** | **P>z** | **[95% conf.interval]** | |
| --- | --- | --- | --- | --- | --- | --- | --- |
| ACU VS CT | cov1 | -0.1461577 | 1.320206 | -0.11 | 0.912 | -2.733715 | 2.441399 |
| ACU VS MOXI | cov1 | 0.1229831 | 1.327509 | 0.09 | 0.926 | -2.478887 | 2.724853 |
| ACU VS MOXI+EHM | cov1 | -0.2339395 | 1.34188 | -0.17 | 0.862 | -2.863976 | 2.396097 |
| ACU VS MOXI+IHM | cov1 | -0.2241901 | 1.320476 | -0.17 | 0.865 | -2.812276 | 2.363896 |

Meta-regression analysis of the median SNCV in the primary analysis of DPN: age(Table S50)

|  |  | **Coefficient** | **Std. err.** | **z** | **P>z** | **[95% conf.interval]** | |
| --- | --- | --- | --- | --- | --- | --- | --- |
| ACU VS CT | age | 0.7305733 | 2.145602 | 0.34 | 0.733 | -3.474729 | 4.935876 |
| ACU VS EHM | age | 2.302196 | 3.077137 | 0.75 | 0.454 | -3.72888 | 8.333273 |
| ACU VS MOXI | age | 0.2930262 | 2.131696 | 0.14 | 0.891 | -3.885022 | 4.471074 |
| ACU VS MOXI+EHM | age | 1.94311 | 2.455357 | 0.79 | 0.429 | -2.869302 | 6.755522 |
| ACU VS MOXI+IHM | age | 0.5659872 | 2.163888 | 0.26 | 0.794 | -3.675155 | 4.80713 |

Meta-regression analysis of the median SNCV in the primary analysis of DPN: disease duration (Table S51)

|  |  | **Coefficient** | **Std. err.** | **z** | **P>z** | **[95% conf.interval]** | |
| --- | --- | --- | --- | --- | --- | --- | --- |
| ACU VS EHM | time | 0.2020331 | 0.5200811 | 0.39 | 0.698 | -0.8173072 | 1.221373 |
| ACU VS MOXI+ACU | time | -0.0365348 | 0.6369074 | -0.06 | 0.954 | -1.28485 | 1.211781 |
| ACU VS MOXI+EHM | time | -0.6370827 | 0.4699732 | -1.36 | 0.175 | -1.558213 | 0.2840479 |

Meta-regression analysis of the TCSS in the primary analysis of DPN: intervention duration(Table S52)

|  |  | **Coefficient** | **Std. err.** | **z** | **P>z** | **[95% conf.interval]** | |
| --- | --- | --- | --- | --- | --- | --- | --- |
| ACU VS CT | cov1 | 0.0677472 | 0.177911 | 0.38 | 0.703 | -0.280952 | 0.4164464 |
| ACU VS MOXI | cov1 | -0.0113558 | 0.1587511 | -0.07 | 0.943 | -0.3225022 | 0.2997907 |
| ACU VS MOXI+EHM | cov1 | 0.0652993 | 0.1782619 | 0.37 | 0.714 | -0.2840877 | 0.4146863 |
| ACU VS MOXI+IHM | cov1 | 0.0877348 | 0.1785888 | 0.49 | 0.623 | -0.2622928 | 0.4377624 |

Meta-regression analysis of the TCSS in the primary analysis of DPN: age(Table S53)

|  |  | **Coefficient** | **Std. err.** | **z** | **P>z** | **[95% conf.interval]** | |
| --- | --- | --- | --- | --- | --- | --- | --- |
| ACU VS CT | age | -0.0757628 | 0.2040393 | -0.37 | 0.71 | -0.4756725 | 0.3241469 |
| ACU VS MOXI | age | -0.0122026 | 0.1373358 | -0.09 | 0.929 | -0.2813758 | 0.2569705 |
| ACU VS MOXI+EHM | age | -0.0038491 | 0.3508384 | -0.01 | 0.991 | -0.6914798 | 0.6837815 |
| ACU VS MOXI+IHM | age | -0.1085576 | 0.2512541 | -0.43 | 0.666 | -0.6010067 | 0.3838915 |

Meta-regression analysis of the TCSS in the primary analysis of DPN: disease duration (Table S54)

|  |  | **Coefficient** | **Std. err.** | **z** | **P>z** | **[95% conf.interval]** | |
| --- | --- | --- | --- | --- | --- | --- | --- |
| ACU VS CT | time | 0.14457 | 2.075459 | 0.07 | 0.944 | -3.923255 | 4.212395 |
| ACU VS EHM | time | 0.5912836 | 2.063993 | 0.29 | 0.775 | -3.454069 | 4.636636 |
| ACU VS MOXI | time | 0.3056596 | 2.081749 | 0.15 | 0.883 | -3.774493 | 4.385812 |

Meta-regression analysis of the markedly effective rate in the primary analysis of DPN: intervention duration(Table S55)

|  |  | **Coefficient** | **Std. err.** | **z** | **P>z** | **[95% conf.interval]** | |
| --- | --- | --- | --- | --- | --- | --- | --- |
| ACU VS CT | cov1 | 0.0019344 | 0.2750504 | 0.01 | 0.994 | -0.5371545 | 0.5410234 |
| ACU VS MOXI | cov1 | 0.0053736 | 0.2755801 | 0.02 | 0.984 | -0.5347535 | 0.5455006 |
| ACU VS MOXI+ACU | cov1 | -0.0878735 | 0.3610274 | -0.24 | 0.808 | -0.7954741 | 0.6197272 |
| ACU VS MOXI+EHM | cov1 | -0.0430257 | 0.2761667 | -0.16 | 0.876 | -0.5843024 | 0.498251 |
| ACU VS MOXI+IHM | cov1 | -0.000526 | 0.2750792 | 0 | 0.998 | -0.5396714 | 0.5386194 |

Meta-regression analysis of the markedly effective rate in the primary analysis of DPN: age(Table S56)

|  |  | **Coefficient** | **Std. err.** | **z** | **P>z** | **[95% conf.interval]** | |
| --- | --- | --- | --- | --- | --- | --- | --- |
| ACU VS CT | age | -0.0130793 | 0.0340334 | -0.38 | 0.701 | -0.0797836 | 0.053625 |
| ACU VS EHM | age | -0.1355294 | 0.2439093 | -0.56 | 0.578 | -0.6135829 | 0.3425241 |
| ACU VS MOXI | age | -0.0264208 | 0.0327136 | -0.81 | 0.419 | -0.0905382 | 0.0376967 |
| ACU VS MOXI+ACU | age | -0.0901274 | 0.048807 | -1.85 | 0.065 | -0.1857873 | 0.0055325 |
| ACU VS MOXI+EHM | age | 0.0377763 | 0.0402853 | 0.94 | 0.348 | -0.0411815 | 0.1167341 |
| ACU VS MOXI+IHM | age | -0.0051738 | 0.0390426 | -0.13 | 0.895 | -0.0816959 | 0.0713482 |

Meta-regression analysis of the markedly effective rate in the primary analysis of DPN: disease duration (Table S57)

|  |  | **Coefficient** | **Std. err.** | **z** | **P>z** | **[95% conf.interval]** | |
| --- | --- | --- | --- | --- | --- | --- | --- |
| ACU VS CT | time | -0.2453008 | 4.833963 | -0.05 | 0.96 | -9.719694 | 9.229092 |
| ACU VS MOXI | time | -0.2879109 | 4.834483 | -0.06 | 0.953 | -9.763323 | 9.187501 |
| ACU VS MOXI+EHM | time | 0.4687763 | 4.840523 | 0.1 | 0.923 | -9.018474 | 9.956027 |
| ACU VS MOXI+IHM | time | -0.2698601 | 4.835513 | -0.06 | 0.955 | -9.747292 | 9.207572 |

Meta-regression analysis of the FPG in the primary analysis of DPN: intervention duration(Table S58)

|  |  | **Coefficient** | **Std. err.** | **z** | **P>z** | **[95% conf.interval]** | |
| --- | --- | --- | --- | --- | --- | --- | --- |
| CT VS MOXI | cov1 | -0.0010748 | 0.0021813 | -0.49 | 0.622 | -0.0053501 | 0.0032005 |
| CT VS MOXI+EHM | cov1 | -0.0128834 | 0.0144272 | -0.89 | 0.372 | -0.0411601 | 0.0153933 |
| CT VS MOXI+IHM | cov1 | 0.0069102 | 0.0110202 | 0.63 | 0.531 | -0.0146889 | 0.0285093 |

Meta-regression analysis of the FPG in the primary analysis of DPN: age(Table S59)

|  |  | **Coefficient** | **Std. err.** | **z** | **P>z** | **[95% conf.interval]** | |
| --- | --- | --- | --- | --- | --- | --- | --- |
| CT VS MOXI | age | 0.0489207 | 0.1743711 | 0.28 | 0.779 | -0.2928403 | 0.3906817 |
| CT VS MOXI+EHM | age | -0.6420686 | 0.6641548 | -0.97 | 0.334 | -1.943788 | 0.6596508 |
| CT VS MOXI+IHM | age | -0.0089779 | 0.0751519 | -0.12 | 0.905 | -0.1562729 | 0.1383172 |

Meta-regression analysis of the FPG in the primary analysis of DPN: disease duration (Table S60)

|  |  | **Coefficient** | **Std. err.** | **z** | **P>z** | **[95% conf.interval]** | |
| --- | --- | --- | --- | --- | --- | --- | --- |
| CT VS MOXI | time | 0.084 | 0.1123186 | 0.75 | 0.455 | -0.1361404 | 0.3041403 |
| CT VS MOXI+EHM | time | -0.5633358 | 0.1914761 | 2.94 | 0.067 | -0.9386221 | 0.1880495 |
| CT VS MOXI+IHM | time | 0.1459795 | 0.0877809 | 1.66 | 0.096 | -0.0260679 | 0.3180268 |

Meta-regression analysis of the 2hPG in the primary analysis of DPN: intervention duration(Table S61)

|  |  | **Coefficient** | **Std. err.** | **z** | **P>z** | **[95% conf.interval]** | |
| --- | --- | --- | --- | --- | --- | --- | --- |
| CT VS MOXI | cov1 | -0.0031955 | 0.0029189 | -1.09 | 0.274 | -0.0089165 | 0.0025255 |
| CT VS MOXI+EHM | cov1 | -0.0607144 | 0.0223565 | -1.72 | 0.283 | -0.1045324 | 0.0168964 |
| CT VS MOXI+IHM | cov1 | 0.0117999 | 0.0081681 | 1.44 | 0.149 | -0.0042093 | 0.0278092 |

Meta-regression analysis of the 2hPG in the primary analysis of DPN: age(Table S62)

|  |  | **Coefficient** | **Std. err.** | **z** | **P>z** | **[95% conf.interval]** | |
| --- | --- | --- | --- | --- | --- | --- | --- |
| CT VS MOXI | age | 0.0699466 | 0.0594212 | 1.18 | 0.239 | -0.0465167 | 0.1864099 |
| CT VS MOXI+EHM | age | 0.0926593 | 0.0781452 | 2.57 | 0.056 | -0.0540204 | 0.6029825 |
| CT VS MOXI+IHM | age | 0.0331887 | 0.0620367 | 0.53 | 0.593 | -0.088401 | 0.1547783 |

Meta-regression analysis of the 2hPG in the primary analysis of DPN: disease duration (Table S63)

|  |  | **Coefficient** | **Std. err.** | **z** | **P>z** | **[95% conf.interval]** | |
| --- | --- | --- | --- | --- | --- | --- | --- |
| CT VS MOXI+EHM | time | 0.1450743 | 0.1466634 | 0.99 | 0.323 | -0.1423807 | 0.4325293 |

Meta-regression in the subgroup analysis

Meta-regression analysis based on the common peroneal MNCV in the subgroup analysis of DPN: intervention duration(Table S64)

|  |  | **Coefficient** | **Std. err.** | **z** | **P>z** | **[95% conf.interval]** | |
| --- | --- | --- | --- | --- | --- | --- | --- |
| ACU VS CT | cov1 | -0.6128003 | 0.3087477 | -1.95 | 0.051 | -1.217935 | -0.007666 |
| ACU VS GIM | cov1 | -0.6630362 | 0.3216568 | -1.86 | 0.057 | -1.293472 | -0.0326005 |
| ACU VS HM | cov1 | -0.6427272 | 0.3723467 | -1.73 | 0.084 | -1.372513 | 0.087059 |
| ACU VS MM | cov1 | -0.6040906 | 0.3202772 | -1.89 | 0.059 | -1.231822 | 0.0236412 |
| ACU VS TF | cov1 | -0.6276856 | 0.3667564 | -1.71 | 0.087 | -1.346515 | 0.0911438 |
| ACU VS WDM | cov1 | -0.4964253 | 0.3692218 | -1.34 | 0.179 | -1.220087 | 0.227236 |
| ACU VS WN | cov1 | -0.6671152 | 0.3078824 | -1.87 | 0.055 | -1.270554 | -0.0636767 |

Meta-regression analysis based on the common peroneal MNCV in the subgroup analysis of DPN: age(Table S65)

|  |  | **Coefficient** | **Std. err.** | **z** | **P>z** | **[95% conf. interval** | |
| --- | --- | --- | --- | --- | --- | --- | --- |
| ACU VS CT | age | 0.6444258 | 0.2632589 | 1.98 | 0.052 | -0.1284479 | 1.160404 |
| ACU VS GIM | age | 0.1125135 | 0.6489831 | 0.17 | 0.862 | -1.15947 | 1.384497 |
| ACU VS IHM | age | 0.5393199 | 0.8359497 | 0.65 | 0.519 | -1.099111 | 2.177751 |
| ACU VS MM | age | 0.4273223 | 0.3632446 | 1.18 | 0.239 | -0.2846239 | 1.139269 |
| ACU VS TF | age | 0.6093021 | 0.5715517 | 1.07 | 0.286 | -0.5109187 | 1.729523 |
| ACU VS WDM | age | 0.6531689 | 0.3663258 | 1.78 | 0.075 | -0.0648165 | 1.371154 |
| ACU VS WN | age | 0.470734 | 0.2264209 | 1.92 | 0.058 | -0.0269572 | 0.9145108 |

Meta-regression analysis based on the common peroneal MNCV in the subgroup analysis of DPN:disease duration (Table S66)

|  |  | **Coefficient** | **Std. err.** | **z** | **P>z** | **[95% conf. interval]** |
| --- | --- | --- | --- | --- | --- | --- |
| ACU VS CT | time | 0.2768762 | 12.8843 | 0.02 | 0.983 | -24.97588 25.52964 |
| ACU VS GIM | time | 0.4310898 | 12.95375 | 0.03 | 0.973 | -24.9578 25.81997 |
| ACU VS HM | time | 0.1368278 | 12.91622 | 0.01 | 0.992 | -25.1785 25.45215 |
| ACU VS MM | time | 5.561504 | 126.125 | 0.04 | 0.965 | -241.639 252.762 |
| ACU VS TF | time | 0.3469384 | 12.91227 | 0.03 | 0.979 | -24.96065 25.65453 |
| ACU VS WDM | time | 0.7774463 | 12.90854 | 0.06 | 0.952 | -24.52282 26.07771 |
| ACU VS WN | time | -0.6633679 | 12.88939 | 0.05 | 0.959 | -25.92612 24.59938 |

Meta-regression analysis based on the median MNCV in the subgroup analysis of DPN: intervention duration(Table S67)

|  |  | **Coefficient** | **Std. err.** | **z** | **P>z** | **[95% conf.interval]** | |
| --- | --- | --- | --- | --- | --- | --- | --- |
| ACU VS CT | cov1 | -0.0525513 | 1.374674 | -0.04 | 0.97 | -2.746864 | 2.641761 |
| ACU VS GIM | cov1 | -0.0829022 | 1.380754 | -0.06 | 0.952 | -2.78913 | 2.623326 |
| ACU VS MM | cov1 | -0.7625441 | 2.266413 | -0.34 | 0.737 | -5.204632 | 3.679543 |
| ACU VS TF | cov1 | -0.2637641 | 1.391566 | -0.19 | 0.85 | -2.991184 | 2.463656 |
| ACU VS WDM | cov1 | 0.1337988 | 1.442718 | 0.09 | 0.926 | -2.693877 | 2.961474 |
| ACU VS WN | cov1 | -0.1392932 | 1.374436 | -0.1 | 0.919 | -2.833138 | 2.554552 |

Meta-regression analysis based on the median MNCV in the subgroup analysis of DPN: age(Table S68)

|  |  | **Coefficient** | **Std. err.** | **z** | **P>z** | **[95% conf.interval]** | |
| --- | --- | --- | --- | --- | --- | --- | --- |
| ACU VS CT | age | 0.4630226 | 2.75795 | 0.17 | 0.867 | -4.942459 | 5.868505 |
| ACU VS GIM | age | 0.250407 | 3.089188 | 0.08 | 0.935 | -5.804291 | 6.305105 |
| ACU VS MM | age | 0.2600517 | 2.867353 | 0.09 | 0.928 | -5.359858 | 5.879961 |
| ACU VS TF | age | -0.0250213 | 2.941886 | -0.01 | 0.993 | -5.791012 | 5.740969 |
| ACU VS WDM | age | 0.5869768 | 2.793087 | 0.21 | 0.834 | -4.887372 | 6.061326 |
| ACU VS WN | age | 0.3306381 | 2.731615 | 0.12 | 0.904 | -5.02323 | 5.684506 |

Meta-regression analysis based on the median MNCV in the subgroup analysis of DPN:disease duration (Table S69)

|  |  | **Coefficient** | **Std. err.** | **z** | **P>z** | **[95% conf.interval]** | |
| --- | --- | --- | --- | --- | --- | --- | --- |
| ACU VS CT | time | 0.2928556 | 15.53856 | 0.02 | 0.985 | -30.16216 | 30.74787 |
| ACU VS TF | time | -0.7084312 | 15.55851 | -0.05 | 0.964 | -31.20256 | 29.78569 |
| ACU VS WDM | time | -0.4517094 | 15.58113 | -0.03 | 0.977 | -30.99017 | 30.08675 |
| ACU VS WN | time | -0.9995525 | 15.54099 | -0.06 | 0.949 | -31.45934 | 29.46023 |

Meta-regression analysis based on the common peroneal SNCV in the subgroup analysis of DPN: intervention duration(Table S70)

|  |  | **Coefficient** | **Std. err.** | **z** | **P>z** | **[95% conf.interval]** | |
| --- | --- | --- | --- | --- | --- | --- | --- |
| ACU VS CT | cov1 | -0.0103185 | 1.210613 | -0.01 | 0.993 | -2.383077 | 2.36244 |
| ACU VS MM | cov1 | 0.0764441 | 1.210139 | 0.06 | 0.95 | -2.295384 | 2.448272 |
| ACU VS WDM | cov1 | 0.0546689 | 1.236986 | 0.04 | 0.965 | -2.36978 | 2.479118 |
| ACU VS WN | cov1 | -0.0845475 | 1.211007 | -0.07 | 0.944 | -2.458078 | 2.288983 |

Meta-regression analysis based on the common peroneal SNCV in the subgroup analysis of DPN: age(Table S71)

|  |  | **Coefficient** | **Std. err.** | **z** | **P>z** | **[95% conf.interval]** | |
| --- | --- | --- | --- | --- | --- | --- | --- |
| ACU VS CT | age | 0.0760668 | 2.742393 | 0.03 | 0.978 | -5.298925 | 5.451059 |
| ACU VS EHM | age | 0.4548255 | 3.768922 | 0.12 | 0.904 | -6.932125 | 7.841776 |
| ACU VS MM | age | 0.3201811 | 2.773222 | 0.12 | 0.908 | -5.115235 | 5.755597 |
| ACU VS WDM | age | 0.2052155 | 2.836672 | 0.07 | 0.942 | -5.35456 | 5.764991 |
| ACU VS WN | age | -0.0114175 | 2.74651 | 0 | 0.997 | -5.394478 | 5.371643 |

Meta-regression analysis based on the common peroneal SNCV in the subgroup analysis of DPN:disease duration (Table S72)

|  |  | **Coefficient** | **Std. err.** | **z** | **P>z** | **[95% conf.interval]** | |
| --- | --- | --- | --- | --- | --- | --- | --- |
| ACU VS IHM | time | 0.9111547 | 3.566691 | 0.26 | 0.798 | -6.079431 | 7.901741 |
| ACU VS MM | time | -1.359239 | 0.6538389 | -1.08 | 0.058 | -2.640739 | 0.077738 |

Meta-regression analysis based on the median SNCV in the subgroup analysis of DPN: intervention duration(Table S73)

|  |  | **Coefficient** | **Std. err.** | **z** | **P>z** | **[95% conf.interval]** | |
| --- | --- | --- | --- | --- | --- | --- | --- |
| ACU VS CT | cov1 | -0.0772913 | 1.322144 | -0.06 | 0.953 | -2.668645 | 2.514063 |
| ACU VS HM | cov1 | -0.0701487 | 1.341061 | -0.05 | 0.958 | -2.698579 | 2.558282 |
| ACU VS MM | cov1 | -1.902254 | 2.054379 | -0.93 | 0.354 | -5.928762 | 2.124255 |
| ACU VS WDM | cov1 | -0.3008357 | 1.336443 | -0.23 | 0.822 | -2.920217 | 2.318545 |
| ACU VS WN | cov1 | -0.1560782 | 1.321976 | -0.12 | 0.906 | -2.747103 | 2.434947 |

Meta-regression analysis based on the median SNCV in the subgroup analysis of DPN: age(Table S74)

|  |  | **Coefficient** | **Std. err.** | **z** | **P>z** | **[95% conf.interval]** | |
| --- | --- | --- | --- | --- | --- | --- | --- |
| ACU VS CT | age | 0.5172565 | 2.287429 | 0.23 | 0.821 | -3.966021 | 5.000534 |
| ACU VS EHM | age | 0.2372459 | 32.43362 | 0.01 | 0.994 | -63.33148 | 63.80597 |
| ACU VS HM | age | 0.4920681 | 2.533394 | 0.19 | 0.846 | -4.473292 | 5.457428 |
| ACU VS MM | age | -0.0042522 | 2.370562 | 0 | 0.999 | -4.650469 | 4.641964 |
| ACU VS TF | age | -0.114599 | 2.432595 | -0.05 | 0.962 | -4.882398 | 4.6532 |
| ACU VS WDM | age | -0.1227351 | 32.38076 | 0 | 0.997 | -63.58787 | 63.3424 |
| ACU VS WN | age | 0.3612842 | 2.272904 | 0.16 | 0.874 | -4.093527 | 4.816095 |

Meta-regression analysis based on the median SNCV in the subgroup analysis of DPN:disease duration (Table S75)

|  |  | **Coefficient** | **Std. err.** | **z** | **P>z** | **[95% conf.interval]** | |
| --- | --- | --- | --- | --- | --- | --- | --- |
| ACU VS HM | time | -1.029966 | 0.6148005 | -1.68 | 0.094 | -2.234952 | 0.1750214 |
| ACU VS TF | time | -1.329857 | 0.256993 | -2.17 | 0.052 | -1.833554 | 0.82616 |

Meta-regression analysis based on the TCSS in the subgroup analysis of DPN: intervention duration(Table S76)

|  |  | **Coefficient** | **Std. err.** | **z** | **P>z** | **[95% conf.interval]** | |
| --- | --- | --- | --- | --- | --- | --- | --- |
| ACU VS CT | cov1 | -0.0100877 | 0.1750432 | -0.06 | 0.954 | -0.3531661 | 0.3329908 |
| ACU VS HM | cov1 | -0.0907909 | 0.2203513 | -0.41 | 0.68 | -0.5226715 | 0.3410896 |
| ACU VS MM | cov1 | -0.016019 | 0.1740062 | -0.09 | 0.927 | -0.3570649 | 0.325027 |
| ACU VS TF | cov1 | -0.0450989 | 0.1788053 | -0.25 | 0.801 | -0.3955508 | 0.305353 |
| ACU VS WN | cov1 | 0.011011 | 0.1754196 | 0.06 | 0.95 | -0.332805 | 0.3548271 |

Meta-regression analysis based on the TCSS in the subgroup analysis of DPN: age(Table S77)

|  |  | **Coefficient** | **Std. err.** | **z** | **P>z** | **[95% conf.interval]** | |
| --- | --- | --- | --- | --- | --- | --- | --- |
| ACU VS CT | age | -0.0384882 | 0.2528658 | -0.15 | 0.879 | -0.534096 | 0.4571196 |
| ACU VS HM | age | 0.0557052 | 0.3107262 | 0.18 | 0.858 | -0.5533069 | 0.6647173 |
| ACU VS MM | age | -0.1996242 | 0.3538163 | -0.56 | 0.573 | -0.8930915 | 0.4938431 |
| ACU VS TF | age | -0.7977131 | 1.093564 | -0.73 | 0.466 | -2.941058 | 1.345632 |
| ACU VS WN | age | -0.0126578 | 0.1550645 | -0.08 | 0.935 | -0.3165786 | 0.2912631 |

Meta-regression analysis based on the TCSS in the subgroup analysis of DPN:disease duration (Table S78)

|  |  | **Coefficient** | **Std. err.** | **z** | **P>z** | **[95% conf.interval]** | |
| --- | --- | --- | --- | --- | --- | --- | --- |
| ACU VS CT | time | 0.1537489 | 2.073554 | 0.07 | 0.941 | -3.910342 | 4.21784 |
| ACU VS MM | time | 1.63375 | 2.237 | 0.73 | 0.465 | -2.75069 | 6.018189 |
| ACU VS TF | time | 0.3208089 | 2.075197 | 0.15 | 0.877 | -3.746502 | 4.38812 |

Meta-regression analysis based on the markedly effective rate in the subgroup analysis of DPN: intervention duration(Table S79)

|  |  | **Coefficient** | **Std. err.** | **z** | **P>z** | **[95% conf.interval]** | |
| --- | --- | --- | --- | --- | --- | --- | --- |
| ACU VS CT | cov1 | 0.0483308 | 0.2700581 | 0.18 | 0.858 | -0.4809733 | 0.5776349 |
| ACU VS HM | cov1 | 0.0932265 | 0.2746067 | 0.34 | 0.734 | -0.4449927 | 0.6314456 |
| ACU VS MM | cov1 | -0.0040032 | 0.3486107 | -0.01 | 0.991 | -0.6872677 | 0.6792613 |
| ACU VS TF | cov1 | 0.0500133 | 0.2704123 | 0.18 | 0.853 | -0.4799851 | 0.5800117 |
| ACU VS WDM | cov1 | 0.0428286 | 0.2720767 | 0.16 | 0.875 | -0.4904319 | 0.5760892 |
| ACU VS WN | cov1 | 0.0421177 | 0.2699881 | 0.16 | 0.876 | -0.4870492 | 0.5712847 |

Meta-regression analysis based on the markedly effective rate in the subgroup analysis of DPN: age(Table S80)

|  |  | **Coefficient** | **Std. err.** | **z** | **P>z** | **[95% conf.interval]** | |
| --- | --- | --- | --- | --- | --- | --- | --- |
| ACU VS CT | age | -0.0113422 | 0.0507579 | -0.22 | 0.823 | -0.110826 | 0.0881415 |
| ACU VS EHM | age | -0.1956364 | 0.3413082 | -0.57 | 0.567 | -0.8645883 | 0.4733154 |
| ACU VS HM | age | -0.1370564 | 0.1639749 | -0.84 | 0.403 | -0.4584414 | 0.1843285 |
| ACU VS MM | age | 0.0424362 | 0.1330045 | 0.32 | 0.75 | -0.218248 | 0.3031203 |
| ACU VS TF | age | -0.007055 | 0.0695184 | -0.1 | 0.919 | -0.1433085 | 0.1291985 |
| ACU VS WDM | age | -0.0223299 | 0.097204 | -0.23 | 0.818 | -0.2128463 | 0.1681865 |
| ACU VS WN | age | -0.0109984 | 0.0470616 | -0.23 | 0.815 | -0.1032375 | 0.0812406 |

Meta-regression analysis based on the markedly effective rate in the subgroup analysis of DPN:disease duration (Table S81)

|  |  | **Coefficient** | **Std. err.** | **z** | **P>z** | **[95% conf.interval]** | |
| --- | --- | --- | --- | --- | --- | --- | --- |
| ACU VS CT | time | 0.0842626 | 5.062125 | 0.02 | 0.987 | -9.83732 | 10.00584 |
| ACU VS MM | time | 0.0371323 | 5.069404 | 0.01 | 0.994 | -9.898716 | 9.972981 |
| ACU VS TF | time | 0.0071763 | 5.074652 | 0 | 0.999 | -9.93896 | 9.953312 |
| ACU VS WDM | time | -0.2862957 | 5.066827 | -0.06 | 0.955 | -10.21709 | 9.644503 |

Meta-regression analysis based on the FPG in the subgroup analysis of DPN: intervention duration(Table S82)

|  |  | **Coefficient** | **Std. err.** | **z** | **P>z** | **[95% conf. interval]** | |
| --- | --- | --- | --- | --- | --- | --- | --- |
| CT VS MM | cov1 | 0.0229125 | 0.0402126 | 0.57 | 0.569 | -0.0559027 | 0.1017276 |
| CT VS TF | cov1 | -0.0238095 | 0.029738 | -0.8 | 0.423 | -0.0820949 | 0.0344759 |
| CT VS WDM | cov1 | -0.0014815 | 0.0510446 | -0.03 | 0.977 | -0.1015272 | 0.0985642 |
| CT VS WN | cov1 | -0.0003716 | 0.0200228 | -0.02 | 0.985 | -0.0396156 | 0.0388724 |

Meta-regression analysis based on the FPG in the subgroup analysis of DPN: age(Table S83)

|  |  | **Coefficient** | **Std. err.** | **z** | **P>z** | **[95% conf.interval]** | |
| --- | --- | --- | --- | --- | --- | --- | --- |
| CT VS MM | age | 0.1546692 | 0.4017262 | 0.39 | 0.7 | -0.6326997 | 0.9420381 |
| CT VS TF | age | -0.4999999 | 0.6691916 | -0.75 | 0.455 | -1.811591 | 0.8115916 |
| CT VS WDM | age | -0.2600011 | 8.985246 | -0.03 | 0.977 | -17.87076 | 17.35076 |
| CT VS WN | age | -0.019215 | 0.288569 | -0.07 | 0.947 | -0.5847998 | 0.5463698 |

Meta-regression analysis based on the FPG in the subgroup analysis of DPN:disease duration (Table S84)

|  |  | **Coefficient** | **Std. err.** | **z** | **P>z** | **[95% conf. interval]** | |
| --- | --- | --- | --- | --- | --- | --- | --- |
| CT VS MM | time | 0.9999995 | 0.3155152 | 1.17 | 0.054 | -0.831601 | 1.618398 |
| CT VS WDM | time | 0.1099999 | 0.6274585 | 0.18 | 0.861 | -1.119796 | 1.339796 |

Meta-regression analysis based on the 2hPG in the subgroup analysis of DPN: intervention duration(Table S85)

|  |  | **Coefficient** | **Std. err.** | **z** | **P>z** | **[95% conf. interval]** | |
| --- | --- | --- | --- | --- | --- | --- | --- |
| CT VS GIM | cov1 | 0.0007143 | 0.0150937 | 0.05 | 0.962 | -0.0288689 | 0.0302975 |
| CT VS MM | cov1 | -0.0507692 | 0.0371128 | -1.37 | 0.171 | -0.1235089 | 0.0219705 |
| CT VS WDM | cov1 | -0.0023504 | 0.0892071 | -0.03 | 0.979 | -0.1771931 | 0.1724922 |
| CT VS WN | cov1 | 0.0037533 | 0.0081766 | 0.46 | 0.646 | -0.0122725 | 0.0197791 |

Meta-regression analysis based on the 2hPG in the subgroup analysis of DPN: age(Table S86)

|  |  | **Coefficient** | **Std. err.** | **z** | **P>z** | **[95% conf. interval]** | |
| --- | --- | --- | --- | --- | --- | --- | --- |
| CT VS GIM | age | 0.005 | 0.1056567 | 0.05 | 0.962 | -0.2020833 | 0.2120833 |
| CT VS HM | age | 0.0588889 | 0.056995 | 1.03 | 0.301 | -0.0528192 | 0.170597 |
| CT VS MM | age | -0.4399999 | 0.3216502 | -1.37 | 0.171 | -1.070423 | 0.1904229 |
| CT VS WDM | age | -0.4124913 | 21.10854 | -0.02 | 0.984 | -41.78448 | 40.9595 |
| CT VS WN | age | -0.066104 | 0.1024979 | -0.64 | 0.519 | -0.2669962 | 0.1347883 |

Meta-regression analysis based on the 2hPG in the subgroup analysis of DPN:disease duration (Table S87)

|  |  | **Coefficient** | **Std. err.** | **z** | **P>z** | **[95% conf. interval]** | |
| --- | --- | --- | --- | --- | --- | --- | --- |
| CT VS WN | time | 0.2999997 | 0.5152406 | 0.58 | 0.56 | -0.7098533 | 1.309853 |

Sensitivity analysis of correlation coefficients in the primary analysis

P-values of inconsistency tests for common peroneal MNCV in the primary analysis of DPN patients under different correlation coefficient settings(Table S88)

| **Outcome** | **P value (Global Inconsistency Test)** |
| --- | --- |
| common peroneal MNCV:R=0.5 | 0.3547 |
| common peroneal MNCV:R=0.25 | 0.3603 |
| common peroneal MNCV:R=0.75 | 0.3491 |

P-values of inconsistency tests for median MNCV in the primary analysis of DPN patients under different correlation coefficient settings(Table S89)

| **Outcome** | **P value (Global Inconsistency Test)** |
| --- | --- |
| median MNCV:R=0.5 | 0.066 |
| median MNCV:R=0.25 | 0.0694 |
| median MNCV:R=0.75 | 0.0625 |

P-values of inconsistency tests for common peroneal SNCV in the primary analysis of DPN patients under different correlation coefficient settings(Table S90)

| **Outcome** | **P value (Global Inconsistency Test)** |
| --- | --- |
| common peroneal SNCV:R=0.5 | 0.3267 |
| common peroneal SNCV:R=0.25 | 0.3428 |
| common peroneal SNCV:R=0.75 | 0.3086 |

P-values of inconsistency tests for median SNCV in the primary analysis of DPN patients under different correlation coefficient settings(Table S91)

| **Outcome** | **P value (Global Inconsistency Test)** |
| --- | --- |
| median SNCV:R=0.5 | 0.0561 |
| median SNCV:R=0.25 | 0.0599 |
| median SNCV:R=0.75 | 0.0521 |

P-values of inconsistency tests for TCSS in the primary analysis of DPN patients under different correlation coefficient settings(Table S92)

| **Outcome** | **P value (Global Inconsistency Test)** |
| --- | --- |
| TCSS:R=0.5 | 0.9394 |
| TCSS:R=0.25 | 0.9378 |
| TCSS:R=0.75 | 0.9442 |

Consistency assessment for common peroneal MNCV in the primary analysis of DPN patients with a correlation coefficient of R=0.5(Table S93)

|  |  | **Coefficient** | **Std. err.** | **z** | **P>z** | **[95% conf.interval]** | |
| --- | --- | --- | --- | --- | --- | --- | --- |
| ACU VS CT | _cons | 2.151734 | 1.985036 | 1.08 | 0.278 | -1.738865 | 6.042332 |
| ACU VS EHM | _cons | 5.008618 | 2.65196 | 1.89 | 0.059 | -0.1891283 | 10.20636 |
| ACU VS IHM | _cons | 1.387656 | 3.354297 | 0.41 | 0.679 | -5.186645 | 7.961958 |
| ACU VS MOXI | _cons | 5.204285 | 1.786872 | 2.91 | 0.004 | 1.702081 | 8.706489 |
| ACU VS MOXI+ACU | _cons | 7.536862 | 2.441758 | 3.09 | 0.002 | 2.751105 | 12.32262 |
| ACU VS MOXI+EHM | _cons | 8.290773 | 2.181344 | 3.8 | 0 | 4.015418 | 12.56613 |
| ACU VS MOXI+IHM | _cons | 6.098404 | 2.22149 | 2.75 | 0.006 | 1.744365 | 10.45244 |
| ACU VS MOXI+MT | _cons | 5.836612 | 2.658828 | 2.2 | 0.028 | 0.6254049 | 11.04782 |

Consistency assessment for common peroneal MNCV in the primary analysis of DPN patients with a correlation coefficient of R=0.25(Table S94)

|  |  | **Coefficient** | **Std. err.** | **z** | **P>z** | **[95% conf. interval]** | |
| --- | --- | --- | --- | --- | --- | --- | --- |
| ACU VS CT | _cons | 2.134324 | 2.003713 | 1.07 | 0.287 | -1.792882 | 6.061529 |
| ACU VS EHM | _cons | 4.960463 | 2.683109 | 1.85 | 0.064 | -0.298333 | 10.21926 |
| ACU VS IHM | _cons | 1.365565 | 3.373286 | 0.4 | 0.686 | -5.245955 | 7.977085 |
| ACU VS MOXI | _cons | 5.165883 | 1.806587 | 2.86 | 0.004 | 1.625039 | 8.706728 |
| ACU VS MOXI+ACU | _cons | 7.568349 | 2.453651 | 3.08 | 0.002 | 2.759281 | 12.37742 |
| ACU VS MOXI+EHM | _cons | 8.297061 | 2.204563 | 3.76 | 0 | 3.976197 | 12.61793 |
| ACU VS MOXI+IHM | _cons | 6.076082 | 2.234423 | 2.72 | 0.007 | 1.696693 | 10.45547 |
| ACU VS MOXI+MT | _cons | 5.85578 | 2.676299 | 2.19 | 0.029 | 0.6103313 | 11.10123 |

Consistency assessment for common peroneal MNCV in the primary analysis of DPN patients with a correlation coefficient of R=0.75(Table S95)

|  |  | **Coefficient** | **Std. err.** | **z** | **P>z** | **[95% conf.** | **interval]** |
| --- | --- | --- | --- | --- | --- | --- | --- |
| ACU VS CT | _cons | 2.147822 | 1.962896 | 1.09 | 0.274 | -1.699384 | 5.995028 |
| ACU VS EHM | _cons | 5.03864 | 2.617342 | 1.93 | 0.054 | -0.0912562 | 10.16854 |
| ACU VS IHM | _cons | 1.387749 | 3.334936 | 0.42 | 0.677 | -5.148606 | 7.924103 |
| ACU VS MOXI | _cons | 5.226089 | 1.764739 | 2.96 | 0.003 | 1.767264 | 8.684915 |
| ACU VS MOXI+ACU | _cons | 7.480625 | 2.426645 | 3.08 | 0.002 | 2.724488 | 12.23676 |
| ACU VS MOXI+EHM | _cons | 8.25983 | 2.154495 | 3.83 | 0 | 4.037098 | 12.48256 |
| ACU VS MOXI+IHM | _cons | 6.099131 | 2.205515 | 2.77 | 0.006 | 1.776402 | 10.42186 |
| ACU VS MOXI+MT | _cons | 5.794727 | 2.638574 | 2.2 | 0.028 | 0.6232173 | 10.96624 |

Consistency assessment for median MNCV in the primary analysis of DPN patients with a correlation coefficient of R=0.5(Table S96)

|  |  | **Coefficient** | **Std. err.** | **z** | **P>z** | **[95% conf. interval]** | |
| --- | --- | --- | --- | --- | --- | --- | --- |
| ACU VS CT | _cons | 2.823797 | 3.132234 | 0.9 | 0.367 | -3.315269 | 8.962863 |
| ACU VS EHM | _cons | 4.424953 | 4.356885 | 1.02 | 0.31 | -4.114384 | 12.96429 |
| ACU VS MOXI | _cons | 5.651308 | 2.787065 | 2.03 | 0.043 | 0.1887618 | 11.11385 |
| ACU VS MOXI+ACU | _cons | 9.292499 | 4.104175 | 2.26 | 0.024 | 1.248464 | 17.33654 |
| ACU VS MOXI+EHM | _cons | 9.176138 | 3.447386 | 2.66 | 0.008 | 2.419386 | 15.93289 |
| ACU VS MOXI+IHM | _cons | 6.916987 | 3.29005 | 2.1 | 0.036 | 0.4686075 | 13.36537 |
| ACU VS MOXI+MT | _cons | 6.859615 | 3.676433 | 1.87 | 0.062 | -0.3460604 | 14.06529 |

Consistency assessment for median MNCV in the primary analysis of DPN patients with a correlation coefficient of R=0.25(Table S97)

|  |  | **Coefficient** | **Std. err.** | **z** | **P>z** | **[95% conf. interval]** | |
| --- | --- | --- | --- | --- | --- | --- | --- |
| ACU VS CT | _cons | 2.824078 | 3.20171 | 0.88 | 0.378 | -3.451157 | 9.099314 |
| ACU VS EHM | _cons | 4.458692 | 4.415466 | 1.01 | 0.313 | -4.195463 | 13.11285 |
| ACU VS MOXI | _cons | 5.662501 | 2.858996 | 1.98 | 0.048 | 0.0589708 | 11.26603 |
| ACU VS MOXI+ACU | _cons | 9.293192 | 4.156175 | 2.24 | 0.025 | 1.147238 | 17.43915 |
| ACU VS MOXI+EHM | _cons | 9.209495 | 3.515234 | 2.62 | 0.009 | 2.319762 | 16.09923 |
| ACU VS MOXI+IHM | _cons | 6.93637 | 3.357107 | 2.07 | 0.039 | 0.3565608 | 13.51618 |
| ACU VS MOXI+MT | _cons | 6.854029 | 3.748399 | 1.83 | 0.067 | -0.4926977 | 14.20076 |

Consistency assessment for median MNCV in the primary analysis of DPN patients with a correlation coefficient of R=0.75(Table S98)

|  |  | **Coefficient** | **Std. err.** | **z** | **P>z** | **[95% conf.interval]** | |
| --- | --- | --- | --- | --- | --- | --- | --- |
| ACU VS CT | _cons | 2.798312 | 3.054732 | 0.92 | 0.36 | -3.188853 | 8.785476 |
| ACU VS EHM | _cons | 4.365085 | 4.293209 | 1.02 | 0.309 | -4.04945 | 12.77962 |
| ACU VS MOXI | _cons | 5.619551 | 2.708559 | 2.07 | 0.038 | 0.3108727 | 10.92823 |
| ACU VS MOXI+ACU | _cons | 9.26592 | 4.046541 | 2.29 | 0.022 | 1.334845 | 17.19699 |
| ACU VS MOXI+EHM | _cons | 9.117393 | 3.372883 | 2.7 | 0.007 | 2.506664 | 15.72812 |
| ACU VS MOXI+IHM | _cons | 6.872972 | 3.214994 | 2.14 | 0.033 | 0.5716993 | 13.17424 |
| ACU VS MOXI+MT | _cons | 6.83972 | 3.596857 | 1.9 | 0.057 | -0.2099905 | 13.88943 |

Consistency assessment for common peroneal SNCV in the primary analysis of DPN patients with a correlation coefficient of R=0.5(Table S99)

|  |  | **Coefficient** | **Std. err.** | **z** | **P>z** | **[95% conf. interval]** | |
| --- | --- | --- | --- | --- | --- | --- | --- |
| ACU VS CT | _cons | -3.657015 | 3.43882 | -1.06 | 0.288 | -10.39698 | 3.082949 |
| ACU VS EHM | _cons | 0.9075826 | 4.276301 | 0.21 | 0.832 | -7.473813 | 9.288978 |
| ACU VS IHM | _cons | -2.748209 | 4.426746 | -0.62 | 0.535 | -11.42447 | 5.928055 |
| ACU VS MOXI | _cons | -0.8043797 | 3.5728 | -0.23 | 0.822 | -7.806938 | 6.198179 |
| ACU VS MOXI+ACU | _cons | 2.341927 | 2.478593 | 0.94 | 0.345 | -2.516025 | 7.199879 |
| ACU VS MOXI+EHM | _cons | 4.785793 | 3.905772 | 1.23 | 0.22 | -2.869379 | 12.44096 |
| ACU VS MOXI+IHM | _cons | 0.9216125 | 3.64466 | 0.25 | 0.8 | -6.22179 | 8.065015 |
| ACU VS MOXI+MT | _cons | 0.3132943 | 4.301976 | 0.07 | 0.942 | -8.118424 | 8.745012 |

Consistency assessment for common peroneal SNCV in the primary analysis of DPN patients with a correlation coefficient of R=0.25(Table S100)

|  |  | **Coefficient** | **Std. err.** | **z** | **P>z** | **[95% conf. interval]** | |
| --- | --- | --- | --- | --- | --- | --- | --- |
| ACU VS CT | _cons | -3.664083 | 3.497808 | -1.05 | 0.295 | -10.51966 | 3.191495 |
| ACU VS EHM | _cons | 0.9281315 | 4.358222 | 0.21 | 0.831 | -7.613826 | 9.470089 |
| ACU VS IHM | _cons | -2.724674 | 4.508947 | -0.6 | 0.546 | -11.56205 | 6.112701 |
| ACU VS MOXI | _cons | -0.8215157 | 3.635063 | -0.23 | 0.821 | -7.946107 | 6.303076 |
| ACU VS MOXI+ACU | _cons | 2.338362 | 2.533028 | 0.92 | 0.356 | -2.626283 | 7.303006 |
| ACU VS MOXI+EHM | _cons | 4.805045 | 3.982646 | 1.21 | 0.228 | -3.000797 | 12.61089 |
| ACU VS MOXI+IHM | _cons | 0.9452039 | 3.703611 | 0.26 | 0.799 | -6.31374 | 8.204148 |
| ACU VS MOXI+MT | _cons | 0.3061394 | 4.406734 | 0.07 | 0.945 | -8.330901 | 8.94318 |

Consistency assessment for common peroneal SNCV in the primary analysis of DPN patients with a correlation coefficient of R=0.75(Table S101)

|  |  | **Coefficient** | **Std. err.** | **z** | **P>z** | **[95% conf. interval]** | |
| --- | --- | --- | --- | --- | --- | --- | --- |
| ACU VS CT | _cons | -3.637023 | 3.358254 | -1.08 | 0.279 | -10.21908 | 2.945034 |
| ACU VS EHM | _cons | 0.8951073 | 4.172901 | 0.21 | 0.83 | -7.283628 | 9.073843 |
| ACU VS IHM | _cons | -2.7589 | 4.325086 | -0.64 | 0.524 | -11.23591 | 5.718113 |
| ACU VS MOXI | _cons | -0.7733654 | 3.488364 | -0.22 | 0.825 | -7.610434 | 6.063703 |
| ACU VS MOXI+ACU | _cons | 2.352011 | 2.414845 | 0.97 | 0.33 | -2.380998 | 7.08502 |
| ACU VS MOXI+EHM | _cons | 4.775122 | 3.806642 | 1.25 | 0.21 | -2.685758 | 12.236 |
| ACU VS MOXI+IHM | _cons | 0.9107686 | 3.56445 | 0.26 | 0.798 | -6.075425 | 7.896963 |
| ACU VS MOXI+MT | _cons | 0.3335399 | 4.176893 | 0.08 | 0.936 | -7.85302 | 8.52 |

Consistency assessment for median SNCV in the primary analysis of DPN patients with a correlation coefficient of R=0.5(Table S99)

|  |  | **Coefficient** | **Std. err.** | **z** | **P>z** | **[95% conf. interval]** | |
| --- | --- | --- | --- | --- | --- | --- | --- |
| ACU VS CT | _cons | 3.301873 | 3.129417 | 1.06 | 0.291 | -2.831671 | 9.435417 |
| ACU VS EHM | _cons | 2.943033 | 3.913474 | 0.75 | 0.452 | -4.727235 | 10.6133 |
| ACU VS MOXI | _cons | 6.597469 | 2.861243 | 2.31 | 0.021 | 0.9895368 | 12.2054 |
| ACU VS MOXI+ACU | _cons | 9.90906 | 4.140113 | 2.39 | 0.017 | 1.794587 | 18.02353 |
| ACU VS MOXI+EHM | _cons | 7.62099 | 3.393029 | 2.25 | 0.025 | 0.9707762 | 14.2712 |
| ACU VS MOXI+IHM | _cons | 7.344915 | 3.279007 | 2.24 | 0.025 | 0.9181782 | 13.77165 |

Consistency assessment for median SNCV in the primary analysis of DPN patients with a correlation coefficient of R=0.25(Table S100)

|  |  | **Coefficient** | **Std. err.** | **z** | **P>z** | **[95% conf. interval]** | |
| --- | --- | --- | --- | --- | --- | --- | --- |
| ACU VS CT | _cons | 3.327959 | 3.214859 | 1.04 | 0.301 | -2.973048 | 9.628966 |
| ACU VS EHM | _cons | 3.023224 | 3.998086 | 0.76 | 0.45 | -4.812881 | 10.85933 |
| ACU VS MOXI | _cons | 6.614385 | 2.947218 | 2.24 | 0.025 | 0.8379441 | 12.39082 |
| ACU VS MOXI+ACU | _cons | 9.936052 | 4.208767 | 2.36 | 0.018 | 1.68702 | 18.18508 |
| ACU VS MOXI+EHM | _cons | 7.699571 | 3.479092 | 2.21 | 0.027 | 0.8806749 | 14.51847 |
| ACU VS MOXI+IHM | _cons | 7.391493 | 3.364289 | 2.2 | 0.028 | 0.7976085 | 13.98538 |

Consistency assessment for median SNCV in the primary analysis of DPN patients with a correlation coefficient of R=0.75(Table S101)

|  |  | **Coefficient** | **Std. err.** | **z** | **P>z** | **[95% conf. interval]** | |
| --- | --- | --- | --- | --- | --- | --- | --- |
| ACU VS CT | _cons | 3.239921 | 3.031888 | 1.07 | 0.285 | -2.702471 | 9.182313 |
| ACU VS EHM | _cons | 2.82344 | 3.818143 | 0.74 | 0.46 | -4.659982 | 10.30686 |
| ACU VS MOXI | _cons | 6.550144 | 2.764846 | 2.37 | 0.018 | 1.131145 | 11.96914 |
| ACU VS MOXI+ACU | _cons | 9.844565 | 4.062091 | 2.42 | 0.015 | 1.883013 | 17.80612 |
| ACU VS MOXI+EHM | _cons | 7.503934 | 3.295272 | 2.28 | 0.023 | 1.045319 | 13.96255 |
| ACU VS MOXI+IHM | _cons | 7.261969 | 3.181711 | 2.28 | 0.022 | 1.025929 | 13.49801 |

Consistency assessment for TCSS in the primary analysis of DPN patients with a correlation coefficient of R=0.5(Table S102)

|  |  | **Coefficient** | **Std. err.** | **z** | **P>z** | **[95% conf. interval]** | |
| --- | --- | --- | --- | --- | --- | --- | --- |
| ACU VS CT | _cons | -0.1151501 | 0.9349396 | -0.12 | 0.902 | -1.947598 | 1.717298 |
| ACU VS EHM | _cons | -1.674872 | 1.429127 | -1.17 | 0.241 | -4.47591 | 1.126165 |
| ACU VS MOXI | _cons | -1.566004 | 0.7598116 | -2.06 | 0.039 | -3.055208 | -0.0768009 |
| ACU VS MOXI+ACU | _cons | -1.44 | 1.185603 | -1.21 | 0.225 | -3.763739 | 0.8837389 |
| ACU VS MOXI+EHM | _cons | -2.668484 | 1.066785 | -2.5 | 0.012 | -4.759344 | -0.5776238 |
| ACU VS MOXI+IHM | _cons | -1.691975 | 1.047339 | -1.62 | 0.106 | -3.744722 | 0.360771 |

Consistency assessment for TCSS in the primary analysis of DPN patients with a correlation coefficient of R=0.25(Table S103)

|  |  | **Coefficient** | **Std. err.** | **z** | **P>z** | **[95% conf. interval]** | |
| --- | --- | --- | --- | --- | --- | --- | --- |
| ACU VS CT | _cons | -0.1533346 | 0.9603763 | -0.16 | 0.873 | -2.035638 | 1.728968 |
| ACU VS EHM | _cons | -1.692888 | 1.53343 | -1.1 | 0.27 | -4.698357 | 1.31258 |
| ACU VS MOXI | _cons | -1.574806 | 0.7812339 | -2.02 | 0.044 | -3.105996 | -0.0436155 |
| ACU VS MOXI+ACU | _cons | -1.44 | 1.262317 | -1.14 | 0.254 | -3.914097 | 1.034097 |
| ACU VS MOXI+EHM | _cons | -2.689153 | 1.101193 | -2.44 | 0.015 | -4.847452 | -0.5308546 |
| ACU VS MOXI+IHM | _cons | -1.722472 | 1.073414 | -1.6 | 0.109 | -3.826325 | 0.381381 |

Consistency assessment for TCSS in the primary analysis of DPN patients with a correlation coefficient of R=0.75(Table S104)

|  |  | **Coefficient** | **Std. err.** | **z** | **P>z** | **[95% conf. interval]** | |
| --- | --- | --- | --- | --- | --- | --- | --- |
| ACU VS CT | _cons | -0.0595467 | 0.8991029 | -0.07 | 0.947 | -1.821756 | 1.702663 |
| ACU VS EHM | _cons | -1.642321 | 1.307397 | -1.26 | 0.209 | -4.204772 | 0.9201293 |
| ACU VS MOXI | _cons | -1.545348 | 0.7312355 | -2.11 | 0.035 | -2.978543 | -0.1121527 |
| ACU VS MOXI+ACU | _cons | -1.44 | 1.101495 | -1.31 | 0.191 | -3.59889 | 0.7188896 |
| ACU VS MOXI+EHM | _cons | -2.631624 | 1.019673 | -2.58 | 0.01 | -4.630146 | -0.633101 |
| ACU VS MOXI+IHM | _cons | -1.645499 | 1.011208 | -1.63 | 0.104 | -3.62743 | 0.3364323 |

Consistency assessment for FPG in the primary analysis of DPN patients with a correlation coefficient of R=0.5(Table S105)

|  |  | **Coefficient** | **Std. err.** | **z** | **P>z** | **[95% conf.interval]** | |
| --- | --- | --- | --- | --- | --- | --- | --- |
| CT VS EHM | _cons | -0.0161825 | 0.2097391 | -0.08 | 0.939 | -0.4272635 | 0.3948986 |
| CT VS MOXI | _cons | -0.2725349 | 0.0357399 | -7.63 | 0 | -0.3425839 | -0.202486 |
| CT VS MOXI+EHM | _cons | -0.356188 | 0.1508601 | -2.36 | 0.018 | -0.6518684 | -0.0605076 |
| CT VS MOXI+IHM | _cons | -1.275198 | 0.1144611 | -11.14 | 0 | -1.499538 | -1.050858 |

Consistency assessment for FPG in the primary analysis of DPN patients with a correlation coefficient of R=0.25(Table S106)

|  |  | **Coefficient** | **Std. err.** | **z** | **P>z** | **[95% conf.interval]** | |
| --- | --- | --- | --- | --- | --- | --- | --- |
| CT VS EHM | _cons | -0.0257758 | 0.2494853 | -0.1 | 0.918 | -0.5147579 | 0.4632064 |
| CT VS MOXI | _cons | -0.2733701 | 0.0432299 | -6.32 | 0 | -0.3580992 | -0.1886411 |
| CT VS MOXI+EHM | _cons | -0.3657817 | 0.1752241 | -2.09 | 0.037 | -0.7092146 | -0.0223487 |
| CT VS MOXI+IHM | _cons | -1.276404 | 0.1374199 | -9.29 | 0 | -1.545742 | -1.007066 |

Consistency assessment for FPG in the primary analysis of DPN patients with a correlation coefficient of R=0.75(Table S107)

|  |  | **Coefficient** | **Std. err.** | **z** | **P>z** | **[95% conf.interval]** | |
| --- | --- | --- | --- | --- | --- | --- | --- |
| CT VS EHM | _cons | 0.001299 | 0.1603132 | 0.01 | 0.994 | -0.3129091 | 0.3155072 |
| CT VS MOXI | _cons | -0.3211307 | 0.1508063 | -2.13 | 0.033 | -0.6167056 | -0.0255559 |
| CT VS MOXI+EHM | _cons | -0.3387059 | 0.1215579 | -2.79 | 0.005 | -0.576955 | -0.1004568 |
| CT VS MOXI+IHM | _cons | -1.270896 | 0.0849072 | -14.97 | 0 | -1.437311 | -1.104481 |

Consistency assessment for 2hPG in the primary analysis of DPN patients with a correlation coefficient of R=0.5(Table S108)

|  |  | **Coefficient** | **Std. err.** | **z** | **P>z** | **[95% conf.interval]** | |
| --- | --- | --- | --- | --- | --- | --- | --- |
| CT VS EHM | _cons | 0.116134 | 0.3774813 | 0.31 | 0.758 | -0.6237158 | 0.8559837 |
| CT VS MOXI | _cons | -0.3235516 | 0.3300019 | -0.98 | 0.327 | -0.9703435 | 0.3232402 |
| CT VS MOXI+EHM | _cons | -0.7538775 | 0.2776431 | -2.72 | 0.007 | -1.298048 | -0.2097071 |
| CT VS MOXI+IHM | _cons | -1.430544 | 0.150369 | -9.51 | 0 | -1.725262 | -1.135826 |
| CT VS MT | _cons | -1.96 | 0.3004728 | -6.52 | 0 | -2.548916 | -1.371084 |

Consistency assessment for 2hPG in the primary analysis of DPN patients with a correlation coefficient of R=0.25(Table S109)

|  |  | **Coefficient** | **Std. err.** | **z** | **P>z** | **[95% conf. interval]** | |
| --- | --- | --- | --- | --- | --- | --- | --- |
| CT VS EHM | _cons | 0.0837435 | 0.4519878 | 0.19 | 0.853 | -0.8021363 | 0.9696232 |
| CT VS MOXI | _cons | -0.3603491 | 0.3874102 | -0.93 | 0.352 | -1.119659 | 0.3989609 |
| CT VS MOXI+EHM | _cons | -0.7862691 | 0.3271889 | -2.4 | 0.016 | -1.427547 | -0.1449907 |
| CT VS MOXI+IHM | _cons | -1.425414 | 0.1789127 | -7.97 | 0 | -1.776077 | -1.074752 |
| CT VS MT | _cons | -1.96 | 0.3679389 | -5.33 | 0 | -2.681147 | -1.238853 |

Consistency assessment for 2hPG in the primary analysis of DPN patients with a correlation coefficient of R=0.75(Table S110)

|  |  | **Coefficient** | **Std. err.** | **z** | **P>z** | **[95% conf. interval]** | |
| --- | --- | --- | --- | --- | --- | --- | --- |
| CT VS EHM | _cons | 0.1712623 | 0.2832837 | 0.6 | 0.545 | -0.3839635 | 0.7264882 |
| CT VS MOXI | _cons | -0.2544607 | 0.2500919 | -1.02 | 0.309 | -0.7446318 | 0.2357103 |
| CT VS MOXI+EHM | _cons | -0.6987472 | 0.2160489 | -3.23 | 0.001 | -1.122195 | -0.2752992 |
| CT VS MOXI+IHM | _cons | -1.446153 | 0.1142217 | -12.66 | 0 | -1.670024 | -1.222283 |
| CT VS MT | _cons | -1.96 | 0.2125764 | -9.22 | 0 | -2.376642 | -1.543358 |

Sensitivity analysis of correlation coefficients in the subgroup analysis

P-values of inconsistency tests for TCSS in the subgroup analysis of DPN patients under different correlation coefficient settings(Table S111)

| **Outcome** | **P value (Global Inconsistency Test)** |
| --- | --- |
| TCSS:R=0.5 | 0.4515 |
| TCSS:R=0.25 | 0.4719 |
| TCSS:R=0.75 | 0.4268 |

Consistency assessment for common peroneal MNCV in the subgroup analysis of DPN patients with a correlation coefficient of R=0.5(Table S112)

|  |  | **Coefficient** | **Std. err.** | **z** | **P>z** | **[95% conf. interval]** | |
| --- | --- | --- | --- | --- | --- | --- | --- |
| ACU VS CT | _cons | 1.874996 | 1.805537 | 1.04 | 0.299 | -1.663793 | 5.413784 |
| ACU VS EHM | _cons | 1.931411 | 3.089077 | 0.63 | 0.532 | -4.12307 | 7.985891 |
| ACU VS GIM | _cons | 5.29927 | 2.224477 | 2.38 | 0.017 | 0.939376 | 9.659164 |
| ACU VS HM | _cons | 4.089157 | 2.4195 | 1.69 | 0.091 | -0.6529747 | 8.831289 |
| ACU VS IHM | _cons | 3.047019 | 3.093575 | 0.98 | 0.325 | -3.016275 | 9.110314 |
| ACU VS MM | _cons | 7.757892 | 2.118193 | 3.66 | 0 | 3.606311 | 11.90947 |
| ACU VS TF | _cons | 8.254498 | 2.37681 | 3.47 | 0.001 | 3.596036 | 12.91296 |
| ACU VS WDM | _cons | 6.072139 | 2.210169 | 2.75 | 0.006 | 1.740288 | 10.40399 |
| ACU VS WN | _cons | 5.184467 | 1.60798 | 3.22 | 0.001 | 2.032884 | 8.336051 |

Consistency assessment for common peroneal MNCV in the subgroup analysis of DPN patients with a correlation coefficient of R=0.25(Table S113)

|  |  | **Coefficient** | **Std. err.** | **z** | **P>z** | **[95% conf. interval]** | |
| --- | --- | --- | --- | --- | --- | --- | --- |
| ACU VS CT | _cons | 1.814525 | 1.840509 | 0.99 | 0.324 | -1.792806 | 5.421857 |
| ACU VS EHM | _cons | 1.877037 | 3.112819 | 0.6 | 0.547 | -4.223976 | 7.97805 |
| ACU VS GIM | _cons | 5.226038 | 2.265944 | 2.31 | 0.021 | 0.7848704 | 9.667206 |
| ACU VS HM | _cons | 4.029815 | 2.468646 | 1.63 | 0.103 | -0.8086424 | 8.868273 |
| ACU VS IHM | _cons | 2.971779 | 3.147844 | 0.94 | 0.345 | -3.197882 | 9.141439 |
| ACU VS MM | _cons | 7.682392 | 2.154843 | 3.57 | 0 | 3.458977 | 11.90581 |
| ACU VS TF | _cons | 8.192399 | 2.406693 | 3.4 | 0.001 | 3.475368 | 12.90943 |
| ACU VS WDM | _cons | 6.017519 | 2.245551 | 2.68 | 0.007 | 1.61632 | 10.41872 |
| ACU VS WN | _cons | 5.136703 | 1.64593 | 3.12 | 0.002 | 1.910739 | 8.362667 |

Consistency assessment for common peroneal MNCV in the subgroup analysis of DPN patients with a correlation coefficient of R=0.75(Table S114)

|  |  | **Coefficient** | **Std. err.** | **z** | **P>z** | **[95% conf. interval]** | |
| --- | --- | --- | --- | --- | --- | --- | --- |
| ACU VS CT | _cons | 1.925808 | 1.769113 | 1.09 | 0.276 | -1.541589 | 5.393205 |
| ACU VS EHM | _cons | 1.974988 | 3.068005 | 0.64 | 0.52 | -4.038192 | 7.988168 |
| ACU VS GIM | _cons | 5.361751 | 2.181743 | 2.46 | 0.014 | 1.085613 | 9.63789 |
| ACU VS HM | _cons | 4.138692 | 2.369971 | 1.75 | 0.081 | -0.5063663 | 8.78375 |
| ACU VS IHM | _cons | 3.112888 | 3.040946 | 1.02 | 0.306 | -2.847257 | 9.073033 |
| ACU VS MM | _cons | 7.824479 | 2.080624 | 3.76 | 0 | 3.746531 | 11.90243 |
| ACU VS TF | _cons | 8.306387 | 2.347349 | 3.54 | 0 | 3.705666 | 12.90711 |
| ACU VS WDM | _cons | 6.116433 | 2.174426 | 2.81 | 0.005 | 1.854637 | 10.37823 |
| ACU VS WN | _cons | 5.224776 | 1.569178 | 3.33 | 0.001 | 2.149243 | 8.300309 |

Consistency assessment for median MNCV in the subgroup analysis of DPN patients with a correlation coefficient of R=0.5(Table S115)

|  |  | **Coefficient** | **Std. err.** | **z** | **P>z** | **[95% conf. interval]** | |
| --- | --- | --- | --- | --- | --- | --- | --- |
| ACU VS CT | _cons | 0.4534153 | 1.196303 | 0.38 | 0.705 | -1.891296 | 2.798126 |
| ACU VS EHM | _cons | 0.2268657 | 1.529317 | 0.15 | 0.882 | -2.770541 | 3.224272 |
| ACU VS GIM | _cons | 5.241923 | 1.429923 | 3.67 | 0 | 2.439325 | 8.044521 |
| ACU VS HM | _cons | 3.823365 | 1.535491 | 2.49 | 0.013 | 0.8138588 | 6.832872 |
| ACU VS MM | _cons | 5.111947 | 1.365463 | 3.74 | 0 | 2.43569 | 7.788205 |
| ACU VS TF | _cons | 4.906771 | 1.265423 | 3.88 | 0 | 2.426587 | 7.386956 |
| ACU VS WDM | _cons | 4.976906 | 1.314484 | 3.79 | 0 | 2.400564 | 7.553249 |
| ACU VS WN | _cons | 5.68663 | 1.15421 | 4.93 | 0 | 3.42442 | 7.948839 |

Consistency assessment for median MNCV in the subgroup analysis of DPN patients with a correlation coefficient of R=0.25(Table S116)

|  |  | **Coefficient** | **Std. err.** | **z** | **P>z** | **[95% conf. interval]** | |
| --- | --- | --- | --- | --- | --- | --- | --- |
| ACU VS CT | _cons | 0.4757264 | 1.459776 | 0.33 | 0.745 | -2.385381 | 3.336834 |
| ACU VS EHM | _cons | 0.2610668 | 1.864653 | 0.14 | 0.889 | -3.393586 | 3.915719 |
| ACU VS GIM | _cons | 5.26401 | 1.746645 | 3.01 | 0.003 | 1.840648 | 8.687372 |
| ACU VS HM | _cons | 3.845676 | 1.875871 | 2.05 | 0.04 | 0.1690359 | 7.522316 |
| ACU VS MM | _cons | 5.131176 | 1.66597 | 3.08 | 0.002 | 1.865935 | 8.396418 |
| ACU VS TF | _cons | 4.943317 | 1.543627 | 3.2 | 0.001 | 1.917864 | 7.968771 |
| ACU VS WDM | _cons | 5.011108 | 1.60119 | 3.13 | 0.002 | 1.872833 | 8.149382 |
| ACU VS WN | _cons | 5.686568 | 1.408955 | 4.04 | 0 | 2.925067 | 8.448069 |

Consistency assessment for median MNCV in the subgroup analysis of DPN patients with a correlation coefficient of R=0.75(Table S117)

|  |  | **Coefficient** | **Std. err.** | **z** | **P>z** | **[95% conf. interval]** | |
| --- | --- | --- | --- | --- | --- | --- | --- |
| ACU VS CT | _cons | 0.3951692 | 0.8551425 | 0.46 | 0.644 | -1.280879 | 2.071218 |
| ACU VS EHM | _cons | 0.1363941 | 1.095542 | 0.12 | 0.901 | -2.010828 | 2.283616 |
| ACU VS GIM | _cons | 5.184348 | 1.019079 | 5.09 | 0 | 3.186991 | 7.181706 |
| ACU VS HM | _cons | 3.76512 | 1.093847 | 3.44 | 0.001 | 1.621219 | 5.909022 |
| ACU VS MM | _cons | 5.062622 | 0.9764244 | 5.18 | 0 | 3.148866 | 6.976379 |
| ACU VS TF | _cons | 4.809249 | 0.9053447 | 5.31 | 0 | 3.034806 | 6.583692 |
| ACU VS WDM | _cons | 4.886434 | 0.9441669 | 5.18 | 0 | 3.035901 | 6.736967 |
| ACU VS WN | _cons | 5.686786 | 0.8241626 | 6.9 | 0 | 4.071457 | 7.302115 |

Consistency assessment for common peroneal SNCV in the subgroup analysis of DPN patients with a correlation coefficient of R=0.5(Table S118)

|  |  | **Coefficient** | **Std. err.** | **z** | **P>z** | **[95% conf. interval]** | |
| --- | --- | --- | --- | --- | --- | --- | --- |
| ACU VS CT | _cons | -3.089 | 3.290521 | -0.94 | 0.348 | -9.538304 | 3.360303 |
| ACU VS EHM | _cons | -3.475891 | 4.23428 | -0.82 | 0.412 | -11.77493 | 4.823146 |
| ACU VS GIM | _cons | 0.881235 | 4.30494 | 0.2 | 0.838 | -7.556293 | 9.318763 |
| ACU VS HM | _cons | -1.52808 | 4.224585 | -0.36 | 0.718 | -9.808115 | 6.751954 |
| ACU VS IHM | _cons | -1.327497 | 3.806055 | -0.35 | 0.727 | -8.787227 | 6.132233 |
| ACU VS MM | _cons | 2.342618 | 2.673552 | 0.88 | 0.381 | -2.897448 | 7.582684 |
| ACU VS TF | _cons | 2.921029 | 4.206948 | 0.69 | 0.487 | -5.324438 | 11.1665 |
| ACU VS WDM | _cons | 0.4015014 | 3.792942 | 0.11 | 0.916 | -7.032528 | 7.835531 |
| ACU VS WN | _cons | 0.981677 | 3.46141 | 0.28 | 0.777 | -5.802562 | 7.765916 |

Consistency assessment for common peroneal SNCV in the subgroup analysis of DPN patients with a correlation coefficient of R=0.25(Table S119)

|  |  | **Coefficient** | **Std. err.** | **z** | **P>z** | **[95% conf. interval]** | |
| --- | --- | --- | --- | --- | --- | --- | --- |
| ACU VS CT | _cons | -3.086238 | 3.384155 | -0.91 | 0.362 | -9.719059 | 3.546584 |
| ACU VS EHM | _cons | -3.474735 | 4.340626 | -0.8 | 0.423 | -11.98221 | 5.032736 |
| ACU VS GIM | _cons | 0.8839306 | 4.442183 | 0.2 | 0.842 | -7.822588 | 9.590449 |
| ACU VS HM | _cons | -1.525603 | 4.325191 | -0.35 | 0.724 | -10.00282 | 6.951616 |
| ACU VS IHM | _cons | -1.331271 | 3.899885 | -0.34 | 0.733 | -8.974905 | 6.312363 |
| ACU VS MM | _cons | 2.33881 | 2.738207 | 0.85 | 0.393 | -3.027976 | 7.705596 |
| ACU VS TF | _cons | 2.923782 | 4.299321 | 0.68 | 0.496 | -5.502733 | 11.3503 |
| ACU VS WDM | _cons | 0.4018926 | 3.89372 | 0.1 | 0.918 | -7.229659 | 8.033444 |
| ACU VS WN | _cons | 1.00019 | 3.555655 | 0.28 | 0.778 | -5.968766 | 7.969146 |

Consistency assessment for common peroneal SNCV in the subgroup analysis of DPN patients with a correlation coefficient of R=0.75(Table S120)

|  |  | **Coefficient** | **Std. err.** | **z** | **P>z** | **[95% conf. interval]** | |
| --- | --- | --- | --- | --- | --- | --- | --- |
| ACU VS CT | _cons | -3.082387 | 3.176706 | -0.97 | 0.332 | -9.308616 | 3.143841 |
| ACU VS EHM | _cons | -3.466606 | 4.108038 | -0.84 | 0.399 | -11.51821 | 4.585 |
| ACU VS GIM | _cons | 0.8880439 | 4.147693 | 0.21 | 0.83 | -7.241285 | 9.017373 |
| ACU VS HM | _cons | -1.520634 | 4.105572 | -0.37 | 0.711 | -9.567407 | 6.526139 |
| ACU VS IHM | _cons | -1.316772 | 3.700062 | -0.36 | 0.722 | -8.56876 | 5.935215 |
| ACU VS MM | _cons | 2.353443 | 2.5966 | 0.91 | 0.365 | -2.735799 | 7.442685 |
| ACU VS TF | _cons | 2.927665 | 4.096576 | 0.71 | 0.475 | -5.101476 | 10.95681 |
| ACU VS WDM | _cons | 0.4113519 | 3.671892 | 0.11 | 0.911 | -6.785424 | 7.608128 |
| ACU VS WN | _cons | 0.9718893 | 3.346545 | 0.29 | 0.771 | -5.587218 | 7.530996 |

Consistency assessment for median SNCV in the subgroup analysis of DPN patients with a correlation coefficient of R=0.5(Table S121)

|  |  | **Coefficient** | **Std. err.** | **z** | **P>z** | **[95% conf. interval]** | |
| --- | --- | --- | --- | --- | --- | --- | --- |
| ACU VS CT | _cons | 0.8411069 | 1.12564 | 0.75 | 0.455 | -1.365107 | 3.047321 |
| ACU VS EHM | _cons | -1.387468 | 1.456125 | -0.95 | 0.341 | -4.241421 | 1.466484 |
| ACU VS GIM | _cons | 5.881041 | 1.448444 | 4.06 | 0 | 3.042142 | 8.719939 |
| ACU VS HM | _cons | 3.05772 | 1.266981 | 2.41 | 0.016 | 0.5744829 | 5.540958 |
| ACU VS MM | _cons | 7.417435 | 1.258114 | 5.9 | 0 | 4.951576 | 9.883294 |
| ACU VS TF | _cons | 6.331131 | 1.204755 | 5.26 | 0 | 3.969856 | 8.692406 |
| ACU VS WDM | _cons | 3.240973 | 1.359826 | 2.38 | 0.017 | 0.5757641 | 5.906183 |
| ACU VS WN | _cons | 6.655042 | 1.100217 | 6.05 | 0 | 4.498656 | 8.811429 |

Consistency assessment for median SNCV in the subgroup analysis of DPN patients with a correlation coefficient of R=0.25(Table S122)

|  |  | **Coefficient** | **Std. err.** | **z** | **P>z** | **[95% conf. interval]** | |
| --- | --- | --- | --- | --- | --- | --- | --- |
| ACU VS CT | _cons | 0.8493336 | 1.371642 | 0.62 | 0.536 | -1.839035 | 3.537703 |
| ACU VS EHM | _cons | -1.384583 | 1.776417 | -0.78 | 0.436 | -4.866296 | 2.09713 |
| ACU VS GIM | _cons | 5.889267 | 1.768365 | 3.33 | 0.001 | 2.423335 | 9.355199 |
| ACU VS HM | _cons | 3.320904 | 1.565636 | 2.12 | 0.034 | 0.252313 | 6.389494 |
| ACU VS MM | _cons | 7.425428 | 1.53445 | 4.84 | 0 | 4.41796 | 10.43289 |
| ACU VS TF | _cons | 6.014852 | 1.461315 | 4.12 | 0 | 3.150727 | 8.878976 |
| ACU VS WDM | _cons | 3.249198 | 1.659626 | 1.96 | 0.05 | -0.0036093 | 6.502005 |
| ACU VS WN | _cons | 6.654972 | 1.340758 | 4.96 | 0 | 4.027135 | 9.28281 |

Consistency assessment for median SNCV in the subgroup analysis of DPN patients with a correlation coefficient of R=0.75(Table S123)

|  |  | **Coefficient** | **Std. err.** | **z** | **P>z** | **[95% conf. interval]** | |
| --- | --- | --- | --- | --- | --- | --- | --- |
| ACU VS CT | _cons | 0.8222788 | 0.80788 | 1.02 | 0.309 | -0.7611369 | 2.405694 |
| ACU VS EHM | _cons | -1.391338 | 1.041414 | -1.34 | 0.182 | -3.432472 | 0.6497958 |
| ACU VS GIM | _cons | 5.862213 | 1.033832 | 5.67 | 0 | 3.83594 | 7.888486 |
| ACU VS HM | _cons | 3.037692 | 0.9091087 | 3.34 | 0.001 | 1.255872 | 4.819513 |
| ACU VS MM | _cons | 7.399307 | 0.900615 | 8.22 | 0 | 5.634134 | 9.16448 |
| ACU VS TF | _cons | 6.355609 | 0.8666105 | 7.33 | 0 | 4.657083 | 8.054134 |
| ACU VS WDM | _cons | 3.222151 | 0.9715112 | 3.32 | 0.001 | 1.318024 | 5.126278 |
| ACU VS WN | _cons | 6.655094 | 0.7895075 | 8.43 | 0 | 5.107688 | 8.202501 |

Consistency assessment for TCSS in the subgroup analysis of DPN patients with a correlation coefficient of R=0.5(Table S124)

|  |  | **Coefficient** | **Std. err.** | **z** | **P>z** | **[95% conf. interval]** | |
| --- | --- | --- | --- | --- | --- | --- | --- |
| ACU VS CT | _cons | 0.2427151 | 0.8138591 | 0.3 | 0.766 | -1.352419 | 1.83785 |
| ACU VS HM | _cons | -1.466492 | 1.153923 | -1.27 | 0.204 | -3.728139 | 0.795155 |
| ACU VS MM | _cons | -2.094826 | 0.8854936 | -2.37 | 0.018 | -3.830361 | -0.3592902 |
| ACU VS TF | _cons | -1.364147 | 1.122507 | -1.22 | 0.224 | -3.56422 | 0.8359257 |
| ACU VS WN | _cons | -1.289789 | 0.7256572 | -1.78 | 0.076 | -2.712051 | 0.1324727 |

Consistency assessment for TCSS in the subgroup analysis of DPN patients with a correlation coefficient of R=0.25(Table S125)

|  |  | **Coefficient** | **Std. err.** | **z** | **P>z** | **[95% conf. interval]** | |
| --- | --- | --- | --- | --- | --- | --- | --- |
| ACU VS CT | _cons | 0.2148702 | 0.8377857 | 0.26 | 0.798 | -1.42716 | 1.8569 |
| ACU VS HM | _cons | -1.478111 | 1.183895 | -1.25 | 0.212 | -3.798503 | 0.8422815 |
| ACU VS MM | _cons | -2.122265 | 0.9166211 | -2.32 | 0.021 | -3.918809 | -0.3257205 |
| ACU VS TF | _cons | -1.364587 | 1.144116 | -1.19 | 0.233 | -3.607013 | 0.8778398 |
| ACU VS WN | _cons | -1.305517 | 0.7443312 | -1.75 | 0.079 | -2.764379 | 0.15334 |

Consistency assessment for TCSS in the subgroup analysis of DPN patients with a correlation coefficient of R=0.75(Table S126)

|  |  | **Coefficient** | **Std. err.** | **z** | **P>z** | **[95% conf. interval]** | |
| --- | --- | --- | --- | --- | --- | --- | --- |
| ACU VS CT | _cons | 0.2820081 | 0.7816281 | 0.36 | 0.718 | -1.249955 | 1.813971 |
| ACU VS HM | _cons | -1.44506 | 1.116301 | -1.29 | 0.195 | -3.63297 | 0.7428493 |
| ACU VS MM | _cons | -2.056532 | 0.8473721 | -2.43 | 0.015 | -3.717351 | -0.3957136 |
| ACU VS TF | _cons | -1.348959 | 1.088216 | -1.24 | 0.215 | -3.481823 | 0.7839051 |
| ACU VS WN | _cons | -1.267173 | 0.7022161 | -1.8 | 0.071 | -2.643491 | 0.1091458 |

Consistency assessment for FPG in the subgroup analysis of DPN patients with a correlation coefficient of R=0.5(Table S127)

|  |  | **Coefficient** | **Std. err.** | **z** | **P>z** | **[95% conf. interval]** | |
| --- | --- | --- | --- | --- | --- | --- | --- |
| CT VS EHM | _cons | -0.3486891 | 0.989168 | -0.35 | 0.724 | -2.287423 | 1.590045 |
| CT VS GIM | _cons | -1.64 | 0.6428495 | -2.55 | 0.011 | -2.899962 | -0.380038 |
| CT VS HM | _cons | -0.26 | 0.5908656 | -0.44 | 0.66 | -1.418075 | 0.8980754 |
| CT VS MM | _cons | -1.122398 | 0.4114433 | -2.73 | 0.006 | -1.928812 | -0.3159841 |
| CT VS TF | _cons | -0.7048758 | 0.4314677 | -1.63 | 0.102 | -1.550537 | 0.1407853 |
| CT VS WDM | _cons | -0.6889662 | 0.7970844 | -0.86 | 0.387 | -2.251223 | 0.8732904 |
| CT VS WN | _cons | -0.7451403 | 0.4284547 | -1.74 | 0.082 | -1.584896 | 0.0946154 |

Consistency assessment for FPG in the subgroup analysis of DPN patients with a correlation coefficient of R=0.25(Table S128)

|  |  | **Coefficient** | **Std. err.** | **z** | **P>z** | **[95% conf. interval]** | |
| --- | --- | --- | --- | --- | --- | --- | --- |
| CT VS EHM | _cons | -0.3491988 | 0.9315955 | -0.37 | 0.708 | -2.175092 | 1.476695 |
| CT VS GIM | _cons | -1.64 | 0.6157792 | -2.66 | 0.008 | -2.846905 | -0.433095 |
| CT VS HM | _cons | -0.26 | 0.5392327 | -0.48 | 0.63 | -1.316877 | 0.7968767 |
| CT VS MM | _cons | -1.166787 | 0.4021836 | -2.9 | 0.004 | -1.955052 | -0.3785212 |
| CT VS TF | _cons | -0.6673133 | 0.4041398 | -1.65 | 0.099 | -1.459413 | 0.1247862 |
| CT VS WDM | _cons | -0.6893553 | 0.762966 | -0.9 | 0.366 | -2.184741 | 0.8060305 |
| CT VS WN | _cons | -0.7515493 | 0.4243616 | -1.77 | 0.077 | -1.583283 | 0.0801841 |

Consistency assessment for FPG in the subgroup analysis of DPN patients with a correlation coefficient of R=0.75(Table S129)

|  |  | **Coefficient** | **Std. err.** | **z** | **P>z** | **[95% conf. interval]** | |
| --- | --- | --- | --- | --- | --- | --- | --- |
| CT VS EHM | _cons | -0.3471083 | 1.060151 | -0.33 | 0.743 | -2.424965 | 1.730749 |
| CT VS GIM | _cons | -1.64 | 0.6826694 | -2.4 | 0.016 | -2.978007 | -0.3019925 |
| CT VS HM | _cons | -0.26 | 0.6528269 | -0.4 | 0.69 | -1.539517 | 1.019517 |
| CT VS MM | _cons | -1.071769 | 0.4230523 | -2.53 | 0.011 | -1.900936 | -0.2426019 |
| CT VS TF | _cons | -0.7505779 | 0.4759693 | -1.58 | 0.115 | -1.68346 | 0.1823047 |
| CT VS WDM | _cons | -0.6877674 | 0.840265 | -0.82 | 0.413 | -2.334656 | 0.9591217 |
| CT VS WN | _cons | -0.7381852 | 0.4384907 | -1.68 | 0.092 | -1.597611 | 0.1212407 |

Consistency assessment for 2hPG in the subgroup analysis of DPN patients with a correlation coefficient of R=0.5(Table S130)

|  |  | **Coefficient** | **Std. err.** | **z** | **P>z** | **[95% conf. interval]** | |
| --- | --- | --- | --- | --- | --- | --- | --- |
| CT VS EHM | _cons | -0.3892667 | 0.9489357 | -0.41 | 0.682 | -2.249146 | 1.470613 |
| CT VS GIM | _cons | -1.970109 | 0.2112998 | -9.32 | 0 | -2.384249 | -1.555969 |
| CT VS HM | _cons | -0.3060295 | 0.2408705 | -1.27 | 0.204 | -0.778127 | 0.166068 |
| CT VS MM | _cons | -2.012506 | 0.4055607 | -4.96 | 0 | -2.80739 | -1.217621 |
| CT VS TF | _cons | -1.1 | 0.339834 | -3.24 | 0.001 | -1.766063 | -0.4339377 |
| CT VS WDM | _cons | -1.259296 | 0.913853 | -1.38 | 0.168 | -3.050415 | 0.5318226 |
| CT VS WN | _cons | -1.144873 | 0.2137439 | -5.36 | 0 | -1.563803 | -0.7259422 |

Consistency assessment for 2hPG in the subgroup analysis of DPN patients with a correlation coefficient of R=0.25(Table S131)

|  |  | **Coefficient** | **Std. err.** | **z** | **P>z** | **[95% conf. interval]** | |
| --- | --- | --- | --- | --- | --- | --- | --- |
| CT VS EHM | _cons | -0.3892995 | 1.131916 | -0.34 | 0.731 | -2.607813 | 1.829214 |
| CT VS GIM | _cons | -1.970169 | 0.2579606 | -7.64 | 0 | -2.475763 | -1.464576 |
| CT VS HM | _cons | -0.3129342 | 0.2886541 | -1.08 | 0.278 | -0.8786858 | 0.2528174 |
| CT VS MM | _cons | -2.022735 | 0.4595322 | -4.4 | 0 | -2.923401 | -1.122068 |
| CT VS TF | _cons | -1.1 | 0.4063621 | -2.71 | 0.007 | -1.896455 | -0.3035451 |
| CT VS WDM | _cons | -1.259329 | 1.08815 | -1.16 | 0.247 | -3.392064 | 0.8734054 |
| CT VS WN | _cons | -1.132893 | 0.2502676 | -4.53 | 0 | -1.623408 | -0.6423774 |

Consistency assessment for 2hPG in the subgroup analysis of DPN patients with a correlation coefficient of R=0.75(Table S132)

|  |  | **Coefficient** | **Std. err.** | **z** | **P>z** | **[95% conf. interval]** | |
| --- | --- | --- | --- | --- | --- | --- | --- |
| CT VS EHM | _cons | -0.3891713 | 0.7209132 | -0.54 | 0.589 | -1.802135 | 1.023793 |
| CT VS GIM | _cons | -1.969935 | 0.1508052 | -13.06 | 0 | -2.265507 | -1.674362 |
| CT VS HM | _cons | -0.2870246 | 0.180195 | -1.59 | 0.111 | -0.6402003 | 0.0661511 |
| CT VS MM | _cons | -1.995293 | 0.3429299 | -5.82 | 0 | -2.667423 | -1.323163 |
| CT VS TF | _cons | -1.1 | 0.2566014 | -4.29 | 0 | -1.60293 | -0.5970707 |
| CT VS WDM | _cons | -1.259201 | 0.6972629 | -1.81 | 0.071 | -2.625812 | 0.1074088 |
| CT VS WN | _cons | -1.16429 | 0.1674274 | -6.95 | 0 | -1.492442 | -0.8361387 |

Leave-one-out sensitivity analysis in the primary analysis

Leave-one-out sensitivity analysis of common peroneal MNCV in the primary analysis of patients with DPN (Table S133)

| **dropped_id** | **comparison** | **eff** | **lci** | **uci** | **connected** |
| --- | --- | --- | --- | --- | --- |
| Ma Guoqing 2018 | CT VS ACU | 0.6289182 | -4.067283 | 5.5850053 | 1 |
| Ma Guoqing 2018 | CT VS EHM | 2.8412988 | -0.8197042 | 6.502302 | 1 |
| Ma Guoqing 2018 | CT VS IHM | 0.76129431 | -5.741816 | 4.219227 | 1 |
| Ma Guoqing 2018 | CT VS MOXI | 3.0409197 | 1.438418 | 4.643422 | 1 |
| Ma Guoqing 2018 | CT VS MOXI+ACU | 5.4005483 | 2.784978 | 8.016119 | 1 |
| Ma Guoqing 2018 | CT VS MOXI+EHM | 6.1453588 | 3.961274 | 8.329444 | 1 |
| Ma Guoqing 2018 | CT VS MOXI+IHM | 3.9489879 | 2.115963 | 5.782013 | 1 |
| Ma Guoqing 2018 | CT VS MOXI+MT | 3.6952804 | 0.4374304 | 6.953131 | 1 |
| Feng Yun 2022 | CT VS ACU | 0.2851501 | -3.92321 | 4.552909 | 1 |
| Feng Yun 2022 | CT VS EHM | 2.8449286 | -0.8742018 | 6.564059 | 1 |
| Feng Yun 2022 | CT VS IHM | 1.4895909 | -6.628865 | 7.649684 | 1 |
| Feng Yun 2022 | CT VS MOXI | 3.0422699 | 1.411973 | 4.672567 | 1 |
| Feng Yun 2022 | CT VS MOXI+ACU | 5.3968087 | 2.734435 | 8.059183 | 1 |
| Feng Yun 2022 | CT VS MOXI+EHM | 6.1430898 | 3.922507 | 8.363672 | 1 |
| Feng Yun 2022 | CT VS MOXI+IHM | 3.2205676 | 1.164005 | 5.27713 | 1 |
| Feng Yun 2022 | CT VS MOXI+MT | 3.6925962 | 0.3783839 | 7.006808 | 1 |
| Xie Lingling 2025 | CT VS ACU | -2.1956205 | -6.160624 | 1.769383 | 1 |
| Xie Lingling 2025 | CT VS EHM | 2.8571221 | -1.074864 | 6.789108 | 1 |
| Xie Lingling 2026 | CT VS IHM | 0.76263006 | -6.142595 | 6.617334 | 1 |
| Xie Lingling 2027 | CT VS MOXI | 3.0469904 | 1.314849 | 4.779131 | 1 |
| Xie Lingling 2028 | CT VS MOXI+ACU | 5.3845632 | 2.55124 | 8.217887 | 1 |
| Xie Lingling 2029 | CT VS MOXI+EHM | 6.1355784 | 3.781369 | 8.489787 | 1 |
| Xie Lingling 2030 | CT VS MOXI+IHM | 3.9476976 | 1.95966 | 5.935735 | 1 |
| Xie Lingling 2025 | CT VS MOXI+MT | 2.4399998 | 0.690861 | 7.570861 | 1 |
| Wang Dan 2021 | CT VS ACU | 0.8096836 | -3.465279 | 4.8459117 | 1 |
| Wang Dan 2021 | CT VS EHM | 2.496151 | -1.115898 | 6.108201 | 1 |
| Wang Dan 2021 | CT VS IHM | 0.76091847 | -5.650322 | 6.128485 | 1 |
| Wang Dan 2021 | CT VS MOXI | 2.4078017 | 0.7343299 | 4.081274 | 1 |
| Wang Dan 2021 | CT VS MOXI+ACU | 5.4047375 | 2.838954 | 7.970521 | 1 |
| Wang Dan 2021 | CT VS MOXI+EHM | 5.9061678 | 3.749416 | 8.06292 | 1 |
| Wang Dan 2021 | CT VS MOXI+IHM | 3.9493539 | 2.151793 | 5.746914 | 1 |
| Wang Dan 2021 | CT VS MOXI+MT | 3.6982914 | 0.5003997 | 6.896183 | 1 |
| Ji Hua 2025 | CT VS ACU | 0.4292146 | -3.435226 | 3.576797 | 1 |
| Ji Hua 2025 | CT VS EHM | 2.728666 | -1.20904 | 6.666372 | 1 |
| Ji Hua 2025 | CT VS IHM | 0.76258908 | -6.128101 | 4.602923 | 1 |
| Ji Hua 2025 | CT VS MOXI | 2.812701 | 0.9703541 | 4.655048 | 1 |
| Ji Hua 2025 | CT VS MOXI+ACU | 5.3850816 | 2.559622 | 8.210542 | 1 |
| Ji Hua 2025 | CT VS MOXI+EHM | 6.0453793 | 3.684263 | 8.406495 | 1 |
| Ji Hua 2025 | CT VS MOXI+IHM | 3.9477369 | 1.965296 | 5.930177 | 1 |
| Ji Hua 2025 | CT VS MOXI+MT | 3.6841999 | 0.1732288 | 7.195171 | 1 |
| Bai Fubin 2025 | CT VS ACU | 0.193252 | -3.103184 | 3.71668 | 1 |
| Bai Fubin 2025 | CT VS EHM | 2.8543342 | -1.025998 | 6.734666 | 1 |
| Bai Fubin 2025 | CT VS IHM | 3.0459123 | -1.338463 | 4.753362 | 1 |
| Bai Fubin 2025 | CT VS MOXI | 5.3873392 | 2.59547 | 8.179208 | 1 |
| Bai Fubin 2025 | CT VS MOXI+ACU | 6.1373056 | 3.815519 | 8.459092 | 1 |
| Bai Fubin 2025 | CT VS MOXI+EHM | 3.9480088 | 1.989388 | 5.90663 | 1 |
| Bai Fubin 2025 | CT VS MOXI+IHM | 3.6858138 | 0.2153625 | 7.156265 | 1 |
| Tang Huazhen 2022 | CT VS ACU | 0.1956205 | -3.160624 | 3.769383 | 1 |
| Tang Huazhen 2022 | CT VS EHM | 2.8571221 | -1.074864 | 6.789108 | 1 |
| Tang Huazhen 2022 | CT VS IHM | 0.76263006 | -6.142595 | 6.617334 | 1 |
| Tang Huazhen 2022 | CT VS MOXI | 3.0469904 | 1.314849 | 4.779131 | 1 |
| Tang Huazhen 2022 | CT VS MOXI+ACU | 5.3845632 | 2.55124 | 8.217887 | 1 |
| Tang Huazhen 2022 | CT VS MOXI+EHM | 6.1355784 | 3.781369 | 8.489787 | 1 |
| Tang Huazhen 2022 | CT VS MOXI+IHM | 3.9476976 | 1.95966 | 5.935735 | 1 |
| Tang Huazhen 2022 | CT VS MOXI+MT | 4.7900001 | 0.0486128 | 9.628613 | 1 |
| Wu Naijun 2015 | CT VS ACU | 0.1530229 | -3.223153 | 3.917108 | 1 |
| Wu Naijun 2015 | CT VS EHM | 2.8838987 | -1.110634 | 6.878432 | 1 |
| Wu Naijun 2015 | CT VS IHM | 0.76281303 | -5.209404 | 6.683777 | 1 |
| Wu Naijun 2015 | CT VS MOXI | 3.0925025 | 1.212058 | 4.972947 | 1 |
| Wu Naijun 2015 | CT VS MOXI+ACU | 5.3822251 | 2.512655 | 8.251796 | 1 |
| Wu Naijun 2015 | CT VS MOXI+EHM | 6.151387 | 3.754369 | 8.548405 | 1 |
| Wu Naijun 2015 | CT VS MOXI+IHM | 3.9475226 | 1.933694 | 5.961351 | 1 |
| Wu Naijun 2015 | CT VS MOXI+MT | 3.6821596 | 0.1179213 | 7.246398 | 1 |
| Xu Xiaowei 2025 | CT VS ACU | 0.9635662 | -3.686199 | 5.759067 | 1 |
| Xu Xiaowei 2025 | CT VS EHM | 3.6918255 | -0.1224559 | 7.506107 | 1 |
| Xu Xiaowei 2025 | CT VS IHM | 0.7615006 | -5.795423 | 6.272421 | 1 |
| Xu Xiaowei 2025 | CT VS MOXI | 3.262179 | 1.623937 | 4.900421 | 1 |
| Xu Xiaowei 2025 | CT VS MOXI+ACU | 5.3981952 | 2.753451 | 8.04294 | 1 |
| Xu Xiaowei 2025 | CT VS MOXI+EHM | 7.2094535 | 4.706179 | 9.712728 | 1 |
| Xu Xiaowei 2025 | CT VS MOXI+IHM | 3.9487875 | 2.094997 | 5.802578 | 1 |
| Xu Xiaowei 2025 | CT VS MOXI+MT | 3.693591 | 0.4006047 | 6.986577 | 1 |
| Zhang Suzhao 2024 | CT VS ACU | 0.1945315 | -3.133928 | 3.474865 | 1 |
| Zhang Suzhao 2024 | CT VS EHM | 2.8558381 | -1.052135 | 6.763811 | 1 |
| Zhang Suzhao 2024 | CT VS IHM | 1.1450222 | -6.563176 | 7.273131 | 1 |
| Zhang Suzhao 2024 | CT VS MOXI | 3.0464939 | 1.325832 | 4.767156 | 1 |
| Zhang Suzhao 2024 | CT VS MOXI+ACU | 5.38584 | 2.571777 | 8.199903 | 1 |
| Zhang Suzhao 2024 | CT VS MOXI+EHM | 6.1363746 | 3.797238 | 8.475512 | 1 |
| Zhang Suzhao 2024 | CT VS MOXI+IHM | 3.5652236 | 1.399526 | 5.730921 | 1 |
| Zhang Suzhao 2024 | CT VS MOXI+MT | 3.6847419 | 0.1875304 | 7.181953 | 1 |
| Hu Guanghui 2024 | CT VS ACU | 0.192316 | -3.081154 | 3.696522 | 1 |
| Hu Guanghui 2024 | CT VS EHM | 2.8532373 | -1.00732 | 6.713795 | 1 |
| Hu Guanghui 2024 | CT VS IHM | 0.27514302 | -5.620577 | 6.070291 | 1 |
| Hu Guanghui 2024 | CT VS MOXI | 3.0454879 | 1.347499 | 4.743476 | 1 |
| Hu Guanghui 2024 | CT VS MOXI+ACU | 5.3884353 | 2.612422 | 8.164448 | 1 |
| Hu Guanghui 2024 | CT VS MOXI+EHM | 6.1379834 | 3.828609 | 8.447357 | 1 |
| Hu Guanghui 2024 | CT VS MOXI+IHM | 4.4352688 | 2.303735 | 6.566803 | 1 |
| Hu Guanghui 2024 | CT VS MOXI+MT | 3.6865977 | 0.2353124 | 7.137883 | 1 |
| Liu Xiangxiu 2012 | CT VS ACU | 0.1969398 | -3.193666 | 3.799786 | 1 |
| Liu Xiangxiu 2012 | CT VS EHM | 2.8586826 | -1.103052 | 6.820417 | 1 |
| Liu Xiangxiu 2012 | CT VS IHM | 0.59051724 | -6.087383 | 6.906349 | 1 |
| Liu Xiangxiu 2012 | CT VS MOXI | 3.0475937 | 1.301236 | 4.793952 | 1 |
| Liu Xiangxiu 2012 | CT VS MOXI+ACU | 5.3830153 | 2.52584 | 8.240191 | 1 |
| Liu Xiangxiu 2012 | CT VS MOXI+EHM | 6.1346088 | 3.76173 | 8.507487 | 1 |
| Liu Xiangxiu 2012 | CT VS MOXI+IHM | 4.1198517 | 1.92479 | 6.314913 | 1 |
| Liu Xiangxiu 2012 | CT VS MOXI+MT | 3.6827239 | 0.1334555 | 7.231992 | 1 |
| Xu Lilii 2021 | CT VS ACU | 0.1968734 | -3.191982 | 3.798236 | 1 |
| Xu Lilii 2021 | CT VS EHM | 2.8586039 | -1.101614 | 6.818822 | 1 |
| Xu Lilii 2021 | CT VS IHM | 0.76274544 | -6.184315 | 6.658824 | 1 |
| Xu Lilii 2021 | CT VS MOXI | 3.0475633 | 1.30193 | 4.793197 | 1 |
| Xu Lilii 2021 | CT VS MOXI+ACU | 4.9056487 | 1.370356 | 8.440942 | 1 |
| Xu Lilii 2021 | CT VS MOXI+EHM | 6.1346577 | 3.762731 | 8.506585 | 1 |
| Xu Lilii 2021 | CT VS MOXI+IHM | 3.9475872 | 1.943442 | 5.951732 | 1 |
| Xu Lilii 2021 | CT VS MOXI+MT | 3.6827795 | 0.134979 | 7.23058 | 1 |
| Wan Zhihui 2025 | CT VS ACU | 0.825672 | -3.591218 | 3.939874 | 1 |
| Wan Zhihui 2025 | CT VS EHM | 2.3002865 | -1.476838 | 6.077411 | 1 |
| Wan Zhihui 2025 | CT VS IHM | 0.7616433 | -5.834048 | 6.310762 | 1 |
| Wan Zhihui 2025 | CT VS MOXI | 3.4021472 | 1.718405 | 5.085889 | 1 |
| Wan Zhihui 2025 | CT VS MOXI+ACU | 5.396544 | 2.730803 | 8.062285 | 1 |
| Wan Zhihui 2025 | CT VS MOXI+EHM | 5.2842869 | 2.851847 | 7.716727 | 1 |
| Wan Zhihui 2025 | CT VS MOXI+IHM | 3.9486491 | 2.079903 | 5.817396 | 1 |
| Wan Zhihui 2025 | CT VS MOXI+MT | 3.6924062 | 0.3741108 | 7.010702 | 1 |
| Wu Min 2017 | CT VS ACU | 0.0590207 | -3.096786 | 3.097874 | 1 |
| Wu Min 2017 | CT VS EHM | 2.9335427 | -1.035363 | 6.902449 | 1 |
| Wu Min 2017 | CT VS IHM | 0.76271925 | -6.174724 | 6.649285 | 1 |
| Wu Min 2017 | CT VS MOXI | 3.1850306 | 1.33148 | 5.038581 | 1 |
| Wu Min 2017 | CT VS MOXI+ACU | 5.3834283 | 2.532673 | 8.234184 | 1 |
| Wu Min 2017 | CT VS MOXI+EHM | 6.1881273 | 3.807761 | 8.568494 | 1 |
| Wu Min 2017 | CT VS MOXI+IHM | 3.9476122 | 1.94717 | 5.948054 | 1 |
| Wu Min 2017 | CT VS MOXI+MT | 3.6830188 | 0.1415024 | 7.224535 | 1 |
| Jin Zhu 2020 | CT VS ACU | 0.9952397 | -3.016891 | 5.026412 | 1 |
| Jin Zhu 2020 | CT VS EHM | 2.9671456 | -0.9859295 | 6.920221 | 1 |
| Jin Zhu 2020 | CT VS IHM | 0.76265316 | -6.150838 | 6.625532 | 1 |
| Jin Zhu 2020 | CT VS MOXI | 3.2477798 | 1.399549 | 5.096011 | 1 |
| Jin Zhu 2020 | CT VS MOXI+ACU | 5.3842701 | 2.546475 | 8.222065 | 1 |
| Jin Zhu 2020 | CT VS MOXI+EHM | 6.2130326 | 3.842547 | 8.583518 | 1 |
| Jin Zhu 2020 | CT VS MOXI+IHM | 3.9476755 | 1.956455 | 5.938896 | 1 |
| Jin Zhu 2020 | CT VS MOXI+MT | 3.6836201 | 0.1577532 | 7.209487 | 1 |
| Wang Yue 2022 | CT VS ACU | 0.1925058 | -3.085594 | 3.700582 | 1 |
| Wang Yue 2022 | CT VS EHM | 2.8534595 | -1.011083 | 6.718001 | 1 |
| Wang Yue 2022 | CT VS IHM | 0.76233831 | -6.042903 | 6.518226 | 1 |
| Wang Yue 2022 | CT VS MOXI | 3.0455739 | 1.345679 | 4.745469 | 1 |
| Wang Yue 2022 | CT VS MOXI+ACU | 6.4051125 | 3.076813 | 9.733412 | 1 |
| Wang Yue 2022 | CT VS MOXI+EHM | 6.1378462 | 3.825971 | 8.449721 | 1 |
| Wang Yue 2022 | CT VS MOXI+IHM | 3.9479777 | 1.998451 | 5.897505 | 1 |
| Wang Yue 2022 | CT VS MOXI+MT | 3.6864387 | 0.2312918 | 7.141586 | 1 |
| Feng Xueqiang 2024 | CT VS ACU | 0.98025641 | -3.872605 | 5.833118 | 1 |
| Feng Xueqiang 2024 | CT VS EHM | 2.8412115 | -0.8197591 | 6.502182 | 1 |
| Feng Xueqiang 2024 | CT VS IHM | 0.76129415 | -5.741774 | 6.219186 | 1 |
| Feng Xueqiang 2024 | CT VS MOXI | 3.040761 | 1.438301 | 4.643221 | 1 |
| Feng Xueqiang 2024 | CT VS MOXI+ACU | 5.4005501 | 2.785002 | 8.016098 | 1 |
| Feng Xueqiang 2024 | CT VS MOXI+EHM | 6.1452993 | 3.961234 | 8.329365 | 1 |
| Feng Xueqiang 2024 | CT VS MOXI+IHM | 3.9489881 | 2.115979 | 5.781998 | 1 |
| Feng Xueqiang 2024 | CT VS MOXI+MT | 3.6952818 | 0.4374588 | 6.953104 | 1 |
| Du Xinze 2023 | CT VS ACU | 0.1872558 | -3.967738 | 4.593227 | 1 |
| Du Xinze 2023 | CT VS EHM | 2.8473538 | -0.9115738 | 6.606281 | 1 |
| Du Xinze 2023 | CT VS IHM | 0.0949393 | -5.291699 | 5.301821 | 1 |
| Du Xinze 2023 | CT VS MOXI | 3.0432099 | 1.393855 | 4.692564 | 1 |
| Du Xinze 2023 | CT VS MOXI+ACU | 5.3943515 | 2.699978 | 8.088725 | 1 |
| Du Xinze 2023 | CT VS MOXI+EHM | 6.1416042 | 3.896034 | 8.387175 | 1 |
| Du Xinze 2023 | CT VS MOXI+IHM | 4.6154838 | 2.539142 | 6.691826 | 1 |
| Du Xinze 2023 | CT VS MOXI+MT | 3.6908341 | 0.3380375 | 7.043631 | 1 |
| Ma Mengmeng 2024 | CT VS ACU | 0.151904 | -3.225644 | 3.921835 | 1 |
| Ma Mengmeng 2024 | CT VS EHM | 2.8846572 | -1.112269 | 6.881583 | 1 |
| Ma Mengmeng 2024 | CT VS IHM | 0.76282061 | -6.212246 | 6.686605 | 1 |
| Ma Mengmeng 2024 | CT VS MOXI | 3.0937429 | 1.209041 | 4.978445 | 1 |
| Ma Mengmeng 2024 | CT VS MOXI+ACU | 5.3821274 | 2.511015 | 8.25324 | 1 |
| Ma Mengmeng 2024 | CT VS MOXI+EHM | 6.1517922 | 3.753166 | 8.550418 | 1 |
| Ma Mengmeng 2024 | CT VS MOXI+IHM | 3.9475153 | 1.93259 | 5.96244 | 1 |
| Ma Mengmeng 2024 | CT VS MOXI+MT | 3.6820899 | 0.1159894 | 7.24819 | 1 |
| Li Xiaoting 2014 | CT VS ACU | 0.196331 | -3.178321 | 3.785659 | 1 |
| Li Xiaoting 2014 | CT VS EHM | 2.8579619 | -1.089954 | 6.805878 | 1 |
| Li Xiaoting 2014 | CT VS IHM | 0.76269563 | -6.166137 | 6.640746 | 1 |
| Li Xiaoting 2014 | CT VS MOXI | 3.0473151 | 1.307561 | 4.78707 | 1 |
| Li Xiaoting 2014 | CT VS MOXI+ACU | 4.771684 | 1.246861 | 8.296507 | 1 |
| Li Xiaoting 2014 | CT VS MOXI+EHM | 6.1350569 | 3.770851 | 8.499263 | 1 |
| Li Xiaoting 2014 | CT VS MOXI+IHM | 3.9476348 | 1.950508 | 5.944762 | 1 |
| Li Xiaoting 2014 | CT VS MOXI+MT | 3.683234 | 0.1473438 | 7.219124 | 1 |
| Luo Xiaohong 2022 | CT VS ACU | 0.2189382 | -3.23382 | 3.795944 | 1 |
| Luo Xiaohong 2022 | CT VS EHM | 2.7773415 | -1.320721 | 6.875403 | 1 |
| Luo Xiaohong 2022 | CT VS IHM | 0.76278571 | -6.199203 | 6.673631 | 1 |
| Luo Xiaohong 2022 | CT VS MOXI | 3.0261353 | 1.255234 | 4.797036 | 1 |
| Luo Xiaohong 2022 | CT VS MOXI+ACU | 5.3825767 | 2.51854 | 8.246614 | 1 |
| Luo Xiaohong 2022 | CT VS MOXI+EHM | 6.0312375 | 3.330155 | 8.73232 | 1 |
| Luo Xiaohong 2022 | CT VS MOXI+IHM | 3.9475487 | 1.937657 | 5.95744 | 1 |
| Luo Xiaohong 2022 | CT VS MOXI+MT | 3.6824106 | 0.1248558 | 7.239965 | 1 |
| Wang Jiawei 2023 | CT VS ACU | 0.2053609 | -3.220253 | 3.809532 | 1 |
| Wang Jiawei 2023 | CT VS EHM | 2.8287401 | -1.266297 | 6.923777 | 1 |
| Wang Jiawei 2023 | CT VS IHM | 0.76278662 | -6.199541 | 6.673967 | 1 |
| Wang Jiawei 2023 | CT VS MOXI | 3.0397302 | 1.269238 | 4.810223 | 1 |
| Wang Jiawei 2023 | CT VS MOXI+ACU | 5.382565 | 2.518345 | 8.246785 | 1 |
| Wang Jiawei 2023 | CT VS MOXI+EHM | 6.0960075 | 3.402524 | 8.789492 | 1 |
| Wang Jiawei 2023 | CT VS MOXI+IHM | 3.9475478 | 1.937526 | 5.95757 | 1 |
| Wang Jiawei 2023 | CT VS MOXI+MT | 3.6824023 | 0.124626 | 7.240179 | 1 |
| Hu Shuhan 2026 | CT VS ACU | 0.2338548 | -3.298132 | 3.830423 | 1 |
| Hu Shuhan 2026 | CT VS EHM | 2.0757164 | -3.49477 | 7.646203 | 1 |
| Hu Shuhan 2026 | CT VS IHM | 0.76292836 | -6.25337 | 6.727513 | 1 |
| Hu Shuhan 2026 | CT VS MOXI | 3.0135085 | 1.202133 | 4.824884 | 1 |
| Hu Shuhan 2026 | CT VS MOXI+ACU | 5.3807313 | 2.487287 | 8.274176 | 1 |
| Hu Shuhan 2026 | CT VS MOXI+EHM | 6.2167439 | 3.638736 | 8.794751 | 1 |
| Hu Shuhan 2026 | CT VS MOXI+IHM | 3.9474125 | 1.916618 | 5.978207 | 1 |
| Hu Shuhan 2026 | CT VS MOXI+MT | 3.6810935 | 0.0880387 | 7.274148 | 1 |
| Peng Jing 2022 | CT VS ACU | 0.1589851 | -3.140016 | 3.822046 | 1 |
| Peng Jing 2022 | CT VS EHM | 3.7125823 | -1.442053 | 8.867218 | 1 |
| Peng Jing 2022 | CT VS IHM | 0.76268202 | -6.161212 | 6.635848 | 1 |
| Peng Jing 2022 | CT VS MOXI | 3.0844538 | 1.340299 | 4.828609 | 1 |
| Peng Jing 2022 | CT VS MOXI+ACU | 5.3839032 | 2.54048 | 8.227326 | 1 |
| Peng Jing 2022 | CT VS MOXI+EHM | 6.0473019 | 3.660248 | 8.434357 | 1 |
| Peng Jing 2022 | CT VS MOXI+IHM | 3.9476479 | 1.952422 | 5.942873 | 1 |
| Peng Jing 2022 | CT VS MOXI+MT | 3.683358 | 0.1506948 | 7.216021 | 1 |
| Liang Guoyu 2025 | CT VS ACU | 0.194959 | -3.257162 | 3.867244 | 1 |
| Liang Guoyu 2025 | CT VS EHM | 2.8606485 | -1.128907 | 6.850204 | 1 |
| Liang Guoyu 2025 | CT VS IHM | 0.76279802 | -6.203787 | 6.678192 | 1 |
| Liang Guoyu 2025 | CT VS MOXI | 3.0503166 | 1.180014 | 4.920619 | 1 |
| Liang Guoyu 2025 | CT VS MOXI+ACU | 5.3824184 | 2.515894 | 8.248942 | 1 |
| Liang Guoyu 2025 | CT VS MOXI+EHM | 6.135198 | 3.741553 | 8.528843 | 1 |
| Liang Guoyu 2025 | CT VS MOXI+IHM | 3.9475369 | 1.935876 | 5.959198 | 1 |
| Liang Guoyu 2025 | CT VS MOXI+MT | 3.6822976 | 0.1217387 | 7.242857 | 1 |
| Hu Guanghui 2023 | CT VS ACU | 0.1962904 | -3.177305 | 3.784724 | 1 |
| Hu Guanghui 2023 | CT VS EHM | 2.8579139 | -1.089087 | 6.804914 | 1 |
| Hu Guanghui 2023 | CT VS IHM | 0.98386888 | -6.452334 | 7.484597 | 1 |
| Hu Guanghui 2023 | CT VS MOXI | 3.0472965 | 1.30798 | 4.786613 | 1 |
| Hu Guanghui 2023 | CT VS MOXI+ACU | 5.3837773 | 2.538415 | 8.229139 | 1 |
| Hu Guanghui 2023 | CT VS MOXI+EHM | 6.1350867 | 3.771455 | 8.498718 | 1 |
| Hu Guanghui 2023 | CT VS MOXI+IHM | 3.7264156 | 1.556566 | 5.896265 | 1 |
| Hu Guanghui 2023 | CT VS MOXI+MT | 3.6832681 | 0.1482643 | 7.218272 | 1 |

Leave-one-out sensitivity analysis of median MNCV in the primary analysis of patients with DPN (Table S134)

| **dropped_id** | **comparison** | **eff** | **lci** | **uci** | **connected** |
| --- | --- | --- | --- | --- | --- |
| Feng Yun 2022 | CT VS ACU | 0.8655205 | -2.500559 | 2.769517 | 1 |
| Feng Yun 2022 | CT VS EHM | 1.6136974 | -4.35876 | 7.586155 | 1 |
| Feng Yun 2022 | CT VS MOXI | 2.8244766 | 0.2622505 | 5.386703 | 1 |
| Feng Yun 2022 | CT VS MOXI+ACU | 6.4700003 | 1.761725 | 11.17828 | 1 |
| Feng Yun 2022 | CT VS MOXI+EHM | 6.3642689 | 2.72279 | 10.00575 | 1 |
| Feng Yun 2022 | CT VS MOXI+IHM | 3.4231817 | 1.474965 | 5.371398 | 1 |
| Feng Yun 2022 | CT VS MOXI+MT | 4.0341784 | 0.5973642 | 7.470993 | 1 |
| Xie Lingling 2025 | CT VS ACU | 0.8739347 | -2.333765 | 3.585895 | 1 |
| Xie Lingling 2025 | CT VS EHM | 1.5917384 | -5.3262 | 8.509677 | 1 |
| Xie Lingling 2025 | CT VS MOXI | 2.8160603 | 0.1473769 | 5.779498 | 1 |
| Xie Lingling 2025 | CT VS MOXI+ACU | 6.4700003 | 0.9992669 | 11.94073 | 1 |
| Xie Lingling 2025 | CT VS MOXI+EHM | 6.3425025 | 2.136407 | 10.5486 | 1 |
| Xie Lingling 2025 | CT VS MOXI+IHM | 4.0900734 | 2.010892 | 6.169255 | 1 |
| Xie Lingling 2025 | CT VS MOXI+MT | 4.3500004 | -1.368327 | 10.06833 | 1 |
| Wang Dan 2021 | CT VS ACU | 0.8604851 | -2.22805 | 2.507082 | 1 |
| Wang Dan 2021 | CT VS EHM | 1.0954276 | -5.494725 | 7.68558 | 1 |
| Wang Dan 2021 | CT VS MOXI | 1.8293692 | 1.476706 | 5.135445 | 1 |
| Wang Dan 2021 | CT VS MOXI+ACU | 6.4700003 | 1.312762 | 11.62724 | 1 |
| Wang Dan 2021 | CT VS MOXI+EHM | 5.8460226 | 1.772501 | 9.919544 | 1 |
| Wang Dan 2021 | CT VS MOXI+IHM | 4.0949925 | 2.133736 | 6.05625 | 1 |
| Wang Dan 2021 | CT VS MOXI+MT | 4.0366707 | 0.2908142 | 7.782527 | 1 |
| Ji Hua 2025 | CT VS ACU | 0.8755635 | -2.24955 | 2.498421 | 1 |
| Ji Hua 2025 | CT VS EHM | 1.0878302 | -5.499272 | 7.674932 | 1 |
| Ji Hua 2025 | CT VS MOXI | 1.8142889 | 1.511379 | 5.139956 | 1 |
| Ji Hua 2025 | CT VS MOXI+ACU | 6.4700003 | 1.317432 | 11.62257 | 1 |
| Ji Hua 2025 | CT VS MOXI+EHM | 5.8384228 | 1.763924 | 9.912921 | 1 |
| Ji Hua 2025 | CT VS MOXI+IHM | 4.0950731 | 2.135567 | 6.054579 | 1 |
| Ji Hua 2025 | CT VS MOXI+MT | 4.0366478 | 0.294012 | 7.779284 | 1 |
| Tang Huazhen 2022 | CT VS ACU | 0.8739347 | -2.333765 | 3.585895 | 1 |
| Tang Huazhen 2022 | CT VS EHM | 1.5917384 | -5.3262 | 8.509677 | 1 |
| Tang Huazhen 2022 | CT VS MOXI | 2.8160603 | 0.1473769 | 5.779498 | 1 |
| Tang Huazhen 2022 | CT VS MOXI+ACU | 6.4700003 | 0.9992668 | 11.94073 | 1 |
| Tang Huazhen 2022 | CT VS MOXI+EHM | 6.3425025 | 2.136407 | 10.5486 | 1 |
| Tang Huazhen 2022 | CT VS MOXI+IHM | 4.0900734 | 2.010892 | 6.169255 | 1 |
| Tang Huazhen 2022 | CT VS MOXI+MT | 3.7499999 | -1.745508 | 9.245508 | 1 |
| Zhang Xiujun 2020 | CT VS ACU | 0.8738164 | -2.319235 | 3.571602 | 1 |
| Zhang Xiujun 2020 | CT VS EHM | 1.5920486 | -5.309449 | 8.493546 | 1 |
| Zhang Xiujun 2020 | CT VS MOXI | 2.8161786 | 0.1402771 | 5.772635 | 1 |
| Zhang Xiujun 2020 | CT VS MOXI+ACU | 6.4700003 | 1.012508 | 11.92749 | 1 |
| Zhang Xiujun 2020 | CT VS MOXI+EHM | 6.3428091 | 2.146544 | 10.53907 | 1 |
| Zhang Xiujun 2020 | CT VS MOXI+IHM | 4.2264742 | 1.984623 | 6.468326 | 1 |
| Zhang Xiujun 2020 | CT VS MOXI+MT | 4.0380277 | 0.0848258 | 7.99123 | 1 |
| Xu Xiaowei 2025 | CT VS ACU | 0.8961197 | -2.59075 | 3.798511 | 1 |
| Xu Xiaowei 2025 | CT VS EHM | 5.4392689 | -1.763171 | 12.64171 | 1 |
| Xu Xiaowei 2025 | CT VS MOXI | 3.7939942 | 1.046491 | 6.541497 | 1 |
| Xu Xiaowei 2025 | CT VS MOXI+ACU | 6.4700003 | 1.794594 | 11.14541 | 1 |
| Xu Xiaowei 2025 | CT VS MOXI+EHM | 10.190373 | 4.733426 | 15.64732 | 1 |
| Xu Xiaowei 2025 | CT VS MOXI+IHM | 4.1047293 | 2.324565 | 5.884893 | 1 |
| Xu Xiaowei 2025 | CT VS MOXI+MT | 4.0339696 | 0.6197297 | 7.44821 | 1 |
| Zhang Suzhao 2024 | CT VS ACU | 0.8739396 | -2.334371 | 3.586492 | 1 |
| Zhang Suzhao 2024 | CT VS EHM | 1.5917255 | -5.326899 | 8.510349 | 1 |
| Zhang Suzhao 2024 | CT VS MOXI | 2.8160554 | 0.1476729 | 5.779784 | 1 |
| Zhang Suzhao 2024 | CT VS MOXI+ACU | 6.4700003 | 0.9987145 | 11.94129 | 1 |
| Zhang Suzhao 2024 | CT VS MOXI+EHM | 6.3424898 | 2.135984 | 10.549 | 1 |
| Zhang Suzhao 2024 | CT VS MOXI+IHM | 3.9566904 | 1.704408 | 6.208972 | 1 |
| Zhang Suzhao 2024 | CT VS MOXI+MT | 4.0380851 | 0.0753469 | 8.000823 | 1 |
| Zhang Xiujun 2020 | CT VS ACU | 0.874091 | -2.353119 | 3.604937 | 1 |
| Zhang Xiujun 2020 | CT VS EHM | 1.5913284 | -5.34851 | 8.531167 | 1 |
| Zhang Xiujun 2020 | CT VS MOXI | 2.815904 | 0.1568326 | 5.78864 | 1 |
| Zhang Xiujun 2020 | CT VS MOXI+ACU | 6.4700003 | 0.9816321 | 11.95837 | 1 |
| Zhang Xiujun 2020 | CT VS MOXI+EHM | 6.3420974 | 2.122904 | 10.56129 | 1 |
| Zhang Xiujun 2020 | CT VS MOXI+IHM | 4.1498598 | 1.892053 | 6.407667 | 1 |
| Zhang Xiujun 2020 | CT VS MOXI+MT | 4.0381556 | 0.0636055 | 8.012706 | 1 |
| Hu Guanghui 2024 | CT VS ACU | 0.8623428 | -2.263988 | 2.539302 | 1 |
| Hu Guanghui 2024 | CT VS EHM | 1.6219394 | -4.080955 | 7.324834 | 1 |
| Hu Guanghui 2024 | CT VS MOXI | 2.8276548 | 0.3796995 | 5.27561 | 1 |
| Hu Guanghui 2024 | CT VS MOXI+ACU | 6.4700003 | 1.979567 | 10.96043 | 1 |
| Hu Guanghui 2024 | CT VS MOXI+EHM | 6.3724611 | 2.891645 | 9.853277 | 1 |
| Hu Guanghui 2024 | CT VS MOXI+IHM | 4.840433 | 2.998304 | 6.682562 | 1 |
| Hu Guanghui 2024 | CT VS MOXI+MT | 4.0327163 | 0.7453557 | 7.320077 | 1 |
| Liu Xiangxiu 2012 | CT VS ACU | 0.873994 | -2.34109 | 3.593102 | 1 |
| Liu Xiangxiu 2012 | CT VS EHM | 1.5915828 | -5.334645 | 8.517811 | 1 |
| Liu Xiangxiu 2012 | CT VS MOXI | 2.8160009 | 0.150956 | 5.782958 | 1 |
| Liu Xiangxiu 2012 | CT VS MOXI+ACU | 6.4700003 | 0.992592 | 11.94741 | 1 |
| Liu Xiangxiu 2012 | CT VS MOXI+EHM | 6.3423487 | 2.131295 | 10.5534 | 1 |
| Liu Xiangxiu 2012 | CT VS MOXI+IHM | 4.1202977 | 1.878606 | 6.361989 | 1 |
| Liu Xiangxiu 2012 | CT VS MOXI+MT | 4.0381104 | 0.0711389 | 8.005082 | 1 |
| Xu Lilii 2021 | CT VS ACU | 0.871377 | -2.040478 | 3.297724 | 1 |
| Xu Lilii 2021 | CT VS EHM | 1.5984363 | -4.987285 | 8.184157 | 1 |
| Xu Lilii 2021 | CT VS MOXI | 2.8186188 | 0.0037834 | 5.641021 | 1 |
| Xu Lilii 2021 | CT VS MOXI+ACU | 6.3491296 | 2.341653 | 10.35661 | 1 |
| Xu Lilii 2021 | CT VS MOXI+EHM | 4.094213 | 2.11574 | 6.072686 | 1 |
| Xu Lilii 2021 | CT VS MOXI+IHM | 4.0368923 | 0.2593873 | 7.814397 | 1 |
| Wan Zhihui 2025 | CT VS ACU | 0.8944036 | -2.589345 | 3.800538 | 1 |
| Wan Zhihui 2025 | CT VS EHM | 1.3804358 | -5.119424 | 8.358552 | 1 |
| Wan Zhihui 2025 | CT VS MOXI | 3.7957105 | 1.047817 | 6.543604 | 1 |
| Wan Zhihui 2025 | CT VS MOXI+ACU | 6.4700003 | 1.794444 | 11.14556 | 1 |
| Wan Zhihui 2025 | CT VS MOXI+EHM | 3.3697048 | 1.458746 | 8.198155 | 1 |
| Wan Zhihui 2025 | CT VS MOXI+IHM | 4.1047258 | 2.324505 | 5.884946 | 1 |
| Wan Zhihui 2025 | CT VS MOXI+MT | 4.0339706 | 0.6196275 | 7.448314 | 1 |
| Jin Zhu 2020 | CT VS ACU | 0.0563761 | -4.526516 | 4.613764 | 1 |
| Jin Zhu 2020 | CT VS EHM | 2.0124372 | -4.693877 | 8.718751 | 1 |
| Jin Zhu 2020 | CT VS MOXI | 3.6337399 | 0.2759885 | 6.991491 | 1 |
| Jin Zhu 2020 | CT VS MOXI+ACU | 6.4700003 | 1.219299 | 11.7207 | 1 |
| Jin Zhu 2020 | CT VS MOXI+EHM | 6.7632165 | 2.620041 | 10.90639 | 1 |
| Jin Zhu 2020 | CT VS MOXI+IHM | 4.0934273 | 2.097017 | 6.089838 | 1 |
| Jin Zhu 2020 | CT VS MOXI+MT | 4.0371164 | 0.2267694 | 7.847463 | 1 |
| Feng Xueqiang 2024 | ACU VS CT | 1.5984329 | -4.98741 | 8.184276 | 1 |
| Feng Xueqiang 2024 | ACU VS EHM | 2.8186159 | -0.0039402 | 5.641172 | 1 |
| Feng Xueqiang 2024 | ACU VS MOXI | 6.4700003 | 1.266796 | 11.67321 | 1 |
| Feng Xueqiang 2024 | ACU VS MOXI+ACU | 6.3491262 | 2.341564 | 10.35669 | 1 |
| Feng Xueqiang 2024 | ACU VS MOXI+EHM | 4.0942115 | 2.115705 | 6.072718 | 1 |
| Feng Xueqiang 2024 | ACU VS MOXI+IHM | 4.0368927 | 0.2593265 | 7.814459 | 1 |
| Peng Jing 2022 | CT VS ACU | 0.8711732 | -3.040439 | 3.298092 | 1 |
| Peng Jing 2022 | CT VS EHM | 2.8188225 | -0.0036877 | 5.641333 | 1 |
| Peng Jing 2022 | CT VS MOXI | 6.4700003 | 1.266747 | 11.67325 | 1 |
| Peng Jing 2022 | CT VS MOXI+ACU | 6.3499378 | 2.341961 | 10.35791 | 1 |
| Peng Jing 2022 | CT VS MOXI+EHM | 4.0942107 | 2.115686 | 6.072735 | 1 |
| Peng Jing 2022 | CT VS MOXI+IHM | 4.0368929 | 0.2592931 | 7.814493 | 1 |
| Hu Guanghui 2023 | CT VS ACU | 0.8735461 | -3.286404 | 3.539312 | 1 |
| Hu Guanghui 2023 | CT VS EHM | 1.5927573 | -5.271579 | 8.457094 | 1 |
| Hu Guanghui 2023 | CT VS MOXI | 2.816449 | 0.1242283 | 5.757126 | 1 |
| Hu Guanghui 2023 | CT VS MOXI+ACU | 6.4700003 | 1.042433 | 11.89757 | 1 |
| Hu Guanghui 2023 | CT VS MOXI+EHM | 6.3435098 | 2.169468 | 10.51755 | 1 |
| Hu Guanghui 2023 | CT VS MOXI+IHM | 3.9201563 | 1.703976 | 6.136336 | 1 |
| Hu Guanghui 2023 | CT VS MOXI+MT | 4.0379018 | 0.1053868 | 7.970417 | 1 |

Leave-one-out sensitivity analysis of common peroneal SNCV in the primary analysis of patients with DPN (Table S135)

| **dropped_id** | **comparison** | **eff** | **lci** | **uci** | **connected** |
| --- | --- | --- | --- | --- | --- |
| Feng Yun 2022 | CT VS ACU | 3.677772 | -1.387158 | 8.742702 | 1 |
| Feng Yun 2022 | CT VS EHM | 4.6088137 | -0.8414733 | 8.376154 | 1 |
| Feng Yun 2022 | CT VS IHM | 0.65717099 | -4.911163 | 5.596821 | 1 |
| Feng Yun 2022 | CT VS MOXI | 2.8338401 | 1.399643 | 4.268037 | 1 |
| Feng Yun 2022 | CT VS MOXI+ACU | 6.0085594 | 2.522833 | 9.494286 | 1 |
| Feng Yun 2022 | CT VS MOXI+EHM | 8.4852307 | 5.71245 | 11.25801 | 1 |
| Feng Yun 2022 | CT VS MOXI+IHM | 3.0129583 | 0.9717649 | 5.054152 | 1 |
| Feng Yun 2022 | CT VS MOXI+MT | 3.9699998 | -0.0525998 | 7.8874 | 1 |
| Wang Dan 2021 | CT VS ACU | 3.6756599 | -3.353775 | 10.7051 | 1 |
| Wang Dan 2021 | CT VS EHM | 4.4304061 | -0.7396902 | 9.600502 | 1 |
| Wang Dan 2021 | CT VS IHM | 0.90266113 | -4.740749 | 6.546071 | 1 |
| Wang Dan 2021 | CT VS MOXI | 2.6101922 | 0.4710947 | 4.74929 | 1 |
| Wang Dan 2021 | CT VS MOXI+ACU | 6.0071513 | 1.104951 | 10.90935 | 1 |
| Wang Dan 2021 | CT VS MOXI+EHM | 8.309776 | 4.53013 | 12.08942 | 1 |
| Wang Dan 2021 | CT VS MOXI+IHM | 4.5732299 | 2.117682 | 7.028778 | 1 |
| Wang Dan 2021 | CT VS MOXI+MT | 3.9699998 | -1.248987 | 9.188987 | 1 |
| Ji Hua 2025 | CT VS ACU | 3.6756004 | -3.401242 | 10.75244 | 1 |
| Ji Hua 2025 | CT VS EHM | 4.4526658 | -0.751709 | 9.657041 | 1 |
| Ji Hua 2025 | CT VS IHM | 0.90173347 | -4.778617 | 6.582084 | 1 |
| Ji Hua 2025 | CT VS MOXI | 2.6548012 | 0.4961907 | 4.813412 | 1 |
| Ji Hua 2025 | CT VS MOXI+ACU | 6.0071117 | 1.070919 | 10.9433 | 1 |
| Ji Hua 2025 | CT VS MOXI+EHM | 8.3320963 | 4.527599 | 12.13659 | 1 |
| Ji Hua 2025 | CT VS MOXI+IHM | 4.5723096 | 2.099792 | 7.044828 | 1 |
| Ji Hua 2025 | CT VS MOXI+MT | 3.9699998 | -1.280966 | 9.220966 | 1 |
| Bai Fubin 2025 | CT VS ACU | 3.675925 | -3.138054 | 10.4899 | 1 |
| Bai Fubin 2025 | CT VS EHM | 4.5604512 | -0.439135 | 9.560038 | 1 |
| Bai Fubin 2025 | CT VS IHM | 2.851354 | -0.9369706 | 4.765738 | 1 |
| Bai Fubin 2025 | CT VS MOXI | 6.0073281 | 1.259697 | 10.75496 | 1 |
| Bai Fubin 2025 | CT VS MOXI+ACU | 8.4396003 | 4.793063 | 12.08614 | 1 |
| Bai Fubin 2025 | CT VS MOXI+EHM | 4.5779246 | 2.1995 | 6.956349 | 1 |
| Bai Fubin 2025 | CT VS MOXI+IHM | 3.9699998 | 1.10391 | 9.04391 | 1 |
| Tang Huazhen 2022 | CT VS ACU | 3.6759252 | -3.137907 | 10.48976 | 1 |
| Tang Huazhen 2022 | CT VS EHM | 4.5604541 | -0.4390278 | 9.559936 | 1 |
| Tang Huazhen 2022 | CT VS IHM | 0.90713674 | -4.568339 | 6.382612 | 1 |
| Tang Huazhen 2022 | CT VS MOXI | 2.8513531 | 0.9370102 | 4.765696 | 1 |
| Tang Huazhen 2022 | CT VS MOXI+ACU | 6.0073282 | 1.259803 | 10.75485 | 1 |
| Tang Huazhen 2022 | CT VS MOXI+EHM | 8.439603 | 4.793139 | 12.08607 | 1 |
| Tang Huazhen 2022 | CT VS MOXI+IHM | 4.5776727 | 2.199405 | 6.95594 | 1 |
| Zhang Suzhao 2024 | CT VS ACU | 3.6758236 | -3.221332 | 10.57298 | 1 |
| Zhang Suzhao 2024 | CT VS EHM | 4.5588683 | -0.49967 | 9.617407 | 1 |
| Zhang Suzhao 2024 | CT VS IHM | 0.26568851 | -5.446187 | 5.977564 | 1 |
| Zhang Suzhao 2024 | CT VS MOXI | 2.8518655 | 0.9146038 | 4.789127 | 1 |
| Zhang Suzhao 2024 | CT VS MOXI+ACU | 6.0072605 | 1.199941 | 10.81458 | 1 |
| Zhang Suzhao 2024 | CT VS MOXI+EHM | 8.4381171 | 4.749614 | 12.12662 | 1 |
| Zhang Suzhao 2024 | CT VS MOXI+IHM | 3.9361089 | 1.155843 | 6.716375 | 1 |
| Zhang Suzhao 2024 | CT VS MOXI+MT | 3.9699998 | -1.159865 | 9.099864 | 1 |
| Hu Guanghui 2024 | CT VS ACU | 3.6769011 | -2.276991 | 9.630794 | 1 |
| Hu Guanghui 2024 | CT VS EHM | 4.57998 | -0.1881444 | 8.971816 | 1 |
| Hu Guanghui 2024 | CT VS IHM | 2.1413674 | -2.809247 | 7.091981 | 1 |
| Hu Guanghui 2024 | CT VS MOXI | 2.8446963 | 1.16659 | 4.522803 | 1 |
| Hu Guanghui 2024 | CT VS MOXI+ACU | 6.0079788 | 1.878997 | 10.13696 | 1 |
| Hu Guanghui 2024 | CT VS MOXI+EHM | 8.4579747 | 5.243193 | 11.67276 | 1 |
| Hu Guanghui 2024 | CT VS MOXI+IHM | 5.811965 | 3.42906 | 8.19487 | 1 |
| Hu Guanghui 2024 | CT VS MOXI+MT | 3.9699998 | -0.5297474 | 8.469747 | 1 |
| Xu Lilii 2021 | —— | —— | —— | —— | disconnected |
| Wan Zhihui 2025 | CT VS ACU | 3.6759022 | -3.156853 | 10.50866 | 1 |
| Wan Zhihui 2025 | CT VS EHM | 2.7959491 | -3.334255 | 8.926153 | 1 |
| Wan Zhihui 2025 | CT VS IHM | 0.90672618 | -4.583512 | 6.396965 | 1 |
| Wan Zhihui 2025 | CT VS MOXI | 3.1239117 | 1.128555 | 5.119268 | 1 |
| Wan Zhihui 2025 | CT VS MOXI+ACU | 6.0073129 | 1.246205 | 10.76842 | 1 |
| Wan Zhihui 2025 | CT VS MOXI+EHM | 6.6746687 | 1.592991 | 11.75635 | 1 |
| Wan Zhihui 2025 | CT VS MOXI+IHM | 4.577265 | 2.192145 | 6.962385 | 1 |
| Wan Zhihui 2025 | CT VS MOXI+MT | 3.9699998 | -1.116536 | 9.056535 | 1 |
| Du Minzhen 2019 | CT VS ACU | 3.6755455 | -3.444851 | 10.79594 | 1 |
| Du Minzhen 2019 | CT VS EHM | 4.6357814 | -0.6003206 | 9.871883 | 1 |
| Du Minzhen 2019 | CT VS IHM | 0.90089858 | -4.813397 | 6.615195 | 1 |
| Du Minzhen 2019 | CT VS MOXI | 3.0090103 | 0.831423 | 5.186597 | 1 |
| Du Minzhen 2019 | CT VS MOXI+ACU | 6.0070751 | 1.039659 | 10.97449 | 1 |
| Du Minzhen 2019 | CT VS MOXI+EHM | 8.5153125 | 4.687674 | 12.34295 | 1 |
| Du Minzhen 2019 | CT VS MOXI+IHM | 4.5714815 | 2.083377 | 7.059587 | 1 |
| Du Minzhen 2019 | CT VS MOXI+MT | 3.9699998 | -1.310365 | 9.250364 | 1 |
| Jin Zhu 2020 | CT VS ACU | 3.6755628 | -3.431139 | 10.78226 | 1 |
| Jin Zhu 2020 | CT VS EHM | 4.4974047 | -0.7265713 | 9.72138 | 1 |
| Jin Zhu 2020 | CT VS IHM | 0.90115898 | -4.802463 | 6.604781 | 1 |
| Jin Zhu 2020 | CT VS MOXI | 2.7420467 | 0.5898249 | 4.894269 | 1 |
| Jin Zhu 2020 | CT VS MOXI+ACU | 6.0070865 | 1.049487 | 10.96469 | 1 |
| Jin Zhu 2020 | CT VS MOXI+EHM | 8.3768817 | 4.559464 | 12.1943 | 1 |
| Jin Zhu 2020 | CT VS MOXI+IHM | 4.5717397 | 2.088536 | 7.054943 | 1 |
| Jin Zhu 2020 | CT VS MOXI+MT | 3.9699998 | -1.301119 | 9.241118 | 1 |
| Luo Xingzi 2020 | CT VS ACU | 4.5604469 | -0.4392968 | 9.560191 | 1 |
| Luo Xingzi 2020 | CT VS EHM | 0.90712868 | -4.568635 | 6.382892 | 1 |
| Luo Xingzi 2020 | CT VS IHM | 2.8513554 | -0.9369109 | 4.7658 | 1 |
| Luo Xingzi 2020 | CT VS MOXI | 6.0100002 | 1.2609 | 10.7591 | 1 |
| Luo Xingzi 2020 | CT VS MOXI+ACU | 8.4395963 | 4.792946 | 12.08625 | 1 |
| Luo Xingzi 2020 | CT VS MOXI+EHM | 4.5776647 | 2.199265 | 6.956065 | 1 |
| Luo Xingzi 2020 | CT VS MOXI+IHM | 3.9699998 | 1.104059 | 9.044059 | 1 |
| Ma Mengmeng 2024 | CT VS ACU | 3.6755078 | -3.474609 | 10.82563 | 1 |
| Ma Mengmeng 2024 | CT VS EHM | 4.6258108 | -0.6329792 | 9.884601 | 1 |
| Ma Mengmeng 2024 | CT VS IHM | 0.90033764 | -4.837126 | 6.637802 | 1 |
| Ma Mengmeng 2024 | CT VS MOXI | 2.9910066 | 0.791306 | 5.190707 | 1 |
| Ma Mengmeng 2024 | CT VS MOXI+ACU | 6.0070499 | 1.01833 | 10.99577 | 1 |
| Ma Mengmeng 2024 | CT VS MOXI+EHM | 8.5053733 | 4.660522 | 12.35022 | 1 |
| Ma Mengmeng 2024 | CT VS MOXI+IHM | 4.5709252 | 2.072184 | 7.069667 | 1 |
| Ma Mengmeng 2024 | CT VS MOXI+MT | 3.9699998 | -1.330435 | 9.270434 | 1 |
| Wang Jiawei 2023 | CT VS ACU | 3.6759026 | -3.156564 | 10.50837 | 1 |
| Wang Jiawei 2023 | CT VS EHM | 6.4420739 | -0.16394 | 12.72021 | 1 |
| Wang Jiawei 2023 | CT VS IHM | 0.90673243 | -4.583281 | 6.396746 | 1 |
| Wang Jiawei 2023 | CT VS MOXI | 3.1219317 | 1.126934 | 5.116929 | 1 |
| Wang Jiawei 2023 | CT VS MOXI+ACU | 6.0073131 | 1.246413 | 10.76821 | 1 |
| Wang Jiawei 2023 | CT VS MOXI+EHM | 10.321728 | 5.062343 | 15.58111 | 1 |
| Wang Jiawei 2023 | CT VS MOXI+IHM | 4.5772712 | 2.192255 | 6.962288 | 1 |
| Wang Jiawei 2023 | CT VS MOXI+MT | 3.9699998 | -1.116341 | 9.056341 | 1 |
| Liu Maomao 2023 | CT VS ACU | 3.676405 | -2.729045 | 10.08185 | 1 |
| Liu Maomao 2023 | CT VS EHM | 4.5689429 | -0.1416304 | 9.279516 | 1 |
| Liu Maomao 2023 | CT VS IHM | 1.8919236 | -3.419071 | 7.202919 | 1 |
| Liu Maomao 2023 | CT VS MOXI | 2.8485348 | 1.046418 | 4.650651 | 1 |
| Liu Maomao 2023 | CT VS MOXI+ACU | 6.0076481 | 1.553511 | 10.46179 | 1 |
| Liu Maomao 2023 | CT VS MOXI+EHM | 8.4475754 | 5.006515 | 11.88864 | 1 |
| Liu Maomao 2023 | CT VS MOXI+IHM | 5.5625694 | 2.996639 | 8.1285 | 1 |
| Liu Maomao 2023 | CT VS MOXI+MT | 3.9699998 | -0.8301142 | 8.770114 | 1 |
| Huang Xingduan 2024 | CT VS ACU | 3.6754985 | -3.481946 | 10.83294 | 1 |
| Huang Xingduan 2024 | CT VS EHM | 4.805044 | -1.533093 | 11.14318 | 1 |
| Huang Xingduan 2024 | CT VS IHM | 0.90020046 | -4.842975 | 6.643376 | 1 |
| Huang Xingduan 2024 | CT VS MOXI | 2.8537241 | 0.8448386 | 4.86261 | 1 |
| Huang Xingduan 2024 | CT VS MOXI+ACU | 6.0070437 | 1.013072 | 11.00101 | 1 |
| Huang Xingduan 2024 | CT VS MOXI+EHM | 8.4363662 | 4.615739 | 12.25699 | 1 |
| Huang Xingduan 2024 | CT VS MOXI+IHM | 4.5707892 | 2.069426 | 7.072153 | 1 |
| Huang Xingduan 2024 | CT VS MOXI+MT | 3.9699998 | -1.335384 | 9.275383 | 1 |
| Peng Jing 2022 | CT VS ACU | 3.6754987 | -3.481772 | 10.83277 | 1 |
| Peng Jing 2022 | CT VS EHM | 4.30296 | -2.071234 | 10.67715 | 1 |
| Peng Jing 2022 | CT VS IHM | 0.9002037 | -4.842837 | 6.643244 | 1 |
| Peng Jing 2022 | CT VS MOXI | 2.8535663 | 0.8447431 | 4.86239 | 1 |
| Peng Jing 2022 | CT VS MOXI+ACU | 6.0070439 | 1.013197 | 11.00089 | 1 |
| Peng Jing 2022 | CT VS MOXI+EHM | 8.4352767 | 4.615069 | 12.25548 | 1 |
| Peng Jing 2022 | CT VS MOXI+IHM | 4.5707924 | 2.069491 | 7.072094 | 1 |
| Peng Jing 2022 | CT VS MOXI+MT | 3.9699998 | -1.335267 | 9.275267 | 1 |
| Liang Guoyu 2025 | CT VS ACU | 3.6754928 | -3.486482 | 10.83747 | 1 |
| Liang Guoyu 2025 | CT VS EHM | 4.5360596 | -0.7294959 | 9.801615 | 1 |
| Liang Guoyu 2025 | CT VS IHM | 0.90011586 | -4.846591 | 6.646823 | 1 |
| Liang Guoyu 2025 | CT VS MOXI | 2.8185886 | 0.6291481 | 5.008029 | 1 |
| Liang Guoyu 2025 | CT VS MOXI+ACU | 6.0070399 | 1.009821 | 11.00426 | 1 |
| Liang Guoyu 2025 | CT VS MOXI+EHM | 8.4156104 | 4.56701 | 12.26421 | 1 |
| Liang Guoyu 2025 | CT VS MOXI+IHM | 4.5707053 | 2.067721 | 7.07369 | 1 |
| Liang Guoyu 2025 | CT VS MOXI+MT | 3.9699998 | -1.338444 | 9.278443 | 1 |

Leave-one-out sensitivity analysis of median SNCV in the primary analysis of patients with DPN (Table S136)

| **dropped_id** | **comparison** | **eff** | **lci** | **uci** | **connected** |
| --- | --- | --- | --- | --- | --- |
| Feng Yun 2022 | CT VS ACU | 0.3695077 | -2.505438 | 2.766422 | 1 |
| Feng Yun 2022 | CT VS EHM | 0.3265738 | -3.486725 | 4.833577 | 1 |
| Feng Yun 2022 | CT VS MOXI | 3.2904874 | 1.235431 | 5.345543 | 1 |
| Feng Yun 2022 | CT VS MOXI+ACU | 6.6100007 | 2.296026 | 10.92397 | 1 |
| Feng Yun 2022 | CT VS MOXI+EHM | 4.3482708 | 1.609798 | 7.086744 | 1 |
| Feng Yun 2022 | CT VS MOXI+IHM | 3.2040909 | 1.503592 | 4.90459 | 1 |
| Wang Dan 2021 | CT VS ACU | 0.7391652 | -2.25217 | 3.7738398 | 1 |
| Wang Dan 2021 | CT VS EHM | 0.8137071 | -4.217773 | 6.590359 | 1 |
| Wang Dan 2021 | CT VS MOXI | 1.9206507 | -0.524847 | 4.366148 | 1 |
| Wang Dan 2021 | CT VS MOXI+ACU | 6.6100006 | 2.041689 | 11.17831 | 1 |
| Wang Dan 2021 | CT VS MOXI+EHM | 3.8616469 | 0.953077 | 6.770217 | 1 |
| Wang Dan 2021 | CT VS MOXI+IHM | 4.0602237 | 2.394089 | 5.726359 | 1 |
| Zhang Xiujun 2020 | CT VS ACU | 0.375185 | -2.723734 | 2.973364 | 1 |
| Zhang Xiujun 2020 | CT VS EHM | 0.36276032 | -3.571831 | 4.84631 | 1 |
| Zhang Xiujun 2020 | CT VS MOXI | 3.2848068 | 0.6999747 | 5.869639 | 1 |
| Zhang Xiujun 2020 | CT VS MOXI+ACU | 6.6100006 | 1.125325 | 12.09468 | 1 |
| Zhang Xiujun 2020 | CT VS MOXI+EHM | 4.314388 | 0.9089531 | 7.719823 | 1 |
| Zhang Xiujun 2020 | CT VS MOXI+IHM | 4.200246 | 2.089668 | 6.310824 | 1 |
| Xu Xiaowei 2025 | CT VS ACU | 0.3306874 | -2.325801 | 3.064426 | 1 |
| Xu Xiaowei 2025 | CT VS EHM | 0.87056733 | -4.773122 | 6.514257 | 1 |
| Xu Xiaowei 2025 | CT VS MOXI | 3.5293462 | 0.9711974 | 6.087495 | 1 |
| Xu Xiaowei 2025 | CT VS MOXI+ACU | 6.6100006 | 1.290811 | 11.92919 | 1 |
| Xu Xiaowei 2025 | CT VS MOXI+EHM | 5.5478602 | 1.399335 | 9.696385 | 1 |
| Xu Xiaowei 2025 | CT VS MOXI+IHM | 4.0444595 | 2.119684 | 5.969235 | 1 |
| Zhang Suzhao 2024 | CT VS ACU | 0.3752877 | -2.753913 | 3.003338 | 1 |
| Zhang Suzhao 2024 | CT VS EHM | 0.36340324 | -3.598331 | 4.871525 | 1 |
| Zhang Suzhao 2024 | CT VS MOXI | 3.284704 | 0.6868361 | 5.882572 | 1 |
| Zhang Suzhao 2024 | CT VS MOXI+ACU | 6.6100006 | 1.096653 | 12.12335 | 1 |
| Zhang Suzhao 2024 | CT VS MOXI+EHM | 4.3137871 | 0.8918608 | 7.735713 | 1 |
| Zhang Suzhao 2024 | CT VS MOXI+IHM | 4.1746434 | 2.04046 | 6.308827 | 1 |
| Zhang Xiujun 2020 | CT VS ACU | 0.375349 | -2.772131 | 3.021433 | 1 |
| Zhang Xiujun 2020 | CT VS EHM | 0.36378706 | -3.614321 | 4.886747 | 1 |
| Zhang Xiujun 2020 | CT VS MOXI | 3.2846426 | 0.6789071 | 5.890378 | 1 |
| Zhang Xiujun 2020 | CT VS MOXI+ACU | 6.6100006 | 1.079351 | 12.14065 | 1 |
| Zhang Xiujun 2020 | CT VS MOXI+EHM | 4.3134284 | 0.8815488 | 7.745308 | 1 |
| Zhang Xiujun 2020 | CT VS MOXI+IHM | 4.0358366 | 1.90526 | 6.166414 | 1 |
| Hu Guanghui 2024 | CT VS ACU | 0.3736798 | -2.323058 | 2.575699 | 1 |
| Hu Guanghui 2024 | CT VS EHM | 0.35328596 | -3.218557 | 4.511985 | 1 |
| Hu Guanghui 2024 | CT VS MOXI | 3.2863132 | 0.8748965 | 5.69773 | 1 |
| Hu Guanghui 2024 | CT VS MOXI+ACU | 6.6100006 | 1.507267 | 11.71273 | 1 |
| Hu Guanghui 2024 | CT VS MOXI+EHM | 4.32325 | 1.136883 | 7.509616 | 1 |
| Hu Guanghui 2024 | CT VS MOXI+IHM | 4.5630433 | 2.58317 | 6.542916 | 1 |
| Liu Xiangxiu 2012 | CT VS ACU | 0.3753303 | -2.766547 | 3.015887 | 1 |
| Liu Xiangxiu 2012 | CT VS EHM | 0.36366975 | -3.60942 | 4.882081 | 1 |
| Liu Xiangxiu 2012 | CT VS MOXI | 3.2846613 | 0.6813372 | 5.887986 | 1 |
| Liu Xiangxiu 2012 | CT VS MOXI+ACU | 6.6100006 | 1.084654 | 12.13535 | 1 |
| Liu Xiangxiu 2012 | CT VS MOXI+EHM | 4.3135381 | 0.8847098 | 7.742366 | 1 |
| Liu Xiangxiu 2012 | CT VS MOXI+IHM | 3.9472972 | 1.813566 | 6.081028 | 1 |
| Xu Lilii 2021 | CT VS ACU | 0.3745686 | -2.55066 | 2.801523 | 1 |
| Xu Lilii 2021 | CT VS EHM | 0.3588919 | -3.419554 | 4.70177 | 1 |
| Xu Lilii 2021 | CT VS MOXI | 3.2854237 | 0.7754186 | 5.795429 | 1 |
| Xu Lilii 2021 | CT VS MOXI+ACU | 4.3180049 | 1.007196 | 7.628814 | 1 |
| Xu Lilii 2021 | CT VS MOXI+EHM | 4.0444461 | 2.119418 | 5.969474 | 1 |
| Wan Zhihui 2025 | CT VS ACU | 0.4740757 | -2.243106 | 3.294955 | 1 |
| Wan Zhihui 2025 | CT VS EHM | 0.2802427 | -3.332952 | 3.772466 | 1 |
| Wan Zhihui 2025 | CT VS MOXI | 4.1860481 | 1.707205 | 6.664891 | 1 |
| Wan Zhihui 2025 | CT VS MOXI+ACU | 6.6100006 | 1.752599 | 11.4674 | 1 |
| Wan Zhihui 2025 | CT VS MOXI+EHM | 2.3953137 | -1.242695 | 6.033322 | 1 |
| Wan Zhihui 2025 | CT VS MOXI+IHM | 4.053259 | 2.287794 | 5.818725 | 1 |
| Du Minzhen 2019 | CT VS ACU | 0.354924 | -0.905393 | 3.195545 | 1 |
| Du Minzhen 2019 | CT VS EHM | 0.35694914 | -3.636694 | 4.922795 | 1 |
| Du Minzhen 2019 | CT VS MOXI | 3.3050713 | 0.358978 | 6.251164 | 1 |
| Du Minzhen 2019 | CT VS MOXI+ACU | 6.6100006 | 1.070234 | 12.14977 | 1 |
| Du Minzhen 2019 | CT VS MOXI+EHM | 4.320282 | 0.8511114 | 7.789453 | 1 |
| Du Minzhen 2019 | CT VS MOXI+IHM | 4.0410292 | 2.039938 | 6.04212 | 1 |
| Jin Zhu 2020 | CT VS ACU | 0.2517294 | -2.7982 | 3.294741 | 1 |
| Jin Zhu 2020 | CT VS EHM | 0.32132308 | -3.596322 | 4.953675 | 1 |
| Jin Zhu 2020 | CT VS MOXI | 3.4082846 | 0.4605857 | 6.355984 | 1 |
| Jin Zhu 2020 | CT VS MOXI+ACU | 6.6100006 | 1.075819 | 12.14418 | 1 |
| Jin Zhu 2020 | CT VS MOXI+EHM | 4.3559124 | 0.889513 | 7.822312 | 1 |
| Jin Zhu 2020 | CT VS MOXI+IHM | 4.0411109 | 2.041954 | 6.040268 | 1 |
| Li Jianshu 2025 | CT VS ACU | 0.204676 | -2.503674 | 3.094321 | 1 |
| Li Jianshu 2025 | CT VS EHM | 0.49685439 | -5.192569 | 6.186278 | 1 |
| Li Jianshu 2025 | CT VS MOXI | 3.4553458 | 0.8553139 | 6.055378 | 1 |
| Li Jianshu 2025 | CT VS MOXI+ACU | 6.6100006 | 1.190037 | 12.02996 | 1 |
| Li Jianshu 2025 | CT VS MOXI+EHM | 5.1741934 | 1.028286 | 9.320102 | 1 |
| Li Jianshu 2025 | CT VS MOXI+IHM | 4.0428408 | 2.083217 | 6.002464 | 1 |
| Feng Xueqiang 2024 | ACU VS CT | 0.35889481 | -3.419632 | 4.701842 | 1 |
| Feng Xueqiang 2024 | ACU VS EHM | 3.2854204 | -0.7752972 | 5.795544 | 1 |
| Feng Xueqiang 2024 | ACU VS MOXI | 6.6100006 | 1.289926 | 11.93007 | 1 |
| Feng Xueqiang 2024 | ACU VS MOXI+ACU | 4.3180021 | 1.007141 | 7.628863 | 1 |
| Feng Xueqiang 2024 | ACU VS MOXI+EHM | 4.0444449 | 2.11939 | 5.9695 | 1 |
| Liu Maomao 2023 | CT VS ACU | 0.3749884 | -2.667063 | 2.917086 | 1 |
| Liu Maomao 2023 | CT VS EHM | 0.36152832 | -3.522031 | 4.798974 | 1 |
| Liu Maomao 2023 | CT VS MOXI | 3.2850035 | 0.7246594 | 5.845347 | 1 |
| Liu Maomao 2023 | CT VS MOXI+ACU | 6.6100006 | 1.179199 | 12.0408 | 1 |
| Liu Maomao 2023 | CT VS MOXI+EHM | 4.3155396 | 0.9410735 | 7.690006 | 1 |
| Liu Maomao 2023 | CT VS MOXI+IHM | 4.2987949 | 2.199045 | 6.398545 | 1 |
| Huang Xingduan 2024 | CT VS ACU | 0.3752364 | -3.774268 | 3.023796 | 1 |
| Huang Xingduan 2024 | CT VS EHM | 0.00656286 | -6.571891 | 6.588765 | 1 |
| Huang Xingduan 2024 | CT VS MOXI | 3.2847552 | 0.6780214 | 5.891489 | 1 |
| Huang Xingduan 2024 | CT VS MOXI+ACU | 6.6100006 | 1.077219 | 12.14278 | 1 |
| Huang Xingduan 2024 | CT VS MOXI+EHM | 4.31399 | 0.8803336 | 7.747646 | 1 |
| Huang Xingduan 2024 | CT VS MOXI+IHM | 4.0411315 | 2.042459 | 6.039804 | 1 |
| Peng Jing 2022 | CT VS ACU | 0.3752688 | -3.774181 | 3.023643 | 1 |
| Peng Jing 2022 | CT VS EHM | 0.72696634 | -5.337766 | 5.883833 | 1 |
| Peng Jing 2022 | CT VS MOXI | 3.2847228 | 0.6780505 | 5.891395 | 1 |
| Peng Jing 2022 | CT VS MOXI+ACU | 6.6100006 | 1.077328 | 12.14267 | 1 |
| Peng Jing 2022 | CT VS MOXI+EHM | 4.313827 | 0.8804567 | 7.747197 | 1 |
| Peng Jing 2022 | CT VS MOXI+IHM | 4.0411331 | 2.042498 | 6.039768 | 1 |
| Liang Guoyu 2025 | CT VS ACU | 0.3343145 | -3.652286 | 3.383657 | 1 |
| Liang Guoyu 2025 | CT VS EHM | 0.28040106 | -4.533478 | 4.972676 | 1 |
| Liang Guoyu 2025 | CT VS MOXI | 3.5257206 | 0.5967813 | 6.45466 | 1 |
| Liang Guoyu 2025 | CT VS MOXI+ACU | 6.6100006 | 1.099559 | 12.12044 | 1 |
| Liang Guoyu 2025 | CT VS MOXI+EHM | 4.3968137 | 0.9448498 | 7.848778 | 1 |
| Liang Guoyu 2025 | CT VS MOXI+IHM | 4.0414611 | 2.050523 | 6.032399 | 1 |
| Hu Guanghui 2023 | CT VS ACU | 0.3752603 | -2.745831 | 2.99531 | 1 |
| Hu Guanghui 2023 | CT VS EHM | 0.36323194 | -3.591234 | 4.86477 | 1 |
| Hu Guanghui 2023 | CT VS MOXI | 3.2847314 | 0.6903543 | 5.879108 | 1 |
| Hu Guanghui 2023 | CT VS MOXI+ACU | 6.6100006 | 1.104331 | 12.11567 | 1 |
| Hu Guanghui 2023 | CT VS MOXI+EHM | 4.3139472 | 0.8964388 | 7.731455 | 1 |
| Hu Guanghui 2023 | CT VS MOXI+IHM | 3.9026282 | 1.779378 | 6.025878 | 1 |

Leave-one-out sensitivity analysis of TCSS in the primary analysis of patients with DPN (Table S137)

| **dropped_id** | **comparison** | **eff** | **lci** | **uci** | **connected** |
| --- | --- | --- | --- | --- | --- |
| Ma Guoqing 2018 | CT VS ACU | -0.22086177 | -2.34948 | 2.791204 | 1 |
| Ma Guoqing 2018 | CT VS EHM | -1.560416 | -4.057815 | 0.9369829 | 1 |
| Ma Guoqing 2018 | CT VS MOXI | -1.4492991 | -2.598932 | -0.2996665 | 1 |
| Ma Guoqing 2018 | CT VS MOXI+ACU | -1.2190844 | -4.754098 | 2.315929 | 1 |
| Ma Guoqing 2018 | CT VS MOXI+EHM | -2.5565632 | -3.881203 | -1.231923 | 1 |
| Ma Guoqing 2018 | CT VS MOXI+IHM | -1.5809368 | -2.561488 | -0.600386 | 1 |
| Wang Jiawei 2023 | CT VS ACU | -0.03393521 | -1.418473 | 1.486343 | 1 |
| Wang Jiawei 2023 | CT VS EHM | -2.3089317 | -4.395423 | 0.2224403 | 1 |
| Wang Jiawei 2023 | CT VS MOXI | -1.5613763 | -2.406406 | -0.7163461 | 1 |
| Wang Jiawei 2023 | CT VS MOXI+ACU | -1.4060189 | -3.79612 | 0.9840819 | 1 |
| Wang Jiawei 2023 | CT VS MOXI+EHM | -3.6713643 | -4.941149 | -2.40158 | 1 |
| Wang Jiawei 2023 | CT VS MOXI+IHM | -1.550431 | -2.258785 | -0.8420767 | 1 |
| Fang Xiujuan 2020 | CT VS ACU | -0.43602143 | -1.55249 | 2.424533 | 1 |
| Fang Xiujuan 2020 | CT VS EHM | -1.401017 | -3.846136 | 1.044102 | 1 |
| Fang Xiujuan 2020 | CT VS MOXI | -1.1611978 | -2.428698 | -0.1063023 | 1 |
| Fang Xiujuan 2020 | CT VS MOXI+ACU | -1.00395 | -4.078824 | 2.070924 | 1 |
| Fang Xiujuan 2020 | CT VS MOXI+EHM | -2.4849872 | -3.762162 | -1.207812 | 1 |
| Fang Xiujuan 2020 | CT VS MOXI+IHM | -1.5778451 | -2.517654 | -0.6380362 | 1 |
| Feng Yun 2022 | CT VS ACU | -0.14994158 | -1.788925 | 2.088808 | 1 |
| Feng Yun 2022 | CT VS EHM | -1.5590808 | -4.030253 | 0.9120914 | 1 |
| Feng Yun 2022 | CT VS MOXI | -1.4474946 | -2.58071 | -0.314279 | 1 |
| Feng Yun 2022 | CT VS MOXI+ACU | -1.2899983 | -4.374526 | 1.794529 | 1 |
| Feng Yun 2022 | CT VS MOXI+EHM | -2.5552912 | -3.860883 | -1.249699 | 1 |
| Feng Yun 2022 | CT VS MOXI+IHM | -1.4372121 | -2.529158 | -0.3452661 | 1 |
| Zhang Xiujun 2020 | CT VS ACU | -0.15280323 | -1.742211 | 2.047818 | 1 |
| Zhang Xiujun 2020 | CT VS EHM | -1.5568331 | -3.985451 | 0.8717853 | 1 |
| Zhang Xiujun 2020 | CT VS MOXI | -1.4445026 | -2.550958 | -0.3380471 | 1 |
| Zhang Xiujun 2020 | CT VS MOXI+ACU | -1.2871392 | -4.308743 | 1.734464 | 1 |
| Zhang Xiujun 2020 | CT VS MOXI+EHM | -2.5531111 | -3.82759 | -1.278633 | 1 |
| Zhang Xiujun 2020 | CT VS MOXI+IHM | -1.3882569 | -2.429339 | -0.3471745 | 1 |
| Zhang Suzhao 2024 | CT VS ACU | -0.15008173 | -1.786549 | 2.086713 | 1 |
| Zhang Suzhao 2024 | CT VS EHM | -1.558971 | -4.027974 | 0.910032 | 1 |
| Zhang Suzhao 2024 | CT VS MOXI | -1.447348 | -2.579185 | -0.3155105 | 1 |
| Zhang Suzhao 2024 | CT VS MOXI+ACU | -1.2898583 | -4.371183 | 1.791467 | 1 |
| Zhang Suzhao 2024 | CT VS MOXI+EHM | -2.5551848 | -3.859191 | -1.251179 | 1 |
| Zhang Suzhao 2024 | CT VS MOXI+IHM | -1.4584957 | -2.526644 | -0.3903475 | 1 |
| Hu Guanghui 2024 | CT VS ACU | -0.14984795 | -1.79049 | 2.090186 | 1 |
| Hu Guanghui 2024 | CT VS EHM | -1.5591541 | -4.031767 | 0.9134591 | 1 |
| Hu Guanghui 2024 | CT VS MOXI | -1.4475925 | -2.581682 | -0.3135031 | 1 |
| Hu Guanghui 2024 | CT VS MOXI+ACU | -1.2900919 | -4.376749 | 1.796565 | 1 |
| Hu Guanghui 2024 | CT VS MOXI+EHM | -2.5553623 | -3.861995 | -1.24873 | 1 |
| Hu Guanghui 2024 | CT VS MOXI+IHM | -1.7333437 | -2.821051 | -0.6456367 | 1 |
| Li Shuomiao 2024 | CT VS ACU | -0.07873237 | -2.179293 | 2.021828 | 1 |
| Li Shuomiao 2024 | CT VS EHM | -1.6862952 | -4.205203 | 0.8326124 | 1 |
| Li Shuomiao 2024 | CT VS MOXI | -1.6762501 | -3.059366 | -0.2931343 | 1 |
| Li Shuomiao 2024 | CT VS MOXI+ACU | -1.5186475 | -4.714932 | 1.677637 | 1 |
| Li Shuomiao 2024 | CT VS MOXI+EHM | -2.6114816 | -3.938271 | -1.284693 | 1 |
| Li Shuomiao 2024 | CT VS MOXI+IHM | -1.5802939 | -2.551972 | -0.6086157 | 1 |
| Luo Xingzi 2020 | CT VS ACU | -0.15423376 | -1.719044 | 2.027512 | 1 |
| Luo Xingzi 2020 | CT VS EHM | -1.5556618 | -3.963197 | 0.8518735 | 1 |
| Luo Xingzi 2020 | CT VS MOXI | -1.4429573 | -2.535986 | -0.3499283 | 1 |
| Luo Xingzi 2020 | CT VS MOXI+ACU | -2.5519662 | -3.810901 | 1.293032 | 1 |
| Luo Xingzi 2020 | CT VS MOXI+EHM | -1.577259 | -2.509789 | -0.644729 | 1 |
| Feng Xueqiang 2024 | CT VS ACU | -0.08098438 | -2.413514 | 2.575483 | 1 |
| Feng Xueqiang 2024 | CT VS EHM | -1.5603906 | -4.05776 | 0.9369789 | 1 |
| Feng Xueqiang 2024 | CT VS MOXI | -1.4492541 | -2.598841 | -0.2996669 | 1 |
| Feng Xueqiang 2024 | CT VS MOXI+ACU | -1.3589467 | -4.839196 | 2.121303 | 1 |
| Feng Xueqiang 2024 | CT VS MOXI+EHM | -2.5565513 | -3.881172 | -1.231931 | 1 |
| Feng Xueqiang 2024 | CT VS MOXI+IHM | -1.5809358 | -2.561473 | -0.6003983 | 1 |
| Wang Lina 2022 | CT VS ACU | -0.15870583 | -1.65509 | 1.972502 | 1 |
| Wang Lina 2022 | CT VS EHM | -1.5521565 | -3.902562 | 0.7982495 | 1 |
| Wang Lina 2022 | CT VS MOXI | -1.4383358 | -2.495165 | -0.3815068 | 1 |
| Wang Lina 2022 | CT VS MOXI+ACU | -1.2812411 | -4.186714 | 1.624232 | 1 |
| Wang Lina 2022 | CT VS MOXI+EHM | -2.5485613 | -3.765239 | -1.331883 | 1 |
| Wang Lina 2022 | CT VS MOXI+IHM | -1.8889927 | -2.905536 | -0.8724489 | 1 |
| Wang Jiawei 2023 | CT VS ACU | -0.36645964 | -0.9902446 | 1.723164 | 1 |
| Wang Jiawei 2023 | CT VS EHM | -0.73881222 | -2.738819 | 1.261195 | 1 |
| Wang Jiawei 2023 | CT VS MOXI | -1.228224 | -2.000643 | -0.4558048 | 1 |
| Wang Jiawei 2023 | CT VS MOXI+ACU | -1.0735187 | -3.340859 | 1.193821 | 1 |
| Wang Jiawei 2023 | CT VS MOXI+EHM | -1.3901741 | -2.537582 | -0.242766 | 1 |
| Wang Jiawei 2023 | CT VS MOXI+IHM | -1.5422018 | -2.206149 | -0.8782543 | 1 |
| Hu Shuhan 2026 | CT VS ACU | -0.1686952 | -1.841235 | 2.178626 | 1 |
| Hu Shuhan 2026 | CT VS EHM | -1.4287798 | -2.664387 | -0.1931722 | 1 |
| Hu Shuhan 2026 | CT VS MOXI | -1.271246 | -4.413075 | 1.870583 | 1 |
| Hu Shuhan 2026 | CT VS MOXI+ACU | -2.584446 | -4.06464 | -1.104252 | 1 |
| Hu Shuhan 2026 | CT VS MOXI+EHM | -1.5804999 | -2.554995 | -0.606005 | 1 |
| Hu Shuhan 2026 | CT VS ACU | -0.04154605 | -2.057476 | 2.140568 | 1 |
| Hu Shuhan 2026 | CT VS EHM | -1.6199572 | -4.154062 | 0.9141477 | 1 |
| Hu Shuhan 2026 | CT VS MOXI | -1.5560033 | -2.917299 | -0.1947077 | 1 |
| Hu Shuhan 2026 | CT VS MOXI+ACU | -1.3983805 | -4.610366 | 1.813606 | 1 |
| Hu Shuhan 2026 | CT VS MOXI+EHM | -2.5829219 | -3.922728 | -1.243116 | 1 |
| Hu Shuhan 2026 | CT VS MOXI+IHM | -1.5810887 | -2.563765 | -0.5984126 | 1 |

Leave-one-out sensitivity analysis of markedly effective rate in the primary analysis of patients with DPN (Table S138)

| **dropped_id** | **comparison** | **log_eff** | **log_lci** | **log_uci** | **OR** | **OR_lci** | **OR_uci** |
| --- | --- | --- | --- | --- | --- | --- | --- |
| Wang Dan 2021 | CT VS ACU | -0.11589966 | -0.5343747 | 0.3025754 | 0.89056458 | 0.5860356 | 1.35334 |
| Wang Dan 2021 | CT VS EHM | 0.22008936 | -0.185985 | 0.6261637 | 1.2461881 | 0.8302861 | 1.870421 |
| Wang Dan 2021 | CT VS MOXI | 0.25989862 | 0.0280953 | 0.4917019 | 1.2967986 | 1.028494 | 1.635097 |
| Wang Dan 2021 | CT VS MOXI+ACU | 0.44899429 | 0.0346189 | 0.8633696 | 1.5667357 | 1.035225 | 2.371137 |
| Wang Dan 2021 | CT VS MOXI+EHM | 0.52902114 | 0.2073569 | 0.8506854 | 1.6972701 | 1.230422 | 2.341251 |
| Wang Dan 2021 | CT VS MOXI+IHM | 0.26048101 | 0.1010303 | 0.4199317 | 1.2975541 | 1.10631 | 1.521858 |
| Ji Hua 2025 | CT VS ACU | -0.04492062 | -0.4709316 | 0.3810903 | 0.95607337 | 0.6244203 | 1.46388 |
| Ji Hua 2025 | CT VS EHM | 0.25956673 | -0.1589878 | 0.6781213 | 1.2963683 | 0.8530068 | 1.970173 |
| Ji Hua 2025 | CT VS MOXI | 0.35184706 | 0.1332286 | 0.5704655 | 1.4216911 | 1.142511 | 1.76909 |
| Ji Hua 2025 | CT VS MOXI+ACU | 0.47428342 | 0.0541721 | 0.8943948 | 1.6068623 | 1.055666 | 2.445855 |
| Ji Hua 2025 | CT VS MOXI+EHM | 0.56800202 | 0.2417509 | 0.8942531 | 1.7647376 | 1.273477 | 2.445509 |
| Ji Hua 2025 | CT VS MOXI+IHM | 0.26220782 | 0.0946246 | 0.429791 | 1.2997966 | 1.099246 | 1.536936 |
| Zhang Xiujun 2020 | CT VS ACU | -0.0705938 | -0.5062145 | 0.3650269 | 0.93184033 | 0.6027731 | 1.440553 |
| Zhang Xiujun 2020 | CT VS EHM | 0.25417503 | -0.1831064 | 0.6914565 | 1.2893975 | 0.8326795 | 1.996621 |
| Zhang Xiujun 2020 | CT VS MOXI | 0.32373472 | 0.1147438 | 0.5327256 | 1.3822806 | 1.121586 | 1.703569 |
| Zhang Xiujun 2020 | CT VS MOXI+ACU | 0.46996939 | 0.0410681 | 0.8988706 | 1.5999452 | 1.041923 | 2.456827 |
| Zhang Xiujun 2020 | CT VS MOXI+EHM | 0.56203197 | 0.2266589 | 0.8974051 | 1.7542334 | 1.254402 | 2.453229 |
| Zhang Xiujun 2020 | CT VS MOXI+IHM | 0.24763532 | 0.0454053 | 0.4498653 | 1.2809927 | 1.046452 | 1.568101 |
| Zhang Suzhao 2024 | CT VS ACU | -0.06184059 | -0.4669965 | 0.3433153 | 0.94003272 | 0.6268823 | 1.409613 |
| Zhang Suzhao 2024 | CT VS EHM | 0.24421751 | -0.1521035 | 0.6405385 | 1.276622 | 0.8588994 | 1.897502 |
| Zhang Suzhao 2024 | CT VS MOXI | 0.32623802 | 0.1322639 | 0.5202122 | 1.3857452 | 1.141409 | 1.682385 |
| Zhang Suzhao 2024 | CT VS MOXI+ACU | 0.46512834 | 0.0546733 | 0.8755834 | 1.5922185 | 1.056195 | 2.400275 |
| Zhang Suzhao 2024 | CT VS MOXI+EHM | 0.55330442 | 0.2401169 | 0.8664919 | 1.7389899 | 1.271398 | 2.378552 |
| Zhang Suzhao 2024 | CT VS MOXI+IHM | 0.2346198 | 0.0760292 | 0.3932104 | 1.2644279 | 1.078994 | 1.48173 |
| Hu Guanghui 2024 | CT VS ACU | -0.06771753 | -0.4925138 | 0.3570787 | 0.93452441 | 0.6110883 | 1.429148 |
| Hu Guanghui 2024 | CT VS EHM | 0.25063759 | -0.1722273 | 0.6735025 | 1.2848444 | 0.8417878 | 1.961094 |
| Hu Guanghui 2024 | CT VS MOXI | 0.32443098 | 0.1208116 | 0.5280504 | 1.3832433 | 1.128412 | 1.695623 |
| Hu Guanghui 2024 | CT VS MOXI+ACU | 0.46818051 | 0.0460012 | 0.8903598 | 1.5970857 | 1.047076 | 2.436006 |
| Hu Guanghui 2024 | CT VS MOXI+EHM | 0.55889882 | 0.2314025 | 0.8863951 | 1.7487458 | 1.260366 | 2.426367 |
| Hu Guanghui 2024 | CT VS MOXI+IHM | 0.25400205 | 0.0752209 | 0.4327832 | 1.2891744 | 1.078122 | 1.541542 |
| Liu Xiangxiu 2012 | CT VS ACU | -0.06385077 | -0.4753733 | 0.3476717 | 0.93814499 | 0.621653 | 1.415767 |
| Liu Xiangxiu 2012 | CT VS EHM | 0.24630618 | -0.1586617 | 0.651274 | 1.2792912 | 0.853285 | 1.917983 |
| Liu Xiangxiu 2012 | CT VS MOXI | 0.32556801 | 0.1284648 | 0.5226712 | 1.384817 | 1.137081 | 1.686527 |
| Liu Xiangxiu 2012 | CT VS MOXI+ACU | 0.4660923 | 0.0519033 | 0.8802813 | 1.5937541 | 1.053274 | 2.411578 |
| Liu Xiangxiu 2012 | CT VS MOXI+EHM | 0.55511027 | 0.2373236 | 0.8728969 | 1.7421331 | 1.267851 | 2.393836 |
| Liu Xiangxiu 2012 | CT VS MOXI+IHM | 0.24934231 | 0.0881216 | 0.4105631 | 1.2831812 | 1.092121 | 1.507666 |
| Wan Zhihui 2025 | CT VS ACU | -0.03140879 | -0.4340687 | 0.3712511 | 0.96907934 | 0.6478677 | 1.449547 |
| Wan Zhihui 2025 | CT VS EHM | 0.11739893 | -0.3346789 | 0.5694768 | 1.124568 | 0.7155678 | 1.767342 |
| Wan Zhihui 2025 | CT VS MOXI | 0.36176271 | 0.1608877 | 0.5626377 | 1.4358582 | 1.174553 | 1.755296 |
| Wan Zhihui 2025 | CT VS MOXI+ACU | 0.47285464 | 0.0661773 | 0.879532 | 1.6045681 | 1.068416 | 2.409772 |
| Wan Zhihui 2025 | CT VS MOXI+EHM | 0.42682087 | 0.0405585 | 0.8130832 | 1.5323781 | 1.041392 | 2.254849 |
| Wan Zhihui 2025 | CT VS MOXI+IHM | 0.25895456 | 0.1070442 | 0.4108649 | 1.2955749 | 1.112983 | 1.508122 |
| Wu Min 2017 | CT VS ACU | -0.08738048 | -0.5024123 | 0.3276514 | 0.91632839 | 0.6050693 | 1.387705 |
| Wu Min 2017 | CT VS EHM | 0.23544374 | -0.1699168 | 0.6408043 | 1.2654702 | 0.843735 | 1.898007 |
| Wu Min 2017 | CT VS MOXI | 0.29641609 | 0.0929225 | 0.4999097 | 1.3450297 | 1.097377 | 1.648572 |
| Wu Min 2017 | CT VS MOXI+ACU | 0.45888884 | 0.0443641 | 0.8734136 | 1.5823148 | 1.045363 | 2.395073 |
| Wu Min 2017 | CT VS MOXI+EHM | 0.54421232 | 0.2265488 | 0.8618758 | 1.7232505 | 1.254264 | 2.367598 |
| Wu Min 2017 | CT VS MOXI+IHM | 0.26102584 | 0.0992511 | 0.4228006 | 1.2982612 | 1.104344 | 1.52623 |
| Jin Zhu 2020 | CT VS ACU | -0.05614136 | -0.4773383 | 0.3650556 | 0.94540548 | 0.6204326 | 1.440594 |
| Jin Zhu 2020 | CT VS EHM | 0.25344393 | -0.1623753 | 0.6692632 | 1.2884551 | 0.850122 | 1.952798 |
| Jin Zhu 2020 | CT VS MOXI | 0.33730783 | 0.1283844 | 0.5462312 | 1.4011703 | 1.13699 | 1.726733 |
| Jin Zhu 2020 | CT VS MOXI+ACU | 0.47035044 | 0.0514366 | 0.8892643 | 1.600555 | 1.052782 | 2.433339 |
| Jin Zhu 2020 | CT VS MOXI+EHM | 0.56194195 | 0.2376417 | 0.8862422 | 1.7540755 | 1.268255 | 2.425996 |
| Jin Zhu 2020 | CT VS MOXI+IHM | 0.26197742 | 0.0955336 | 0.4284213 | 1.2994972 | 1.100246 | 1.534832 |
| Luo Xingzi 2020 | CT VS ACU | 0.06137251 | -0.3984183 | 0.5211633 | 1.0632949 | 0.6713811 | 1.683985 |
| Luo Xingzi 2020 | CT VS EHM | 0.25327967 | -0.1450801 | 0.6516395 | 1.2882435 | 0.864953 | 1.918684 |
| Luo Xingzi 2020 | CT VS MOXI | 0.34905984 | 0.1507243 | 0.5473954 | 1.417734 | 1.162676 | 1.728745 |
| Luo Xingzi 2020 | CT VS MOXI+ACU | 0.33542019 | -0.1380081 | 0.8088484 | 1.3985279 | 0.8710917 | 2.245321 |
| Luo Xingzi 2020 | CT VS MOXI+EHM | 0.56232696 | 0.2475705 | 0.8770835 | 1.754751 | 1.28091 | 2.403878 |
| Luo Xingzi 2020 | CT VS MOXI+IHM | 0.26011114 | 0.1026098 | 0.4176125 | 1.2970742 | 1.108059 | 1.518332 |
| Li Jianshu 2025 | CT VS ACU | -0.07378067 | -0.4807086 | 0.3331473 | 0.9288754 | 0.6183451 | 1.395353 |
| Li Jianshu 2025 | CT VS EHM | 0.1467394 | -0.2714722 | 0.564951 | 1.1580521 | 0.7622564 | 1.759362 |
| Li Jianshu 2025 | CT VS MOXI | 0.31163306 | 0.1154236 | 0.5078425 | 1.3656535 | 1.122349 | 1.661702 |
| Li Jianshu 2025 | CT VS MOXI+ACU | 0.46158565 | 0.0506948 | 0.8724765 | 1.5865878 | 1.052002 | 2.392829 |
| Li Jianshu 2025 | CT VS MOXI+EHM | 0.4557863 | 0.115398 | 0.7961746 | 1.5774132 | 1.12232 | 2.217044 |
| Li Jianshu 2025 | CT VS MOXI+IHM | 0.26010398 | 0.1027473 | 0.4174607 | 1.2970649 | 1.108211 | 1.518102 |
| Wang Yue 2022 | CT VS ACU | 0.06264672 | -0.3495458 | 0.4748392 | 1.0646507 | 0.7050082 | 1.607756 |
| Wang Yue 2022 | CT VS EHM | 0.24933026 | -0.1329538 | 0.6316143 | 1.2831657 | 0.8755056 | 1.880644 |
| Wang Yue 2022 | CT VS MOXI | 0.35009294 | 0.1602913 | 0.5398946 | 1.4191994 | 1.173853 | 1.715826 |
| Wang Yue 2022 | CT VS MOXI+ACU | 0.8742969 | 0.2824352 | 1.466159 | 2.3971892 | 1.326356 | 4.33256 |
| Wang Yue 2022 | CT VS MOXI+EHM | 0.55892747 | 0.2527599 | 0.865095 | 1.7487959 | 1.287574 | 2.375232 |
| Wang Yue 2022 | CT VS MOXI+IHM | 0.25845417 | 0.1093129 | 0.4075955 | 1.2949268 | 1.115511 | 1.503199 |
| Feng Xueqiang 2024 | CT VS ACU | -0.47551055 | -1.317667 | 0.3666454 | 0.62156763 | 0.2677594 | 1.442886 |
| Feng Xueqiang 2024 | CT VS EHM | 0.25328104 | -0.1450794 | 0.6516415 | 1.2882453 | 0.8649536 | 1.918688 |
| Feng Xueqiang 2024 | CT VS MOXI | 0.34906308 | 0.150726 | 0.5474002 | 1.4177386 | 1.162678 | 1.728753 |
| Feng Xueqiang 2024 | CT VS MOXI+ACU | 0.33541987 | 0.1379986 | 0.8088384 | 1.3985275 | 1.0870999 | 2.245298 |
| Feng Xueqiang 2024 | CT VS MOXI+EHM | 0.56232831 | 0.2475713 | 0.8770853 | 1.7547534 | 1.280911 | 2.403883 |
| Feng Xueqiang 2024 | CT VS MOXI+IHM | 0.26011119 | 0.1026096 | 0.4176128 | 1.2970743 | 1.108059 | 1.518333 |
| Du Xinze 2023 | CT VS ACU | -0.06659152 | -0.4882075 | 0.3550245 | 0.93557729 | 0.6137255 | 1.426216 |
| Du Xinze 2023 | CT VS EHM | 0.24932993 | -0.1692403 | 0.6679002 | 1.2831653 | 0.844306 | 1.950138 |
| Du Xinze 2023 | CT VS MOXI | 0.32473994 | 0.1229541 | 0.5265258 | 1.3836708 | 1.130833 | 1.69304 |
| Du Xinze 2023 | CT VS MOXI+ACU | 0.46753783 | 0.0475444 | 0.8875312 | 1.5960596 | 1.048693 | 2.429125 |
| Du Xinze 2023 | CT VS MOXI+EHM | 0.55774922 | 0.2321127 | 0.8833858 | 1.7467365 | 1.261262 | 2.419076 |
| Du Xinze 2023 | CT VS MOXI+IHM | 0.22676889 | 0.0263274 | 0.4272104 | 1.2545399 | 1.026677 | 1.532975 |
| Wang Lina 2022 | CT VS ACU | -0.05997975 | -0.4595762 | 0.3396167 | 0.94178361 | 0.6315513 | 1.404409 |
| Wang Lina 2022 | CT VS EHM | 0.24237115 | -0.1463428 | 0.6310851 | 1.2742671 | 0.8638615 | 1.879649 |
| Wang Lina 2022 | CT VS MOXI | 0.32690125 | 0.1356781 | 0.5181245 | 1.3866645 | 1.145313 | 1.678876 |
| Wang Lina 2022 | CT VS MOXI+ACU | 0.46430095 | 0.0570731 | 0.8715288 | 1.5909017 | 1.058733 | 2.390563 |
| Wang Lina 2022 | CT VS MOXI+EHM | 0.55172062 | 0.2424999 | 0.8609413 | 1.7362379 | 1.274431 | 2.365386 |
| Wang Lina 2022 | CT VS MOXI+IHM | 0.30452777 | 0.1353431 | 0.4737124 | 1.3559845 | 1.14493 | 1.605945 |
| Li Xiaoting 2014 | CT VS ACU | -0.1014745 | -0.5228003 | 0.3198513 | 0.90350422 | 0.592858 | 1.376923 |
| Li Xiaoting 2014 | CT VS EHM | 0.24393357 | -0.1620298 | 0.649897 | 1.2762595 | 0.8504158 | 1.915343 |
| Li Xiaoting 2014 | CT VS MOXI | 0.31857034 | 0.1203219 | 0.5168188 | 1.3751604 | 1.12786 | 1.676685 |
| Li Xiaoting 2014 | CT VS MOXI+ACU | 0.34500507 | 0.1263528 | 0.8163629 | 1.4119971 | 1.0883039 | 2.262257 |
| Li Xiaoting 2014 | CT VS MOXI+EHM | 0.55270142 | 0.2344572 | 0.8709456 | 1.7379416 | 1.264222 | 2.389169 |
| Li Xiaoting 2014 | CT VS MOXI+IHM | 0.26102909 | 0.0991693 | 0.4228888 | 1.2982654 | 1.104253 | 1.526365 |
| Luo Xiaohong 2022 | CT VS ACU | -0.01438018 | -0.3957696 | 0.3670092 | 0.98572272 | 0.6731618 | 1.443411 |
| Luo Xiaohong 2022 | CT VS EHM | 0.55902207 | -0.0950122 | 1.023032 | 1.7489613 | 0.991672 | 2.781616 |
| Luo Xiaohong 2022 | CT VS MOXI | 0.37645711 | 0.1906178 | 0.5622965 | 1.457113 | 1.209997 | 1.754697 |
| Luo Xiaohong 2022 | CT VS MOXI+ACU | 0.47337182 | 0.0771759 | 0.8695677 | 1.6053982 | 1.080232 | 2.385879 |
| Luo Xiaohong 2022 | CT VS MOXI+EHM | 0.86943922 | 0.4540178 | 1.284861 | 2.3855727 | 1.574626 | 3.614165 |
| Luo Xiaohong 2022 | CT VS MOXI+IHM | 0.25625995 | 0.1183265 | 0.3941934 | 1.2920886 | 1.125612 | 1.483187 |
| Huang Xingduan 2024 | CT VS ACU | -0.07048153 | -0.5056465 | 0.3646834 | 0.93194495 | 0.6031156 | 1.440058 |
| Huang Xingduan 2024 | CT VS EHM | 0.24557119 | -0.2263004 | 0.7174428 | 1.2783513 | 0.7974786 | 2.049186 |
| Huang Xingduan 2024 | CT VS MOXI | 0.32376135 | 0.1149885 | 0.5325342 | 1.3823174 | 1.121861 | 1.703243 |
| Huang Xingduan 2024 | CT VS MOXI+ACU | 0.46989727 | 0.0412775 | 0.8985171 | 1.5998298 | 1.042141 | 2.455958 |
| Huang Xingduan 2024 | CT VS MOXI+EHM | 0.5619234 | 0.2268925 | 0.8969544 | 1.754043 | 1.254695 | 2.452123 |
| Huang Xingduan 2024 | CT VS MOXI+IHM | 0.26445409 | 0.0868239 | 0.4420843 | 1.3027196 | 1.090705 | 1.555947 |
| Peng Jing 2022 | CT VS ACU | -0.07048029 | -0.50565 | 0.3646895 | 0.93194611 | 0.6031134 | 1.440067 |
| Peng Jing 2022 | CT VS EHM | 0.27424796 | -0.3381093 | 0.8866052 | 1.315541 | 0.7131174 | 2.426877 |
| Peng Jing 2022 | CT VS MOXI | 0.32376423 | 0.1149888 | 0.5325396 | 1.3823214 | 1.121861 | 1.703252 |
| Peng Jing 2022 | CT VS MOXI+ACU | 0.46989887 | 0.0412759 | 0.8985218 | 1.5998324 | 1.04214 | 2.45597 |
| Peng Jing 2022 | CT VS MOXI+EHM | 0.56194554 | 0.2268997 | 0.8969914 | 1.7540818 | 1.254704 | 2.452214 |
| Peng Jing 2022 | CT VS MOXI+IHM | 0.26445485 | 0.0868213 | 0.4420884 | 1.3027206 | 1.090702 | 1.555953 |
| Liang Guoyu 2025 | CT VS ACU | -0.08519238 | -0.5251788 | 0.3547941 | 0.9183356 | 0.5914496 | 1.425887 |
| Liang Guoyu 2025 | CT VS EHM | 0.24516319 | -0.1885278 | 0.6788542 | 1.2778298 | 0.8281775 | 1.971617 |
| Liang Guoyu 2025 | CT VS MOXI | 0.30410543 | 0.0686694 | 0.5395415 | 1.3554119 | 1.071082 | 1.71522 |
| Liang Guoyu 2025 | CT VS MOXI+ACU | 0.46426992 | 0.0371083 | 0.8914315 | 1.5908523 | 1.037805 | 2.438618 |
| Liang Guoyu 2025 | CT VS MOXI+EHM | 0.55317645 | 0.2181594 | 0.8881935 | 1.7387674 | 1.243785 | 2.430734 |
| Liang Guoyu 2025 | CT VS MOXI+IHM | 0.26385679 | 0.0889058 | 0.4388078 | 1.3019417 | 1.092978 | 1.550857 |
| Hu Shuhan 2026 | CT VS ACU | -0.06395118 | -0.4862072 | 0.3583049 | 0.93805079 | 0.6149544 | 1.430902 |
| Hu Shuhan 2026 | CT VS EHM | 0.25015069 | -0.1666554 | 0.6669568 | 1.2842189 | 0.8464913 | 1.948299 |
| Hu Shuhan 2026 | CT VS MOXI | 0.32778601 | 0.1187963 | 0.5367757 | 1.3878919 | 1.12614 | 1.710483 |
| Hu Shuhan 2026 | CT VS MOXI+ACU | 0.46813073 | 0.0487236 | 0.8875378 | 1.5970062 | 1.04993 | 2.429141 |
| Hu Shuhan 2026 | CT VS MOXI+EHM | 0.5586088 | 0.2339684 | 0.8832492 | 1.7482387 | 1.263605 | 2.418746 |
| Hu Shuhan 2026 | CT VS MOXI+IHM | 0.26212266 | 0.0950166 | 0.4292287 | 1.299686 | 1.099677 | 1.536072 |
| Hu Guanghui 2023 | CT VS ACU | -0.05668198 | -0.4529145 | 0.3395505 | 0.94489451 | 0.6357725 | 1.404316 |
| Hu Guanghui 2023 | CT VS EHM | 0.23928353 | -0.1416497 | 0.6202168 | 1.2703387 | 0.8679252 | 1.859331 |
| Hu Guanghui 2023 | CT VS MOXI | 0.32816923 | 0.1395564 | 0.516782 | 1.3884239 | 1.149764 | 1.676624 |
| Hu Guanghui 2023 | CT VS MOXI+ACU | 0.46297202 | 0.0601889 | 0.8657551 | 1.5887889 | 1.062037 | 2.3768 |
| Hu Guanghui 2023 | CT VS MOXI+EHM | 0.54910073 | 0.2420108 | 0.8561906 | 1.7316951 | 1.273808 | 2.354176 |
| Hu Guanghui 2023 | CT VS MOXI+IHM | 0.31928529 | 0.147363 | 0.4912076 | 1.3761439 | 1.158774 | 1.634289 |

Leave-one-out sensitivity analysis of FPG in the primary analysis of patients with DPN (Table S139)

| **dropped_id** | **comparison** | **eff** | **lci** | **uci** | **connected** |
| --- | --- | --- | --- | --- | --- |
| Wang Jiawei 2023 | CT VS EHM | -0.35842806 | -1.426344 | 0.7094882 | 1 |
| Wang Jiawei 2023 | CT VS MOXI | -0.3889113 | -0.9640743 | 0.1862517 | 1 |
| Wang Jiawei 2023 | CT VS MOXI+EHM | -0.69854595 | -1.465055 | 0.0679629 | 1 |
| Wang Jiawei 2023 | CT VS MOXI+IHM | -1.2305087 | -1.63935 | -0.8216673 | 1 |
| Zhang Xiujun 2020 | CT VS EHM | -0.1967601 | -1.215063 | 0.8215424 | 1 |
| Zhang Xiujun 2020 | CT VS MOXI | -0.39689588 | -1.002811 | 0.2090189 | 1 |
| Zhang Xiujun 2020 | CT VS MOXI+EHM | -0.53685276 | -1.178607 | 0.1049017 | 1 |
| Zhang Xiujun 2020 | CT VS MOXI+IHM | -1.2174326 | -1.695798 | -0.739067 | 1 |
| Xu Xiaowei 2025 | CT VS EHM | -0.57833976 | -1.598354 | 0.4416746 | 1 |
| Xu Xiaowei 2025 | CT VS MOXI | -0.35088877 | -0.827756 | 0.1259784 | 1 |
| Xu Xiaowei 2025 | CT VS MOXI+EHM | -0.91843435 | -1.768763 | 0.0681055 | 1 |
| Xu Xiaowei 2025 | CT VS MOXI+IHM | -1.2429992 | -1.584469 | -0.9015294 | 1 |
| Zhang Suzhao 2024 | CT VS EHM | -0.11563809 | -0.9358969 | 0.7046208 | 1 |
| Zhang Suzhao 2024 | CT VS MOXI | -0.34109792 | -0.8386648 | 0.156469 | 1 |
| Zhang Suzhao 2024 | CT VS MOXI+EHM | -0.45566927 | -1.086363 | 0.1750245 | 1 |
| Zhang Suzhao 2024 | CT VS MOXI+IHM | -1.3508472 | -1.72108 | -0.9806148 | 1 |
| Hu Guanghui 2024 | CT VS EHM | -0.16243574 | -1.049984 | 0.7251125 | 1 |
| Hu Guanghui 2024 | CT VS MOXI | -0.37325054 | -0.9050287 | 0.1585276 | 1 |
| Hu Guanghui 2024 | CT VS MOXI+EHM | -0.50249432 | -1.093314 | 0.0883254 | 1 |
| Hu Guanghui 2024 | CT VS MOXI+IHM | -1.2827332 | -1.674369 | -0.8910976 | 1 |
| Liu Xiangxiu 2012 | CT VS EHM | -0.20108845 | -1.238654 | 0.8364773 | 1 |
| Liu Xiangxiu 2012 | CT VS MOXI | -0.39988641 | -1.016114 | 0.2163411 | 1 |
| Liu Xiangxiu 2012 | CT VS MOXI+EHM | -0.54118677 | -1.190397 | 0.1080236 | 1 |
| Liu Xiangxiu 2012 | CT VS MOXI+IHM | -1.1753444 | -1.685768 | -0.6649209 | 1 |
| Li Shuomiao 2024 | CT VS EHM | -0.17930804 | -1.128116 | 0.7695004 | 1 |
| Li Shuomiao 2024 | CT VS MOXI | -0.61742337 | -1.565735 | 0.3308887 | 1 |
| Li Shuomiao 2024 | CT VS MOXI+EHM | -0.5193813 | -1.13563 | 0.0968675 | 1 |
| Li Shuomiao 2024 | CT VS MOXI+IHM | -1.2318796 | -1.632903 | -0.8308558 | 1 |
| Du Minzhen 2019 | CT VS EHM | -0.17529604 | -1.108393 | 0.7578014 | 1 |
| Du Minzhen 2019 | CT VS MOXI | -0.35678336 | -0.9533826 | 0.2398159 | 1 |
| Du Minzhen 2019 | CT VS MOXI+EHM | -0.5153655 | -1.124579 | 0.0938481 | 1 |
| Du Minzhen 2019 | CT VS MOXI+IHM | -1.2328052 | -1.628012 | -0.837598 | 1 |
| Dong Qingmei 2023 | CT VS EHM | -0.17187103 | -1.092982 | 0.7492402 | 1 |
| Dong Qingmei 2023 | CT VS MOXI | -0.31491689 | -0.9005598 | 0.2707261 | 1 |
| Dong Qingmei 2023 | CT VS MOXI+EHM | -0.51193741 | -1.116934 | 0.093059 | 1 |
| Dong Qingmei 2023 | CT VS MOXI+IHM | -1.2335897 | -1.624101 | -0.8430784 | 1 |
| Wang Jiawei 2023 | CT VS EHM | -0.11491746 | -0.3302526 | 0.5600876 | 1 |
| Wang Jiawei 2023 | CT VS MOXI | -0.27590268 | -0.4842336 | 0.0675718 | 1 |
| Wang Jiawei 2023 | CT VS MOXI+EHM | -0.22508436 | -0.5565552 | 0.1063865 | 1 |
| Wang Jiawei 2023 | CT VS MOXI+IHM | -1.2732265 | -1.52404 | -1.022413 | 1 |
| Huang Xingduan 2024 | CT VS EHM | -0.36775782 | -0.8810087 | 0.1454931 | 1 |
| Huang Xingduan 2024 | CT VS MOXI | -0.4945711 | -1.070275 | 0.081133 | 1 |
| Huang Xingduan 2024 | CT VS MOXI+EHM | -1.2375213 | -1.605083 | 0.8699592 | 1 |
| Hu Guanghui 2023 | CT VS EHM | -0.16131153 | -1.045829 | 0.7232063 | 1 |
| Hu Guanghui 2023 | CT VS MOXI | -0.37247755 | -0.902568 | 0.1576129 | 1 |
| Hu Guanghui 2023 | CT VS MOXI+EHM | -0.50136924 | -1.091802 | 0.0890631 | 1 |
| Hu Guanghui 2023 | CT VS MOXI+IHM | -1.1363216 | -1.562853 | -0.7097899 | 1 |

Leave-one-out sensitivity analysis of 2hPG in the primary analysis of patients with DPN (Table S140)

| **dropped_id** | **comparison** | **eff** | **lci** | **uci** | **connected** |
| --- | --- | --- | --- | --- | --- |
| Tang Huazhen 2022 | CT VS EHM | -0.26422323 | -1.825447 | 1.297 | 1 |
| Tang Huazhen 2022 | CT VS MOXI | -0.45231825 | -1.366627 | 0.4619908 | 1 |
| Tang Huazhen 2022 | CT VS MOXI+EHM | -1.1343364 | -2.247641 | -0.0210323 | 1 |
| Tang Huazhen 2022 | CT VS MOXI+IHM | -1.41458 | -1.951266 | -0.8778943 | 1 |
| Zhang Xiujun 2020 | CT VS EHM | -0.34107551 | -2.078339 | 1.396188 | 1 |
| Zhang Xiujun 2020 | CT VS MOXI | -0.47685967 | -1.461166 | 0.5074467 | 1 |
| Zhang Xiujun 2020 | CT VS MOXI+EHM | -1.2112375 | -2.422701 | -0.0002261 | 1 |
| Zhang Xiujun 2020 | CT VS MOXI+IHM | -1.4940914 | -2.167444 | -0.8207384 | 1 |
| Zhang Xiujun 2020 | CT VS MOXI+MT | -1.96 | -3.24339 | -0.6766105 | 1 |
| Xu Xiaowei 2025 | CT VS EHM | -2.1577183 | -4.045271 | 0.2701653 | 1 |
| Xu Xiaowei 2025 | CT VS MOXI | -0.37731784 | -1.127505 | 0.3728698 | 1 |
| Xu Xiaowei 2025 | CT VS MOXI+EHM | -3.027908 | -4.769567 | -1.286249 | 1 |
| Xu Xiaowei 2025 | CT VS MOXI+IHM | -1.4223666 | -1.808669 | -1.036064 | 1 |
| Xu Xiaowei 2025 | CT VS MOXI+MT | -1.96 | -2.750876 | -1.169124 | 1 |
| Zhang Suzhao 2024 | CT VS EHM | -0.37144124 | -2.173552 | 1.430669 | 1 |
| Zhang Suzhao 2024 | CT VS MOXI | -0.48649849 | -1.499826 | 0.5268289 | 1 |
| Zhang Suzhao 2024 | CT VS MOXI+EHM | -1.2416277 | -2.47801 | -0.0052451 | 1 |
| Zhang Suzhao 2024 | CT VS MOXI+IHM | -1.4539752 | -2.168162 | -0.7397885 | 1 |
| Zhang Suzhao 2024 | CT VS MOXI+MT | -1.96 | -3.307497 | -0.6125034 | 1 |
| Hu Guanghui 2024 | CT VS EHM | -0.31512602 | -1.99655 | 1.366298 | 1 |
| Hu Guanghui 2024 | CT VS MOXI | -0.46860044 | -1.429087 | 0.491886 | 1 |
| Hu Guanghui 2024 | CT VS MOXI+EHM | -1.1852697 | -2.371208 | -0.0006682 | 1 |
| Hu Guanghui 2024 | CT VS MOXI+IHM | -1.5062341 | -2.141252 | -0.8712158 | 1 |
| Hu Guanghui 2024 | CT VS MOXI+MT | -1.96 | -3.191772 | -0.7282283 | 1 |
| Liu Xiangxiu 2012 | CT VS EHM | -0.34123461 | -2.075188 | 1.392718 | 1 |
| Liu Xiangxiu 2012 | CT VS MOXI | -0.47691024 | -1.460715 | 0.5068942 | 1 |
| Liu Xiangxiu 2012 | CT VS MOXI+EHM | -1.2113968 | -2.417755 | -0.0050387 | 1 |
| Liu Xiangxiu 2012 | CT VS MOXI+IHM | -1.3520868 | -2.011191 | -0.6929829 | 1 |
| Liu Xiangxiu 2012 | CT VS MOXI+MT | -1.96 | -3.243715 | -0.6762854 | 1 |
| Du Minzhen 2019 | CT VS EHM | -0.30854287 | -1.976227 | 1.359141 | 1 |
| Du Minzhen 2019 | CT VS MOXI | -0.33615437 | -1.491943 | 0.8196345 | 1 |
| Du Minzhen 2019 | CT VS MOXI+EHM | -1.1786822 | -2.358289 | -0.0009248 | 1 |
| Du Minzhen 2019 | CT VS MOXI+IHM | -1.413523 | -1.985487 | -0.8415588 | 1 |
| Du Minzhen 2019 | CT VS MOXI+MT | -1.96 | -3.179095 | -0.7409055 | 1 |
| Dong Qingmei 2023 | CT VS EHM | -0.26584041 | -1.839765 | 1.308085 | 1 |
| Dong Qingmei 2023 | CT VS MOXI | -0.25834937 | -1.264957 | 0.7482587 | 1 |
| Dong Qingmei 2023 | CT VS MOXI+EHM | -1.1359545 | -2.264132 | -0.0077764 | 1 |
| Dong Qingmei 2023 | CT VS MOXI+IHM | -1.4145394 | -1.952481 | -0.8765984 | 1 |
| Dong Qingmei 2023 | CT VS MOXI+MT | -1.96 | -3.100505 | -0.8194955 | 1 |
| Wang Jiawei 2023 | CT VS EHM | 0.39001046 | -0.6777811 | 1.457802 | 1 |
| Wang Jiawei 2023 | CT VS MOXI | -0.37731777 | -1.127505 | 0.3728697 | 1 |
| Wang Jiawei 2023 | CT VS MOXI+EHM | -0.47999272 | -1.261239 | -0.3012538 | 1 |
| Wang Jiawei 2023 | CT VS MOXI+IHM | -1.4223666 | -1.808669 | -1.036064 | 1 |
| Wang Jiawei 2023 | CT VS MOXI+MT | -1.96 | -2.750876 | -1.169124 | 1 |
| Huang Xingduan 2024 | CT VS EHM | -0.45235632 | -1.366767 | 0.4620542 | 1 |
| Huang Xingduan 2024 | CT VS MOXI | -1.1346318 | -2.248387 | -0.0208765 | 1 |
| Huang Xingduan 2024 | CT VS MOXI+EHM | -1.4145771 | -1.951354 | -0.8778006 | 1 |
| Huang Xingduan 2024 | CT VS MOXI+IHM | -1.96 | -3.097852 | -0.8221478 | 1 |
| Hu Shuhan 2026 | CT VS EHM | -0.25649503 | -1.815407 | 1.302417 | 1 |
| Hu Shuhan 2026 | CT VS MOXI | -0.94552736 | -2.204139 | 0.3130839 | 1 |
| Hu Shuhan 2026 | CT VS MOXI+EHM | -1.1266042 | -2.250411 | -0.0027974 | 1 |
| Hu Shuhan 2026 | CT VS MOXI+IHM | -1.4147764 | -1.945618 | -0.883935 | 1 |
| Hu Shuhan 2026 | CT VS MOXI+MT | -1.96 | -3.084072 | -0.8359277 | 1 |
| Hu Guanghui 2023 | CT VS EHM | -0.20545613 | -1.767026 | 1.356113 | 1 |
| Hu Guanghui 2023 | CT VS MOXI | -0.43334379 | -1.322705 | 0.4560178 | 1 |
| Hu Guanghui 2023 | CT VS MOXI+EHM | -1.0755414 | -2.282373 | -0.1312907 | 1 |
| Hu Guanghui 2023 | CT VS MOXI+IHM | -1.2501591 | -1.812077 | -0.6882411 | 1 |
| Hu Guanghui 2023 | CT VS MOXI+MT | -1.96 | -2.99847 | -0.9215302 | 1 |

Leave-one-out sensitivity analysis in the subgroup analysis

Leave-one-out sensitivity analysis of common peroneal MNCV in the subgroup analysis of patients with DPN (Table S141)

| **dropped_id** | **comparison** | **eff** | **lci** | **uci** | **connected** |
| --- | --- | --- | --- | --- | --- |
| Ma Guoqing 2018 | CT VS ACU | 0.3539991 | -3.785166 | 3.9228324 | 1 |
| Ma Guoqing 2018 | CT VS EHM | 0.05981061 | -4.36219 | 4.481811 | 1 |
| Ma Guoqing 2018 | CT VS GIM | 3.4185384 | 1.109239 | 5.727838 | 1 |
| Ma Guoqing 2018 | CT VS HM | 2.2155822 | -0.6560744 | 5.087239 | 1 |
| Ma Guoqing 2018 | CT VS IHM | 1.1662943 | -3.299308 | 5.631897 | 1 |
| Ma Guoqing 2018 | CT VS MM | 5.8767652 | 3.91362 | 7.839911 | 1 |
| Ma Guoqing 2018 | CT VS TF | 6.3795385 | 3.648719 | 9.110358 | 1 |
| Ma Guoqing 2018 | CT VS WDM | 4.2001236 | 1.941415 | 6.458832 | 1 |
| Ma Guoqing 2018 | CT VS WN | 3.3159027 | 1.85978 | 4.772025 | 1 |
| Feng Yun 2022 | CT VS ACU | 0.5864501 | -3.747846 | 4.5749459 | 1 |
| Feng Yun 2022 | CT VS EHM | 0.06078575 | -4.179573 | 4.301144 | 1 |
| Feng Yun 2022 | CT VS GIM | 3.4153732 | 1.1937 | 5.637047 | 1 |
| Feng Yun 2022 | CT VS HM | 2.216012 | -0.5509347 | 4.982959 | 1 |
| Feng Yun 2022 | CT VS IHM | 1.1628672 | -3.134332 | 5.460067 | 1 |
| Feng Yun 2022 | CT VS MM | 5.8733041 | 3.987062 | 7.759546 | 1 |
| Feng Yun 2022 | CT VS TF | 6.3790707 | 3.758827 | 8.999314 | 1 |
| Feng Yun 2022 | CT VS WDM | 4.2010734 | 2.031312 | 6.370835 | 1 |
| Feng Yun 2022 | CT VS WN | 2.5758054 | 1.056296 | 4.095314 | 1 |
| Xie Lingling 2025 | CT VS ACU | 0.9164984 | -2.568786 | 3.735789 | 1 |
| Xie Lingling 2025 | CT VS EHM | 0.25815406 | -4.659109 | 5.142801 | 1 |
| Xie Lingling 2025 | CT VS GIM | 3.4270552 | 0.8067048 | 6.047406 | 1 |
| Xie Lingling 2025 | CT VS HM | 2.214372 | -1.030907 | 5.459651 | 1 |
| Xie Lingling 2025 | CT VS IHM | 1.1759282 | -3.889036 | 6.240892 | 1 |
| Xie Lingling 2025 | CT VS MM | 5.8865309 | 3.650632 | 8.12243 | 1 |
| Xie Lingling 2025 | CT VS TF | 6.3808164 | 3.258915 | 9.502717 | 1 |
| Xie Lingling 2025 | CT VS WDM | 3.8821962 | 0.6958387 | 7.068554 | 1 |
| Xie Lingling 2025 | CT VS WN | 3.3010132 | 1.634995 | 4.967031 | 1 |
| Wang Dan 2021 | CT VS ACU | 0.9063409 | -2.467755 | 3.655074 | 1 |
| Wang Dan 2021 | CT VS EHM | 0.05762075 | -4.870383 | 4.985625 | 1 |
| Wang Dan 2021 | CT VS GIM | 3.4255332 | 0.8712503 | 5.979816 | 1 |
| Wang Dan 2021 | CT VS HM | 2.2145944 | -0.9511079 | 5.380297 | 1 |
| Wang Dan 2021 | CT VS IHM | 0.58030121 | -4.503105 | 5.663707 | 1 |
| Wang Dan 2021 | CT VS MM | 5.290778 | 2.799516 | 7.78204 | 1 |
| Wang Dan 2021 | CT VS TF | 6.3805858 | 3.341592 | 9.41958 | 1 |
| Wang Dan 2021 | CT VS WDM | 4.1980101 | 1.690856 | 6.705164 | 1 |
| Wang Dan 2021 | CT VS WN | 3.3036834 | 1.682138 | 4.925229 | 1 |
| Ji Hua 2025 | CT VS ACU | 0.916482 | -2.568614 | 3.73565 | 1 |
| Ji Hua 2025 | CT VS EHM | 0.20690377 | -4.57428 | 5.160473 | 1 |
| Ji Hua 2025 | CT VS GIM | 3.4270528 | 0.8068131 | 6.047293 | 1 |
| Ji Hua 2025 | CT VS HM | 2.2143724 | -1.030773 | 5.459518 | 1 |
| Ji Hua 2025 | CT VS IHM | 1.1759254 | -3.888825 | 6.240676 | 1 |
| Ji Hua 2025 | CT VS MM | 5.886528 | 3.650726 | 8.12233 | 1 |
| Ji Hua 2025 | CT VS TF | 6.380816 | 3.259054 | 9.502579 | 1 |
| Ji Hua 2025 | CT VS WDM | 3.9334564 | 0.8041005 | 7.062812 | 1 |
| Ji Hua 2025 | CT VS WN | 3.3010175 | 1.635075 | 4.96696 | 1 |
| Bai Fubin 2025 | CT VS ACU | 0.9054907 | -2.459453 | 3.648471 | 1 |
| Bai Fubin 2025 | CT VS EHM | 0.05766146 | -4.859397 | 4.97472 | 1 |
| Bai Fubin 2025 | CT VS GIM | 3.4254051 | 0.8764379 | 5.974372 | 1 |
| Bai Fubin 2025 | CT VS HM | 2.214613 | -0.9446968 | 5.373923 | 1 |
| Bai Fubin 2025 | CT VS IHM | 5.8848552 | -3.711427 | 8.058284 | 1 |
| Bai Fubin 2025 | CT VS MM | 6.3805665 | 3.348241 | 9.412891 | 1 |
| Bai Fubin 2025 | CT VS TF | 4.1980491 | 1.696278 | 6.69982 | 1 |
| Bai Fubin 2025 | CT VS WDM | 3.3039077 | 1.685963 | 4.921852 | 1 |
| Tang Huazhen 2022 | CT VS ACU | 0.9143311 | -2.54663 | 3.717968 | 1 |
| Tang Huazhen 2022 | CT VS EHM | 0.05723853 | -4.976948 | 5.091425 | 1 |
| Tang Huazhen 2022 | CT VS GIM | 3.8742384 | 0.732112 | 7.016365 | 1 |
| Tang Huazhen 2022 | CT VS HM | 2.2144195 | -1.013365 | 5.442204 | 1 |
| Tang Huazhen 2022 | CT VS IHM | 1.1755514 | -3.861395 | 6.212498 | 1 |
| Tang Huazhen 2022 | CT VS MM | 5.8861476 | 3.662969 | 8.109326 | 1 |
| Tang Huazhen 2022 | CT VS TF | 6.3807673 | 3.277077 | 9.484457 | 1 |
| Tang Huazhen 2022 | CT VS WDM | 4.1976449 | 1.638249 | 6.757041 | 1 |
| Tang Huazhen 2022 | CT VS WN | 3.3015814 | 1.645333 | 4.95783 | 1 |
| Wu Naijun 2015 | CT VS ACU | 0.9158482 | -2.562109 | 3.730413 | 1 |
| Wu Naijun 2015 | CT VS EHM | 0.05716601 | -4.99789 | 5.112223 | 1 |
| Wu Naijun 2015 | CT VS GIM | 3.8212905 | 0.5305387 | 7.112042 | 1 |
| Wu Naijun 2015 | CT VS HM | 2.2143862 | -1.025614 | 5.454386 | 1 |
| Wu Naijun 2015 | CT VS IHM | 1.1758152 | -3.880695 | 6.232326 | 1 |
| Wu Naijun 2015 | CT VS MM | 5.8864159 | 3.654354 | 8.118478 | 1 |
| Wu Naijun 2015 | CT VS TF | 6.3808017 | 3.264395 | 9.497209 | 1 |
| Wu Naijun 2015 | CT VS WDM | 4.1975758 | 1.627908 | 6.767244 | 1 |
| Wu Naijun 2015 | CT VS WN | 3.3011836 | 1.638112 | 4.964255 | 1 |
| Xu Xiaowei 2025 | CT VS ACU | 0.9167457 | -2.571331 | 3.73784 | 1 |
| Xu Xiaowei 2025 | CT VS EHM | 0.05712312 | -5.01039 | 5.124637 | 1 |
| Xu Xiaowei 2025 | CT VS GIM | 3.4270921 | 0.80507 | 6.049114 | 1 |
| Xu Xiaowei 2025 | CT VS HM | 2 | -2.640868 | 6.640869 | 1 |
| Xu Xiaowei 2025 | CT VS IHM | 1.1759712 | -3.892219 | 6.244162 | 1 |
| Xu Xiaowei 2025 | CT VS MM | 5.8865747 | 3.649211 | 8.123939 | 1 |
| Xu Xiaowei 2025 | CT VS TF | 6.380822 | 3.256824 | 9.50482 | 1 |
| Xu Xiaowei 2025 | CT VS WDM | 4.197535 | 1.621735 | 6.773335 | 1 |
| Xu Xiaowei 2025 | CT VS WN | 3.3009484 | 1.633806 | 4.968091 | 1 |
| Zhang Suzhao 2024 | CT VS ACU | 0.3285351 | -2.880917 | 3.223847 | 1 |
| Zhang Suzhao 2024 | CT VS EHM | 0.05802249 | -4.764859 | 4.880904 | 1 |
| Zhang Suzhao 2024 | CT VS GIM | 3.424265 | 0.9209694 | 5.927561 | 1 |
| Zhang Suzhao 2024 | CT VS HM | 2.2147775 | -0.8895822 | 5.319137 | 1 |
| Zhang Suzhao 2024 | CT VS IHM | 1.1727056 | -3.666457 | 6.011868 | 1 |
| Zhang Suzhao 2024 | CT VS MM | 5.8832567 | 3.749944 | 8.016569 | 1 |
| Zhang Suzhao 2024 | CT VS TF | 6.3803945 | 3.405442 | 9.355347 | 1 |
| Zhang Suzhao 2024 | CT VS WDM | 4.1983953 | 1.742927 | 6.653863 | 1 |
| Zhang Suzhao 2024 | CT VS WN | 2.8752506 | 1.158043 | 4.592458 | 1 |
| Hu Guanghui 2024 | CT VS ACU | 0.6077698 | -2.240658 | 3.025118 | 1 |
| Hu Guanghui 2024 | CT VS EHM | 0.05756193 | -4.886376 | 5.0015 | 1 |
| Hu Guanghui 2024 | CT VS GIM | 3.4257182 | 0.8637015 | 5.987735 | 1 |
| Hu Guanghui 2024 | CT VS HM | 2.2145675 | -0.9604434 | 5.389578 | 1 |
| Hu Guanghui 2024 | CT VS IHM | 1.174376 | -3.77803 | 6.126782 | 1 |
| Hu Guanghui 2024 | CT VS MM | 5.8849526 | 3.700168 | 8.069737 | 1 |
| Hu Guanghui 2024 | CT VS TF | 6.3806138 | 3.331912 | 9.429316 | 1 |
| Hu Guanghui 2024 | CT VS WDM | 4.1979538 | 1.682962 | 6.712946 | 1 |
| Hu Guanghui 2024 | CT VS WN | 3.6032121 | 1.847092 | 5.359332 | 1 |
| Liu Xiangxiu 2012 | CT VS ACU | 0.8888673 | -2.308792 | 3.531057 | 1 |
| Liu Xiangxiu 2012 | CT VS EHM | 0.05845936 | -4.656995 | 4.773913 | 1 |
| Liu Xiangxiu 2012 | CT VS GIM | 3.4228773 | 0.9716381 | 5.874116 | 1 |
| Liu Xiangxiu 2012 | CT VS HM | 2.2149759 | -0.8268263 | 5.256778 | 1 |
| Liu Xiangxiu 2012 | CT VS IHM | 2.1393966 | -2.755682 | 7.034475 | 1 |
| Liu Xiangxiu 2012 | CT VS MM | 6.8500707 | 4.42848 | 9.271662 | 1 |
| Liu Xiangxiu 2012 | CT VS TF | 6.3801858 | 3.470669 | 9.289702 | 1 |
| Liu Xiangxiu 2012 | CT VS WDM | 4.1988156 | 1.79613 | 6.601501 | 1 |
| Liu Xiangxiu 2012 | CT VS WN | 3.3083179 | 1.756247 | 4.860389 | 1 |
| Xu Lilii 2021 | CT VS ACU | 0.9177869 | -2.582106 | 3.746532 | 1 |
| Xu Lilii 2021 | CT VS EHM | 0.05707337 | -5.024998 | 5.139144 | 1 |
| Xu Lilii 2021 | CT VS GIM | 3.4272472 | 0.798152 | 6.056342 | 1 |
| Xu Lilii 2021 | CT VS HM | 2.2143438 | -1.041475 | 5.470163 | 1 |
| Xu Lilii 2021 | CT VS IHM | 1.1761523 | -3.90569 | 6.257994 | 1 |
| Xu Lilii 2021 | CT VS MM | 5.8867589 | 3.643199 | 8.13032 | 1 |
| Xu Lilii 2021 | CT VS TF | 6.4899998 | 1.969122 | 11.01088 | 1 |
| Xu Lilii 2021 | CT VS WDM | 4.1974876 | 1.614521 | 6.780454 | 1 |
| Xu Lilii 2021 | CT VS WN | 3.3006757 | 1.628776 | 4.972575 | 1 |
| Wu Min 2017 | CT VS ACU | 0.9167457 | -2.571331 | 3.73784 | 1 |
| Wu Min 2017 | CT VS EHM | 0.05712312 | -5.01039 | 5.124637 | 1 |
| Wu Min 2017 | CT VS GIM | 3.4270921 | 0.80507 | 6.049114 | 1 |
| Wu Min 2017 | CT VS HM | 2.4199998 | -2.125351 | 6.965351 | 1 |
| Wu Min 2017 | CT VS IHM | 1.1759712 | -3.892219 | 6.244162 | 1 |
| Wu Min 2017 | CT VS MM | 5.8865747 | 3.649211 | 8.123939 | 1 |
| Wu Min 2017 | CT VS TF | 6.380822 | 3.256824 | 9.50482 | 1 |
| Wu Min 2017 | CT VS WDM | 4.197535 | 1.621735 | 6.773335 | 1 |
| Wu Min 2017 | CT VS WN | 3.3009484 | 1.633806 | 4.968091 | 1 |
| Jin Zhu 2020 | CT VS ACU | 0.6431626 | -2.284162 | 2.997837 | 1 |
| Jin Zhu 2020 | CT VS EHM | 0.05749022 | -4.906072 | 5.021053 | 1 |
| Jin Zhu 2020 | CT VS GIM | 3.4259435 | 0.8544024 | 5.997485 | 1 |
| Jin Zhu 2020 | CT VS HM | 2.2145347 | -0.9719445 | 5.401014 | 1 |
| Jin Zhu 2020 | CT VS IHM | 1.1746365 | -3.796144 | 6.145417 | 1 |
| Jin Zhu 2020 | CT VS MM | 5.8852174 | 3.692086 | 8.078348 | 1 |
| Jin Zhu 2020 | CT VS TF | 6.3806479 | 3.31999 | 9.441306 | 1 |
| Jin Zhu 2020 | CT VS WDM | 4.1978852 | 1.67324 | 6.722531 | 1 |
| Jin Zhu 2020 | CT VS WN | 3.5689176 | 1.815831 | 5.322004 | 1 |
| Wang Yue 2022 | CT VS ACU | 0.8803112 | -2.58645 | 3.825828 | 1 |
| Wang Yue 2022 | CT VS EHM | 0.05713728 | -5.006256 | 5.12053 | 1 |
| Wang Yue 2022 | CT VS GIM | 3.4270479 | 0.8070278 | 6.047068 | 1 |
| Wang Yue 2022 | CT VS HM | 2.2143731 | -1.030508 | 5.459254 | 1 |
| Wang Yue 2022 | CT VS IHM | 1.1759197 | -3.888407 | 6.240247 | 1 |
| Wang Yue 2022 | CT VS MM | 5.8865223 | 3.650913 | 8.122132 | 1 |
| Wang Yue 2022 | CT VS TF | 6.3808153 | 3.259328 | 9.502302 | 1 |
| Wang Yue 2022 | CT VS WDM | 4.1975484 | 1.623777 | 6.77132 | 1 |
| Wang Yue 2022 | CT VS WN | 3.337171 | 1.555501 | 5.118841 | 1 |
| Feng Xueqiang 2024 | CT VS ACU | 1.2553368 | -2.957212 | 5.467886 | 1 |
| Feng Xueqiang 2024 | CT VS EHM | 0.05981074 | -4.362163 | 4.481784 | 1 |
| Feng Xueqiang 2024 | CT VS GIM | 3.418538 | 1.109251 | 5.727825 | 1 |
| Feng Xueqiang 2024 | CT VS HM | 2.2155822 | -0.6560587 | 5.087223 | 1 |
| Feng Xueqiang 2024 | CT VS IHM | 1.1662938 | -3.299284 | 5.631871 | 1 |
| Feng Xueqiang 2024 | CT VS MM | 5.8767647 | 3.91363 | 7.839899 | 1 |
| Feng Xueqiang 2024 | CT VS TF | 6.3795384 | 3.648735 | 9.110342 | 1 |
| Feng Xueqiang 2024 | CT VS WDM | 4.2001238 | 1.941429 | 6.458819 | 1 |
| Feng Xueqiang 2024 | CT VS WN | 3.3157655 | 1.859669 | 4.771862 | 1 |
| Du Xinze 2023 | CT VS ACU | 0.4445933 | -1.967947 | 2.07876 | 1 |
| Du Xinze 2023 | CT VS EHM | 0.05820865 | -4.718057 | 4.834474 | 1 |
| Du Xinze 2023 | CT VS GIM | 3.4236747 | 0.9429713 | 5.904378 | 1 |
| Du Xinze 2023 | CT VS HM | 2.2148622 | -0.8623351 | 5.292059 | 1 |
| Du Xinze 2023 | CT VS IHM | 1.1720321 | -3.623575 | 5.967639 | 1 |
| Du Xinze 2023 | CT VS MM | 5.8825735 | 3.769066 | 7.996081 | 1 |
| Du Xinze 2023 | CT VS TF | 6.3803056 | 3.433749 | 9.326862 | 1 |
| Du Xinze 2023 | CT VS WDM | 4.1985742 | 1.766015 | 6.631134 | 1 |
| Du Xinze 2023 | CT VS WN | 3.7564916 | 2.051862 | 5.461121 | 1 |
| Ma Mengmeng 2024 | CT VS ACU | 0.8179447 | -2.54783 | 3.91194 | 1 |
| Ma Mengmeng 2024 | CT VS EHM | 0.05708897 | -5.020403 | 5.134582 | 1 |
| Ma Mengmeng 2024 | CT VS GIM | 3.4271985 | 0.800328 | 6.054069 | 1 |
| Ma Mengmeng 2024 | CT VS HM | 2.2143509 | -1.038786 | 5.467488 | 1 |
| Ma Mengmeng 2024 | CT VS IHM | 1.1760955 | -3.901453 | 6.253644 | 1 |
| Ma Mengmeng 2024 | CT VS MM | 5.8867011 | 3.64509 | 8.128313 | 1 |
| Ma Mengmeng 2024 | CT VS TF | 6.3808382 | 3.250759 | 9.510917 | 1 |
| Ma Mengmeng 2024 | CT VS WDM | 4.1975024 | 1.61679 | 6.778214 | 1 |
| Ma Mengmeng 2024 | CT VS WN | 3.4002953 | 1.585008 | 5.215582 | 1 |
| Li Xiaoting 2014 | CT VS ACU | 0.9164057 | -2.567838 | 3.735027 | 1 |
| Li Xiaoting 2014 | CT VS EHM | 0.05713937 | -5.005646 | 5.119925 | 1 |
| Li Xiaoting 2014 | CT VS GIM | 3.4270414 | 0.8073164 | 6.046766 | 1 |
| Li Xiaoting 2014 | CT VS HM | 2.214374 | -1.030151 | 5.458899 | 1 |
| Li Xiaoting 2014 | CT VS IHM | 0.94127415 | -4.299412 | 6.18196 | 1 |
| Li Xiaoting 2014 | CT VS MM | 5.6518365 | 3.04021 | 8.263462 | 1 |
| Li Xiaoting 2014 | CT VS TF | 6.3808143 | 3.259698 | 9.50193 | 1 |
| Li Xiaoting 2014 | CT VS WDM | 4.1975504 | 1.624078 | 6.771023 | 1 |
| Li Xiaoting 2014 | CT VS WN | 3.3010375 | 1.63544 | 4.966635 | 1 |
| Luo Xiaohong 2022 | CT VS ACU | 0.9177869 | -2.582106 | 3.746532 | 1 |
| Luo Xiaohong 2022 | CT VS EHM | 0.05707337 | -5.024998 | 5.139144 | 1 |
| Luo Xiaohong 2022 | CT VS GIM | 3.4272472 | 0.798152 | 6.056342 | 1 |
| Luo Xiaohong 2022 | CT VS HM | 2.2143438 | -1.041475 | 5.470163 | 1 |
| Luo Xiaohong 2022 | CT VS IHM | 1.1761523 | -3.90569 | 6.257994 | 1 |
| Luo Xiaohong 2022 | CT VS MM | 5.8867589 | 3.643199 | 8.13032 | 1 |
| Luo Xiaohong 2022 | CT VS TF | 6.2799997 | 1.934583 | 10.62542 | 1 |
| Luo Xiaohong 2022 | CT VS WDM | 4.1974876 | 1.614521 | 6.780454 | 1 |
| Luo Xiaohong 2022 | CT VS WN | 3.3006757 | 1.628776 | 4.972575 | 1 |
| Wang Jiawei 2023 | CT VS ACU | 0.9167471 | -2.571348 | 3.737854 | 1 |
| Wang Jiawei 2023 | CT VS EHM | 0.05712305 | -5.010411 | 5.124657 | 1 |
| Wang Jiawei 2023 | CT VS GIM | 3.4270923 | 0.8050603 | 6.049124 | 1 |
| Wang Jiawei 2023 | CT VS HM | 2.2143666 | -1.032939 | 5.461672 | 1 |
| Wang Jiawei 2023 | CT VS IHM | 1.0548036 | -4.167108 | 6.276715 | 1 |
| Wang Jiawei 2023 | CT VS MM | 5.7653863 | 3.19868 | 8.332093 | 1 |
| Wang Jiawei 2023 | CT VS TF | 6.380822 | 3.256812 | 9.504832 | 1 |
| Wang Jiawei 2023 | CT VS WDM | 4.1975349 | 1.621725 | 6.773345 | 1 |
| Wang Jiawei 2023 | CT VS WN | 3.300948 | 1.633799 | 4.968097 | 1 |
| Peng Jing 2022 | CT VS ACU | 0.905492 | -2.459466 | 3.648481 | 1 |
| Peng Jing 2022 | CT VS EHM | 3.4254053 | -0.8764298 | 5.974381 | 1 |
| Peng Jing 2022 | CT VS GIM | 2.2146129 | 0.9447068 | 5.373933 | 1 |
| Peng Jing 2022 | CT VS HM | 1.1740148 | -3.753243 | 6.101273 | 1 |
| Peng Jing 2022 | CT VS IHM | 5.8845857 | -3.711236 | 8.057936 | 1 |
| Peng Jing 2022 | CT VS MM | 6.3805665 | 3.348231 | 9.412902 | 1 |
| Peng Jing 2022 | CT VS TF | 4.1983194 | 1.696381 | 6.700257 | 1 |
| Peng Jing 2022 | CT VS WDM | 3.3039073 | 1.685957 | 4.921857 | 1 |
| Peng Jing 2022 | CT VS ACU | 0.9127567 | -2.530766 | 3.705252 | 1 |
| Liang Guoyu 2025 | CT VS EHM | 0.62968653 | -4.691185 | 5.950559 | 1 |
| Liang Guoyu 2025 | CT VS GIM | 3.4264964 | 0.8310628 | 6.02193 | 1 |
| Liang Guoyu 2025 | CT VS HM | 2.214454 | -1.000801 | 5.429709 | 1 |
| Liang Guoyu 2025 | CT VS IHM | 1.1752777 | -3.841602 | 6.192157 | 1 |
| Liang Guoyu 2025 | CT VS MM | 5.8858692 | 3.671802 | 8.099936 | 1 |
| Liang Guoyu 2025 | CT VS TF | 6.3807316 | 3.290089 | 9.471375 | 1 |
| Liang Guoyu 2025 | CT VS WDM | 4.7701979 | 1.65866 | 7.881735 | 1 |
| Liang Guoyu 2025 | CT VS WN | 3.3019948 | 1.652742 | 4.951247 | 1 |
| Hu Guanghui 2023 | CT VS ACU | 0.9074728 | -2.478671 | 3.663725 | 1 |
| Hu Guanghui 2023 | CT VS EHM | 0.05756657 | -4.88511 | 5.000243 | 1 |
| Hu Guanghui 2023 | CT VS GIM | 2.6082913 | 0.495698 | 5.712281 | 1 |
| Hu Guanghui 2023 | CT VS HM | 2.2145696 | -0.9597042 | 5.388844 | 1 |
| Hu Guanghui 2023 | CT VS IHM | 1.1743591 | -3.776865 | 6.125583 | 1 |
| Hu Guanghui 2023 | CT VS MM | 5.8849355 | 3.70069 | 8.069181 | 1 |
| Hu Guanghui 2023 | CT VS TF | 6.3806116 | 3.332679 | 9.428544 | 1 |
| Hu Guanghui 2023 | CT VS WDM | 4.1979582 | 1.683587 | 6.712329 | 1 |
| Hu Guanghui 2023 | CT VS WN | 3.303385 | 1.677045 | 4.929725 | 1 |

Leave-one-out sensitivity analysis of median MNCV in the subgroup analysis of patients with DPN (Table S142)

| **dropped_id** | **comparison** | **eff** | **lci** | **uci** | **connected** |
| --- | --- | --- | --- | --- | --- |
| Feng Yun 2022 | CT VS ACU | 0.841549 | -2.471057 | 3.7879592 | 1 |
| Feng Yun 2022 | CT VS EHM | 0.25066581 | -4.815955 | 4.914623 | 1 |
| Feng Yun 2022 | CT VS GIM | 4.778935 | 1.92514 | 7.63273 | 1 |
| Feng Yun 2022 | CT VS HM | 3.3699999 | -0.5197504 | 7.25975 | 1 |
| Feng Yun 2022 | CT VS MM | 4.6208987 | 1.890274 | 7.351523 | 1 |
| Feng Yun 2022 | CT VS TF | 4.4914978 | 2.365733 | 6.617262 | 1 |
| Feng Yun 2022 | CT VS WDM | 4.4995237 | 1.867748 | 7.131299 | 1 |
| Feng Yun 2022 | CT VS WN | 1.8483695 | 0.3287261 | 4.025465 | 1 |
| Xie Lingling 2025 | CT VS ACU | 0.3215711 | -3.566353 | 3.923211 | 1 |
| Xie Lingling 2025 | CT VS EHM | 0.48841854 | -7.117282 | 8.094119 | 1 |
| Xie Lingling 2025 | CT VS GIM | 4.7770427 | 0.8158419 | 8.738243 | 1 |
| Xie Lingling 2025 | CT VS HM | 3.3699999 | -2.127585 | 8.867584 | 1 |
| Xie Lingling 2025 | CT VS MM | 4.6154172 | 0.7419494 | 8.488885 | 1 |
| Xie Lingling 2025 | CT VS TF | 4.4942698 | 1.403859 | 7.584681 | 1 |
| Xie Lingling 2025 | CT VS WDM | 5.2389489 | 0.1322048 | 10.6101 | 1 |
| Xie Lingling 2025 | CT VS WN | 3.3685088 | 0.6825098 | 6.054508 | 1 |
| Wang Dan 2021 | CT VS ACU | 0.3213545 | -3.561131 | 3.918423 | 1 |
| Wang Dan 2021 | CT VS EHM | 0.25352364 | -6.841291 | 6.934244 | 1 |
| Wang Dan 2021 | CT VS GIM | 4.7770459 | 0.8190026 | 8.735089 | 1 |
| Wang Dan 2021 | CT VS HM | 3.3699999 | -2.123034 | 8.863034 | 1 |
| Wang Dan 2021 | CT VS MM | 3.9000001 | 1.594374 | 9.394374 | 1 |
| Wang Dan 2021 | CT VS TF | 4.4942655 | 1.406553 | 7.581978 | 1 |
| Wang Dan 2021 | CT VS WDM | 4.4968686 | 0.6960847 | 8.297652 | 1 |
| Wang Dan 2021 | CT VS WN | 3.3687253 | 0.6850511 | 6.0524 | 1 |
| Ji Hua 2025 | CT VS ACU | 0.3215711 | -3.566353 | 3.92321 | 1 |
| Ji Hua 2025 | CT VS EHM | 0.2007623 | -3.617959 | 6.916435 | 1 |
| Ji Hua 2025 | CT VS GIM | 4.7770427 | 0.8158419 | 8.738243 | 1 |
| Ji Hua 2025 | CT VS HM | 3.3699999 | -2.127585 | 8.867584 | 1 |
| Ji Hua 2025 | CT VS MM | 4.6154172 | 0.7419495 | 8.488885 | 1 |
| Ji Hua 2025 | CT VS TF | 4.4942698 | 1.403859 | 7.584681 | 1 |
| Ji Hua 2025 | CT VS WDM | 3.7494923 | 1.637935 | 9.13692 | 1 |
| Ji Hua 2025 | CT VS WN | 3.3685088 | 0.6825098 | 6.054508 | 1 |
| Tang Huazhen 2022 | CT VS ACU | 0.3220772 | -3.578601 | 3.934447 | 1 |
| Tang Huazhen 2022 | CT VS EHM | 0.25354118 | -6.06035 | 6.353268 | 1 |
| Tang Huazhen 2022 | CT VS GIM | 5.2000003 | 0.3990793 | 10.79908 | 1 |
| Tang Huazhen 2022 | CT VS HM | 3.3699999 | -2.138267 | 8.878266 | 1 |
| Tang Huazhen 2022 | CT VS MM | 4.6153961 | 0.7343476 | 8.496445 | 1 |
| Tang Huazhen 2022 | CT VS TF | 4.4942797 | 1.397534 | 7.591025 | 1 |
| Tang Huazhen 2022 | CT VS WDM | 4.4968534 | 0.6850671 | 8.30864 | 1 |
| Tang Huazhen 2022 | CT VS WN | 3.3680029 | 0.6765615 | 6.059444 | 1 |
| Zhang Xiujun 2020 | CT VS ACU | 0.3191163 | -3.50797 | 3.869738 | 1 |
| Zhang Xiujun 2020 | CT VS EHM | 0.25346967 | -6.083312 | 6.276373 | 1 |
| Zhang Xiujun 2020 | CT VS GIM | 4.7770796 | 0.8511786 | 8.702981 | 1 |
| Zhang Xiujun 2020 | CT VS HM | 3.3699999 | -2.076708 | 8.816708 | 1 |
| Zhang Xiujun 2020 | CT VS MM | 4.6155196 | 0.7781599 | 8.452879 | 1 |
| Zhang Xiujun 2020 | CT VS TF | 5.0945094 | 1.353517 | 8.835503 | 1 |
| Zhang Xiujun 2020 | CT VS WDM | 4.4969158 | 0.7296029 | 8.264229 | 1 |
| Zhang Xiujun 2020 | CT VS WN | 3.3709625 | 0.7109144 | 6.031011 | 1 |
| Xu Xiaowei 2025 | CT VS ACU | 0.3069809 | -3.242034 | 3.628072 | 1 |
| Xu Xiaowei 2025 | CT VS EHM | 0.25318502 | -6.49396 | 6.98759 | 1 |
| Xu Xiaowei 2025 | CT VS GIM | 4.7772601 | 1.011417 | 8.543103 | 1 |
| Xu Xiaowei 2025 | CT VS HM | 4.6160231 | -0.9425831 | 8.289463 | 1 |
| Xu Xiaowei 2025 | CT VS MM | 4.4939816 | 1.570929 | 7.417035 | 1 |
| Xu Xiaowei 2025 | CT VS TF | 4.4971673 | 0.8968936 | 8.097441 | 1 |
| Xu Xiaowei 2025 | CT VS WDM | 3.3830935 | 0.8413849 | 5.924802 | 1 |
| Zhang Suzhao 2024 | CT VS ACU | 0.831006 | -2.194556 | 3.532544 | 1 |
| Zhang Suzhao 2024 | CT VS EHM | 0.25344963 | -6.062046 | 6.255147 | 1 |
| Zhang Suzhao 2024 | CT VS GIM | 4.7770921 | 0.8629748 | 8.69121 | 1 |
| Zhang Suzhao 2024 | CT VS HM | 3.3699999 | -2.05972 | 8.79972 | 1 |
| Zhang Suzhao 2024 | CT VS MM | 4.6155545 | 0.7902516 | 8.440857 | 1 |
| Zhang Suzhao 2024 | CT VS TF | 4.4942049 | 1.444052 | 7.544358 | 1 |
| Zhang Suzhao 2024 | CT VS WDM | 4.4969333 | 0.7418972 | 8.251969 | 1 |
| Zhang Suzhao 2024 | CT VS WN | 2.8589957 | 0.2157677 | 5.933759 | 1 |
| Zhang Xiujun 2020 | CT VS ACU | 0.3223475 | -3.585178 | 3.940484 | 1 |
| Zhang Xiujun 2020 | CT VS EHM | 0.25354776 | -6.067523 | 6.360427 | 1 |
| Zhang Xiujun 2020 | CT VS GIM | 4.777031 | 0.8044382 | 8.749623 | 1 |
| Zhang Xiujun 2020 | CT VS HM | 3.3699999 | -2.144 | 8.884 | 1 |
| Zhang Xiujun 2020 | CT VS MM | 4.6153848 | 0.7302675 | 8.500502 | 1 |
| Zhang Xiujun 2020 | CT VS TF | 4.8777874 | 1.070245 | 8.68533 | 1 |
| Zhang Xiujun 2020 | CT VS WDM | 4.4968476 | 0.6809204 | 8.312775 | 1 |
| Zhang Xiujun 2020 | CT VS WN | 3.3677327 | 0.67336 | 6.062105 | 1 |
| Hu Guanghui 2024 | CT VS ACU | 0.2453109 | -3.038652 | 3.248031 | 1 |
| Hu Guanghui 2024 | CT VS EHM | 0.25207718 | -4.593926 | 5.089772 | 1 |
| Hu Guanghui 2024 | CT VS GIM | 4.7779926 | 1.505456 | 8.050529 | 1 |
| Hu Guanghui 2024 | CT VS HM | 3.3699999 | -1.131171 | 7.871171 | 1 |
| Hu Guanghui 2024 | CT VS MM | 4.6181095 | 1.45237 | 7.783849 | 1 |
| Hu Guanghui 2024 | CT VS TF | 4.492956 | 1.997089 | 6.988823 | 1 |
| Hu Guanghui 2024 | CT VS WDM | 4.4981815 | 1.417441 | 7.578922 | 1 |
| Hu Guanghui 2024 | CT VS WN | 4.6448793 | 2.155417 | 7.134342 | 1 |
| Liu Xiangxiu 2012 | CT VS ACU | 0.3213545 | -3.561131 | 3.918423 | 1 |
| Liu Xiangxiu 2012 | CT VS EHM | 0.25352364 | -6.041291 | 6.334244 | 1 |
| Liu Xiangxiu 2012 | CT VS GIM | 4.7770459 | 0.8190026 | 8.735089 | 1 |
| Liu Xiangxiu 2012 | CT VS HM | 3.3699999 | -2.123034 | 8.863034 | 1 |
| Liu Xiangxiu 2012 | CT VS MM | 5.3200002 | 0.1325426 | 10.77254 | 1 |
| Liu Xiangxiu 2012 | CT VS TF | 4.4942655 | 1.406553 | 7.581978 | 1 |
| Liu Xiangxiu 2012 | CT VS WDM | 4.4968686 | 0.6960847 | 8.297652 | 1 |
| Liu Xiangxiu 2012 | CT VS WN | 3.3687253 | 0.6850511 | 6.0524 | 1 |
| Xu Lilii 2021 | CT VS ACU | 0.3094822 | -3.294138 | 3.675173 | 1 |
| Xu Lilii 2021 | CT VS EHM | 0.25324268 | -5.550397 | 6.043912 | 1 |
| Xu Lilii 2021 | CT VS GIM | 4.7772231 | 0.9802092 | 8.574237 | 1 |
| Xu Lilii 2021 | CT VS HM | 3.3699999 | -1.89079 | 8.63079 | 1 |
| Xu Lilii 2021 | CT VS MM | 4.6159197 | 0.9105302 | 8.321309 | 1 |
| Xu Lilii 2021 | CT VS TF | 3.5123467 | -0.0965807 | 7.121274 | 1 |
| Xu Lilii 2021 | CT VS WDM | 4.497116 | 0.8642623 | 8.12997 | 1 |
| Xu Lilii 2021 | CT VS WN | 3.380593 | 0.8154035 | 5.945783 | 1 |
| Jin Zhu 2020 | CT VS ACU | 0.4694346 | -3.408318 | 4.469448 | 1 |
| Jin Zhu 2020 | CT VS EHM | 0.25299301 | -5.313599 | 5.807613 | 1 |
| Jin Zhu 2020 | CT VS GIM | 4.7773843 | 1.110988 | 8.443781 | 1 |
| Jin Zhu 2020 | CT VS HM | 3.3699999 | -1.702106 | 8.442106 | 1 |
| Jin Zhu 2020 | CT VS MM | 4.6163721 | 1.044955 | 8.18779 | 1 |
| Jin Zhu 2020 | CT VS TF | 4.4938138 | 1.656303 | 7.331325 | 1 |
| Jin Zhu 2020 | CT VS WDM | 4.4973393 | 1.001183 | 7.993495 | 1 |
| Jin Zhu 2020 | CT VS WN | 4.2207465 | 1.395145 | 7.046348 | 1 |
| Feng Xueqiang 2024 | ACU VS CT | 0.25318514 | -5.494074 | 5.987704 | 1 |
| Feng Xueqiang 2024 | ACU VS EHM | 4.77726 | -1.011353 | 8.543166 | 1 |
| Feng Xueqiang 2024 | ACU VS GIM | 3.3699999 | 1.845879 | 8.585879 | 1 |
| Feng Xueqiang 2024 | ACU VS HM | 4.6160229 | -0.9425184 | 8.289528 | 1 |
| Feng Xueqiang 2024 | ACU VS MM | 4.4939818 | -1.570875 | 7.417089 | 1 |
| Feng Xueqiang 2024 | ACU VS TF | 4.4971672 | -0.8968277 | 8.097507 | 1 |
| Feng Xueqiang 2024 | ACU VS WDM | 3.3831213 | -0.8412895 | 5.924953 | 1 |
| Feng Xueqiang 2024 | CT VS ACU | 0.3069894 | -3.242206 | 3.628227 | 1 |
| Peng Jing 2022 | CT VS EHM | 4.7772599 | -1.011313 | 8.543206 | 1 |
| Peng Jing 2022 | CT VS GIM | 3.3699999 | 1.845937 | 8.585937 | 1 |
| Peng Jing 2022 | CT VS HM | 4.6160228 | -0.9424772 | 8.289568 | 1 |
| Peng Jing 2022 | CT VS MM | 4.4939818 | 1.57084 | 7.417123 | 1 |
| Peng Jing 2022 | CT VS TF | 4.4975171 | 0.8968391 | 8.098195 | 1 |
| Peng Jing 2022 | CT VS WDM | 3.383085 | 0.8413 | 5.92487 | 1 |
| Hu Guanghui 2023 | CT VS ACU | 0.3220772 | -3.578601 | 3.934447 | 1 |
| Hu Guanghui 2023 | CT VS EHM | 0.25354118 | -6.06035 | 6.353268 | 1 |
| Hu Guanghui 2023 | CT VS GIM | 4.3500004 | 1.27595 | 9.97595 | 1 |
| Hu Guanghui 2023 | CT VS HM | 3.3699999 | -2.138267 | 8.878266 | 1 |
| Hu Guanghui 2023 | CT VS MM | 4.6153961 | 0.7343476 | 8.496445 | 1 |
| Hu Guanghui 2023 | CT VS TF | 4.4942797 | 1.397534 | 7.591025 | 1 |
| Hu Guanghui 2023 | CT VS WDM | 4.4968534 | 0.6850671 | 8.30864 | 1 |
| Hu Guanghui 2023 | CT VS WN | 3.3680029 | 0.6765614 | 6.059444 | 1 |

Leave-one-out sensitivity analysis of common peroneal SNCV in the subgroup analysis of patients with DPN (Table S143)

| **dropped_id** | **comparison** | **eff** | **lci** | **uci** | **connected** |
| --- | --- | --- | --- | --- | --- |
| Feng Yun 2022 | CT VS ACU | 3.0856623 | -1.496495 | 7.667819 | 1 |
| Feng Yun 2022 | CT VS EHM | -0.39301474 | -4.023075 | 3.237046 | 1 |
| Feng Yun 2022 | CT VS GIM | 3.9699998 | -0.0443387 | 7.895661 | 1 |
| Feng Yun 2022 | CT VS HM | 1.5600004 | -2.017042 | 5.137043 | 1 |
| Feng Yun 2022 | CT VS IHM | 1.7459611 | -2.882288 | 6.37421 | 1 |
| Feng Yun 2022 | CT VS MM | 5.4163568 | 2.691162 | 8.141551 | 1 |
| Feng Yun 2022 | CT VS TF | 6.0100002 | 2.514476 | 9.505525 | 1 |
| Feng Yun 2022 | CT VS WDM | 3.4827172 | -0.9056883 | 6.059746 | 1 |
| Feng Yun 2022 | CT VS WN | 2.9990234 | 1.396885 | 4.601161 | 1 |
| Wang Dan 2021 | CT VS ACU | 4.3412019 | -3.504306 | 12.18671 | 1 |
| Wang Dan 2021 | CT VS EHM | -0.38981074 | -5.820539 | 5.040917 | 1 |
| Wang Dan 2021 | CT VS GIM | 3.9699998 | -1.663806 | 9.603806 | 1 |
| Wang Dan 2021 | CT VS HM | 1.5600004 | -3.836682 | 6.956683 | 1 |
| Wang Dan 2021 | CT VS IHM | 3.0020173 | -4.871012 | 10.87505 | 1 |
| Wang Dan 2021 | CT VS MM | 6.6732016 | 1.044849 | 12.30155 | 1 |
| Wang Dan 2021 | CT VS TF | 6.0100002 | 0.6670008 | 11.353 | 1 |
| Wang Dan 2021 | CT VS WDM | 3.4886889 | -0.3584269 | 7.335805 | 1 |
| Wang Dan 2021 | CT VS WN | 4.0672903 | 1.868732 | 6.265848 | 1 |
| Ji Hua 2025 | CT VS ACU | 3.1063203 | -3.784173 | 9.996814 | 1 |
| Ji Hua 2025 | CT VS EHM | -0.83992415 | -7.616888 | 5.93704 | 1 |
| Ji Hua 2025 | CT VS GIM | 3.9699998 | -1.78143 | 9.72143 | 1 |
| Ji Hua 2025 | CT VS HM | 1.5600004 | -3.959363 | 7.079363 | 1 |
| Ji Hua 2025 | CT VS IHM | 1.76702 | -5.154555 | 8.688596 | 1 |
| Ji Hua 2025 | CT VS MM | 5.4379415 | 1.406071 | 9.469812 | 1 |
| Ji Hua 2025 | CT VS TF | 6.0100002 | 0.5431157 | 11.47688 | 1 |
| Ji Hua 2025 | CT VS WDM | 3.0385344 | -2.491047 | 8.568116 | 1 |
| Ji Hua 2025 | CT VS WN | 4.0657176 | 1.816882 | 6.314554 | 1 |
| Bai Fubin 2025 | CT VS ACU | 3.1053898 | -3.407414 | 9.618194 | 1 |
| Bai Fubin 2025 | CT VS EHM | -0.38995144 | -5.629591 | 4.849689 | 1 |
| Bai Fubin 2025 | CT VS GIM | 3.9699998 | -1.479602 | 9.419601 | 1 |
| Bai Fubin 2025 | CT VS HM | 1.5600004 | -3.644092 | 6.764092 | 1 |
| Bai Fubin 2025 | CT VS IHM | 5.436834 | 1.619663 | 9.254004 | 1 |
| Bai Fubin 2025 | CT VS MM | 6.0100002 | 0.8615993 | 11.1584 | 1 |
| Bai Fubin 2025 | CT VS TF | 3.4883649 | -0.2238605 | 7.20059 | 1 |
| Bai Fubin 2025 | CT VS WDM | 4.0699859 | 1.950423 | 6.189548 | 1 |
| Tang Huazhen 2022 | CT VS ACU | 3.1045257 | -3.407759 | 9.616811 | 1 |
| Tang Huazhen 2022 | CT VS EHM | -0.3899516 | -3.643883 | 6.763885 | 1 |
| Tang Huazhen 2022 | CT VS GIM | 1.5600004 | -3.643883 | 6.763885 | 1 |
| Tang Huazhen 2022 | CT VS HM | 1.7651487 | -4.779922 | 8.31022 | 1 |
| Tang Huazhen 2022 | CT VS IHM | 5.4359696 | 1.619402 | 9.252538 | 1 |
| Tang Huazhen 2022 | CT VS MM | 6.0100002 | 0.8618095 | 11.15819 | 1 |
| Tang Huazhen 2022 | CT VS TF | 3.4883645 | -0.2237152 | 7.200444 | 1 |
| Tang Huazhen 2022 | CT VS WDM | 4.069989 | 1.950512 | 6.189466 | 1 |
| Zhang Suzhao 2024 | CT VS ACU | 3.1045302 | -3.408601 | 9.617661 | 1 |
| Zhang Suzhao 2024 | CT VS EHM | -0.38995103 | -5.630084 | 4.850182 | 1 |
| Zhang Suzhao 2024 | CT VS GIM | 3.9699998 | -1.480076 | 9.420075 | 1 |
| Zhang Suzhao 2024 | CT VS HM | 1.5600004 | -3.644588 | 6.764589 | 1 |
| Zhang Suzhao 2024 | CT VS IHM | 1.7651534 | -4.78076 | 8.311067 | 1 |
| Zhang Suzhao 2024 | CT VS MM | 5.4359744 | 1.618922 | 9.253027 | 1 |
| Zhang Suzhao 2024 | CT VS TF | 6.0100002 | 0.8610974 | 11.1589 | 1 |
| Zhang Suzhao 2024 | CT VS WDM | 3.4883658 | -0.2242076 | 7.200939 | 1 |
| Zhang Suzhao 2024 | CT VS WN | 3.5764798 | 1.2514 | 5.901559 | 1 |
| Hu Guanghui 2024 | CT VS ACU | 3.1025135 | -3.065155 | 9.270182 | 1 |
| Hu Guanghui 2024 | CT VS EHM | -0.39022246 | -5.34392 | 4.563476 | 1 |
| Hu Guanghui 2024 | CT VS GIM | 3.9699998 | -1.20495 | 9.14495 | 1 |
| Hu Guanghui 2024 | CT VS HM | 1.5600004 | -3.355742 | 6.475742 | 1 |
| Hu Guanghui 2024 | CT VS IHM | 1.7630704 | -4.439132 | 7.965272 | 1 |
| Hu Guanghui 2024 | CT VS MM | 5.4338045 | 1.813098 | 9.054511 | 1 |
| Hu Guanghui 2024 | CT VS TF | 6.0100002 | 1.153254 | 10.86675 | 1 |
| Hu Guanghui 2024 | CT VS WDM | 3.4877885 | -0.0226249 | 6.998202 | 1 |
| Hu Guanghui 2024 | CT VS WN | 4.7157961 | 2.522719 | 6.908873 | 1 |
| Xu Lilii 2021 | CT VS ACU | 3.1045257 | -3.407759 | 9.616811 | 1 |
| Xu Lilii 2021 | CT VS EHM | -0.3899516 | -5.629385 | 4.849482 | 1 |
| Xu Lilii 2021 | CT VS GIM | 3.9699998 | -1.479403 | 9.419403 | 1 |
| Xu Lilii 2021 | CT VS HM | 1.5600004 | -3.643883 | 6.763885 | 1 |
| Xu Lilii 2021 | CT VS IHM | 1.7651487 | -4.779922 | 8.31022 | 1 |
| Xu Lilii 2021 | CT VS MM | 5.4359696 | 1.619402 | 9.252538 | 1 |
| Xu Lilii 2021 | CT VS TF | 3.4883645 | 0.2237152 | 7.200444 | 1 |
| Xu Lilii 2021 | CT VS WDM | 4.069989 | -1.950512 | 6.189466 | 1 |
| Du Minzhen 2019 | CT VS ACU | 3.1050901 | -3.517482 | 9.727662 | 1 |
| Du Minzhen 2019 | CT VS EHM | -0.38988059 | -5.720654 | 4.940893 | 1 |
| Du Minzhen 2019 | CT VS GIM | 3.9699998 | -1.56739 | 9.50739 | 1 |
| Du Minzhen 2019 | CT VS HM | 1.5600004 | -3.735951 | 6.855952 | 1 |
| Du Minzhen 2019 | CT VS IHM | 1.765735 | -4.889105 | 8.420575 | 1 |
| Du Minzhen 2019 | CT VS MM | 5.4365846 | 1.557265 | 9.315904 | 1 |
| Du Minzhen 2019 | CT VS TF | 6.0100002 | 0.7687634 | 11.25124 | 1 |
| Du Minzhen 2019 | CT VS WDM | 3.488525 | -0.2880301 | 7.26508 | 1 |
| Du Minzhen 2019 | CT VS WN | 4.4747001 | 2.113324 | 6.836076 | 1 |
| Jin Zhu 2020 | CT VS ACU | 3.1062312 | -3.763387 | 9.975849 | 1 |
| Jin Zhu 2020 | CT VS EHM | -0.3897457 | -5.924969 | 5.145477 | 1 |
| Jin Zhu 2020 | CT VS GIM | 3.9699998 | -1.76474 | 9.704739 | 1 |
| Jin Zhu 2020 | CT VS HM | 1.5600004 | -3.941967 | 7.061968 | 1 |
| Jin Zhu 2020 | CT VS IHM | 1.7669265 | -5.133862 | 8.667715 | 1 |
| Jin Zhu 2020 | CT VS MM | 5.4378423 | 1.417863 | 9.457821 | 1 |
| Jin Zhu 2020 | CT VS TF | 6.0100002 | 0.5606782 | 11.45932 | 1 |
| Jin Zhu 2020 | CT VS WDM | 3.4888484 | -0.4320386 | 7.409735 | 1 |
| Jin Zhu 2020 | CT VS WN | 4.1610106 | 1.728686 | 6.593336 | 1 |
| Luo Xingzi 2020 | ACU VS CT | 0.38995135 | -3.629693 | 4.84979 | 1 |
| Luo Xingzi 2020 | ACU VS EHM | 3.9699998 | -1.479699 | 9.419699 | 1 |
| Luo Xingzi 2020 | ACU VS GIM | 1.5600004 | -3.644194 | 6.764194 | 1 |
| Luo Xingzi 2020 | ACU VS HM | 1.7666732 | -4.779167 | 8.312513 | 1 |
| Luo Xingzi 2020 | ACU VS IHM | 5.4374945 | -1.620029 | 9.25496 | 1 |
| Luo Xingzi 2020 | ACU VS MM | 6.0100002 | -0.8614961 | 11.1585 | 1 |
| Luo Xingzi 2020 | ACU VS TF | 3.4883651 | -0.2239318 | 7.200662 | 1 |
| Luo Xingzi 2020 | ACU VS WDM | 4.0699844 | -1.95038 | 6.189589 | 1 |
| Ma Mengmeng 2024 | CT VS ACU | 3.1052116 | -3.542042 | 9.752465 | 1 |
| Ma Mengmeng 2024 | CT VS EHM | -0.38986565 | -5.741075 | 4.961344 | 1 |
| Ma Mengmeng 2024 | CT VS GIM | 3.9699998 | -1.587092 | 9.527091 | 1 |
| Ma Mengmeng 2024 | CT VS HM | 1.5600004 | -3.756548 | 6.876548 | 1 |
| Ma Mengmeng 2024 | CT VS IHM | 1.7658615 | -4.913547 | 8.44527 | 1 |
| Ma Mengmeng 2024 | CT VS MM | 5.4367176 | 1.543351 | 9.330084 | 1 |
| Ma Mengmeng 2024 | CT VS TF | 6.0100002 | 0.7479529 | 11.27205 | 1 |
| Ma Mengmeng 2024 | CT VS WDM | 3.4885595 | -0.3024215 | 7.279541 | 1 |
| Ma Mengmeng 2024 | CT VS WN | 4.4709908 | 2.085994 | 6.855988 | 1 |
| Wang Jiawei 2023 | CT VS ACU | 1.9053668 | -5.874137 | 9.684871 | 1 |
| Wang Jiawei 2023 | CT VS EHM | -0.38981074 | -5.820537 | 5.040915 | 1 |
| Wang Jiawei 2023 | CT VS GIM | 3.9699998 | -1.663804 | 9.603804 | 1 |
| Wang Jiawei 2023 | CT VS HM | 1.5600004 | -3.83668 | 6.956681 | 1 |
| Wang Jiawei 2023 | CT VS IHM | 0.56589431 | -7.241348 | 8.373137 | 1 |
| Wang Jiawei 2023 | CT VS MM | 4.2364819 | 1.299432 | 9.772395 | 1 |
| Wang Jiawei 2023 | CT VS TF | 6.0100002 | 0.6670029 | 11.353 | 1 |
| Wang Jiawei 2023 | CT VS WDM | 3.4886889 | -0.3584255 | 7.335804 | 1 |
| Wang Jiawei 2023 | CT VS WN | 4.0672904 | 1.868733 | 6.265848 | 1 |
| Liu Maomao 2023 | CT VS ACU | 3.1045257 | -3.407759 | 9.616811 | 1 |
| Liu Maomao 2023 | CT VS EHM | -0.3899516 | -5.629385 | 4.849482 | 1 |
| Liu Maomao 2023 | CT VS GIM | 3.9699998 | -1.479403 | 9.419403 | 1 |
| Liu Maomao 2023 | CT VS HM | 1.7651487 | -4.779922 | 8.31022 | 1 |
| Liu Maomao 2023 | CT VS IHM | 5.4359696 | -1.619402 | 9.252538 | 1 |
| Liu Maomao 2023 | CT VS MM | 6.0100002 | 0.8618095 | 11.15819 | 1 |
| Liu Maomao 2023 | CT VS TF | 3.4883645 | 0.2237152 | 7.200444 | 1 |
| Liu Maomao 2023 | CT VS WDM | 4.069989 | -1.950512 | 6.189466 | 1 |
| Huang Xingduan 2024 | CT VS ACU | 3.1064032 | -3.803742 | 10.01655 | 1 |
| Huang Xingduan 2024 | CT VS EHM | -0.14085996 | -6.941673 | 6.659953 | 1 |
| Huang Xingduan 2024 | CT VS GIM | 3.9699998 | -1.797146 | 9.737145 | 1 |
| Huang Xingduan 2024 | CT VS HM | 1.5600004 | -3.975736 | 7.095737 | 1 |
| Huang Xingduan 2024 | CT VS IHM | 1.7671071 | -5.174037 | 8.708252 | 1 |
| Huang Xingduan 2024 | CT VS MM | 5.438034 | 1.394967 | 9.481101 | 1 |
| Huang Xingduan 2024 | CT VS TF | 6.0100002 | 0.526585 | 11.49342 | 1 |
| Huang Xingduan 2024 | CT VS WDM | 3.4895411 | -0.4558699 | 7.434952 | 1 |
| Huang Xingduan 2024 | CT VS WN | 4.0655157 | 1.80997 | 6.321061 | 1 |
| Peng Jing 2022 | CT VS ACU | 3.1064022 | -3.803486 | 10.01629 | 1 |
| Peng Jing 2022 | CT VS EHM | -0.64129232 | -7.475399 | 6.192814 | 1 |
| Peng Jing 2022 | CT VS GIM | 3.9699998 | -1.79694 | 9.736939 | 1 |
| Peng Jing 2022 | CT VS HM | 1.5600004 | -3.975523 | 7.095523 | 1 |
| Peng Jing 2022 | CT VS IHM | 1.767106 | -5.173783 | 8.707994 | 1 |
| Peng Jing 2022 | CT VS MM | 5.4380328 | 1.395112 | 9.480953 | 1 |
| Peng Jing 2022 | CT VS TF | 6.0100002 | 0.526801 | 11.4932 | 1 |
| Peng Jing 2022 | CT VS WDM | 3.4893274 | -0.4555539 | 7.434209 | 1 |
| Peng Jing 2022 | CT VS WN | 4.0655183 | 1.81006 | 6.320976 | 1 |
| Liang Guoyu 2025 | CT VS ACU | 3.1063203 | -3.784173 | 9.996814 | 1 |
| Liang Guoyu 2025 | CT VS EHM | 0.06850023 | -6.759136 | 6.896136 | 1 |
| Liang Guoyu 2025 | CT VS GIM | 3.9699998 | -1.781431 | 9.721431 | 1 |
| Liang Guoyu 2025 | CT VS HM | 1.5600004 | -3.959363 | 7.079364 | 1 |
| Liang Guoyu 2025 | CT VS IHM | 1.76702 | -5.154556 | 8.688596 | 1 |
| Liang Guoyu 2025 | CT VS MM | 5.4379415 | 1.406071 | 9.469812 | 1 |
| Liang Guoyu 2025 | CT VS TF | 6.0100002 | 0.5431153 | 11.47688 | 1 |
| Liang Guoyu 2025 | CT VS WDM | 3.9472629 | -1.644347 | 9.538873 | 1 |
| Liang Guoyu 2025 | CT VS WN | 4.0657176 | 1.816881 | 6.314554 | 1 |

Leave-one-out sensitivity analysis of median SNCV in the subgroup analysis of patients with DPN (Table S144)

| **dropped_id** | **comparison** | **eff** | **lci** | **uci** | **connected** |
| --- | --- | --- | --- | --- | --- |
| Feng Yun 2022 | CT VS ACU | 0.1885283 | -2.732693 | 3.043636 | 1 |
| Feng Yun 2022 | CT VS EHM | 0.2668498 | -2.814816 | 3.081116 | 1 |
| Feng Yun 2022 | CT VS GIM | 5.04 | -1.97564 | 8.10436 | 1 |
| Feng Yun 2022 | CT VS HM | 2.2119932 | -0.1108354 | 4.313151 | 1 |
| Feng Yun 2022 | CT VS MM | 6.5394748 | 4.462649 | 8.616301 | 1 |
| Feng Yun 2022 | CT VS TF | 4.7662548 | 3.021337 | 6.511172 | 1 |
| Feng Yun 2022 | CT VS WDM | 2.3999739 | -0.5038701 | 5.303818 | 1 |
| Feng Yun 2022 | CT VS WN | 2.4714206 | 1.165264 | 3.777577 | 1 |
| Wang Dan 2021 | CT VS ACU | 0.9502281 | -2.562673 | 3.662217 | 1 |
| Wang Dan 2021 | CT VS EHM | 0.2756677 | -3.384631 | 3.833296 | 1 |
| Wang Dan 2021 | CT VS GIM | 5.0399999 | -0.0492692 | 10.12927 | 1 |
| Wang Dan 2021 | CT VS HM | 2.2106952 | -1.349448 | 5.770838 | 1 |
| Wang Dan 2021 | CT VS MM | 4.7000002 | 0.320497 | 9.720497 | 1 |
| Wang Dan 2021 | CT VS TF | 4.6008973 | 1.677146 | 7.524649 | 1 |
| Wang Dan 2021 | CT VS WDM | 2.3999306 | -2.591916 | 7.391778 | 1 |
| Wang Dan 2021 | CT VS WN | 3.7098272 | 1.675912 | 5.743742 | 1 |
| Zhang Xiujun 2020 | CT VS ACU | 0.9530212 | -2.653399 | 3.747357 | 1 |
| Zhang Xiujun 2020 | CT VS EHM | 0.2758171 | -3.488365 | 3.936731 | 1 |
| Zhang Xiujun 2020 | CT VS GIM | 5.04 | -0.1323761 | 10.21238 | 1 |
| Zhang Xiujun 2020 | CT VS HM | 2.2106726 | -1.408872 | 5.830217 | 1 |
| Zhang Xiujun 2020 | CT VS MM | 6.5298044 | 2.924972 | 10.13464 | 1 |
| Zhang Xiujun 2020 | CT VS TF | 5.4060293 | 1.809616 | 9.002442 | 1 |
| Zhang Xiujun 2020 | CT VS WDM | 2.3999284 | -2.676434 | 7.476291 | 1 |
| Zhang Xiujun 2020 | CT VS WN | 3.7070354 | 1.638466 | 5.775605 | 1 |
| Xu Xiaowei 2025 | CT VS ACU | 0.9580655 | -2.830179 | 3.914048 | 1 |
| Xu Xiaowei 2025 | CT VS EHM | 0.2760884 | -3.690552 | 4.138375 | 1 |
| Xu Xiaowei 2025 | CT VS GIM | 5.04 | -0.2945351 | 10.37453 | 1 |
| Xu Xiaowei 2025 | CT VS HM | 2.2599998 | -2.989588 | 7.509587 | 1 |
| Xu Xiaowei 2025 | CT VS MM | 6.5295088 | 2.808391 | 10.25063 | 1 |
| Xu Xiaowei 2025 | CT VS TF | 4.5930677 | 1.526812 | 7.659324 | 1 |
| Xu Xiaowei 2025 | CT VS WDM | 2.3999239 | -2.84119 | 7.641037 | 1 |
| Xu Xiaowei 2025 | CT VS WN | 3.7019936 | 1.56587 | 5.838118 | 1 |
| Zhang Suzhao 2024 | CT VS ACU | 0.8425033 | -2.784436 | 3.099429 | 1 |
| Zhang Suzhao 2024 | CT VS EHM | 0.2760744 | -3.679618 | 4.127469 | 1 |
| Zhang Suzhao 2024 | CT VS GIM | 5.04 | -0.2857599 | 10.36576 | 1 |
| Zhang Suzhao 2024 | CT VS HM | 2.2106336 | -1.51849 | 5.939757 | 1 |
| Zhang Suzhao 2024 | CT VS MM | 6.5295241 | 2.814696 | 10.24435 | 1 |
| Zhang Suzhao 2024 | CT VS TF | 4.5933285 | 1.532167 | 7.65449 | 1 |
| Zhang Suzhao 2024 | CT VS WDM | 2.3999241 | -2.832279 | 7.632127 | 1 |
| Zhang Suzhao 2024 | CT VS WN | 3.8175741 | 1.476274 | 6.158875 | 1 |
| Zhang Xiujun 2020 | CT VS ACU | 0.9573959 | -2.805675 | 3.890883 | 1 |
| Zhang Xiujun 2020 | CT VS EHM | 0.2760523 | -3.66252 | 4.110416 | 1 |
| Zhang Xiujun 2020 | CT VS GIM | 5.04 | -0.2720399 | 10.35204 | 1 |
| Zhang Xiujun 2020 | CT VS HM | 2.210637 | -1.508688 | 5.929962 | 1 |
| Zhang Xiujun 2020 | CT VS MM | 6.5295481 | 2.824556 | 10.23454 | 1 |
| Zhang Xiujun 2020 | CT VS TF | 4.8414874 | 1.124071 | 8.558905 | 1 |
| Zhang Xiujun 2020 | CT VS WDM | 2.3999245 | -2.818346 | 7.618195 | 1 |
| Zhang Xiujun 2020 | CT VS WN | 3.7026629 | 1.575903 | 5.829423 | 1 |
| Hu Guanghui 2024 | CT VS ACU | 0.2967216 | -2.703208 | 3.109765 | 1 |
| Hu Guanghui 2024 | CT VS EHM | 0.2751464 | -3.059132 | 3.508839 | 1 |
| Hu Guanghui 2024 | CT VS GIM | 5.04 | -0.2110662 | 9.868934 | 1 |
| Hu Guanghui 2024 | CT VS HM | 2.210774 | -1.163142 | 5.58469 | 1 |
| Hu Guanghui 2024 | CT VS MM | 6.5305361 | 3.172356 | 9.888717 | 1 |
| Hu Guanghui 2024 | CT VS TF | 4.6106248 | 1.838228 | 7.383022 | 1 |
| Hu Guanghui 2024 | CT VS WDM | 2.3999374 | -2.326776 | 7.126651 | 1 |
| Hu Guanghui 2024 | CT VS WN | 4.3634151 | 2.254143 | 6.472687 | 1 |
| Liu Xiangxiu 2012 | CT VS ACU | 0.9502281 | -2.562673 | 3.662216 | 1 |
| Liu Xiangxiu 2012 | CT VS EHM | 0.2756677 | -3.38463 | 3.833295 | 1 |
| Liu Xiangxiu 2012 | CT VS GIM | 5.0399999 | -0.049269 | 10.12927 | 1 |
| Liu Xiangxiu 2012 | CT VS HM | 2.2106952 | -1.349447 | 5.770838 | 1 |
| Liu Xiangxiu 2012 | CT VS MM | 8.3499994 | 3.343149 | 13.35685 | 1 |
| Liu Xiangxiu 2012 | CT VS TF | 4.6008973 | 1.677146 | 7.524649 | 1 |
| Liu Xiangxiu 2012 | CT VS WDM | 2.3999306 | -2.591916 | 7.391777 | 1 |
| Liu Xiangxiu 2012 | CT VS WN | 3.7098272 | 1.675912 | 5.743742 | 1 |
| Xu Lilii 2021 | CT VS ACU | 0.9502899 | -2.564633 | 3.664053 | 1 |
| Xu Lilii 2021 | CT VS EHM | 0.275671 | -3.386871 | 3.835529 | 1 |
| Xu Lilii 2021 | CT VS GIM | 5.04 | -0.0510631 | 10.13106 | 1 |
| Xu Lilii 2021 | CT VS HM | 2.2106947 | -1.350731 | 5.77212 | 1 |
| Xu Lilii 2021 | CT VS MM | 6.5299637 | 2.983481 | 10.07645 | 1 |
| Xu Lilii 2021 | CT VS TF | 3.4773923 | 0.1735176 | 7.128302 | 1 |
| Xu Lilii 2021 | CT VS WDM | 2.3999305 | -2.593741 | 7.393602 | 1 |
| Xu Lilii 2021 | CT VS WN | 3.7097654 | 1.675099 | 5.744432 | 1 |
| Du Minzhen 2019 | CT VS ACU | 0.8593178 | -2.799572 | 3.080936 | 1 |
| Du Minzhen 2019 | CT VS EHM | 0.2760748 | -3.679925 | 4.127775 | 1 |
| Du Minzhen 2019 | CT VS GIM | 5.04 | -0.2860065 | 10.36601 | 1 |
| Du Minzhen 2019 | CT VS HM | 2.2106336 | -1.518666 | 5.939933 | 1 |
| Du Minzhen 2019 | CT VS MM | 6.5295237 | 2.814519 | 10.24453 | 1 |
| Du Minzhen 2019 | CT VS TF | 4.5933211 | 1.532017 | 7.654626 | 1 |
| Du Minzhen 2019 | CT VS WDM | 2.3999241 | -2.83253 | 7.632378 | 1 |
| Du Minzhen 2019 | CT VS WN | 3.8007569 | 1.464283 | 6.13723 | 1 |
| Jin Zhu 2020 | CT VS ACU | 0.7817462 | -2.697668 | 3.134176 | 1 |
| Jin Zhu 2020 | CT VS EHM | 0.2760365 | -3.65032 | 4.098247 | 1 |
| Jin Zhu 2020 | CT VS GIM | 5.04 | -0.26225 | 10.34225 | 1 |
| Jin Zhu 2020 | CT VS HM | 2.2106394 | -1.501693 | 5.922972 | 1 |
| Jin Zhu 2020 | CT VS MM | 6.5295654 | 2.831591 | 10.22754 | 1 |
| Jin Zhu 2020 | CT VS TF | 4.5940338 | 1.546525 | 7.641542 | 1 |
| Jin Zhu 2020 | CT VS WDM | 2.3999248 | -2.808403 | 7.608253 | 1 |
| Jin Zhu 2020 | CT VS WN | 3.87834 | 1.549596 | 6.207084 | 1 |
| Li Jianshu 2025 | CT VS ACU | 0.7705441 | -2.644292 | 3.103204 | 1 |
| Li Jianshu 2025 | CT VS EHM | 0.2759882 | -3.613651 | 4.061675 | 1 |
| Li Jianshu 2025 | CT VS GIM | 5.04 | -0.2328321 | 10.31283 | 1 |
| Li Jianshu 2025 | CT VS HM | 2.2106467 | -1.480672 | 5.901966 | 1 |
| Li Jianshu 2025 | CT VS MM | 6.5296179 | 2.852736 | 10.2065 | 1 |
| Li Jianshu 2025 | CT VS TF | 4.5949303 | 1.564514 | 7.625347 | 1 |
| Li Jianshu 2025 | CT VS WDM | 2.3999256 | -2.778522 | 7.578373 | 1 |
| Li Jianshu 2025 | CT VS WN | 3.889543 | 1.601136 | 6.177949 | 1 |
| Feng Xueqiang 2024 | ACU VS CT | 0.2756752 | -3.389757 | 3.838407 | 1 |
| Feng Xueqiang 2024 | ACU VS EHM | 5.04 | -0.0533748 | 10.13337 | 1 |
| Feng Xueqiang 2024 | ACU VS GIM | 2.2106941 | 1.352384 | 5.773772 | 1 |
| Feng Xueqiang 2024 | ACU VS HM | 6.5299591 | -2.981817 | 10.0781 | 1 |
| Feng Xueqiang 2024 | ACU VS MM | 4.6007565 | 1.674704 | 7.526809 | 1 |
| Feng Xueqiang 2024 | ACU VS TF | 2.3999305 | 0.596093 | 7.395954 | 1 |
| Feng Xueqiang 2024 | ACU VS WDM | 3.7097023 | -1.674155 | 5.74525 | 1 |
| Liu Maomao 2023 | CT VS ACU | 0.9580656 | -2.83018 | 3.914049 | 1 |
| Liu Maomao 2023 | CT VS EHM | 0.2760884 | -3.690554 | 4.138376 | 1 |
| Liu Maomao 2023 | CT VS GIM | 5.04 | -0.2945363 | 10.37454 | 1 |
| Liu Maomao 2023 | CT VS HM | 2.1600001 | -3.156326 | 7.476326 | 1 |
| Liu Maomao 2023 | CT VS MM | 6.5295088 | 2.80839 | 10.25063 | 1 |
| Liu Maomao 2023 | CT VS TF | 4.5930677 | 1.526811 | 7.659324 | 1 |
| Liu Maomao 2023 | CT VS WDM | 2.3999239 | -2.841191 | 7.641039 | 1 |
| Liu Maomao 2023 | CT VS WN | 3.7019935 | 1.565869 | 5.838118 | 1 |
| Huang Xingduan 2024 | CT VS ACU | 0.9579408 | -2.825589 | 3.909708 | 1 |
| Huang Xingduan 2024 | CT VS EHM | 0.9201615 | -3.300744 | 5.460421 | 1 |
| Huang Xingduan 2024 | CT VS GIM | 5.04 | -0.2903215 | 10.37032 | 1 |
| Huang Xingduan 2024 | CT VS HM | 2.2106325 | -1.521749 | 5.943014 | 1 |
| Huang Xingduan 2024 | CT VS MM | 6.5295161 | 2.811419 | 10.24761 | 1 |
| Huang Xingduan 2024 | CT VS TF | 4.5931928 | 1.529384 | 7.657001 | 1 |
| Huang Xingduan 2024 | CT VS WDM | 2.399892 | -2.838917 | 7.638701 | 1 |
| Huang Xingduan 2024 | CT VS WN | 3.7021183 | 1.567748 | 5.836488 | 1 |
| Peng Jing 2022 | CT VS ACU | 0.957936 | -2.825413 | 3.909541 | 1 |
| Peng Jing 2022 | CT VS EHM | 0.6398637 | -4.06009 | 4.78036 | 1 |
| Peng Jing 2022 | CT VS GIM | 5.04 | -0.2901597 | 10.37016 | 1 |
| Peng Jing 2022 | CT VS HM | 2.2106326 | -1.521633 | 5.942898 | 1 |
| Peng Jing 2022 | CT VS MM | 6.5295164 | 2.811535 | 10.2475 | 1 |
| Peng Jing 2022 | CT VS TF | 4.5931976 | 1.529483 | 7.656912 | 1 |
| Peng Jing 2022 | CT VS WDM | 2.4000903 | -2.837756 | 7.637937 | 1 |
| Peng Jing 2022 | CT VS WN | 3.7021231 | 1.567821 | 5.836426 | 1 |
| Liang Guoyu 2025 | —— | —— | —— | —— | disconnected |
| Hu Guanghui 2023 | CT VS ACU | 0.9503672 | -2.567037 | 3.666303 | 1 |
| Hu Guanghui 2023 | CT VS EHM | 0.2756751 | -3.389671 | 3.838321 | 1 |
| Hu Guanghui 2023 | CT VS GIM | 2.2106941 | -1.352335 | 5.773723 | 1 |
| Hu Guanghui 2023 | CT VS HM | 6.5299592 | -2.981866 | 10.07805 | 1 |
| Hu Guanghui 2023 | CT VS MM | 4.6007588 | 1.674747 | 7.526771 | 1 |
| Hu Guanghui 2023 | CT VS TF | 2.3999305 | 0.596023 | 7.395884 | 1 |
| Hu Guanghui 2023 | CT VS WDM | 3.7096882 | 1.674213 | 5.745163 | 1 |

Leave-one-out sensitivity analysis of TCSS in the subgroup analysis of patients with DPN (Table S145)

| **dropped_id** | **comparison** | **eff** | **lci** | **uci** | **connected** |
| --- | --- | --- | --- | --- | --- |
| Ma Guoqing 2018 | CT VS ACU | -0.3584039 | -2.401578 | 1.68477 | 1 |
| Ma Guoqing 2018 | CT VS HM | -1.7133618 | -3.417832 | -0.0088917 | 1 |
| Ma Guoqing 2018 | CT VS MM | -2.3686079 | -3.861581 | -0.8756344 | 1 |
| Ma Guoqing 2018 | CT VS TF | -1.6279899 | -3.286708 | -0.0307284 | 1 |
| Ma Guoqing 2018 | CT VS WN | -1.5096018 | -2.637278 | -0.3819253 | 1 |
| Wang Jiawei 2023 | CT VS ACU | -0.56396891 | -2.042369 | 0.914431 | 1 |
| Wang Jiawei 2023 | CT VS HM | -1.6984258 | -3.101236 | -0.2956159 | 1 |
| Wang Jiawei 2023 | CT VS MM | -3.2864758 | -4.854615 | -1.718337 | 1 |
| Wang Jiawei 2023 | CT VS TF | -1.5864387 | -2.932091 | -0.2407864 | 1 |
| Wang Jiawei 2023 | CT VS WN | -1.6256439 | -2.544487 | -0.7068006 | 1 |
| Fang Xiujuan 2020 | CT VS ACU | -0.22266644 | -1.90943 | 1.464097 | 1 |
| Fang Xiujuan 2020 | CT VS HM | -1.1799998 | -3.469663 | -0.109664 | 1 |
| Fang Xiujuan 2020 | CT VS MM | -2.3310578 | -3.756832 | -0.905284 | 1 |
| Fang Xiujuan 2020 | CT VS TF | -1.6239977 | -3.24306 | -0.0049353 | 1 |
| Fang Xiujuan 2020 | CT VS WN | -1.5277084 | -2.617703 | -0.4377139 | 1 |
| Feng Yun 2022 | CT VS ACU | -0.09033479 | -1.852041 | 1.671372 | 1 |
| Feng Yun 2022 | CT VS HM | -1.7124007 | -3.391423 | -0.0333789 | 1 |
| Feng Yun 2022 | CT VS MM | -2.2937517 | -3.737109 | -0.8503942 | 1 |
| Feng Yun 2022 | CT VS TF | -1.6253735 | -3.257837 | 0.0070895 | 1 |
| Feng Yun 2022 | CT VS WN | -1.3531314 | -2.60959 | -0.0966729 | 1 |
| Zhang Xiujun 2020 | CT VS ACU | -0.22059042 | -1.87177 | 1.430589 | 1 |
| Zhang Xiujun 2020 | CT VS HM | -1.7104226 | -3.340418 | -0.0804272 | 1 |
| Zhang Xiujun 2020 | CT VS MM | -2.3311305 | -3.723943 | -0.9383179 | 1 |
| Zhang Xiujun 2020 | CT VS TF | -0.97000003 | -3.078453 | -0.138453 | 1 |
| Zhang Xiujun 2020 | CT VS WN | -1.5261729 | -2.591647 | -0.4606989 | 1 |
| Zhang Suzhao 2024 | CT VS ACU | -0.1077171 | -1.857968 | 1.642534 | 1 |
| Zhang Suzhao 2024 | CT VS HM | -1.7123622 | -3.390382 | -0.034342 | 1 |
| Zhang Suzhao 2024 | CT VS MM | -2.2986463 | -3.740183 | -0.8571093 | 1 |
| Zhang Suzhao 2024 | CT VS TF | -1.6252685 | -3.256663 | 0.0061254 | 1 |
| Zhang Suzhao 2024 | CT VS WN | -1.3760426 | -2.606919 | -0.1451664 | 1 |
| Hu Guanghui 2024 | CT VS ACU | -0.34315553 | -2.115697 | 1.429386 | 1 |
| Hu Guanghui 2024 | CT VS HM | -1.7128972 | -3.404919 | -0.0208757 | 1 |
| Hu Guanghui 2024 | CT VS MM | -2.3644471 | -3.819544 | -0.9093501 | 1 |
| Hu Guanghui 2024 | CT VS TF | -1.626726 | -3.272579 | 0.0191269 | 1 |
| Hu Guanghui 2024 | CT VS WN | -1.6855101 | -2.946629 | -0.4243916 | 1 |
| Li Shuomiao 2024 | CT VS ACU | -0.22059042 | -1.87177 | 1.430589 | 1 |
| Li Shuomiao 2024 | CT VS HM | -1.7104226 | -3.340418 | -0.0804272 | 1 |
| Li Shuomiao 2024 | CT VS MM | -2.3311305 | -3.723943 | -0.9383179 | 1 |
| Li Shuomiao 2024 | CT VS TF | -2.4499998 | -4.832689 | -0.0673107 | 1 |
| Li Shuomiao 2024 | CT VS WN | -1.5261729 | -2.591647 | -0.4606989 | 1 |
| Luo Xingzi 2020 | CT VS ACU | -0.20621675 | -1.820873 | 2.233307 | 1 |
| Luo Xingzi 2020 | CT VS HM | -1.7115065 | -3.367822 | -0.0551905 | 1 |
| Luo Xingzi 2020 | CT VS MM | -2.5897982 | -4.165417 | -1.01418 | 1 |
| Luo Xingzi 2020 | CT VS TF | -1.6229324 | -3.231838 | -0.0140268 | 1 |
| Luo Xingzi 2020 | CT VS WN | -1.3914901 | -2.533113 | -0.2498668 | 1 |
| Feng Xueqiang 2024 | CT VS ACU | -0.41879989 | -2.421793 | 1.584194 | 1 |
| Feng Xueqiang 2024 | CT VS HM | -1.7130178 | -3.408244 | -0.0177915 | 1 |
| Feng Xueqiang 2024 | CT VS MM | -2.3856775 | -3.866797 | -0.9045576 | 1 |
| Feng Xueqiang 2024 | CT VS TF | -1.6270542 | -3.276217 | 0.0221088 | 1 |
| Feng Xueqiang 2024 | CT VS WN | -1.5002469 | -2.620297 | -0.3801968 | 1 |
| Wang Lina 2022 | CT VS ACU | -0.47294881 | -2.141105 | 1.195207 | 1 |
| Wang Lina 2022 | CT VS HM | -1.7086282 | -3.297639 | -0.1196174 | 1 |
| Wang Lina 2022 | CT VS MM | -2.4011481 | -3.761754 | -1.040542 | 1 |
| Wang Lina 2022 | CT VS TF | -1.615029 | -3.154205 | -0.0758533 | 1 |
| Wang Lina 2022 | CT VS WN | -1.8592874 | -3.043481 | -0.6750938 | 1 |
| Wang Jiawei 2023 | CT VS ACU | -0.26941902 | -0.9015158 | 1.440354 | 1 |
| Wang Jiawei 2023 | CT VS HM | -1.6714838 | -2.783794 | -0.5591731 | 1 |
| Wang Jiawei 2023 | CT VS MM | -1.085718 | -2.254485 | 0.0830491 | 1 |
| Wang Jiawei 2023 | CT VS TF | -1.506333 | -2.545087 | -0.4675788 | 1 |
| Wang Jiawei 2023 | CT VS WN | -1.3578882 | -2.070364 | -0.6454121 | 1 |
| Hu Shuhan 2026 | CT VS ACU | -0.22266643 | -1.90943 | 1.464097 | 1 |
| Hu Shuhan 2026 | CT VS HM | -2.3099999 | -4.737987 | 0.1179866 | 1 |
| Hu Shuhan 2026 | CT VS MM | -2.3310578 | -3.756832 | -0.905284 | 1 |
| Hu Shuhan 2026 | CT VS TF | -1.6239977 | -3.24306 | -0.0049353 | 1 |
| Hu Shuhan 2026 | CT VS WN | -1.5277084 | -2.617703 | -0.4377139 | 1 |

Leave-one-out sensitivity analysis of markedly effective rate in the subgroup analysis of patients with DPN (Table S146)

| **dropped_id** | **comparison** | **log_eff** | **log_lci** | **log_uci** | **OR** | **OR_lci** | **OR_uci** | **connected** |
| --- | --- | --- | --- | --- | --- | --- | --- | --- |
| Wang Dan 2021 | CT VS ACU | 0.03793654 | -0.3310883 | 0.4069614 | 1.0386653 | 0.7181417 | 1.502246 | 1 |
| Wang Dan 2021 | CT VS EHM | 0.00087108 | -0.3298427 | 0.3315848 | 1.0008715 | 0.7190369 | 1.393174 | 1 |
| Wang Dan 2021 | CT VS GIM | 0.10536052 | -0.1161956 | 0.3269166 | 1.1111111 | 0.890301 | 1.386686 | 1 |
| Wang Dan 2021 | CT VS HM | 0.55037778 | -0.0256921 | 1.126448 | 1.7339079 | 0.9746351 | 3.084679 | 1 |
| Wang Dan 2021 | CT VS MM | 0.87719143 | 0.3579932 | 1.39639 | 2.404138 | 1.430456 | 4.040586 | 1 |
| Wang Dan 2021 | CT VS TF | 0.29371642 | 0.0721596 | 0.5152733 | 1.3414035 | 1.074827 | 1.674096 | 1 |
| Wang Dan 2021 | CT VS WDM | 0.31229396 | 0.0358517 | 0.5887362 | 1.3665563 | 1.036502 | 1.80171 | 1 |
| Wang Dan 2021 | CT VS WN | 0.3171903 | 0.155647 | 0.4787336 | 1.3732639 | 1.168414 | 1.614029 | 1 |
| Ji Hua 2025 | CT VS ACU | -0.03905825 | -0.3885302 | 0.3104137 | 0.96169469 | 0.6780527 | 1.363989 | 1 |
| Ji Hua 2025 | CT VS EHM | 0.07940729 | -0.3115926 | 0.4704072 | 1.0826452 | 0.7322798 | 1.600646 | 1 |
| Ji Hua 2025 | CT VS GIM | 0.10536052 | -0.1161956 | 0.3269166 | 1.1111111 | 0.890301 | 1.386686 | 1 |
| Ji Hua 2025 | CT VS HM | 0.55037776 | -0.0256921 | 1.126448 | 1.7339079 | 0.9746351 | 3.084679 | 1 |
| Ji Hua 2025 | CT VS MM | 0.58498873 | 0.3257068 | 0.8442706 | 1.7949708 | 1.385009 | 2.326281 | 1 |
| Ji Hua 2025 | CT VS TF | 0.29371644 | 0.0721596 | 0.5152733 | 1.3414035 | 1.074827 | 1.674096 | 1 |
| Ji Hua 2025 | CT VS WDM | 0.39083091 | 0.0445211 | 0.7371407 | 1.4782085 | 1.045527 | 2.089951 | 1 |
| Ji Hua 2025 | CT VS WN | 0.30427571 | 0.1439604 | 0.464591 | 1.3556428 | 1.154838 | 1.591363 | 1 |
| Zhang Xiujun 2020 | CT VS ACU | -0.04010514 | -0.3980155 | 0.3178052 | 0.96068842 | 0.6716516 | 1.374109 | 1 |
| Zhang Xiujun 2020 | CT VS EHM | 0.00048987 | -0.338116 | 0.3390957 | 1.00049 | 0.7131126 | 1.403678 | 1 |
| Zhang Xiujun 2020 | CT VS GIM | 0.10536052 | -0.124881 | 0.335602 | 1.1111111 | 0.8826019 | 1.398782 | 1 |
| Zhang Xiujun 2020 | CT VS HM | 0.5511134 | -0.0281662 | 1.130393 | 1.7351839 | 0.9722268 | 3.096873 | 1 |
| Zhang Xiujun 2020 | CT VS MM | 0.5874338 | 0.2943173 | 0.8805503 | 1.799365 | 1.34221 | 2.412227 | 1 |
| Zhang Xiujun 2020 | CT VS TF | 0.22314355 | 0.1796876 | 0.6259747 | 1.25 | 1.0355312 | 1.870068 | 1 |
| Zhang Xiujun 2020 | CT VS WDM | 0.31160489 | 0.0291176 | 0.5940921 | 1.365615 | 1.029546 | 1.811386 | 1 |
| Zhang Xiujun 2020 | CT VS WN | 0.30331297 | 0.1331296 | 0.4734963 | 1.3543383 | 1.142398 | 1.605598 | 1 |
| Zhang Suzhao 2024 | CT VS ACU | -0.0605011 | -0.4110493 | 0.2900471 | 0.94129273 | 0.6629542 | 1.336491 | 1 |
| Zhang Suzhao 2024 | CT VS EHM | 0.00087108 | -0.3298427 | 0.3315848 | 1.0008715 | 0.7190369 | 1.393174 | 1 |
| Zhang Suzhao 2024 | CT VS GIM | 0.10536052 | -0.1161956 | 0.3269166 | 1.1111111 | 0.890301 | 1.386686 | 1 |
| Zhang Suzhao 2024 | CT VS HM | 0.55037778 | -0.0256921 | 1.126448 | 1.7339079 | 0.9746351 | 3.084679 | 1 |
| Zhang Suzhao 2024 | CT VS MM | 0.58187867 | 0.3225662 | 0.8411911 | 1.789397 | 1.380666 | 2.319128 | 1 |
| Zhang Suzhao 2024 | CT VS TF | 0.29371642 | 0.0721596 | 0.5152733 | 1.3414035 | 1.074827 | 1.674096 | 1 |
| Zhang Suzhao 2024 | CT VS WDM | 0.31229396 | 0.0358517 | 0.5887362 | 1.3665563 | 1.036502 | 1.80171 | 1 |
| Zhang Suzhao 2024 | CT VS WN | 0.27737352 | 0.1134013 | 0.4413458 | 1.3196592 | 1.120081 | 1.554798 | 1 |
| Hu Guanghui 2024 | CT VS ACU | -0.04307896 | -0.395444 | 0.309286 | 0.95783575 | 0.673381 | 1.362452 | 1 |
| Hu Guanghui 2024 | CT VS EHM | 0.00087108 | -0.3298427 | 0.3315848 | 1.0008715 | 0.7190369 | 1.393174 | 1 |
| Hu Guanghui 2024 | CT VS GIM | 0.10536052 | -0.1161956 | 0.3269166 | 1.1111111 | 0.890301 | 1.386686 | 1 |
| Hu Guanghui 2024 | CT VS HM | 0.55037778 | -0.0256921 | 1.126448 | 1.7339079 | 0.9746351 | 3.084679 | 1 |
| Hu Guanghui 2024 | CT VS MM | 0.58440563 | 0.3250414 | 0.8437699 | 1.7939244 | 1.384088 | 2.325116 | 1 |
| Hu Guanghui 2024 | CT VS TF | 0.29371642 | 0.0721596 | 0.5152733 | 1.3414035 | 1.074827 | 1.674096 | 1 |
| Hu Guanghui 2024 | CT VS WDM | 0.31229396 | 0.0358517 | 0.5887362 | 1.3665563 | 1.036502 | 1.80171 | 1 |
| Hu Guanghui 2024 | CT VS WN | 0.29923134 | 0.1292402 | 0.4692225 | 1.3488216 | 1.137963 | 1.598751 | 1 |
| Liu Xiangxiu 2012 | CT VS ACU | -0.0437285 | -0.3934993 | 0.3060423 | 0.9572138 | 0.6746918 | 1.35804 | 1 |
| Liu Xiangxiu 2012 | CT VS EHM | 0.00087108 | -0.3298427 | 0.3315848 | 1.0008715 | 0.7190369 | 1.393174 | 1 |
| Liu Xiangxiu 2012 | CT VS GIM | 0.10536052 | -0.1161956 | 0.3269166 | 1.1111111 | 0.890301 | 1.386686 | 1 |
| Liu Xiangxiu 2012 | CT VS HM | 0.55037777 | -0.0256921 | 1.126448 | 1.7339079 | 0.9746351 | 3.084679 | 1 |
| Liu Xiangxiu 2012 | CT VS MM | 0.56726484 | 0.3022417 | 0.8322879 | 1.7634372 | 1.352888 | 2.298572 | 1 |
| Liu Xiangxiu 2012 | CT VS TF | 0.29371642 | 0.0721596 | 0.5152733 | 1.3414035 | 1.074827 | 1.674096 | 1 |
| Liu Xiangxiu 2012 | CT VS WDM | 0.31229396 | 0.0358517 | 0.5887362 | 1.3665564 | 1.036502 | 1.80171 | 1 |
| Liu Xiangxiu 2012 | CT VS WN | 0.30349234 | 0.1431587 | 0.463826 | 1.3545812 | 1.153913 | 1.590146 | 1 |
| Wu Min 2017 | CT VS ACU | -0.0390582 | -0.3885301 | 0.3104137 | 0.96169473 | 0.6780528 | 1.363989 | 1 |
| Wu Min 2017 | CT VS EHM | 0.0008711 | -0.3298426 | 0.3315848 | 1.0008715 | 0.7190369 | 1.393174 | 1 |
| Wu Min 2017 | CT VS GIM | 0.10536052 | -0.1161956 | 0.3269166 | 1.1111111 | 0.890301 | 1.386686 | 1 |
| Wu Min 2017 | CT VS HM | 0.28768207 | -0.4129399 | 0.988304 | 1.3333333 | 0.6617021 | 2.686674 | 1 |
| Wu Min 2017 | CT VS MM | 0.58498863 | 0.3257067 | 0.8442705 | 1.7949706 | 1.385009 | 2.32628 | 1 |
| Wu Min 2017 | CT VS TF | 0.29371647 | 0.0721596 | 0.5152733 | 1.3414035 | 1.074827 | 1.674096 | 1 |
| Wu Min 2017 | CT VS WDM | 0.31229401 | 0.0358518 | 0.5887362 | 1.3665564 | 1.036502 | 1.80171 | 1 |
| Wu Min 2017 | CT VS WN | 0.30427576 | 0.1439605 | 0.4645911 | 1.3556428 | 1.154838 | 1.591363 | 1 |
| Jin Zhu 2020 | CT VS ACU | -0.033145 | -0.3840352 | 0.3177452 | 0.96739828 | 0.6811075 | 1.374026 | 1 |
| Jin Zhu 2020 | CT VS EHM | 0.00087108 | -0.3298427 | 0.3315848 | 1.0008715 | 0.7190369 | 1.393174 | 1 |
| Jin Zhu 2020 | CT VS GIM | 0.10536052 | -0.1161956 | 0.3269166 | 1.1111111 | 0.890301 | 1.386686 | 1 |
| Jin Zhu 2020 | CT VS HM | 0.55037778 | -0.0256921 | 1.126448 | 1.7339079 | 0.9746351 | 3.084679 | 1 |
| Jin Zhu 2020 | CT VS MM | 0.58584648 | 0.3265243 | 0.8451687 | 1.7965111 | 1.386142 | 2.328371 | 1 |
| Jin Zhu 2020 | CT VS TF | 0.29371642 | 0.0721596 | 0.5152733 | 1.3414035 | 1.074827 | 1.674096 | 1 |
| Jin Zhu 2020 | CT VS WDM | 0.31229396 | 0.0358517 | 0.5887362 | 1.3665563 | 1.036502 | 1.80171 | 1 |
| Jin Zhu 2020 | CT VS WN | 0.3116945 | 0.146575 | 0.476814 | 1.3657374 | 1.157862 | 1.610934 | 1 |
| Luo Xingzi 2020 | CT VS ACU | 0.02780593 | -0.3715543 | 0.4271662 | 1.0281961 | 0.6896615 | 1.532907 | 1 |
| Luo Xingzi 2020 | CT VS EHM | 0.00087108 | -0.3298427 | 0.3315848 | 1.0008715 | 0.7190369 | 1.393174 | 1 |
| Luo Xingzi 2020 | CT VS GIM | 0.10536052 | -0.1161956 | 0.3269166 | 1.1111111 | 0.890301 | 1.386686 | 1 |
| Luo Xingzi 2020 | CT VS HM | 0.55037778 | -0.0256921 | 1.126448 | 1.7339079 | 0.9746351 | 3.084679 | 1 |
| Luo Xingzi 2020 | CT VS MM | 0.55328673 | 0.2782816 | 0.8282918 | 1.7389591 | 1.320858 | 2.289405 | 1 |
| Luo Xingzi 2020 | CT VS TF | 0.29371642 | 0.0721596 | 0.5152733 | 1.3414035 | 1.074827 | 1.674096 | 1 |
| Luo Xingzi 2020 | CT VS WDM | 0.31229396 | 0.0358517 | 0.5887362 | 1.3665563 | 1.036502 | 1.80171 | 1 |
| Luo Xingzi 2020 | CT VS WN | 0.31549106 | 0.1519305 | 0.4790516 | 1.3709324 | 1.164079 | 1.614542 | 1 |
| Li Jianshu 2025 | CT VS ACU | -0.06825509 | -0.4188729 | 0.2823628 | 0.93402219 | 0.6577878 | 1.32626 | 1 |
| Li Jianshu 2025 | CT VS EHM | 0.00087108 | -0.3298427 | 0.3315848 | 1.0008715 | 0.7190369 | 1.393174 | 1 |
| Li Jianshu 2025 | CT VS GIM | 0.10536052 | -0.1161956 | 0.3269166 | 1.1111111 | 0.890301 | 1.386686 | 1 |
| Li Jianshu 2025 | CT VS HM | 0.55037777 | -0.0256921 | 1.126448 | 1.7339079 | 0.9746351 | 3.084679 | 1 |
| Li Jianshu 2025 | CT VS MM | 0.580754 | 0.3214396 | 0.8400685 | 1.7873856 | 1.379112 | 2.316525 | 1 |
| Li Jianshu 2025 | CT VS TF | 0.29371642 | 0.0721596 | 0.5152733 | 1.3414035 | 1.074827 | 1.674096 | 1 |
| Li Jianshu 2025 | CT VS WDM | 0.31229396 | 0.0358517 | 0.5887362 | 1.3665563 | 1.036502 | 1.80171 | 1 |
| Li Jianshu 2025 | CT VS WN | 0.26764537 | 0.103439 | 0.4318518 | 1.3068836 | 1.108978 | 1.540107 | 1 |
| Wang Yue 2022 | CT VS ACU | -0.02287202 | -0.3747208 | 0.3289768 | 0.97738756 | 0.6874812 | 1.389546 | 1 |
| Wang Yue 2022 | CT VS EHM | 0.00087108 | -0.3298427 | 0.3315848 | 1.0008715 | 0.7190369 | 1.393174 | 1 |
| Wang Yue 2022 | CT VS GIM | 0.10536052 | -0.1161956 | 0.3269166 | 1.1111111 | 0.890301 | 1.386686 | 1 |
| Wang Yue 2022 | CT VS HM | 0.55037778 | -0.0256921 | 1.126448 | 1.7339079 | 0.9746351 | 3.084679 | 1 |
| Wang Yue 2022 | CT VS MM | 0.58733651 | 0.327987 | 0.846686 | 1.7991899 | 1.388171 | 2.331906 | 1 |
| Wang Yue 2022 | CT VS TF | 0.29371642 | 0.0721596 | 0.5152733 | 1.3414035 | 1.074827 | 1.674096 | 1 |
| Wang Yue 2022 | CT VS WDM | 0.31229396 | 0.0358517 | 0.5887362 | 1.3665563 | 1.036502 | 1.80171 | 1 |
| Wang Yue 2022 | CT VS WN | 0.32458297 | 0.1562832 | 0.4928827 | 1.3834536 | 1.169157 | 1.637029 | 1 |
| Feng Xueqiang 2024 | CT VS ACU | -0.25765056 | -0.9797307 | 0.4644296 | 0.77286525 | 0.3754122 | 1.591106 | 1 |
| Feng Xueqiang 2024 | CT VS EHM | 0.00087108 | -0.3298427 | 0.3315848 | 1.0008715 | 0.7190369 | 1.393174 | 1 |
| Feng Xueqiang 2024 | CT VS GIM | 0.10536052 | -0.1161956 | 0.3269166 | 1.1111111 | 0.890301 | 1.386686 | 1 |
| Feng Xueqiang 2024 | CT VS HM | 0.55037778 | -0.0256921 | 1.126448 | 1.7339079 | 0.9746351 | 3.084679 | 1 |
| Feng Xueqiang 2024 | CT VS MM | 0.55328349 | 0.2782803 | 0.8282866 | 1.7389535 | 1.320856 | 2.289393 | 1 |
| Feng Xueqiang 2024 | CT VS TF | 0.29371642 | 0.0721596 | 0.5152733 | 1.3414035 | 1.074827 | 1.674096 | 1 |
| Feng Xueqiang 2024 | CT VS WDM | 0.31229396 | 0.0358517 | 0.5887362 | 1.3665563 | 1.036502 | 1.80171 | 1 |
| Feng Xueqiang 2024 | CT VS WN | 0.31549294 | 0.1519316 | 0.4790543 | 1.3709349 | 1.164081 | 1.614547 | 1 |
| Du Xinze 2023 | CT VS ACU | -0.08532701 | -0.4564618 | 0.2858078 | 0.91821197 | 0.6335212 | 1.330837 | 1 |
| Du Xinze 2023 | CT VS EHM | 0.00087108 | -0.3298427 | 0.3315848 | 1.0008715 | 0.7190369 | 1.393174 | 1 |
| Du Xinze 2023 | CT VS GIM | 0.10536052 | -0.1161956 | 0.3269166 | 1.1111111 | 0.890301 | 1.386686 | 1 |
| Du Xinze 2023 | CT VS HM | 0.55037778 | -0.0256921 | 1.126448 | 1.7339079 | 0.9746351 | 3.084679 | 1 |
| Du Xinze 2023 | CT VS MM | 0.57827785 | 0.3183634 | 0.8381923 | 1.7829652 | 1.374876 | 2.312183 | 1 |
| Du Xinze 2023 | CT VS TF | 0.29371642 | 0.0721596 | 0.5152733 | 1.3414035 | 1.074827 | 1.674096 | 1 |
| Du Xinze 2023 | CT VS WDM | 0.31229396 | 0.0358517 | 0.5887362 | 1.3665563 | 1.036502 | 1.80171 | 1 |
| Du Xinze 2023 | CT VS WN | 0.24622694 | 0.0220124 | 0.4704415 | 1.2791898 | 1.022256 | 1.600701 | 1 |
| Wang Lina 2022 | CT VS ACU | 0.01404248 | -0.3386134 | 0.3666983 | 1.0141415 | 0.712758 | 1.442963 | 1 |
| Wang Lina 2022 | CT VS EHM | 0.00087108 | -0.3298426 | 0.3315848 | 1.0008715 | 0.7190369 | 1.393174 | 1 |
| Wang Lina 2022 | CT VS GIM | 0.10536052 | -0.1161956 | 0.3269166 | 1.1111111 | 0.890301 | 1.386686 | 1 |
| Wang Lina 2022 | CT VS HM | 0.55037777 | -0.0256921 | 1.126448 | 1.7339079 | 0.9746351 | 3.084679 | 1 |
| Wang Lina 2022 | CT VS MM | 0.59269068 | 0.3333181 | 0.8520632 | 1.8088489 | 1.395591 | 2.344479 | 1 |
| Wang Lina 2022 | CT VS TF | 0.29371643 | 0.0721596 | 0.5152733 | 1.3414035 | 1.074827 | 1.674096 | 1 |
| Wang Lina 2022 | CT VS WDM | 0.31229397 | 0.0358517 | 0.5887362 | 1.3665564 | 1.036502 | 1.80171 | 1 |
| Wang Lina 2022 | CT VS WN | 0.37089591 | 0.1999581 | 0.5418337 | 1.4490322 | 1.221352 | 1.719156 | 1 |
| Li Xiaoting 2014 | CT VS ACU | -0.04713535 | -0.3973113 | 0.3030406 | 0.95395827 | 0.6721247 | 1.353969 | 1 |
| Li Xiaoting 2014 | CT VS EHM | 0.00087108 | -0.3298427 | 0.3315848 | 1.0008715 | 0.7190369 | 1.393174 | 1 |
| Li Xiaoting 2014 | CT VS GIM | 0.10536052 | -0.1161956 | 0.3269166 | 1.1111111 | 0.890301 | 1.386686 | 1 |
| Li Xiaoting 2014 | CT VS HM | 0.55037778 | -0.0256921 | 1.126448 | 1.7339079 | 0.9746351 | 3.084679 | 1 |
| Li Xiaoting 2014 | CT VS MM | 0.55433559 | 0.2817162 | 0.826955 | 1.740784 | 1.325402 | 2.286346 | 1 |
| Li Xiaoting 2014 | CT VS TF | 0.29371642 | 0.0721596 | 0.5152733 | 1.3414035 | 1.074827 | 1.674096 | 1 |
| Li Xiaoting 2014 | CT VS WDM | 0.31229396 | 0.0358517 | 0.5887362 | 1.3665563 | 1.036502 | 1.80171 | 1 |
| Li Xiaoting 2014 | CT VS WN | 0.30292089 | 0.1425624 | 0.4632794 | 1.3538074 | 1.153225 | 1.589277 | 1 |
| Luo Xiaohong 2022 | CT VS ACU | -0.04010679 | -0.3980215 | 0.3178079 | 0.96068684 | 0.6716476 | 1.374112 | 1 |
| Luo Xiaohong 2022 | CT VS EHM | 0.00048937 | -0.3381307 | 0.3391094 | 1.0004895 | 0.7131021 | 1.403697 | 1 |
| Luo Xiaohong 2022 | CT VS GIM | 0.10536051 | -0.1248987 | 0.3356197 | 1.1111111 | 0.8825863 | 1.398807 | 1 |
| Luo Xiaohong 2022 | CT VS HM | 0.55111447 | -0.028168 | 1.130397 | 1.7351858 | 0.9722251 | 3.096885 | 1 |
| Luo Xiaohong 2022 | CT VS MM | 0.5874374 | 0.2943384 | 0.8805364 | 1.7993714 | 1.342238 | 2.412193 | 1 |
| Luo Xiaohong 2022 | CT VS TF | 0.3254224 | 0.051428 | 0.5994168 | 1.3846154 | 1.052773 | 1.821056 | 1 |
| Luo Xiaohong 2022 | CT VS WDM | 0.31160386 | 0.0291115 | 0.5940962 | 1.3656136 | 1.029539 | 1.811393 | 1 |
| Luo Xiaohong 2022 | CT VS WN | 0.30331143 | 0.1331313 | 0.4734916 | 1.3543362 | 1.1424 | 1.60559 | 1 |
| Huang Xingduan 2024 | CT VS ACU | -0.04157805 | -0.4061038 | 0.3229477 | 0.95927446 | 0.666241 | 1.381193 | 1 |
| Huang Xingduan 2024 | CT VS EHM | -0.00586434 | -0.3719083 | 0.3601797 | 0.99415282 | 0.6894174 | 1.433587 | 1 |
| Huang Xingduan 2024 | CT VS GIM | 0.10536052 | -0.1393816 | 0.3501026 | 1.1111111 | 0.869896 | 1.419213 | 1 |
| Huang Xingduan 2024 | CT VS HM | 0.55238641 | -0.0302947 | 1.135067 | 1.7373942 | 0.9701596 | 3.111383 | 1 |
| Huang Xingduan 2024 | CT VS MM | 0.59156983 | 0.2915984 | 0.8915412 | 1.8068226 | 1.338565 | 2.438886 | 1 |
| Huang Xingduan 2024 | CT VS TF | 0.29204302 | 0.0550677 | 0.5290183 | 1.3391606 | 1.056612 | 1.697265 | 1 |
| Huang Xingduan 2024 | CT VS WDM | 0.31047479 | 0.0216829 | 0.5992667 | 1.3640726 | 1.02192 | 1.820783 | 1 |
| Huang Xingduan 2024 | CT VS WN | 0.30200266 | 0.1292625 | 0.4747429 | 1.3525648 | 1.137989 | 1.607601 | 1 |
| Peng Jing 2022 | CT VS ACU | -0.0415789 | -0.4061093 | 0.3229515 | 0.95927364 | 0.6662374 | 1.381198 | 1 |
| Peng Jing 2022 | CT VS EHM | 0.02279344 | -0.5122735 | 0.5578604 | 1.0230552 | 0.5991319 | 1.746931 | 1 |
| Peng Jing 2022 | CT VS GIM | 0.10536052 | -0.1393911 | 0.3501121 | 1.1111111 | 0.8698878 | 1.419227 | 1 |
| Peng Jing 2022 | CT VS HM | 0.55238727 | -0.0302961 | 1.135071 | 1.7373957 | 0.9701582 | 3.111393 | 1 |
| Peng Jing 2022 | CT VS MM | 0.59157257 | 0.2915958 | 0.8915493 | 1.8068276 | 1.338562 | 2.438905 | 1 |
| Peng Jing 2022 | CT VS TF | 0.29204236 | 0.0550617 | 0.529023 | 1.3391597 | 1.056606 | 1.697273 | 1 |
| Peng Jing 2022 | CT VS WDM | 0.31047999 | 0.0216792 | 0.5992807 | 1.3640797 | 1.021916 | 1.820809 | 1 |
| Peng Jing 2022 | CT VS WN | 0.30200192 | 0.1292596 | 0.4747442 | 1.3525638 | 1.137985 | 1.607603 | 1 |
| Liang Guoyu 2025 | CT VS ACU | -0.03905828 | -0.3885302 | 0.3104137 | 0.96169466 | 0.6780527 | 1.363989 | 1 |
| Liang Guoyu 2025 | CT VS EHM | -0.1370759 | -0.6305726 | 0.3564208 | 0.87190404 | 0.5322869 | 1.428208 | 1 |
| Liang Guoyu 2025 | CT VS GIM | 0.10536052 | -0.1161956 | 0.3269166 | 1.1111111 | 0.890301 | 1.386686 | 1 |
| Liang Guoyu 2025 | CT VS HM | 0.55037777 | -0.0256921 | 1.126448 | 1.7339079 | 0.9746351 | 3.084679 | 1 |
| Liang Guoyu 2025 | CT VS MM | 0.5849888 | 0.3257069 | 0.8442707 | 1.7949709 | 1.385009 | 2.326281 | 1 |
| Liang Guoyu 2025 | CT VS TF | 0.29371642 | 0.0721596 | 0.5152733 | 1.3414035 | 1.074827 | 1.674096 | 1 |
| Liang Guoyu 2025 | CT VS WDM | 0.17434569 | 0.0845555 | 0.6332468 | 1.190467 | 1.0523486 | 1.883717 | 1 |
| Liang Guoyu 2025 | CT VS WN | 0.30427569 | 0.1439604 | 0.464591 | 1.3556427 | 1.154838 | 1.591363 | 1 |
| Hu Shuhan 2026 | CT VS ACU | -0.03905828 | -0.3885302 | 0.3104137 | 0.96169466 | 0.6780527 | 1.363989 | 1 |
| Hu Shuhan 2026 | CT VS EHM | 0.00087108 | -0.3298427 | 0.3315848 | 1.0008715 | 0.7190369 | 1.393174 | 1 |
| Hu Shuhan 2026 | CT VS GIM | 0.10536052 | -0.1161956 | 0.3269166 | 1.1111111 | 0.890301 | 1.386686 | 1 |
| Hu Shuhan 2026 | CT VS HM | 1.0986123 | -0.0864726 | 2.110752 | 3 | 0.900322 | 8.254446 | 1 |
| Hu Shuhan 2026 | CT VS MM | 0.5849888 | 0.3257069 | 0.8442707 | 1.7949709 | 1.385009 | 2.326281 | 1 |
| Hu Shuhan 2026 | CT VS TF | 0.29371642 | 0.0721596 | 0.5152733 | 1.3414035 | 1.074827 | 1.674096 | 1 |
| Hu Shuhan 2026 | CT VS WDM | 0.31229396 | 0.0358517 | 0.5887362 | 1.3665563 | 1.036502 | 1.80171 | 1 |
| Hu Shuhan 2026 | CT VS WN | 0.30427569 | 0.1439604 | 0.464591 | 1.3556427 | 1.154838 | 1.591363 | 1 |
| Hu Guanghui 2023 | CT VS ACU | -0.03905828 | -0.3885302 | 0.3104137 | 0.96169466 | 0.6780527 | 1.363989 | 1 |
| Hu Guanghui 2023 | CT VS EHM | 0.00087108 | -0.3298427 | 0.3315848 | 1.0008715 | 0.7190369 | 1.393174 | 1 |
| Hu Guanghui 2023 | CT VS GIM | 0.55037778 | -0.0256921 | 1.126448 | 1.7339079 | 0.9746351 | 3.084679 | 1 |
| Hu Guanghui 2023 | CT VS HM | 0.5849888 | -0.3257069 | 0.8442707 | 1.7949709 | 0.835009 | 2.326281 | 1 |
| Hu Guanghui 2023 | CT VS MM | 0.29371642 | 0.0721596 | 0.5152733 | 1.3414035 | 1.074827 | 1.674096 | 1 |
| Hu Guanghui 2023 | CT VS TF | 0.31229396 | 0.0358517 | 0.5887362 | 1.3665563 | 1.036502 | 1.80171 | 1 |
| Hu Guanghui 2023 | CT VS WDM | 0.30427569 | 0.1439604 | 0.464591 | 1.3556427 | 1.154838 | 1.591363 | 1 |

Leave-one-out sensitivity analysis of FPG in the subgroup analysis of patients with DPN (Table S147)

| **dropped_id** | **comparison** | **eff** | **lci** | **uci** | **connected** |
| --- | --- | --- | --- | --- | --- |
| Wang Jiawei 2023 | CT VS EHM | -0.34906533 | -1.953148 | 1.255017 | 1 |
| Wang Jiawei 2023 | CT VS GIM | -1.64 | -2.636377 | -0.6436231 | 1 |
| Wang Jiawei 2023 | CT VS HM | -0.25999999 | -1.12394 | 0.60394 | 1 |
| Wang Jiawei 2023 | CT VS MM | -1.5318791 | -2.268893 | -0.7948653 | 1 |
| Wang Jiawei 2023 | CT VS TF | -0.65850826 | -1.312435 | 0.0045817 | 1 |
| Wang Jiawei 2023 | CT VS WDM | -0.68921688 | -2.048634 | 0.6701999 | 1 |
| Wang Jiawei 2023 | CT VS WN | -0.75859835 | -1.455387 | 0.0618096 | 1 |
| Zhang Xiujun 2020 | CT VS EHM | -0.34891114 | -2.097873 | 1.400051 | 1 |
| Zhang Xiujun 2020 | CT VS GIM | -1.64 | -2.751921 | -0.5280787 | 1 |
| Zhang Xiujun 2020 | CT VS HM | -0.25999999 | -1.254984 | 0.7349845 | 1 |
| Zhang Xiujun 2020 | CT VS MM | -1.1410134 | -1.878617 | -0.4034098 | 1 |
| Zhang Xiujun 2020 | CT VS TF | -0.27000022 | -1.214841 | 0.674841 | 1 |
| Zhang Xiujun 2020 | CT VS WDM | -0.68911415 | -2.135087 | 0.7568583 | 1 |
| Zhang Xiujun 2020 | CT VS WN | -0.75188712 | -1.513654 | 0.0098797 | 1 |
| Xu Xiaowei 2025 | CT VS EHM | -0.34868907 | -2.287458 | 1.59008 | 1 |
| Xu Xiaowei 2025 | CT VS GIM | -1.64 | -2.899985 | -0.3800149 | 1 |
| Xu Xiaowei 2025 | CT VS HM | -1.1223981 | -1.928827 | 0.3159693 | 1 |
| Xu Xiaowei 2025 | CT VS MM | -0.70487581 | -1.550552 | -0.1408008 | 1 |
| Xu Xiaowei 2025 | CT VS TF | -0.68896619 | -2.251251 | 0.8733191 | 1 |
| Xu Xiaowei 2025 | CT VS WDM | -0.74514028 | -1.584911 | 0.0946308 | 1 |
| Zhang Suzhao 2024 | CT VS EHM | -0.34837432 | -2.528161 | 1.831412 | 1 |
| Zhang Suzhao 2024 | CT VS GIM | -1.64 | -3.084296 | -0.1957044 | 1 |
| Zhang Suzhao 2024 | CT VS HM | -0.25999999 | -1.616324 | 1.096324 | 1 |
| Zhang Suzhao 2024 | CT VS MM | -1.1056867 | -2.014771 | -0.1966018 | 1 |
| Zhang Suzhao 2024 | CT VS TF | -0.72170628 | -1.703075 | 0.2596623 | 1 |
| Zhang Suzhao 2024 | CT VS WDM | -0.68875649 | -2.40252 | 1.025007 | 1 |
| Zhang Suzhao 2024 | CT VS WN | -0.64556766 | -1.893512 | 0.6023766 | 1 |
| Hu Guanghui 2024 | CT VS EHM | -0.34841117 | -2.501369 | 1.804547 | 1 |
| Hu Guanghui 2024 | CT VS GIM | -1.64 | -3.063938 | -0.2160619 | 1 |
| Hu Guanghui 2024 | CT VS HM | -0.25999999 | -1.594625 | 1.074625 | 1 |
| Hu Guanghui 2024 | CT VS MM | -1.1072644 | -2.005144 | -0.2093846 | 1 |
| Hu Guanghui 2024 | CT VS TF | -0.72019851 | -1.686684 | 0.2462875 | 1 |
| Hu Guanghui 2024 | CT VS WDM | -0.68878104 | -2.385505 | 1.007943 | 1 |
| Hu Guanghui 2024 | CT VS WN | -0.7350211 | -1.840977 | 0.3709353 | 1 |
| Liu Xiangxiu 2012 | CT VS EHM | -0.3484263 | -2.490273 | 1.793421 | 1 |
| Liu Xiangxiu 2012 | CT VS GIM | -1.64 | -3.055496 | -0.2245036 | 1 |
| Liu Xiangxiu 2012 | CT VS HM | -0.25999999 | -1.585615 | 1.065615 | 1 |
| Liu Xiangxiu 2012 | CT VS MM | -0.9021066 | -2.099305 | -0.2950921 | 1 |
| Liu Xiangxiu 2012 | CT VS TF | -0.71955188 | -1.679964 | 0.2408606 | 1 |
| Liu Xiangxiu 2012 | CT VS WDM | -0.68879112 | -2.37847 | 1.000887 | 1 |
| Liu Xiangxiu 2012 | CT VS WN | -0.7397072 | -1.662974 | 0.1835593 | 1 |
| Li Shuomiao 2024 | CT VS EHM | -0.34891114 | -2.097873 | 1.400051 | 1 |
| Li Shuomiao 2024 | CT VS GIM | -1.64 | -2.751921 | -0.5280787 | 1 |
| Li Shuomiao 2024 | CT VS HM | -0.25999999 | -1.254984 | 0.7349845 | 1 |
| Li Shuomiao 2024 | CT VS MM | -1.1410134 | -1.878617 | -0.4034097 | 1 |
| Li Shuomiao 2024 | CT VS TF | -1.27 | -2.39485 | 0.1451497 | 1 |
| Li Shuomiao 2024 | CT VS WDM | -0.68911415 | -2.135087 | 0.7568583 | 1 |
| Li Shuomiao 2024 | CT VS WN | -0.75188712 | -1.513654 | 0.0098797 | 1 |
| Du Minzhen 2019 | CT VS EHM | -0.34846073 | -2.464802 | 1.767881 | 1 |
| Du Minzhen 2019 | CT VS GIM | -1.64 | -3.036095 | -0.2439046 | 1 |
| Du Minzhen 2019 | CT VS HM | -0.25999999 | -1.564878 | 1.044878 | 1 |
| Du Minzhen 2019 | CT VS MM | -1.1095184 | -1.992119 | -0.2269175 | 1 |
| Du Minzhen 2019 | CT VS TF | -0.71801601 | -1.66419 | 0.2281582 | 1 |
| Du Minzhen 2019 | CT VS WDM | -0.68881406 | -2.362348 | 0.98472 | 1 |
| Du Minzhen 2019 | CT VS WN | -0.81599087 | -1.879731 | 0.247749 | 1 |
| Dong Qingmei 2023 | —— | —— | —— | —— | disconnected |
| Wang Jiawei 2023 | CT VS EHM | -0.34864751 | -2.320916 | 1.623621 | 1 |
| Wang Jiawei 2023 | CT VS GIM | -1.64 | -2.925816 | -0.354184 | 1 |
| Wang Jiawei 2023 | CT VS HM | -0.25999999 | -1.446149 | 0.9261485 | 1 |
| Wang Jiawei 2023 | CT VS MM | -0.8147488 | -1.807728 | -0.17823 | 1 |
| Wang Jiawei 2023 | CT VS TF | -0.70773857 | -1.572039 | 0.1565621 | 1 |
| Wang Jiawei 2023 | CT VS WDM | -0.68893851 | -2.272047 | 0.8941696 | 1 |
| Wang Jiawei 2023 | CT VS WN | -0.74413497 | -1.597725 | 0.1094555 | 1 |
| Huang Xingduan 2024 | CT VS EHM | -1.64 | -2.900479 | 0.3795207 | 1 |
| Huang Xingduan 2024 | CT VS GIM | -0.25999999 | -1.418634 | -0.8986343 | 1 |
| Huang Xingduan 2024 | CT VS HM | -1.122345 | -1.929056 | 0.315634 | 1 |
| Huang Xingduan 2024 | CT VS MM | -0.70493249 | -1.550979 | -0.1411137 | 1 |
| Huang Xingduan 2024 | CT VS TF | -0.69000006 | -2.254239 | 0.8742386 | 1 |
| Huang Xingduan 2024 | CT VS WDM | -0.74512062 | -1.585158 | 0.0949167 | 1 |
| Hu Guanghui 2023 | CT VS EHM | -0.34868907 | -2.287458 | 1.59008 | 1 |
| Hu Guanghui 2023 | CT VS GIM | -0.25999999 | -1.418097 | -0.8980966 | 1 |
| Hu Guanghui 2023 | CT VS HM | -1.1223981 | -1.928827 | 0.3159693 | 1 |
| Hu Guanghui 2023 | CT VS MM | -0.70487581 | -1.550552 | -0.1408008 | 1 |
| Hu Guanghui 2023 | CT VS TF | -0.68896619 | -2.251251 | 0.8733191 | 1 |
| Hu Guanghui 2023 | CT VS WDM | -0.74514028 | -1.584911 | 0.0946308 | 1 |

Leave-one-out sensitivity analysis of 2hPG in the subgroup analysis of patients with DPN (Table S148)

| **dropped_id** | **comparison** | **eff** | **lci** | **uci** | **connected** |
| --- | --- | --- | --- | --- | --- |
| Tang Huazhen 2022 | CT VS EHM | -0.38926665 | -2.249181 | 1.470647 | 1 |
| Tang Huazhen 2022 | CT VS GIM | -1.9799999 | -2.562512 | -1.397488 | 1 |
| Tang Huazhen 2022 | CT VS HM | -0.30602951 | -0.7781357 | 0.1660767 | 1 |
| Tang Huazhen 2022 | CT VS MM | -2.0125058 | -2.807405 | -1.217607 | 1 |
| Tang Huazhen 2022 | CT VS TF | -1.1000001 | -1.766075 | -0.4339255 | 1 |
| Tang Huazhen 2022 | CT VS WDM | -1.2592964 | -3.050448 | 0.5318556 | 1 |
| Tang Huazhen 2022 | CT VS WN | -1.1448725 | -1.563811 | -0.7259346 | 1 |
| Zhang Xiujun 2020 | CT VS EHM | -0.38926665 | -2.249181 | 1.470647 | 1 |
| Zhang Xiujun 2020 | CT VS GIM | -1.9701095 | -2.384257 | -1.555962 | 1 |
| Zhang Xiujun 2020 | CT VS HM | -0.30602951 | -0.7781357 | 0.1660767 | 1 |
| Zhang Xiujun 2020 | CT VS MM | -2.0125058 | -2.807405 | -1.217607 | 1 |
| Zhang Xiujun 2020 | CT VS TF | -1.2592964 | -3.050448 | -0.5318555 | 1 |
| Zhang Xiujun 2020 | CT VS WDM | -1.1448725 | -1.56381 | 0.7259346 | 1 |
| Xu Xiaowei 2025 | CT VS EHM | -0.38926665 | -2.249181 | 1.470647 | 1 |
| Xu Xiaowei 2025 | CT VS GIM | -1.9701095 | -2.384257 | -1.555962 | 1 |
| Xu Xiaowei 2025 | CT VS HM | -0.05 | -0.774024 | 0.874024 | 1 |
| Xu Xiaowei 2025 | CT VS MM | -2.0125058 | -2.807405 | -1.217607 | 1 |
| Xu Xiaowei 2025 | CT VS TF | -1.1000001 | -1.766075 | -0.4339255 | 1 |
| Xu Xiaowei 2025 | CT VS WDM | -1.2592964 | -3.050448 | 0.5318556 | 1 |
| Xu Xiaowei 2025 | CT VS WN | -1.1448726 | -1.563811 | -0.7259346 | 1 |
| Zhang Suzhao 2024 | CT VS EHM | -0.38926665 | -2.249181 | 1.470647 | 1 |
| Zhang Suzhao 2024 | CT VS GIM | -1.9701095 | -2.384257 | -1.555962 | 1 |
| Zhang Suzhao 2024 | CT VS HM | -0.30602954 | -0.7781357 | 0.1660767 | 1 |
| Zhang Suzhao 2024 | CT VS MM | -2.0125058 | -2.807405 | -1.217607 | 1 |
| Zhang Suzhao 2024 | CT VS TF | -1.1000001 | -1.766075 | -0.4339255 | 1 |
| Zhang Suzhao 2024 | CT VS WDM | -1.2592964 | -3.050448 | 0.5318556 | 1 |
| Zhang Suzhao 2024 | CT VS WN | -0.90097391 | -1.620426 | -0.1815216 | 1 |
| Hu Guanghui 2024 | CT VS EHM | -0.38926665 | -2.249181 | 1.470647 | 1 |
| Hu Guanghui 2024 | CT VS GIM | -1.9701095 | -2.384257 | -1.555962 | 1 |
| Hu Guanghui 2024 | CT VS HM | -0.30602961 | -0.7781358 | 0.1660766 | 1 |
| Hu Guanghui 2024 | CT VS MM | -2.0125057 | -2.807405 | -1.217607 | 1 |
| Hu Guanghui 2024 | CT VS TF | -1.1000001 | -1.766075 | -0.4339255 | 1 |
| Hu Guanghui 2024 | CT VS WDM | -1.2592964 | -3.050448 | 0.5318555 | 1 |
| Hu Guanghui 2024 | CT VS WN | -1.1979028 | -1.676162 | -0.7196431 | 1 |
| Liu Xiangxiu 2012 | CT VS EHM | -0.38926665 | -2.249181 | 1.470647 | 1 |
| Liu Xiangxiu 2012 | CT VS GIM | -1.9701095 | -2.384257 | -1.555962 | 1 |
| Liu Xiangxiu 2012 | CT VS HM | -0.30602952 | -0.7781357 | 0.1660767 | 1 |
| Liu Xiangxiu 2012 | CT VS MM | -3.0299997 | -4.690474 | -1.369526 | 1 |
| Liu Xiangxiu 2012 | CT VS TF | -1.1000001 | -1.766075 | -0.4339255 | 1 |
| Liu Xiangxiu 2012 | CT VS WDM | -1.2592964 | -3.050448 | 0.5318556 | 1 |
| Liu Xiangxiu 2012 | CT VS WN | -1.1448725 | -1.563811 | -0.7259346 | 1 |
| Du Minzhen 2019 | CT VS EHM | -0.38926665 | -2.249181 | 1.470647 | 1 |
| Du Minzhen 2019 | CT VS GIM | -1.9701095 | -2.384257 | -1.555962 | 1 |
| Du Minzhen 2019 | CT VS HM | -0.30602954 | -0.7781357 | 0.1660767 | 1 |
| Du Minzhen 2019 | CT VS MM | -2.0125058 | -2.807405 | -1.217607 | 1 |
| Du Minzhen 2019 | CT VS TF | -1.1000001 | -1.766075 | -0.4339255 | 1 |
| Du Minzhen 2019 | CT VS WDM | -1.2592964 | -3.050448 | 0.5318555 | 1 |
| Du Minzhen 2019 | CT VS WN | -1.191883 | -1.635058 | -0.7487084 | 1 |
| Dong Qingmei 2023 | —— | —— | —— | —— | disconnected |
| Wang Jiawei 2023 | CT VS EHM | -0.38926665 | -2.249181 | 1.470647 | 1 |
| Wang Jiawei 2023 | CT VS GIM | -1.9701095 | -2.384257 | -1.555962 | 1 |
| Wang Jiawei 2023 | CT VS HM | -0.30602952 | -0.7781357 | 0.1660767 | 1 |
| Wang Jiawei 2023 | CT VS MM | -1.71 | -2.615384 | -0.8046159 | 1 |
| Wang Jiawei 2023 | CT VS TF | -1.1000001 | -1.766075 | -0.4339255 | 1 |
| Wang Jiawei 2023 | CT VS WDM | -1.2592964 | -3.050448 | 0.5318555 | 1 |
| Wang Jiawei 2023 | CT VS WN | -1.1448725 | -1.563811 | -0.7259346 | 1 |
| Huang Xingduan 2024 | CT VS EHM | -1.9701095 | -2.384257 | 1.555962 | 1 |
| Huang Xingduan 2024 | CT VS GIM | -0.30602951 | -0.7781357 | -0.1660767 | 1 |
| Huang Xingduan 2024 | CT VS HM | -2.0125058 | -2.807405 | 1.217607 | 1 |
| Huang Xingduan 2024 | CT VS MM | -1.1000001 | -1.766075 | -0.4339255 | 1 |
| Huang Xingduan 2024 | CT VS TF | -1.2599998 | -3.051916 | -0.5319162 | 1 |
| Huang Xingduan 2024 | CT VS WDM | -1.1448725 | -1.56381 | 0.7259346 | 1 |
| Hu Shuhan 2026 | CT VS EHM | -0.38926665 | -2.249181 | 1.470647 | 1 |
| Hu Shuhan 2026 | CT VS GIM | -1.9701095 | -2.384257 | -1.555962 | 1 |
| Hu Shuhan 2026 | CT VS HM | -0.48000002 | -1.056016 | 0.0960161 | 1 |
| Hu Shuhan 2026 | CT VS MM | -2.0125058 | -2.807405 | -1.217607 | 1 |
| Hu Shuhan 2026 | CT VS TF | -1.1000001 | -1.766075 | -0.4339255 | 1 |
| Hu Shuhan 2026 | CT VS WDM | -1.2592964 | -3.050448 | 0.5318556 | 1 |
| Hu Shuhan 2026 | CT VS WN | -1.1448726 | -1.563811 | -0.7259346 | 1 |
| Hu Guanghui 2023 | CT VS EHM | -0.38926665 | -2.249181 | 1.470647 | 1 |
| Hu Guanghui 2023 | CT VS GIM | -1.96 | -2.548927 | -1.371073 | 1 |
| Hu Guanghui 2023 | CT VS HM | -0.30602951 | -0.7781357 | 0.1660767 | 1 |
| Hu Guanghui 2023 | CT VS MM | -2.0125058 | -2.807405 | -1.217607 | 1 |
| Hu Guanghui 2023 | CT VS TF | -1.1000001 | -1.766075 | -0.4339255 | 1 |
| Hu Guanghui 2023 | CT VS WDM | -1.2592964 | -3.050448 | 0.5318555 | 1 |
| Hu Guanghui 2023 | CT VS WN | -1.1448725 | -1.56381 | -0.7259346 | 1 |

Grade assessment

GRADE assessment based on the common peroneal MNCV in the primary analysis of patients with DPN. (Table S149)

| **Comparison** | **Number of studies** | **Within-study bias** | **Reporting bias** | **Indirectness** | **Imprecision** | **Heterogeneity** | **Incoherence** | **Confidence rating** | **Reason(s) for downgrading** |
| --- | --- | --- | --- | --- | --- | --- | --- | --- | --- |
| ACU:MOXI | 2 | Some concerns | Low risk | No concerns | No concerns | Some concerns | Major concerns | Very low | ["Within-study bias","Heterogeneity","Incoherence"] |
| CT:MOXI | 7 | Some concerns | Low risk | No concerns | No concerns | Some concerns | No concerns | Low | ["Within-study bias","Heterogeneity"] |
| CT:MOXI+ACU | 3 | Some concerns | Low risk | No concerns | No concerns | Some concerns | Major concerns | Very low | ["Within-study bias","Heterogeneity","Incoherence"] |
| CT:MOXI+EHM | 3 | Some concerns | Low risk | No concerns | No concerns | No concerns | No concerns | Moderate | ["Within-study bias"] |
| CT:MOXI+IHM | 6 | Some concerns | Low risk | No concerns | No concerns | Some concerns | Major concerns | Very low | ["Within-study bias","Heterogeneity","Incoherence"] |
| CT:MOXI+MT | 2 | Some concerns | Low risk | No concerns | No concerns | Some concerns | Major concerns | Very low | ["Within-study bias","Heterogeneity","Incoherence"] |
| EHM:MOXI | 1 | Some concerns | Low risk | No concerns | Some concerns | No concerns | No concerns | Low | ["Within-study bias","Imprecision"] |
| EHM:MOXI+EHM | 2 | Some concerns | Low risk | No concerns | Some concerns | No concerns | No concerns | Low | ["Within-study bias","Imprecision"] |
| IHM:MOXI+IHM | 1 | Some concerns | Low risk | No concerns | Some concerns | Some concerns | Major concerns | Very low | ["Within-study bias","Imprecision","Heterogeneity","Incoherence"] |
| MOXI:MOXI+EHM | 2 | Some concerns | Low risk | No concerns | No concerns | Major concerns | No concerns | Very low | ["Within-study bias","Heterogeneity"] |
| ACU:CT | 0 | Some concerns | Low risk | No concerns | Some concerns | No concerns | Major concerns | Very low | ["Within-study bias","Imprecision","Incoherence"] |
| ACU:EHM | 0 | Some concerns | Low risk | No concerns | Some concerns | Some concerns | Major concerns | Very low | ["Within-study bias","Imprecision","Heterogeneity","Incoherence"] |
| ACU:IHM | 0 | Some concerns | Low risk | No concerns | Some concerns | No concerns | Major concerns | Very low | ["Within-study bias","Imprecision","Incoherence"] |
| ACU:MOXI+ACU | 0 | Some concerns | Low risk | No concerns | No concerns | No concerns | Major concerns | Very low | ["Within-study bias","Incoherence"] |
| ACU:MOXI+EHM | 0 | Some concerns | Low risk | No concerns | No concerns | No concerns | Major concerns | Very low | ["Within-study bias","Incoherence"] |
| ACU:MOXI+IHM | 0 | Some concerns | Low risk | No concerns | No concerns | Some concerns | Major concerns | Very low | ["Within-study bias","Heterogeneity","Incoherence"] |
| ACU:MOXI+MT | 0 | Some concerns | Low risk | No concerns | No concerns | Major concerns | Major concerns | Very low | ["Within-study bias","Heterogeneity","Incoherence"] |
| CT:EHM | 0 | Some concerns | Low risk | No concerns | Some concerns | Some concerns | Major concerns | Very low | ["Within-study bias","Imprecision","Heterogeneity","Incoherence"] |
| CT:IHM | 0 | Some concerns | Low risk | No concerns | Some concerns | No concerns | Major concerns | Very low | ["Within-study bias","Imprecision","Incoherence"] |
| EHM:IHM | 0 | Some concerns | Low risk | No concerns | Some concerns | No concerns | Major concerns | Very low | ["Within-study bias","Imprecision","Incoherence"] |
| EHM:MOXI+ACU | 0 | Some concerns | Low risk | No concerns | Major concerns | No concerns | Major concerns | Very low | ["Within-study bias","Imprecision","Incoherence"] |
| EHM:MOXI+IHM | 0 | Some concerns | Low risk | No concerns | Some concerns | No concerns | Major concerns | Very low | ["Within-study bias","Imprecision","Incoherence"] |
| EHM:MOXI+MT | 0 | Some concerns | Low risk | No concerns | Some concerns | No concerns | Major concerns | Very low | ["Within-study bias","Imprecision","Incoherence"] |
| IHM:MOXI | 0 | Some concerns | Low risk | No concerns | Some concerns | No concerns | Major concerns | Very low | ["Within-study bias","Imprecision","Incoherence"] |
| IHM:MOXI+ACU | 0 | Some concerns | Low risk | No concerns | No concerns | Some concerns | Major concerns | Very low | ["Within-study bias","Heterogeneity","Incoherence"] |
| IHM:MOXI+EHM | 0 | Some concerns | Low risk | No concerns | No concerns | Some concerns | Major concerns | Very low | ["Within-study bias","Heterogeneity","Incoherence"] |
| IHM:MOXI+MT | 0 | Some concerns | Low risk | No concerns | Major concerns | No concerns | Major concerns | Very low | ["Within-study bias","Imprecision","Incoherence"] |
| MOXI:MOXI+ACU | 0 | Some concerns | Low risk | No concerns | Some concerns | Some concerns | Major concerns | Very low | ["Within-study bias","Imprecision","Heterogeneity","Incoherence"] |
| MOXI:MOXI+IHM | 0 | Some concerns | Low risk | No concerns | Major concerns | No concerns | Major concerns | Very low | ["Within-study bias","Imprecision","Incoherence"] |
| MOXI:MOXI+MT | 0 | Some concerns | Low risk | No concerns | Major concerns | No concerns | Major concerns | Very low | ["Within-study bias","Imprecision","Incoherence"] |
| MOXI+ACU:MOXI+EHM | 0 | Some concerns | Low risk | No concerns | Some concerns | No concerns | Major concerns | Very low | ["Within-study bias","Imprecision","Incoherence"] |
| MOXI+ACU:MOXI+IHM | 0 | Some concerns | Low risk | No concerns | Some concerns | No concerns | Major concerns | Very low | ["Within-study bias","Imprecision","Incoherence"] |
| MOXI+ACU:MOXI+MT | 0 | Some concerns | Low risk | No concerns | Major concerns | No concerns | Major concerns | Very low | ["Within-study bias","Imprecision","Incoherence"] |
| MOXI+EHM:MOXI+IHM | 0 | Some concerns | Low risk | No concerns | Some concerns | Some concerns | Major concerns | Very low | ["Within-study bias","Imprecision","Heterogeneity","Incoherence"] |
| MOXI+EHM:MOXI+MT | 0 | Some concerns | Low risk | No concerns | Some concerns | No concerns | Major concerns | Very low | ["Within-study bias","Imprecision","Incoherence"] |
| MOXI+IHM:MOXI+MT | 0 | Some concerns | Low risk | No concerns | Major concerns | No concerns | Major concerns | Very low | ["Within-study bias","Imprecision","Incoherence"] |

GRADE assessment based on the median MNCV in the primary analysis of patients with DPN. (Table S150)

| **Comparison** | **Number of studies** | **Within-study bias** | **Reporting bias** | **Indirectness** | **Imprecision** | **Heterogeneity** | **Incoherence** | **Confidence rating** | **Reason(s) for downgrading** |
| --- | --- | --- | --- | --- | --- | --- | --- | --- | --- |
| ACU:MOXI | 1 | Some concerns | Low risk | No concerns | Some concerns | Some concerns | Major concerns | Very low | ["Within-study bias","Imprecision","Heterogeneity","Incoherence"] |
| CT:MOXI | 3 | Some concerns | Low risk | No concerns | Some concerns | Some concerns | No concerns | Very low | ["Within-study bias","Imprecision","Heterogeneity"] |
| CT:MOXI+ACU | 1 | Some concerns | Low risk | No concerns | No concerns | Some concerns | Major concerns | Very low | ["Within-study bias","Heterogeneity","Incoherence"] |
| CT:MOXI+EHM | 1 | Some concerns | Low risk | No concerns | No concerns | No concerns | No concerns | Moderate | ["Within-study bias"] |
| CT:MOXI+IHM | 7 | Some concerns | Low risk | No concerns | No concerns | Some concerns | Major concerns | Very low | ["Within-study bias","Heterogeneity","Incoherence"] |
| CT:MOXI+MT | 2 | Some concerns | Low risk | No concerns | No concerns | Some concerns | Major concerns | Very low | ["Within-study bias","Heterogeneity","Incoherence"] |
| EHM:MOXI+EHM | 1 | Some concerns | Low risk | No concerns | Some concerns | Some concerns | Major concerns | Very low | ["Within-study bias","Imprecision","Heterogeneity","Incoherence"] |
| MOXI:MOXI+EHM | 1 | Some concerns | Low risk | No concerns | No concerns | Some concerns | No concerns | Low | ["Within-study bias","Heterogeneity"] |
| ACU:CT | 0 | Some concerns | Low risk | No concerns | Major concerns | No concerns | Major concerns | Very low | ["Within-study bias","Imprecision","Incoherence"] |
| ACU:EHM | 0 | Some concerns | Low risk | No concerns | Major concerns | No concerns | Major concerns | Very low | ["Within-study bias","Imprecision","Incoherence"] |
| ACU:MOXI+ACU | 0 | Some concerns | Low risk | No concerns | No concerns | Some concerns | Major concerns | Very low | ["Within-study bias","Heterogeneity","Incoherence"] |
| ACU:MOXI+EHM | 0 | Some concerns | Low risk | No concerns | No concerns | Some concerns | Major concerns | Very low | ["Within-study bias","Heterogeneity","Incoherence"] |
| ACU:MOXI+IHM | 0 | Some concerns | Low risk | No concerns | No concerns | Major concerns | Major concerns | Very low | ["Within-study bias","Heterogeneity","Incoherence"] |
| ACU:MOXI+MT | 0 | Some concerns | Low risk | No concerns | Some concerns | Some concerns | Major concerns | Very low | ["Within-study bias","Imprecision","Heterogeneity","Incoherence"] |
| CT:EHM | 0 | Some concerns | Low risk | No concerns | Some concerns | No concerns | Major concerns | Very low | ["Within-study bias","Imprecision","Incoherence"] |
| EHM:MOXI | 0 | Some concerns | Low risk | No concerns | Some concerns | No concerns | Major concerns | Very low | ["Within-study bias","Imprecision","Incoherence"] |
| EHM:MOXI+ACU | 0 | Some concerns | Low risk | No concerns | Major concerns | No concerns | Major concerns | Very low | ["Within-study bias","Imprecision","Incoherence"] |
| EHM:MOXI+IHM | 0 | Some concerns | Low risk | No concerns | Some concerns | No concerns | Major concerns | Very low | ["Within-study bias","Imprecision","Incoherence"] |
| EHM:MOXI+MT | 0 | Some concerns | Low risk | No concerns | Some concerns | No concerns | Major concerns | Very low | ["Within-study bias","Imprecision","Incoherence"] |
| MOXI:MOXI+ACU | 0 | Some concerns | Low risk | No concerns | Some concerns | No concerns | Major concerns | Very low | ["Within-study bias","Imprecision","Incoherence"] |
| MOXI:MOXI+IHM | 0 | Some concerns | Low risk | No concerns | Some concerns | No concerns | Major concerns | Very low | ["Within-study bias","Imprecision","Incoherence"] |
| MOXI:MOXI+MT | 0 | Some concerns | Low risk | No concerns | Major concerns | No concerns | Major concerns | Very low | ["Within-study bias","Imprecision","Incoherence"] |
| MOXI+ACU:MOXI+EHM | 0 | Some concerns | Low risk | No concerns | Some concerns | No concerns | Major concerns | Very low | ["Within-study bias","Imprecision","Incoherence"] |
| MOXI+ACU:MOXI+IHM | 0 | Some concerns | Low risk | No concerns | Some concerns | No concerns | Major concerns | Very low | ["Within-study bias","Imprecision","Incoherence"] |
| MOXI+ACU:MOXI+MT | 0 | Some concerns | Low risk | No concerns | Some concerns | No concerns | Major concerns | Very low | ["Within-study bias","Imprecision","Incoherence"] |
| MOXI+EHM:MOXI+IHM | 0 | Some concerns | Low risk | No concerns | Some concerns | No concerns | Major concerns | Very low | ["Within-study bias","Imprecision","Incoherence"] |
| MOXI+EHM:MOXI+MT | 0 | Some concerns | Low risk | No concerns | Some concerns | No concerns | Major concerns | Very low | ["Within-study bias","Imprecision","Incoherence"] |
| MOXI+IHM:MOXI+MT | 0 | Some concerns | Low risk | No concerns | Major concerns | No concerns | Major concerns | Very low | ["Within-study bias","Imprecision","Incoherence"] |

GRADE assessment based on the common peroneal SNCV in the primary analysis of patients with DPN. (Table S151)

| **Comparison** | **Number of studies** | **Within-study bias** | **Reporting bias** | **Indirectness** | **Imprecision** | **Heterogeneity** | **Incoherence** | **Confidence rating** | **Reason(s) for downgrading** |
| --- | --- | --- | --- | --- | --- | --- | --- | --- | --- |
| ACU:MOXI+ACU | 1 | Some concerns | Low risk | No concerns | Major concerns | No concerns | Major concerns | Very low | ["Within-study bias","Imprecision","Incoherence"] |
| CT:MOXI | 6 | Some concerns | Low risk | No concerns | No concerns | Some concerns | No concerns | Low | ["Within-study bias","Heterogeneity"] |
| CT:MOXI+ACU | 1 | Some concerns | Low risk | No concerns | No concerns | Some concerns | Major concerns | Very low | ["Within-study bias","Heterogeneity","Incoherence"] |
| CT:MOXI+EHM | 1 | Some concerns | Low risk | No concerns | No concerns | No concerns | No concerns | Moderate | ["Within-study bias"] |
| CT:MOXI+IHM | 4 | Some concerns | Low risk | No concerns | No concerns | Some concerns | Major concerns | Very low | ["Within-study bias","Heterogeneity","Incoherence"] |
| CT:MOXI+MT | 1 | Some concerns | Low risk | No concerns | Some concerns | No concerns | Major concerns | Very low | ["Within-study bias","Imprecision","Incoherence"] |
| EHM:MOXI+EHM | 2 | Some concerns | Low risk | No concerns | Some concerns | Some concerns | Major concerns | Very low | ["Within-study bias","Imprecision","Heterogeneity","Incoherence"] |
| IHM:MOXI+IHM | 1 | Some concerns | Low risk | No concerns | Major concerns | No concerns | Major concerns | Very low | ["Within-study bias","Imprecision","Incoherence"] |
| MOXI:MOXI+EHM | 1 | Some concerns | Low risk | No concerns | No concerns | Some concerns | No concerns | Low | ["Within-study bias","Heterogeneity"] |
| ACU:CT | 0 | Some concerns | Low risk | No concerns | Some concerns | No concerns | Major concerns | Very low | ["Within-study bias","Imprecision","Incoherence"] |
| ACU:EHM | 0 | Some concerns | Low risk | No concerns | Some concerns | No concerns | Major concerns | Very low | ["Within-study bias","Imprecision","Incoherence"] |
| ACU:IHM | 0 | Some concerns | Low risk | No concerns | Major concerns | No concerns | Major concerns | Very low | ["Within-study bias","Imprecision","Incoherence"] |
| ACU:MOXI | 0 | Some concerns | Low risk | No concerns | Major concerns | No concerns | Major concerns | Very low | ["Within-study bias","Imprecision","Incoherence"] |
| ACU:MOXI+EHM | 0 | Some concerns | Low risk | No concerns | Major concerns | No concerns | Major concerns | Very low | ["Within-study bias","Imprecision","Incoherence"] |
| ACU:MOXI+IHM | 0 | Some concerns | Low risk | No concerns | Major concerns | No concerns | Major concerns | Very low | ["Within-study bias","Imprecision","Incoherence"] |
| ACU:MOXI+MT | 0 | Some concerns | Low risk | No concerns | Major concerns | No concerns | Major concerns | Very low | ["Within-study bias","Incoherence"] |
| CT:EHM | 0 | Some concerns | Low risk | No concerns | Some concerns | Some concerns | Major concerns | Very low | ["Within-study bias","Imprecision","Heterogeneity","Incoherence"] |
| CT:IHM | 0 | Some concerns | Low risk | No concerns | Some concerns | No concerns | Major concerns | Very low | ["Within-study bias","Imprecision","Incoherence"] |
| EHM:IHM | 0 | Some concerns | Low risk | No concerns | Some concerns | No concerns | Major concerns | Very low | ["Within-study bias","Imprecision","Incoherence"] |
| EHM:MOXI | 0 | Some concerns | Low risk | No concerns | Some concerns | No concerns | Major concerns | Very low | ["Within-study bias","Imprecision","Incoherence"] |
| EHM:MOXI+ACU | 0 | Some concerns | Low risk | No concerns | Major concerns | No concerns | Major concerns | Very low | ["Within-study bias","Imprecision","Incoherence"] |
| EHM:MOXI+IHM | 0 | Some concerns | Low risk | No concerns | Some concerns | No concerns | Major concerns | Very low | ["Within-study bias","Imprecision","Incoherence"] |
| EHM:MOXI+MT | 0 | Some concerns | Low risk | No concerns | Some concerns | No concerns | Major concerns | Very low | ["Within-study bias","Imprecision","Incoherence"] |
| IHM:MOXI | 0 | Some concerns | Low risk | No concerns | Major concerns | No concerns | Major concerns | Very low | ["Within-study bias","Imprecision","Incoherence"] |
| IHM:MOXI+ACU | 0 | Some concerns | Low risk | No concerns | Some concerns | No concerns | Major concerns | Very low | ["Within-study bias","Imprecision","Incoherence"] |
| IHM:MOXI+EHM | 0 | Some concerns | Low risk | No concerns | Some concerns | Some concerns | Major concerns | Very low | ["Within-study bias","Imprecision","Heterogeneity","Incoherence"] |
| IHM:MOXI+MT | 0 | Some concerns | Low risk | No concerns | Some concerns | No concerns | Major concerns | Very low | ["Within-study bias","Imprecision","Incoherence"] |
| MOXI:MOXI+ACU | 0 | Some concerns | Low risk | No concerns | Some concerns | No concerns | Major concerns | Very low | ["Within-study bias","Imprecision","Incoherence"] |
| MOXI:MOXI+IHM | 0 | Some concerns | Low risk | No concerns | Major concerns | No concerns | Major concerns | Very low | ["Within-study bias","Imprecision","Incoherence"] |
| MOXI:MOXI+MT | 0 | Some concerns | Low risk | No concerns | Major concerns | No concerns | Major concerns | Very low | ["Within-study bias","Imprecision","Incoherence"] |
| MOXI+ACU:MOXI+EHM | 0 | Some concerns | Low risk | No concerns | Some concerns | No concerns | Major concerns | Very low | ["Within-study bias","Imprecision","Incoherence"] |
| MOXI+ACU:MOXI+IHM | 0 | Some concerns | Low risk | No concerns | Major concerns | No concerns | Major concerns | Very low | ["Within-study bias","Imprecision","Incoherence"] |
| MOXI+ACU:MOXI+MT | 0 | Some concerns | Low risk | No concerns | Major concerns | No concerns | Major concerns | Very low | ["Within-study bias","Imprecision","Incoherence"] |
| MOXI+EHM:MOXI+IHM | 0 | Some concerns | Low risk | No concerns | Some concerns | Some concerns | Major concerns | Very low | ["Within-study bias","Imprecision","Heterogeneity","Incoherence"] |
| MOXI+EHM:MOXI+MT | 0 | Some concerns | Low risk | No concerns | Major concerns | No concerns | Major concerns | Very low | ["Within-study bias","Imprecision","Incoherence"] |
| MOXI+IHM:MOXI+MT | 0 | Some concerns | Low risk | No concerns | Major concerns | No concerns | Major concerns | Very low | ["Within-study bias","Imprecision","Incoherence"] |

GRADE assessment based on the median SNCV in the primary analysis of patients with DPN. (Table S152)

| **Comparison** | **Number of studies** | **Within-study bias** | **Reporting bias** | **Indirectness** | **Imprecision** | **Heterogeneity** | **Incoherence** | **Confidence rating** | **Reason(s) for downgrading** |
| --- | --- | --- | --- | --- | --- | --- | --- | --- | --- |
| ACU:MOXI | 1 | Some concerns | Low risk | No concerns | Some concerns | Some concerns | Major concerns | Very low | ["Within-study bias","Imprecision","Heterogeneity","Incoherence"] |
| CT:MOXI | 4 | Some concerns | Low risk | No concerns | No concerns | Some concerns | No concerns | Low | ["Within-study bias","Heterogeneity"] |
| CT:MOXI+ACU | 1 | Some concerns | Low risk | No concerns | Some concerns | Some concerns | Major concerns | Very low | ["Within-study bias","Imprecision","Heterogeneity","Incoherence"] |
| CT:MOXI+EHM | 2 | Some concerns | Low risk | No concerns | No concerns | No concerns | No concerns | Moderate | ["Within-study bias"] |
| CT:MOXI+IHM | 8 | Some concerns | Low risk | No concerns | No concerns | Some concerns | Major concerns | Very low | ["Within-study bias","Heterogeneity","Incoherence"] |
| EHM:MOXI+EHM | 2 | Some concerns | Low risk | No concerns | Some concerns | Some concerns | Major concerns | Very low | ["Within-study bias","Imprecision","Heterogeneity","Incoherence"] |
| MOXI:MOXI+EHM | 1 | Some concerns | Low risk | No concerns | Some concerns | No concerns | No concerns | Low | ["Within-study bias","Imprecision"] |
| ACU:CT | 0 | Some concerns | Low risk | No concerns | Some concerns | No concerns | Major concerns | Very low | ["Within-study bias","Imprecision","Incoherence"] |
| ACU:EHM | 0 | Some concerns | Low risk | No concerns | Some concerns | No concerns | Major concerns | Very low | ["Within-study bias","Imprecision","Incoherence"] |
| ACU:MOXI+ACU | 0 | Some concerns | Low risk | No concerns | Some concerns | Some concerns | Major concerns | Very low | ["Within-study bias","Imprecision","Heterogeneity","Incoherence"] |
| ACU:MOXI+EHM | 0 | Some concerns | Low risk | No concerns | Some concerns | Some concerns | Major concerns | Very low | ["Within-study bias","Imprecision","Heterogeneity","Incoherence"] |
| ACU:MOXI+IHM | 0 | Some concerns | Low risk | No concerns | Some concerns | Some concerns | Major concerns | Very low | ["Within-study bias","Imprecision","Heterogeneity","Incoherence"] |
| CT:EHM | 0 | Some concerns | Low risk | No concerns | Major concerns | No concerns | Major concerns | Very low | ["Within-study bias","Imprecision","Incoherence"] |
| EHM:MOXI | 0 | Some concerns | Low risk | No concerns | Major concerns | No concerns | Major concerns | Very low | ["Within-study bias","Imprecision","Incoherence"] |
| EHM:MOXI+ACU | 0 | Some concerns | Low risk | No concerns | Some concerns | No concerns | Major concerns | Very low | ["Within-study bias","Imprecision","Incoherence"] |
| EHM:MOXI+IHM | 0 | Some concerns | Low risk | No concerns | Some concerns | No concerns | Major concerns | Very low | ["Within-study bias","Imprecision","Incoherence"] |
| MOXI:MOXI+ACU | 0 | Some concerns | Low risk | No concerns | Major concerns | No concerns | Major concerns | Very low | ["Within-study bias","Imprecision","Incoherence"] |
| MOXI:MOXI+IHM | 0 | Some concerns | Low risk | No concerns | Some concerns | No concerns | Major concerns | Very low | ["Within-study bias","Imprecision","Incoherence"] |
| MOXI+ACU:MOXI+EHM | 0 | Some concerns | Low risk | No concerns | Major concerns | No concerns | Major concerns | Very low | ["Within-study bias","Imprecision","Incoherence"] |
| MOXI+ACU:MOXI+IHM | 0 | Some concerns | Low risk | No concerns | Major concerns | No concerns | Major concerns | Very low | ["Within-study bias","Imprecision","Incoherence"] |
| MOXI+EHM:MOXI+IHM | 0 | Some concerns | Low risk | No concerns | Major concerns | No concerns | Major concerns | Very low | ["Within-study bias","Imprecision","Incoherence"] |

GRADE assessment based on the TCSS in the primary analysis of patients with DPN. (Table S153)

| **Comparison** | **Number of studies** | **Within-study bias** | **Reporting bias** | **Indirectness** | **Imprecision** | **Heterogeneity** | **Incoherence** | **Confidence rating** | **Reason(s) for downgrading** |
| --- | --- | --- | --- | --- | --- | --- | --- | --- | --- |
| ACU:MOXI | 2 | Some concerns | Low risk | No concerns | Some concerns | No concerns | Major concerns | Very low | ["Within-study bias","Imprecision","Incoherence"] |
| ACU:MOXI+ACU | 1 | Some concerns | Low risk | No concerns | Some concerns | No concerns | Major concerns | Very low | ["Within-study bias","Imprecision","Incoherence"] |
| CT:MOXI | 3 | Some concerns | Low risk | No concerns | No concerns | Some concerns | No concerns | Low | ["Within-study bias","Heterogeneity"] |
| CT:MOXI+EHM | 2 | Some concerns | Low risk | No concerns | No concerns | No concerns | No concerns | Moderate | ["Within-study bias"] |
| CT:MOXI+IHM | 5 | Some concerns | Low risk | No concerns | No concerns | Some concerns | Major concerns | Very low | ["Within-study bias","Heterogeneity","Incoherence"] |
| EHM:MOXI | 1 | Some concerns | Low risk | No concerns | No concerns | Some concerns | No concerns | Low | ["Within-study bias","Heterogeneity"] |
| EHM:MOXI+EHM | 1 | Some concerns | Low risk | No concerns | Some concerns | No concerns | No concerns | Low | ["Within-study bias","Imprecision"] |
| MOXI:MOXI+EHM | 1 | Some concerns | Low risk | No concerns | No concerns | Some concerns | No concerns | Low | ["Within-study bias","Heterogeneity"] |
| ACU:CT | 0 | Some concerns | Low risk | No concerns | No concerns | Some concerns | Major concerns | Very low | ["Within-study bias","Heterogeneity","Incoherence"] |
| ACU:EHM | 0 | Some concerns | Low risk | No concerns | Some concerns | No concerns | Major concerns | Very low | ["Within-study bias","Imprecision","Incoherence"] |
| ACU:MOXI+EHM | 0 | Some concerns | Low risk | No concerns | No concerns | Some concerns | Major concerns | Very low | ["Within-study bias","Heterogeneity","Incoherence"] |
| ACU:MOXI+IHM | 0 | Some concerns | Low risk | No concerns | Some concerns | No concerns | Major concerns | Very low | ["Within-study bias","Imprecision","Incoherence"] |
| CT:EHM | 0 | Some concerns | Low risk | No concerns | Some concerns | No concerns | Major concerns | Very low | ["Within-study bias","Imprecision","Incoherence"] |
| CT:MOXI+ACU | 0 | Some concerns | Low risk | No concerns | Some concerns | Some concerns | Major concerns | Very low | ["Within-study bias","Imprecision","Heterogeneity","Incoherence"] |
| EHM:MOXI+ACU | 0 | Some concerns | Low risk | No concerns | Some concerns | No concerns | Major concerns | Very low | ["Within-study bias","Imprecision","Incoherence"] |
| EHM:MOXI+IHM | 0 | Some concerns | Low risk | No concerns | No concerns | Some concerns | Major concerns | Very low | ["Within-study bias","Heterogeneity","Incoherence"] |
| MOXI:MOXI+ACU | 0 | Some concerns | Low risk | No concerns | Some concerns | Some concerns | Major concerns | Very low | ["Within-study bias","Imprecision","Heterogeneity","Incoherence"] |
| MOXI:MOXI+IHM | 0 | Some concerns | Low risk | No concerns | No concerns | Some concerns | Major concerns | Very low | ["Within-study bias","Heterogeneity","Incoherence"] |
| MOXI+ACU:MOXI+EHM | 0 | Some concerns | Low risk | No concerns | Some concerns | Some concerns | Major concerns | Very low | ["Within-study bias","Imprecision","Heterogeneity","Incoherence"] |
| MOXI+ACU:MOXI+IHM | 0 | Some concerns | Low risk | No concerns | Some concerns | No concerns | Major concerns | Very low | ["Within-study bias","Imprecision","Incoherence"] |
| MOXI+EHM:MOXI+IHM | 0 | Some concerns | Low risk | No concerns | No concerns | Some concerns | Major concerns | Very low | ["Within-study bias","Heterogeneity","Incoherence"] |

GRADE assessment based on the markedly effective rate in the primary analysis of patients with DPN. (Table S154)

| **Comparison** | **Number of studies** | **Within-study bias** | **Reporting bias** | **Indirectness** | **Imprecision** | **Heterogeneity** | **Incoherence** | **Confidence rating** | **Reason(s) for downgrading** |
| --- | --- | --- | --- | --- | --- | --- | --- | --- | --- |
| ACU:MOXI | 1 | Some concerns | Low risk | No concerns | Some concerns | No concerns | No concerns | Low | ["Within-study bias","Imprecision"] |
| ACU:MOXI+ACU | 1 | Some concerns | Low risk | No concerns | No concerns | Some concerns | No concerns | Low | ["Within-study bias","Heterogeneity"] |
| CT:MOXI | 6 | Some concerns | Low risk | No concerns | No concerns | Some concerns | No concerns | Low | ["Within-study bias","Heterogeneity"] |
| CT:MOXI+ACU | 2 | Some concerns | Low risk | No concerns | No concerns | No concerns | No concerns | Moderate | ["Within-study bias"] |
| CT:MOXI+EHM | 2 | Some concerns | Low risk | No concerns | No concerns | No concerns | No concerns | Moderate | ["Within-study bias"] |
| CT:MOXI+IHM | 7 | Some concerns | Low risk | No concerns | No concerns | Some concerns | No concerns | Low | ["Within-study bias","Heterogeneity"] |
| EHM:MOXI+EHM | 2 | Some concerns | Low risk | No concerns | No concerns | No concerns | No concerns | Moderate | ["Within-study bias"] |
| MOXI:MOXI+EHM | 1 | Some concerns | Low risk | No concerns | Some concerns | No concerns | No concerns | Low | ["Within-study bias","Imprecision"] |
| ACU:CT | 0 | Some concerns | Low risk | No concerns | Major concerns | No concerns | No concerns | Very low | ["Within-study bias","Imprecision"] |
| ACU:EHM | 0 | Some concerns | Low risk | No concerns | Some concerns | No concerns | No concerns | Low | ["Within-study bias","Imprecision"] |
| ACU:MOXI+EHM | 0 | Some concerns | Low risk | No concerns | No concerns | Some concerns | No concerns | Low | ["Within-study bias","Heterogeneity"] |
| ACU:MOXI+IHM | 0 | Some concerns | Low risk | No concerns | Some concerns | Some concerns | No concerns | Very low | ["Within-study bias","Imprecision","Heterogeneity"] |
| CT:EHM | 0 | Some concerns | Low risk | No concerns | Some concerns | No concerns | No concerns | Low | ["Within-study bias","Imprecision"] |
| EHM:MOXI | 0 | Some concerns | Low risk | No concerns | Some concerns | No concerns | No concerns | Low | ["Within-study bias","Imprecision"] |
| EHM:MOXI+ACU | 0 | Some concerns | Low risk | No concerns | Some concerns | No concerns | No concerns | Low | ["Within-study bias","Imprecision"] |
| EHM:MOXI+IHM | 0 | Some concerns | Low risk | No concerns | Some concerns | No concerns | No concerns | Low | ["Within-study bias","Imprecision"] |
| MOXI:MOXI+ACU | 0 | Some concerns | Low risk | No concerns | Major concerns | No concerns | No concerns | Very low | ["Within-study bias","Imprecision"] |
| MOXI:MOXI+IHM | 0 | Some concerns | Low risk | No concerns | Some concerns | Some concerns | No concerns | Very low | ["Within-study bias","Imprecision","Heterogeneity"] |
| MOXI+ACU:MOXI+EHM | 0 | Some concerns | Low risk | No concerns | Some concerns | No concerns | No concerns | Low | ["Within-study bias","Imprecision"] |
| MOXI+ACU:MOXI+IHM | 0 | Some concerns | Low risk | No concerns | Some concerns | No concerns | No concerns | Low | ["Within-study bias","Imprecision"] |
| MOXI+EHM:MOXI+IHM | 0 | Some concerns | Low risk | No concerns | Some concerns | No concerns | No concerns | Low | ["Within-study bias","Imprecision"] |
